# Synthesis and structure-activity relationships for a new class of tetrahydronaphthalene amide inhibitors of *Mycobacterium tuberculosis*

**DOI:** 10.1016/j.ejmech.2021.114059

**Published:** 2022-02-05

**Authors:** Hamish S. Sutherland, Guo-Liang Lu, Amy S.T. Tong, Daniel Conole, Scott G. Franzblau, Anna M. Upton, Manisha U. Lotlikar, Christopher B. Cooper, Brian D. Palmer, Peter J. Choi, William A. Denny

**Affiliations:** a Auckland Cancer Society Research Centre, School of Medical Sciences, University of Auckland, Private Bag 92019, Auckland, 1142, New Zealand; b Maurice Wilkins Centre, University of Auckland, Private V, Auckland, 1142, New Zealand; c Institute for Tuberculosis Research, College of Pharmacy, University of Illinois at Chicago, 833 South Wood Street, Chicago, IL, 60612, USA; d Global Alliance for TB Drug Development, 40 Wall St, New York, 10005, USA

**Keywords:** Tetrahydronaphthalenes, Structure-activity relationships, Synthesis, Tuberculosis, ATP synthase

## Abstract

Drug resistant tuberculsosis (TB) is global health crisis that demands novel treatment strategies. Bacterial ATP synthase inhibitors such as bedaquiline and next-generation analogues (such as TBAJ-876) have shown promising efficacy in patient populations and preclinical studies, respectively, suggesting that selective targeting of this enzyme presents a validated therapeutic strategy for the treatment of TB. In this work, we report tetrahydronaphthalene amides (THNAs) as a new class of ATP synthase inhibitors that are effective in preventing the growth of *Mycobacterium tuberculosis (M.tb)* in culture. Design, synthesis and comprehensive structure-activity relationship studies for approximately 80 THNA analogues are described, with a small selection of compounds exhibiting potent (in some cases MIC_90_ <1 μg/mL) in vitro *M.tb* growth inhibition taken forward to pharmacokinetic and off-target profiling studies. Ultimately, we show that some of these THNAs possess reduced lipophilic properties, decreased hERG liability, faster mouse/human liver microsomal clearance rates and shorter plasma half-lives compared with bedaquiline, potentially addressing of the main concerns of persistence and phospholipidosis associated with bedaquiline.

## Highlights

• Tetrahydronaphthalene amides are novel inhibitors of *M.tb* in culture.
• Selective and potent inhibitors of the mycobacterial ATP synthase.
• Improved hERG liability, clearance and half-life compared to bedaquiline.

## Graphical abstract

**Figure:**
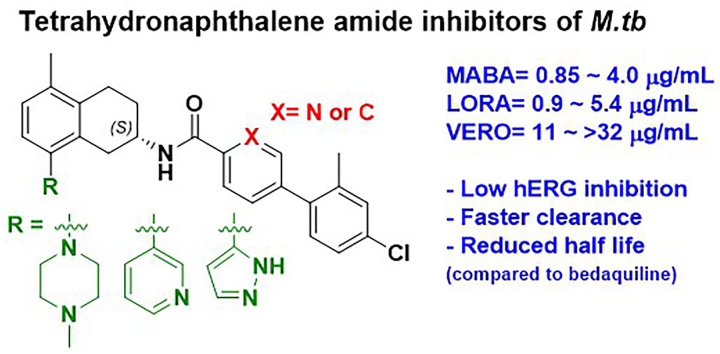

## 1 Introduction

The rise of drug-resistant tuberculosis (TB) in recent times has become a major global health problem [1], and this resurgence of such a major infectious disease has also provided an impetus for the development of new classes of drugs. These are aimed at a wide variety of mycobacterial targets, including the control of gene expression [2], inhibition of drug efflux pumps [3], and of proteins in the mycobacterial electron transport chain [4]. In particular, the spectacular success of the drug bedaquiline in treating multi-drug-resistant TB (MDR-TB) by inhibition of the mycobacterial enzyme ATP synthase has resulted in a largely curative regime (NIX-TB) [5] for this disease, despite bedaquiline's side effect of hERG channel inhibition. This success has led to the development of potentially safer analogues of bedaquiline [6] and a search for alternative classes of ATP synthase inhibitors.

**Fig. 1:**
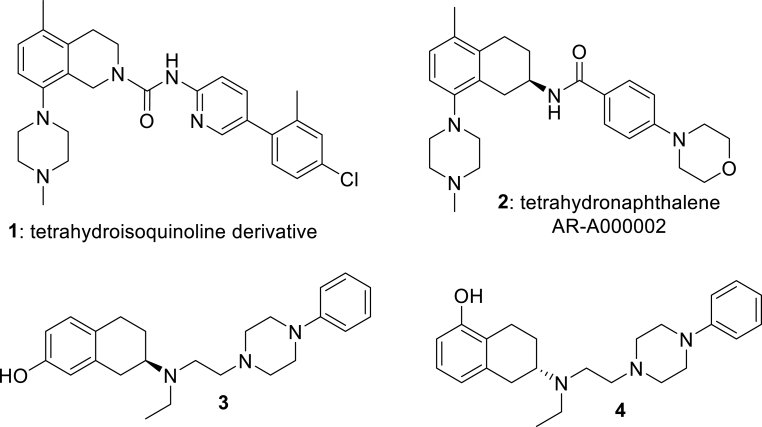
Known tetrahydronaphthalene-based drugs.

We recently reported [7] the synthesis and anti-mycobacterial structure–activity relationships (SARs) of a new class of *N*-substituted tetrahydroisoquinolines (THIQs, **1**; Fig. 1) as effective inhibitors of *Mycobacterium tuberculosis (M.tb)* ATP synthase enzyme and growth.

Building on this work, we now report synthesis and structure-activity-relationship (SAR) studies on a further novel class of tetrahydronaphthalene amide (THNA) derivatives as mycobacterial inhibitors. While the tetrahydronaphthalene amide unit is not widely featured in drugs, the derivative AR-A000002 (**2**; Fig. 1) has been studied as a selective and high affinity 5-hydroxytryptamine (5-HT_1B_) receptor antagonist [8]. It has been shown to be effective in animal models of depression and anxiety [9], and details of a chiral large-scale synthetic route have been published [10]. The related compounds **3** and **4** have demonstrated sub-μM affinity for cloned rat D2L and D3 receptors expressed in HEK293 cells [11]. While a handful of THNA derivatives have been recently reported as *M.tb* inhibitors [12,13] by targeting the cytochrome *bc*_1_ complex [14], the THNA unit did not form part of the central scaffold, and was not progressed into further studies. To the authors knowledge, this is the first systematic SAR report of 2-substituted THNA compounds as inhibitors of ATP synthase for the development of anti-TB drug candidates.

## 2 Results and discussion

### 2.1 Chemistry

**Fig. 2:**
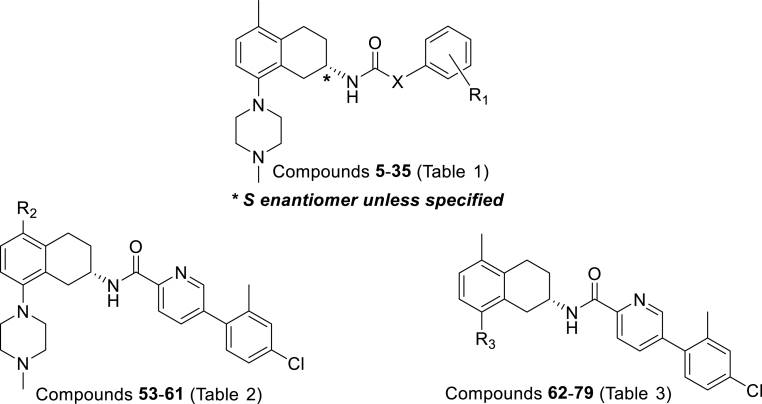
THNA analogues.

Initial synthesis of analogues (compounds **5–35**; Fig. 2) in Table 1 focused on synthesis of analogues with 5-methyl and 8-*N*-methylpiperidyl substituents for the tetrahydronaphthalene unit. This substitution pattern was on the basis of our previous work [7] which showed near-optimal anti-*M.tb* properties with the structurally related tetrahydroisoquinoline compounds (Fig. 1). With this constant THNA core in place, attention was initially turned to SAR studies on the optimal substituents for the heterocyclic linker and the terminal benzene ring and any chiral preferences for activity.

**Table 1:**
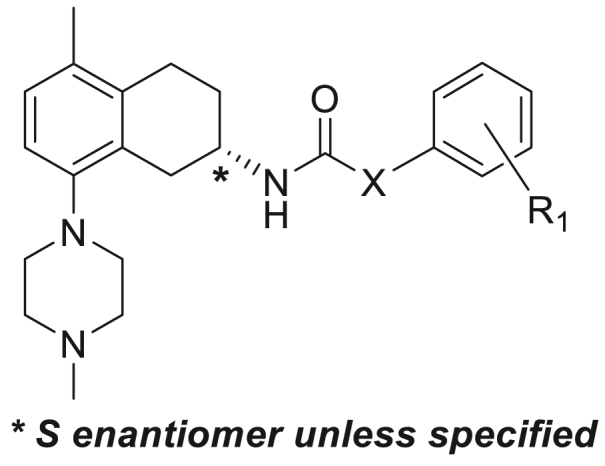
Structures and biological activity of 5-methyl-substituted tetrahydronaphthalenes.

No^a^	X	R_1_	Yield^b^	MIC_90_^c^ (μg/mL)		IC_50_^d^ (μg/mL)	clogP^e^
			%	MABA	LORA	VERO	
**5**		2-Me, 4-Cl	87	0.7	0.9	11	6.76
**5*R***		2-Me, 4-Cl	69	1.5	1.7	19	6.76
**6**		2-Me, 4-O(CH_2_)_2_OMe	85	0.90	5.2	11	5.75
**7**		2-Me, 4-Cl	77	1.9	3.7	20	6.55
**8**		3,5-diCF_3_	25	3.9	6.2	20	7.40
**9**		H	79	1.54	3.0	>32	6.02
**10**		2-Me, 4-F	81	0.94	5.8	21	6.66
**11**		2-Me, 4-Cl	62	1.9	3.8	15	7.73
**12**		2-F	79	0.99	3.1	24	5.96
**13**		3,5-diCF_3_	74	1.44	3.1	24	7.78
**14**		4-Cl	46	3.6	5.4	19	7.05
**15**		2,4-diMe	60	1.95	3.3	11	7.55
**16**		4-Cl	83	7.3	12	23	5.61
**17**		2,4-diMe	80	7.2	14	12	5.85
**18**		H	67	0.22	3.00	11	5.40
**19**		4-CF_3_	62	2.84	2.37	13	6.32
**19*R***		4-CF_3_	89	3.27	8.4	12	6.32
**20**		4-OCF_3_	71	1.91	1.80	12	6.53
**20*R***		4-OCF_3_	69	1.97	5.5	12	6.53
**21**		4-OCF_2_H	87	2.18	6.0	11	5.87
**21*R***		4-OCF_2_H	32	3.43	10	10	5.87
**22**		2-Me, 4-Cl	63	0.85	2.49	13	6.33
**22*R***		2-Me, 4-Cl	32	2.7	3.1	19	6.33
**23**		2-Me, 4-OCF_2_H	45	1.24	3.06	11	6.07
**23*R***		2-Me, 4-OCF_2_H	45	3.67	5.8	11	6.07
**24**		2-Me, 4-OCF_3_	66	1.72	2.57	11	6.73
**24*R***		2-Me, 4-OCF_3_	66	1.8	2.98	11	6.73
**25**		2,4-diCl	59	1.96	2.74	11	6.60
**26**		3-CF_3_, 4-Cl	88	1.74	1.8	13	7.03
**27**		3,5-diCF_3_	52	0.48	0.82	13	6.83
**28**		2-Me, 4-Cl	83	0.71	2.48	11	6.19
**28*R***		2-Me, 4-Cl	72	3.42	6.1	12	6.19
**29**		2-Me, 4-Cl	69	3.77	3.54	18	5.98
**29*R***		2-Me, 4-Cl	90	3.6	4.0	12	5.98
**30**		2-Me, 4-Cl	54	0.46	7.7	13	5.40
**30*R***		2-Me, 4-Cl	56	7.2	16	23	5.40
**31**		2-Me, 4-Cl	48	0.21	1.44	11	5.54
**32**		2-Me, 4-Cl	77	0.95	1.47	11	6.78
**32*R***		2-Me, 4-Cl	37	1.82	2.87	12	6.78
**33**		2-Me, 4-Cl	98	7.2	9.2	12	5.88
**34**		2-Me, 4-Cl	75	3.81	11	21	5.23
**34*R***		2-Me, 4-Cl	71	3.93	10	12	5.23
**35**		2-Me, 4-Cl	51	3.91	3.8	12	4.51
**36**		2-Me, 4-Cl	71	7.4	6.8	12	5.83
**37**		2-Me, 4-Cl	79	7.3	21	14	6.04
**38**		3,5-diCF_3_	59	3.79	3.7	12	6.90
**39**		2-Me, 4-Cl	99	7.1		22	5.19
**40**		3,5-diCF_3_	77	7.0		12	6.69
**41**		H	77	7.3	>32	>32	4.80
**42**		4-OMe	80	8.3	>32	15	4.70
**43**		4-F	74	3.51	>32	>32	5.11
**44**		4-CF_3_	75	14	19	25	5.98
**45**		4-Br	36	3.48	12	25	5.83
**46**		4-aza	64	>32	>32	>32	3.85
**47**		2-aza	62	13.5	>32	>32	3.85
**48**		2-Me, 4-Cl	65	1.92	5.9	21	6.18
**49**		3,5-diCF_3_	60	1.84	3.1	21	6.97
**50**		4-CF_3_	47	>32	27	23	4.17
**51**		2-Me, 4-Cl	70	3.69	6.1	22	5.67
**52**		3,5-diCF_3_	62	3.73	4.7	22	6.64

a Compounds are *S* enantiomers unless labelled *R*.

b Yield (%) in the coupling step in Scheme 1.

c MIC_90_ (μg/mL); minimum inhibitory concentration for 90% inhibition of growth of *M.tb* strain H37Rv, determined under aerobic (replicating; MABA) [15] or non-replicating (LORA) [16] conditions, determined at the Institute for Tuberculosis Research, University of Illinois at Chicago. Each value is the mean of at least two independent determinations.

d IC_50_ values (μg/mL) in green monkey kidney epithelial (VERO) cells as a measure of mammalian cell toxicity [17].

e clogP values are calculated using ChemDraw Professional v18.01 (CambridgeSoft).

Once the optimal linker and terminal groups had been identified from the study outlined in Table 1, efforts next focused on replacing the 5-methyl substituent on the tetrahydronaphthalene unit with groups of various steric bulk, electronic and lipophilic properties (Compounds **53**–**61**, Fig. 2, Table 2) while modification of the 8-*N*-methylpiperidyl motif with more weakly basic heterocycles and cyclic amines (compounds **62**–**79**, Fig. 2, Table 3) were carried out in attempt to address any potential hERG liabilities.

**Table 2:**
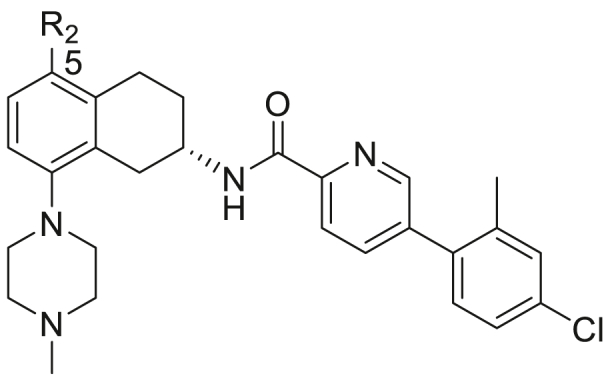
Structures and biological activity of (*S*) 5-substituted tetrahydronaphthalenes.

No	R_2_	Yield^a^ (%)	MIC_90_^b^ (μg/mL)		IC_50_^c^ (μg/mL)	clogP^d^	MR^e^ (cm^3^/mol)	δ_p_^f^
			MABA	LORA	VERO			
**5**	Me	75	0.8	1.5	11	6.76	6.88	−0.17
**53**	H	59	0.79	2.6	11	5.83	1.03	0.00
**54**	Ph	24	0.96	3.1	5.4	7.72	25.28	−0.01
**55**	4-^t^BuPh	28	1.0	1.1	11	9.55	44.95	n/a
**56**	Bn	21	1.0	1.4	11	7.90	31.17	−0.09
**57**	Br	71	0.61	2.8	11	6.86	9.86	+0.23
**58**	NO_2_	6	6.6	6.4	20	5.94	n/a	+0.78
**59**	NH(CH_2_)_2_NMe_2_	13	3.8	4.5	4.8	5.42	27.96	−0.70 (NHMe)
**60**	NMepip	30	6.6	7.5	13	5.73	31.09	−0.83 (NMe_2_)
**61**	CN	32	12	13	>32	5.66	6.74	+0.66

a Yield (%) in the coupling step in Scheme 1.

b MIC_90_ (μg/mL); minimum inhibitory concentration for 90% inhibition of growth of *M.tb* strain H37Rv, determined under aerobic (replicating; MABA [15] or non-replicating (LORA [16] conditions, determined at the Institute for Tuberculosis Research, University of Illinois at Chicago. Each value is the mean of at least two independent determinations.

c IC_50_ values (μg/mL) in green monkey kidney epithelial (VERO) cells as a measure of mammalian cell toxicity [17].

d clogP values, calculated using ChemDraw Professional v18.01 (CambridgeSoft).

e Molar refractivity parameter as a measurement of substituent size.

f Hammett constant as a measure of substituent electronic contribution to the aromatic system [18].

**Table 3:**
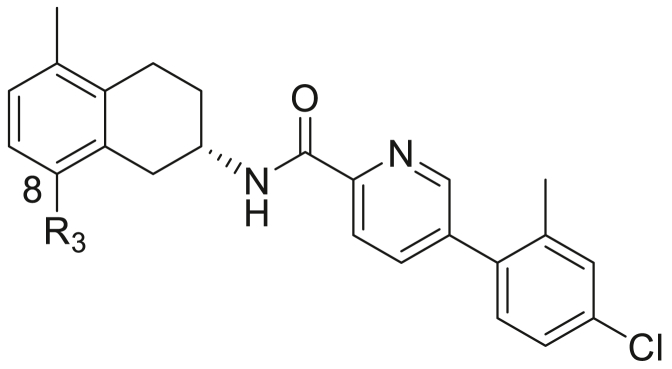
Structures and biological activity of 8-substituted tetrahydronaphthalenes.

No	R_3_	Yield^a^	MIC_90_^b^ (μg/mL)		IC_50_^c^ (μg/mL)	clogP^d^
		%	MABA	LORA	VERO	
**5**		55	0.8	1.5	11	6.76
**62**		100	1.0	1.5	11	5.76
**63**		86	0.90	0.91	10	7.18
**64**		78	1.9	1.2	13	6.77
**65**		63	0.74	1.2	5.8	6.51
**66**		82	0.93	1.7	9.9	6.96
**67**		84	>32	>32	>32	7.15
**68**		92	>32	>32	>32	5.77
**69**		32	>32	>32	>32	6.97
**70**		85	>32	>32	>32	8.37
**71**		88	1.9	>32	>32	6.70
**72**		62	3.7	4.3	>32	6.70
**73**		80	29	>32	>32	7.52
**74**		63	>32	>32	>32	7.42
**75**		22	>32	>32	>32	6.70
**76**		53	15	>32	>32	4.94
**77**		82	4.0	5.4	>32	6.51
**78**		10	15	14	>32	6.53
**79**		74	7.7	>32	>32	6.32

Footnotes forTable 3. As for Table 2.

The amide-linked compounds **5**–**35** of Table 1 (**A**; Scheme 1) were constructed by direct amide formation between the known [8,10] *R* or *S* enantiomers of 5-methyl-8-(4-methylpiperazin-1-yl)-1,2,3,4-tetrahydronaphthalen-2-amine (**80** or **80R**) with pre-assembled bicyclic side chain carboxylic acids that were either commercially available or prepared as outlined in the Experimental section.

**Scheme 1:**
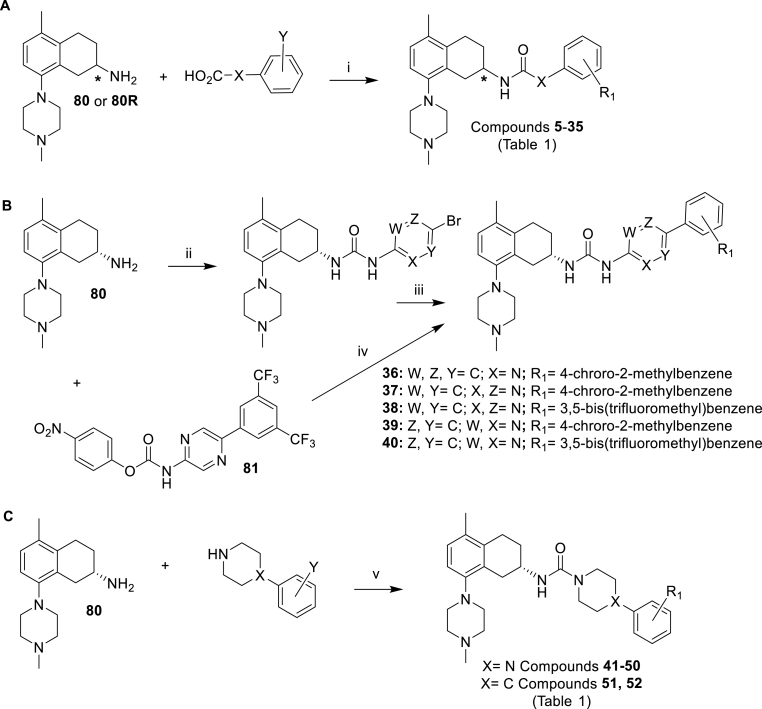
Synthesis of the compounds **5**–**35** (**A**), **36–40** (**B**) and **41–52** (**C**) of Table 1. *Reagents and conditions*: (i) HATU, DIPEA, DMF; (ii) Et_3_N, DCM, MeCN; (iii) Pd(dppf)Cl_2_, 2 M aq Na_2_CO_3_, PhMe:EtOH, 85 °C; (iv) Et_3_N, PhMe, 75 °C; (v) Et_3_N, 4-nitrophenyl chloroformate, DCM.

The urea-linked compounds **36**–**40** of Table 1 (**B**; Scheme 1) were prepared by the formation of an activated 4-nitrophenyl sidechain carbamate intermediate followed by coupling with tetrahydronaphthalen-2-amine **80**. This bromo-intermediate underwent further Suzuki cross-coupling with the appropriately substituted phenylboronic acids to furnish compounds **36**, **37**, **39** and **40**. This route proved to be a superior route to access urea-linked compounds as compound **38** which was accessed *via* reaction between preformed 4-nitrophenyl sidechain **81** and **80** gave a lower overall yield.

Urea-linked compounds **41–52** of Table 1 (**C**; Scheme 1) were prepared by the formation of piperizine/piperidine-benzene ring linker which were coupled to tetrahydronaphthalen-2-amine **80** using 4-nitrophenyl chloroformate and triethylamine in DCM.

**Scheme 2:**
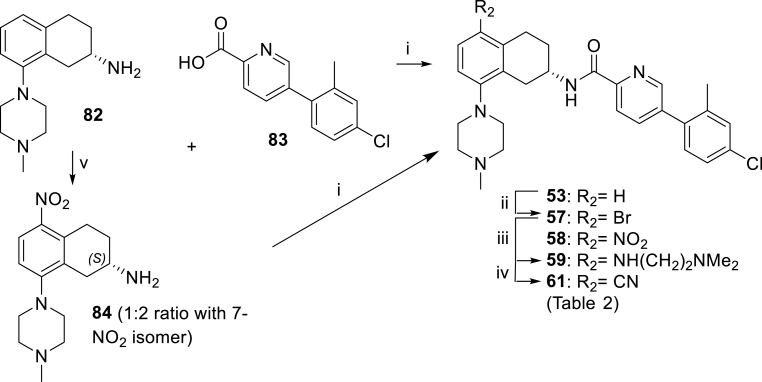
Synthesis of the compounds **53**, **57**, **58**, **59** and **61** of Table 2. *Reagents and conditions*: (i) HATU, DIPEA, DMF (**53**: 25%, **58**: 10% over 2 steps); (ii) NBS, DMF, 28 h (25%); (iii) H_2_N(CH_2_)_2_NMe_2_, Pd_2_(dba)_3_, NaOtBu, XPhos, toluene, 100 °C, 6 h (13%); (iv) Zn, Pd_2_dba_3_, (*o*-tol)_3_P, Zn(CN)_2_, 50 °C, 1 h (32%); (v) conc. sulphuric acid, nitric acid, 0 °C, 45 min.

**Scheme 3:**
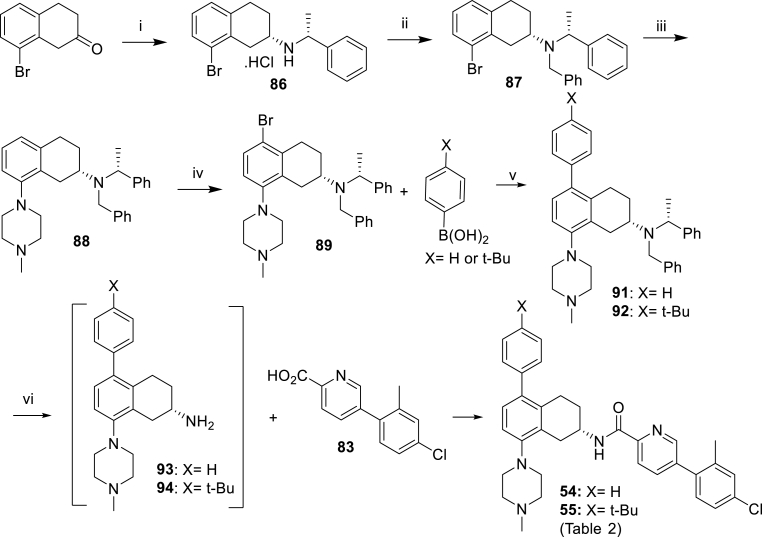
Synthesis of the compounds **54** and **55** of Table 2. *Reagents and conditions*: (i) *p*-TSA, (*R*)-*N*-ethylphenylamine, PhMe, 50 °C, 2 h then NaBH_4_, MeOH:*i*-PrOH (2:3), 70 °C, 18 h (37%); (ii) KI, MeCN, K_2_CO_3_, benzyl bromide, 150 °C, 27 h (82%); (iii) Pd(OAc)_2_, BINAP, *N*-methylpiperazine, PhMe, 80 °C, 30 min then NaOtBu, 100 °C, 3 h (64%); (iv) NBS, DMF, 72 h, (79%); (v) **90** or **91**, Pd(dppf)Cl_2_, 2 M aq K_2_CO_3_, PhMe:EtOH, 80 °C, 3 h (**91**: 30%, **92**: 49%); (vi) H_2_, 10% Pd/C, AcOH, MeOH, 60 psi then HATU, DIPEA, DMF (**54**: 41%, **55**: 28%).

**Scheme 4:**
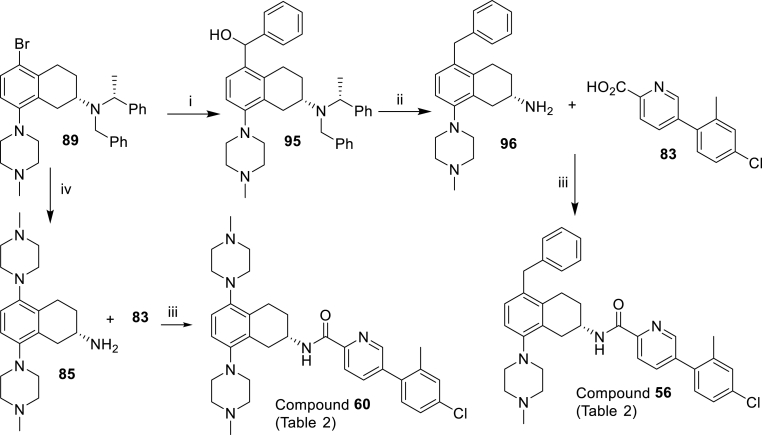
Synthesis of the compounds **56** and **60** of Table 2. *Reagents and conditions*: (i) *n*-BuLi, benzaldehyde −78 °C, 3 h (11%); (ii) TFA, Et_3_SiH, DCM, then H_2_, 10% Pd/C, MeOH (85%); (iii) HATU, DIPEA, DMF (**56**: 21%, **60**: 30%). (iv) Pd(dppf)Cl_2_, 2 M aq K_2_CO_3_, PhMe:EtOH, then H_2_, 10% Pd/C, AcOH, MeOH (25% over two steps).

Scheme 2, Scheme 3, Scheme 4 outline the synthesis of analogues with varied groups at the tetrahydronaphthalene 5-position in an attempt to explore SAR aspects such as steric bulk, electronics and lipophilicity at this site (Table 2). With carboxylic acid side chain **83** established as the favored side chain (rationale for selection discussed in detail in section 2.2.), Scheme 2 describes the formation of compound **53** via well-established amide coupling between **83** and tetrahydronaphthalenamine **82**. THNA analogue **53** could be further elaborated using bromination conditions to yield compound **57**. Nitration of tetrahydronaphthalenamine **82** afforded an inseparable mixture of 7-nitro and desired 5-nitro intermediate **84**. The mixture could be used crude for the next step after which the desired 5-nitro isomer could be isolated using silica chromatography to yield **58**. Bromo-functionality in **57** was used as a synthetic handle to access further diverse analogues. Buchwald-Hartwig amination reaction between **57** and *N,N*-dimethylethane-1,2-diamine required prolonged reaction time of 6 h to afford tethered amine **59** and cyanation reaction of **57** using zinc cyanide led to successful formation of nitrile **61**.

Tetrahydronaphthalene 5-position was further elaborated with para-substituted phenyl groups (compounds **54** and **55**, Scheme 3). Synthesis begins with 8-bromo-3,4-dihydronaphthalen-2(1*H*)-one, which undergoes reductive amination with (*R*)-*N*-ethylphenylamine to yield amine **86**, which is benzyl protected to yield **87** in 82% yield. Buchwald-Hartwig amination reaction with *N*-methylpiperazine gave **88** which underwent selective bromination on the tetrahydronaphthalene 5-position to yield bromide **89**. Suzuki coupling with the appropriate boronic acids gave tetrahydronaphthalenes with para-substituted phenyl groups **91** and **92**, which underwent deprotection followed by amide coupling gave analogues **54** and **55**.

Scheme 4 depicts the preparation of 5-benzyl **56** and more hydrophilic 5-(*N*-methylpiperidyl) **60** analogues. Commencing with common intermediate bromide **89** (prepared in Scheme 3), lithium-halogen exchange followed by quenching with benzaldehyde led to alcohol **95**, which was then reduced to **96**. Final amide coupling of amine **96** with acid **83** led to analogue **56**. Buchwald-Hartwig amination reaction between bromide **89** and *N*-methylpiperazine followed by reduction gave amine **85** and subsequent amide coupling with **83** furnished analogue **60**.

**Scheme 5:**
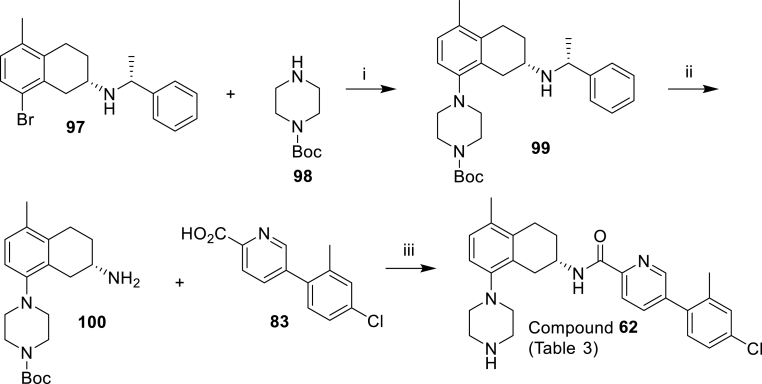
Synthesis of the compound **62** of Table 3. *Reagents and conditions*; (i) Pd_2_(dba)_3_, BINAP, PhMe, NaOtBu, 110 °C, 3 h (76%); (ii) H_2_, 10% Pd–C, MeOH, 50 psi, 24 h (53%); (iii) HATU, DIPEA, DMF, then TFA, DCM (100%).

**Scheme 6:**
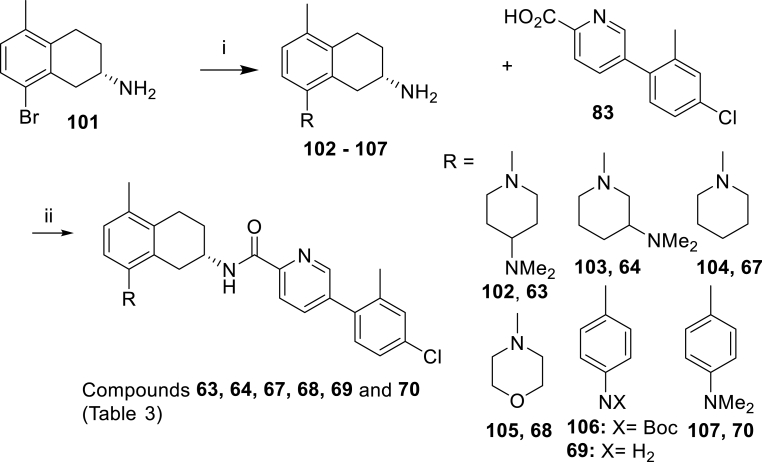
Synthesis of the compounds **63**, **64**, **67**, **68**, **69** and **70** of Table 3. *Reagents and conditions*; (i) Pd_2_(dba)_3_, BINAP, PhMe, NaOtBu, 110 °C, 3 h or Pd(dppf)Cl_2_, Na_2_CO_3_, toluene:MeOH:H_2_O, 105 °C, 5.5 h (**102**: 86%, **103**: 87%, **104**: 47%, **105**: 34%, **106**: 64%, **107**: 85%); (ii) HATU, DIPEA, DMF (**63**: 86%, **64**: 78%, **67**: 84%, **68**: 92%, **69**: 32%, **70**: 85%).

**Scheme 7:**
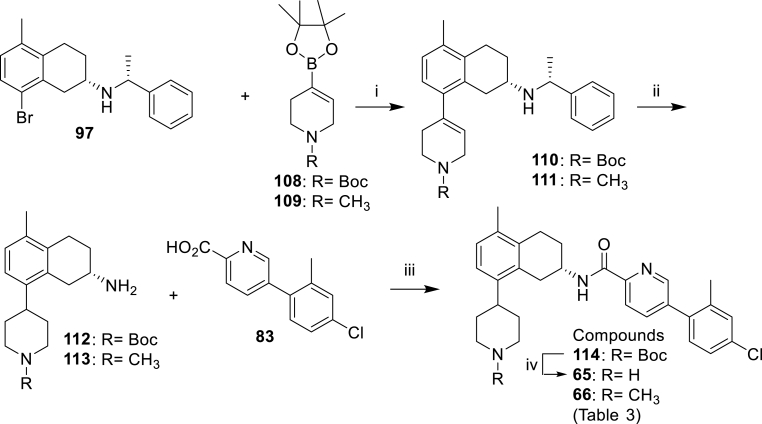
Synthesis of the compounds **65** and **66** of Table 3. *Reagents and conditions*: (i) Pd(dppf)Cl_2_, Na_2_CO_3_, toluene:MeOH:H_2_O, 105 °C, 4 h (**110**: 74%, **111**: 84%); (ii) H_2_, 10% Pd–C, MeOH, 50 psi, 24 h (**112**: 100%, **113**: 92%); (iii) HATU, DIPEA, DMF (**66**: 82%); (iv) TFA, DCM (**65**: 63% over two steps).

**Scheme 8:**
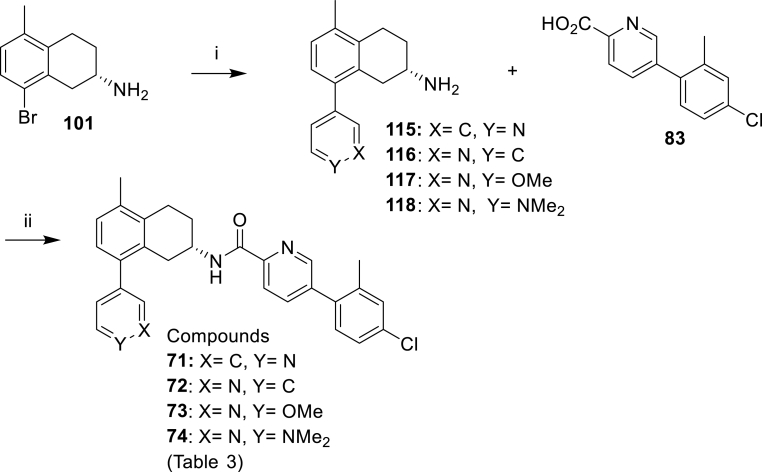
Synthesis of the compounds **71**–**74** of Table 3. *Reagents and conditions*: (i) Pd(dppf)Cl_2_, Na_2_CO_3_, toluene:MeOH:H_2_O, 100 °C, 2 h (**115**: 57%, **116**: 65%, **117**: 65%, **118**: 68%); (ii) HATU, DIPEA, DMF (**71**: 88%, **72**: 62%, **73**: 80%, **74**: 63%).

**Scheme 9:**
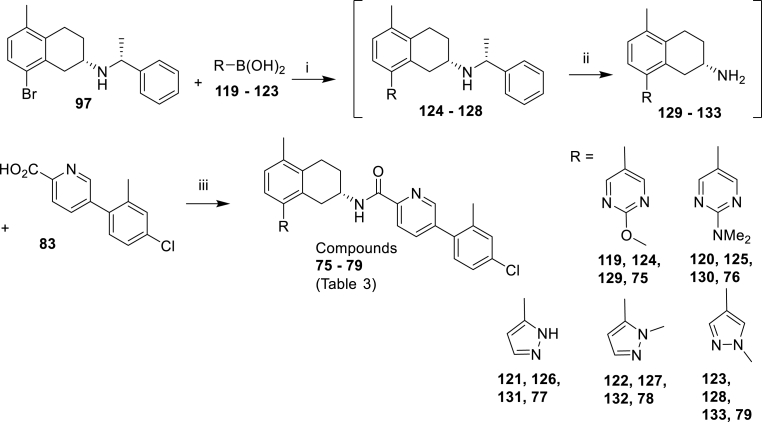
Synthesis of the compounds **75–79** of Table 3. *Reagents and conditions*:: (i) Pd(dppf)Cl_2_, Na_2_CO_3_, toluene:MeOH:H_2_O, 80 °C, 1.5 h (**124**: 35%, **125**: 51%, **126**: 78%, **127**: 75%, **128**: 43%); ii) 10% H_2_/10% Pd–C, AcOH/MeOH, 50 psi, 24 h; (iii) HATU, DIPEA, DMF (**75**: 22%, **76**: 53%, **77**: 46%, **78**: 10%, **79**: 74% over two steps).

To explore the effect of altering the pKa of the 8-methyl-piperazine unit on activity, a range of cyclic amines and heterocyclic amine analogues were prepared as described in Scheme 5, Scheme 6, Scheme 7, Scheme 8, Scheme 9. Scheme 5 reports the synthesis of the simple des-methyl piperidine analogue **62**, while Scheme 6 replaces it with a range of 6-membered aliphatic and aromatic ring systems (compounds **63, 64** and **66**–**70**). Scheme 7 outline the syntheses of the NH and NMe piperidinyl analogues (**65** and **66**) of the corresponding piperazinyl analogues **62** and **5** respectively. Scheme 8, Scheme 9 show the synthesis of compounds **71**–**79**, which have a range of six- and five-membered nitrogen heterocycles in place of the 8-methylpiperazine group.

### 2.2 Structure-activity relationships for the compounds of Table 1

Since the 5-hydroxytryptamine receptor antagonist **3** is a chiral compound, with much effort previously expended in its synthesis to obtain the pure *R* enantiomer for the production of compound **3** [10], we were initially interested to determine if the chirality was also of importance in our related series targeted against *M.tb*. To evaluate this, twelve sets of *R*/*S* enantiomer pairs of THNAs (Table 1) (compounds **5**, **19–24**, **28–30**, **32** and **34**) were prepared, bearing a representative range of both linker units X and terminal ring substituents R_1_, and their activities (as MIC_90_ values) were determined against both aerobic (replicating; MABA) and anaerobic (non-replicating; LORA) cultures of *M.tb*. (strain H37Rv). The results for this set show that the *S* enantiomers (average MIC_90_ values of 1.69 μM against MABA bacterial cultures and 3.04 μM against LORA cultures) were about twice as potent as the corresponding *R* enantiomers (MIC_90_s 3.88 μM for MABA and 6.62 μM for LORA cultures) confirming the *S* stereochemistry as the more potent eutomer (Supplementary Data- Fig. S1). In contrast, there were no significant differences between the isomers for mammalian toxicity, as measured by IC_50_ values in VERO cell cultures (Table 1).

Consequently, the remaining SAR studies were conducted using only the more active *S* enantiomers. For these 48 compounds, there is a modest correlation between the overall lipophilicity of the compounds and their potency of inhibition of bacterial growth in the MABA assay (logMIC_90_ values for inhibition of bacterial growth under aerobic (replicating) conditions) (equation (1)).

(1) Log(MIC_90_(MABA) = −0.24(±0.07)logP + 1.88

N = 48, R = 0.46, P = <0.001, F = 12.4

The general trend suggested that higher overall compound lipophilicity correlated with more potent bacterial survival inhibition (Supplementary Data- Fig. S2). This relationship of the antiproliferative potency of compounds against cultures of live *M.tb* being correlating with increasing overall lipophilicity of the agents has been previously observed across agents with differing mechanisms of action, and has been attributed to drug distribution, with lipophilic drugs needed to efficiently cross the very lipophilic cell walls of mycobacteria [[19], [20], [21]].

In the present case, the THNAs studied were comprised of a constant *N*-(5-methyl-8-(4-methylpiperazin-1-yl)-1,2,3,4-tetrahydronaphthalen-2-yl)acetamide unit with side chains made up of two variable but distinct “linker” and “terminal” regions. There was thus an opportunity to see whether variation in lipophilicity within these regions made differing contributions to MIC potency. The results (equation (2)) suggest that the lipophilicity of the linker unit does have a slightly larger influence on LORA potency than the lipophilicity of the terminal unit.

(2) Log(MIC_90_(LORA) = - 0.18(±0.05)clogP_LINKER_ – 0.11(±0.08)clogP_TERMINUS_ + 0.94

N = 48, R = 0.54, P = <0.001, F = 9.3

A wide variety of both aromatic and cyclic aliphatic linker groups were explored. The most effective linkers in terms of compound potency were the aromatic 1,4-benzene (compounds **5**, **6**) and the 1,4-(2-pyridyl (compounds **18**–**27**). The angular pyridine **29** was less effective, while the linear 2,5-pyrimidine (**30**) and the 2,5-pyrazine (**31**) were among the most active compounds in the set (especially when allowing for their considerably lower lipophilicity). In contrast, the linear aminopyridine (**36**), aminopyrazine (**37**) and aminopyrimidine (**39**) linkers were less effective. The 2,5-thiophene **32** was also among the most potent compounds, but a number of the five-membered aromatic linkers in compounds **33–35** (admittedly of much lower overall lipophilicity) were less effective. Finally, a series of 1,4-piperazines (**41–49**), *N*-piperazin-1-amines (**50–52**) and piperazin-1-amides (**53, 54**) were also less effective than the above compound with linear aromatic linkers.

A number of the different linker units were then paired with a variety of terminal units to evaluate the comparable efficacies of the latter. Comparison of compounds **5**, **7**, **11**, **15**, **22**, **28**, **30**, **48** with their counterparts bearing other terminal substitution show that the 2-methyl-4-chloro terminal unit consistently resulted in better activity.

Having established that the optimal sidechain in the THNAs in this study for anti-tubercular potency was the *S*-configuration with a 2-pyridyl linker group and a 2-methyl-4-chloro terminal ring, we then fixed this side chain and explored variations at the 5-position of the tetrahydronaphthalene unit in compounds **53–61** (Table 2).

Compounds **5**, 2-pyridyl analogues **18** and **27**, pyrimidine **30**, pyrazine **31** and thiophene **32**, which displayed unusually superior anti *M.tb* potency for their lipophilicity profiles (Supplementary Data- Figs. S2B and C) were subject to further study.

### 2.3 Structure-activity relationships for the compounds of Table 2

The results in Table 2 show there is considerable bulk tolerance at the 5-position of the tetrahydronaphthalene unit, with substituents varying in size from H (molar refractivity; MR 1.03; compound **53**) to benzyl (MR 31.2; compound **56**) having no effect on antibacterial potency. This is supported by the MIC/overall lipophilicity relationship for this group (equation (3)) being very similar to that of equation (1), suggesting that, for 5-substituted compounds the primary determinant of MIC potency is again overall lipophilicity, with the 5-substituent not making substantial target interactions.

(3) Log(MIC_90_(MABA) = −0.34(±0.14)logP + 2.52

N = 11 R = 0.64 P = 0.03 F = 6.2

This region did appear to be quite sensitive to changes in electronics contributed to the aromatic system (as measured by the Hammett constant), with both electron donating (**59** and **60**) and electron withdrawing (**58** and **61**) groups detrimental to anti-*M.tb* activity.

Finally, we evaluated variations in the 8-position of the tetrahydronaphthalene unit (Table 3).

### 2.4 Structure-activity relationships for the compounds of Table 3

Compounds **62–66**, bearing a range of cyclic aliphatic strong bases, all showed activity similar to the original *N*-methylpiperazine analogue **5**, whereas the weaker aliphatic (**67** and **68**) and aromatic (**69** and **70**) bases were inactive, despite having high overall lipophilicity. The concept that the pKa rather than the nature of the base is more important is reinforced by the pyridine-type bases **71–74**; the stronger bases **71** and **72** were active inhibitors of bacterial growth, whereas ones with weaker bases (**73–76**) were not, despite retaining high lipophilicity. Finally, the pyrazole analogues **77–79** had weak activity. Overall this suggests an important role for an ionisable base at the 8-position.

### 2.5 Preclinical evaluation

#### 2.5.1 Mammalian cell toxicity of THNA compounds

**Fig. 3:**
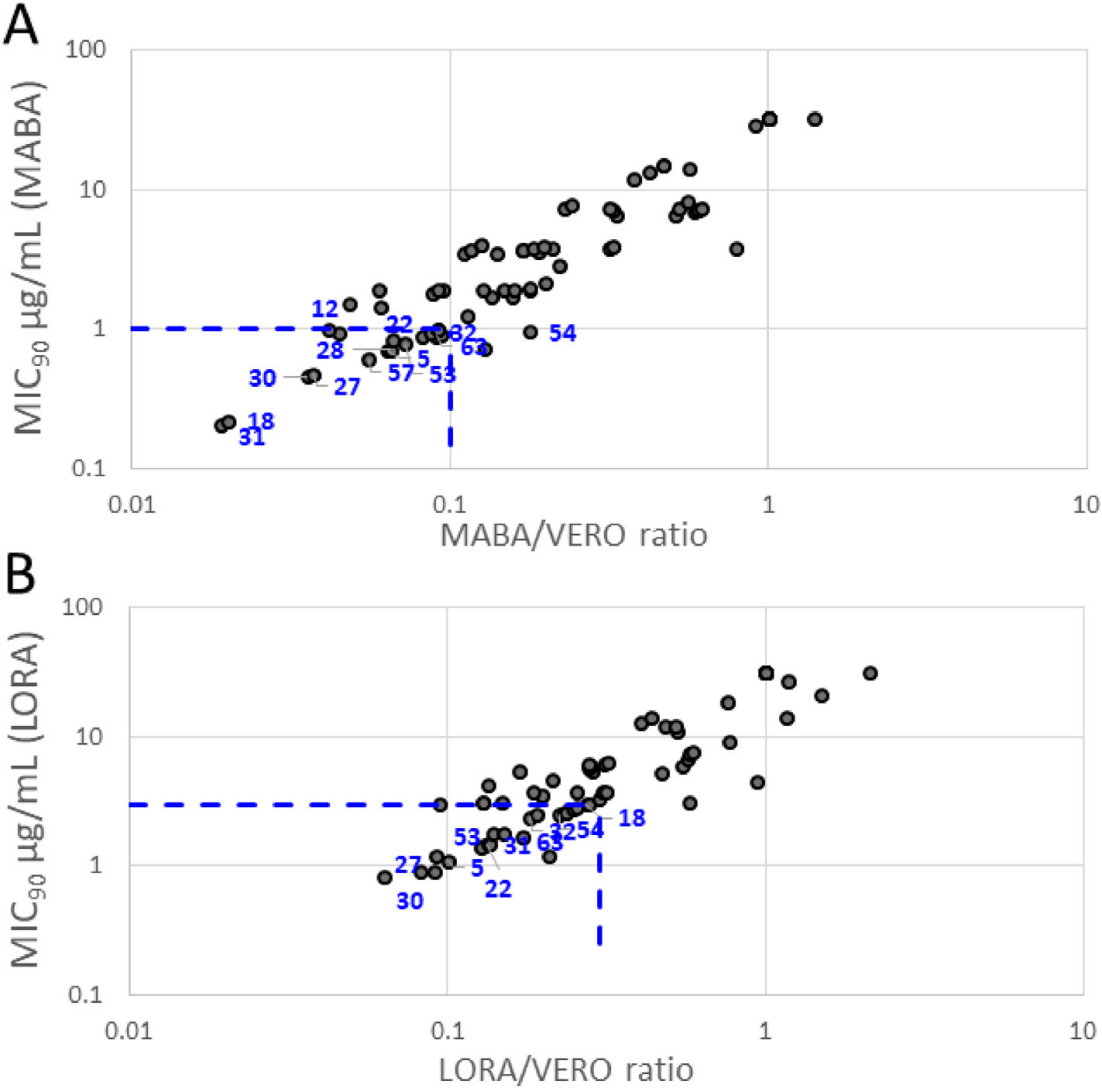
*M.tb* (MABA and LORA) vs mammalian toxicity (VERO) of THNA analogues. Compounds were selected for further evaluation based on those the displayed the most potent *M.tb* inhibition, and the best selectivity profiles with respect to their mammalian cytotoxicity, as measured by the ratio to MABA (**A**) or LORA (**B**) to VERO. Compounds to the left and below the blue dotted lines highlight the area of the graph display the most desirable criteria.

In order to assess safety and selectivity in humans, all compounds were also screened for mammalian cell toxicity in VERO (green monkey kidney cell) [17] cultures (Table 1, Table 2, Table 3). For the compounds of Table 1, Table 2 there was no clear overall relationship between their mammalian and mycobacterial cell potencies, but for compounds of Table 3, the weakly basic compounds **67–79** were much less toxic in both assays than those (**62–66**) bearing more basic side chains off the tetrahydronaphthalene chromophore. Based on the best MABA, LORA potencies, and superior selectivity profiles with respect to mammalian cell toxicity (based on the ratio of MABA or LORA inhibition vs VERO), a subset of THNAs were selected for further evaluation (Fig. 3).

#### 2.5.2 Inhibitory effects of THNA compounds on the mammalian hERG channel

As previously reported [23,24] the ability of the clinically-approved tuberculosis drug bedaquiline (BDQ) to inhibit the hERG cardiac potassium channel, with the concomitant risk of QT prolongation, has been a significant concern. Selected tetrahydronaphthalenes from Table 1, Table 2, Table 3 were also evaluated for hERG channel blockade (Table 4) at two fixed concentrations (0.3 and 1.0 μ g/mL), which translates to about 0.6 and 2 μ M respectively. By this assay, compounds **5**, **18**, **28**, **32**, and **43**, bearing an 8-[(4-*N*-methylpiperazinyl)] unit had hERG inhibitory properties similar to that of BDQ (single digit μ M), However, compounds **71**, **72**, **77** and **79** (Table 3) with aromatic heterocycles in that position, were much less hERG-inhibitory, suggesting that structural variations in this position are influential.

**Table 4:** hERG liability data for selected compounds of Table 1, Table 2, Table 3

No	hERG (% inhibition)^a^	
	0.3 μg/mL	1.0 μg/mL
**5**	74	90
**18**	81	93
**29**	49	88
**32**	55	74
**43**	70	86
**71**	0.6	5.8
**72**	6.2	13
**77**	2.1	1.4
**79**	0.0	5.0

a Inhibition of the hERG potassium channel (% inhibition at a drug concentration of either 0.3 or 1.0 μg/mL).

#### 2.5.3 Inhibitory effects of THNA compounds on ATP synthase enzymes

The primary anti-tubercular mechanism of action of BDQ is its high selectivity for mycobacterial (IC_50_ ∼ 10 nM) compared to human (IC_50_ > 200 μ M) ATP synthases [22]. Representative THNA compounds from Table 1, Table 2, Table 3 were evaluated (Table 5) for their inhibition of both *M.smegmatis* and human ATP synthase enzymes. Compounds had *M.smegmatis* inhibition IC_50_ values ranging from 1 to 5 μM, however they were less selective than BDQ over mammalian ATP synthase enzyme. Compounds **6** and **30** showed the best potency of 0.77 μM and 1 μM respectively, with good selectivity over the human enzyme (70 and 50 fold respectively).

**Table 5:** ATP synthase selectivity for selected compounds of Table 1, Table 2, Table 3

No	*M.smeg* ATP synth IC_50_^a^	Human ATP synth IC_50_^b^	Selectivity ratio^c^
**BDQ**	0.01	>200	>20000
**5**	2.4	18	7.5
**5R**	1.32	17.1	12.95
**6**	0.77	53.6	69.61
**18**	2.81	52.1	18.54
**20**	4.06	16	3.94
**22**	1.5	16	10.67
**22R**	2.18	17.3	7.94
**27**	3.68	12.3	3.34
**28**	2.27	29.7	13.08
**29**	1.9	15	7.89
**30**	1.0	50	50
**31**	0.89	18	20.22
**32**	1.9	15	7.89
**53**	2.7	25	9.26
**54**	2.4	4.7	1.96
**56**	2.0	8.9	4.45
**63**	4.6	16.1	3.5

a Inhibition (μ M) of the ATP synthase from *M.smegmatis*.

b Inhibition (μ M) of human ATP synthase.

c The selectivity for *M.smegmatis* over human ATP synthase was obtained from calculating the ratio (Human/*M.smegmatis)* of the IC_50_ values.

#### 2.5.4 Microsome stability of THNA compounds

Selected compounds from Table 1, Table 2, Table 3 were also evaluated for their stability against mouse and human liver microsome preparations, as a guide to their likely *in vivo* stability (Table 6). BDQ is known to be cleared very slowly, leading to a very long *in vivo* half-life in humans and concomitant concerns about long-term accumulation [22]. The results show that most of the tetrahydronaphthalenes evaluated had desirably faster clearance rates by both mouse liver microsomes (MLM) and human liver microsomes (HLM) than did BDQ.

**Table 6:** Microsomal stability data for selected compounds of Table 1, Table 2, Table 3

No	MLM Cl_int_^a^	HLM Cl_int_^a^
**BDQ**	3	7
**5**	195	18
**5*R***	38	20
**6**	811	53
**18**	219	31
**22**	603	49
**27**	600	24
**28**	933	66
**30**	262	43
**31**	870	82
**32**	100	29
**53**	118	57
**63**	263	39
**71**	51	65
**72**	68	57
**77**	49	28

a Clearance of compound by human or mouse liver microsomes (μl/min/mg protein).

Finally, a small number of representative compounds from Table 1, Table 2, Table 3 were evaluated for their PK properties in a mouse model following a single dose of drug at 100 mg/kg, and the results are shown in Table 7. BDQ is very lipophilic (clogP 7.25) which has been suggested to contribute to its very long terminal half-life in humans (164 days after 8 weeks of dosing) [23]. The THNAs evaluated were considerably less lipophilic than BDQ and had desirably shorter *in vivo* half-lives without compromising on total plasma drug exposure (AUC).

**Table 7:** *In vivo* PK data for representative tetrahydronaphthalenes.

No	Mouse PK (at 100 mg/kg PO)		clogP^c^
	AUC inf h∗μg/mL^a^	t½ (h)^b^	
**BDQ**	21	56	7.25
**5**	21	11	6.76
**18**	47	9	5.40
**32**	17	7	6.78
**71**	25	6.1	6.70
**72**	39	16	6.70
**77**	67	14	6.51
**79**	30	6.5	6.32

a Drug exposure (AUC) after a single dose of 100 mg/kg.

b Drug plasma half-life (hrs).

c Calculated using ChemDraw Professional, version 19.0.0.22.

## 3 Conclusions

In summary, we show that tetrahydronaphthalene amides, a new class of ATP synthase inhibitors are effective inhibitors of *M.tb* in culture. Systematic investigation of THNA structure-activity relationships revealed the optimal linker and terminal units, stereochemical requirements and tolerated positions for improvement of PK properties. The most effective linker units were 1,4-substituted benzene and 1,4-(2-pyridyl), while the most effective terminal unit was 2-methyl-4-chlorobenzene. The *S* enantiomers were about two-fold more potent than the corresponding *R* enantiomers against *M.tb* but had broadly equipotent toxicities in mammalian cell cultures. For the (larger group of) *S* enantiomers there was a modest but significant correlation between lipophilicity and their potency of *M.tb* inhibition. There was considerable bulk tolerance at the 5-position of the tetrahydronaphthalene unit, with substituents varying in size from hydrogen to benzyl having similar antibacterial potencies. An ionisable substituent at the 8-position of the tetrahydronaphthalene unit was important for anti-microbial activity. Results from a representative group of compounds also showed that weak aromatic bases (pyridines and pyrazoles) off the 8-positon of the tetrahydronaphthalene unit could desirably suppress the hERG inhibition activity. A smaller panel of these compounds (**5**, **18**, **32**, **71**, **72**, **77** and **79**) exhibited potent *M.tb* growth inhibition and were therefore taken forward to pharmacokinetic studies. Importantly, THNA analogues **72** and **77** exhibited the best overall profiles**,** with potent MABA and LORA values (3–5 μg/mL) and no mammalian cytotoxicities (>32 μg/mL), reduced lipophilicity, improved hERG liability (1–13% inhibition of the hERG potassium channel at 1 μg/mL), shorter half-life (14–16 h vs 56 h for bedaquiline) and desirably faster clearance rate compared to the clinically-approved tuberculosis drug bedaquiline. These findings show the potential of novel tetrahydronaphthalene amide-based compounds to be further developed into drug candidates for tuberculosis.

## 4 Experimental section

### 4.1 General information

Final products were analysed by reverse-phase HPLC (Alltima C18 5 μm column, 15 × 3.2 mm; Alltech Associated, Inc., Deerfield, IL) using an Agilent HP1100 equipped with a diode-array detector. Mobile phases were gradients of 80% CH_3_CN/20% H_2_O (v/v) in 45 mM NH_4_HCO_2_ at pH 3.5 and 0.5 mL/min. Purity was determined by monitoring at 330 ± 50 nm and was ≥95% for all final products. Melting points were determined on an Electrothermal 9100 melting point apparatus. NMR spectra were obtained on a Bruker Avance 400 spectrometer at 400 MHz for ^1^H. Low-resolution atmospheric pressure chemical ionization (APCI) mass spectra were measured for solutions on a ThermoFinnigan Surveyor MSQ mass spectrometer, connected to a Gilson autosampler. High resolution mass spectra were obtained using an Agilent G6530B Q-TOF spectrometer, and are reported for M + H.

***Representative synthesis of compounds that progressed to advanced testing****(For experimental procedures of all other final compounds, refer to the Supplementary Data*).

#### *4.1.1* *General procedure A: (S)-5-(4-Chloro-2-methylphenyl)-N-(5-methyl-8-(4-methylpiperazin-*1-yl*)-1,2,3,4-tetrahydronaphthalen-*2-yl*)picolinamide (****5****)*

A solution of 5-(4-chloro-2-methylphenyl)picolinic acid **83** (0.184 g, 0.746 mmol) in DMF (15 mL) was purged with nitrogen before DIPEA (0.48 ml, 2.8 mmol) was added to the reaction mixture. HATU (0.285 g, 0.750 mmol) was added and stirred for 15 min. (*S*)-5-methyl-8-(4-methylpiperazin-1-yl)-1,2,3,4-tetrahydronaphthalen-2-amine **80** (0.259 g, 0.679 mmol) was added to the reaction mixture and stirred at r.t. for 40 h. The reaction mixture was diluted with EtOAc and washed with water and 2 M NaOH solution. The organic layer was dried over anhydrous Na_2_SO_4_ and filtered through a pad of Celite. The solvent was removed to give the crude product, which was purified by silica column chromatography using MeOH (0–5% v/v) in EtOAc as eluent to give **5** (0.287 g, 87%) as a white foam. HPLC 99.1%. ^1^H NMR (CDCl_3_) δ 7.81 (ap d, *J* = 8.4 Hz, 2H), 7.35 (ap d, *J* = 8.4 Hz, 2H), 7.28 (d, *J* = 2.0 Hz, 1H), 7.23 (dd, *J* = 7.9, 1.7 Hz, 1H), 7.13 (d, *J* = 8.2 Hz, 1H), 7.04 (d, *J* = 8.0 Hz, 1H), 6.93 (d, *J* = 8.0 Hz, 1H), 6.13 (d, *J* = 7.8 Hz, 1H), 4.45–4.54 (m, 1H), 3.29 (dd, *J* = 16.5, 4.5 Hz, 1H), 2.85–2.94 (m, 4H), 2.81 (t, *J* = 6.7 Hz, 2H), 2.72 (dd, *J* = 16.5, 8.0 Hz, 1H), 2.57 (br, 4H), 2.35 (s, 3H), 2.23 (s, 3H), 2.22 (s, 3H), 2.22 (br, 1H), 1.89–1.99 (m, 1H). ^13^C NMR (CDCl_3_) δ 167.0, 150.0, 144.2, 139.5, 137.4, 135.2, 133.9, 133.7, 132.1, 131.0, 130.5, 129.7, 129.5, 128.2, 127.1, 126.2, 117.4, 55.8, 52.3, 46.3, 45.6, 31.9, 28.8, 25.6, 20.5, 19.6. HRMS calcd. for C_30_H_34_ClN_3_O: 487.2390, found 487.2405.

#### 4.1.2 (R)-5-(4-Chloro-2-methylphenyl)-N-(5-methyl-8-(4-methylpiperazin-1-yl)-1,2,3,4-tetrahydronaphthalen-2-yl)picolinamide (**5R**)

The title compound was obtained from (*R*)-5-methyl-8-(4-methylpiperazin-1-yl)-1,2,3,4-tetrahydronaphthalen-2-amine **80R** and **83** using the general procedure A to give **5*R*** (69%) as a white foam. HPLC 98.5%. ^1^H NMR (CDCl_3_) δ 7.81 (ap d, *J* = 8.4 Hz, 2H), 7.35 (ap d, *J* = 8.4 Hz, 2H), 7.28 (d, *J* = 2.0 Hz, 1H), 7.23 (ddd, *J* = 8.2, 2.0, 0.4 Hz, 1H), 7.13 (d, *J* = 8.2 Hz, 1H), 7.04 (d, *J* = 8.0 Hz, 1H), 6.93 (d, *J* = 8.0 Hz, 1H), 6.14 (d, *J* = 7.8 Hz, 1H), 4.45–4.54 (m, 1H), 3.29 (dd, *J* = 16.5, 4.6 Hz, 1H), 2.85–2.94 (m, 4H), 2.81 (t, *J* = 6.7 Hz, 2H), 2.72 (dd, *J* = 16.5, 8.0 Hz, 1H), 2.57 (br, 4H), 2.35 (s, 3H), 2.23 (s, 3H), 2.22 (s, 3H), 2.22 (br, 1H), 1.89–1.99 (m, 1H). ^13^C NMR (CDCl_3_) δ 167.0, 150.0, 144.2, 139.5, 137.4, 135.2, 133.9, 133.7, 132.1, 131.0, 130.5, 129.7, 129.5, 128.2, 127.1, 126.2, 117.4, 55.8, 52.3, 46.3, 45.6, 31.9, 28.8, 25.6, 20.5, 19.6. HRMS calcd. for C_30_H_34_ClN_3_O: 487.2390, found 487.2400.

#### 4.1.3 (S)-2-(4'-(2-Methoxyethoxy)-2′-methyl-[1,1′-biphenyl]-4-yl)-N-(5-methyl-8-(4-methylpiperazin-1-yl)-1,2,3,4-tetrahydronaphthalen-2-yl)acetamide (**6**)

**Figure:**
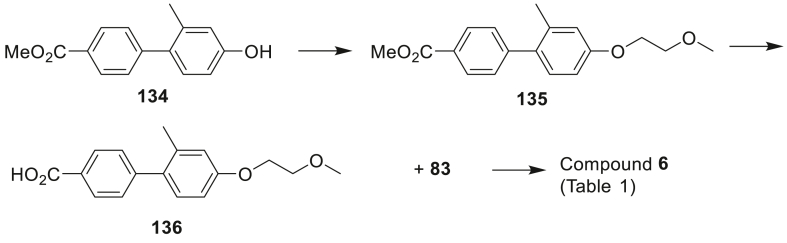

A mixture of (4-(methoxycarbonyl)phenyl)boronic acid (2.90 g, 16.1 mmol), 4-bromo-3-methylphenol (3.00 g, 16.0 mmol) and Cs_2_CO_3_ (10.5 g, 32.2 mmol) in anhydrous DMF (50 mL) was purged with nitrogen. Pd(dppf)Cl_2_.DCM (0.655 g, 0.80 mmol) was added and the mixture was heated to 75 °C under nitrogen in a sealable tube for 2 h. The mixture was partitioned between EtOAc and water, the organic fraction was dried and evaporated. Column chromatography (0–5% EtOAc:DCM) gave methyl 4ˈ-hydroxy-2ˈ-methyl-[1,1ˈ-biphenyl]-4-carboxylate (**134**) (2.89 g, 74%) as a tan solid. mp 166–167 °C. ^1^H NMR (CDCl_3_) δ 8.08 (ap d, *J* = 8.5 Hz, 2H), 7.39 (ap d, *J* = 8.5 Hz, 2H), 7.13 (d, *J* = 8.2 Hz, 1H), 6.80 (d, *J* = 2.6 Hz, 1H), 6.74 (dd, *J* = 8.2, 2.6 Hz, 1H), 5.22 (s, 1H), 3.96 (s, 3H), 2.25 (s, 3H). LRMS Found: [M+H] = 243.1.

Bromo-2-methoxyethane (0.47 mL, 50.0 mmol) was added to a mixture of **134** (1.018 g, 4.20 mmol) and K_2_CO_3_ (91.45 g, 10.5 mmol) in anhydrous DMF (20 mL). The mixture was stirred for 16 h, and then partitioned between EtOAc and water. The organic fraction was dried and evaporated, silica column chromatography (2:1 hexanes:DCM) gave methyl 4ˈ-(2-methoxyethoxy)-2ˈ-methyl-[1,1ˈ-biphenyl]-4-carboxylate (**135**) (1.136 g, 90%) as a colourless oil. ^1^H NMR (CDCl_3_) δ 8.06 (ap d, *J* = 8.5 Hz, 2H), 7.37 (ap d, *J* = 8.6 Hz, 2H), 7.15 (d, *J* = 8.4 Hz, 1H), 6.86 (d, *J* = 2.7 Hz, 1H), 6.83 (dd, *J* = 8.4, 2.5 Hz, 1H), 4.14–4.17 (m, 2H), 3.94 (s, 3H), 3.76–3.79 (m, 2H), 3.47 (s, 3H), 2.25 (s, 3H). LRMS Found: [M+H] = 301.1.

A solution of **135** (0.701 g, 2.45 mmol) in THF (20 mL), MeOH (20 mL) and water (10 mL) was treated with LiOH (0.76 g, 32 mmol). The solution was stirred at room temperature for 16 h, LiOH (0.76 g, 32 mmol) was added and stirring was continued for another 2 h. The solvent was evaporated and the residue was dissolved in water (50 mL), 2 M HCl was added until pH 2 was reached, the resulting white precipitate was filtered, washed with water and dried to give 4ˈ-(2-methoxyethoxy)-2ˈ-methyl-[1,1ˈ-biphenyl]-4-carboxylic acid (**136**) (0.655 g, 98%) as a white solid. mp 130–131 °C. ^1^H NMR ((CD_3_)_2_SO) δ 12.92 (bs, 1H), 7.98 (ap d, *J* = 8.4 Hz, 2H), 7.43 (ap d, *J* = 8.4 Hz, 2H), 7.15 (d, *J* = 8.4 Hz, 1H), 6.91 (d, *J* = 2.5 Hz, 1H), 6.86 (dd, *J* = 8.4, 2.5 Hz, 1H), 4.11–4.13 (m, 2H), 3.66–3.88 (m, 2H), 2.22 (s, 3H). LRMS Found: [M − H] = 285.1.

The title compound was obtained from (*S*)-5-methyl-8-(4-methylpiperazin-1-yl)-1,2,3,4-tetrahydronaphthalen-2-amine **80** and **136** using the general procedure A to give **6** (85%) as a white foam. ^1^H NMR (CDCl_3_) δ 7.79 (ap d, *J* = 8.4 Hz, 2H), 7.36 (ap d, *J* = 8.4 Hz, 2H), 7.12 (d, *J* = 8.4 Hz, 1H), 7.04 (d, *J* = 8.1 Hz, 1H), 6.92 (d, *J* = 8.0 Hz, 1H), 6.86 (d, *J* = 2.6 Hz, 1H), 6.82 (dd, *J* = 8.4, 2.6 Hz, 1H), 6.14 (d, *J* = 7.8 Hz, 1H), 4.47–4.51 (m, 1H), 4.14–4.17 (m, 2H), 3.76–3.78 (m, 2H), 3.47 (s, 3H), 3.28 (dd, *J* = 16.4, 4.6 Hz, 1H), 2.87 (m, 4H), 2.81 (t, *J* = 6.6 Hz, 2H), 2.71 (dd, *J* = 16.5, 8.1 Hz, 1H), 2.57 (bs, 4H), 2.35 (s, 3H), 2.24 (s, 3H), 2.22 (s, 4H), 2.21 (s, 3H), 1.93 (m, 1H). ^13^C NMR (CDCl_3_) δ 167.1, 158.5, 150.0, 145.1, 136.8, 135.2, 133.9, 133.2, 132.0, 130.9, 129.8, 129.8, 129.7, 128.2, 126.9, 117.4, 116.9, 112.1, 71.3, 67.5, 59.5, 55.9, 52.3, 46.4, 45.6, 31.9, 28.9, 25.6, 20.9, 19.6. HRMS calcd. for C_33_H_41_N_3_O_3_: 527.3148, found 527.3161.

#### 4.1.4 N-[(2S)-5-Methyl-8-(4-methyl-1-piperazinyl)-1,2,3,4-tetrahydro-2-naphthalenyl]-5-phenyl-2-pyridinecarboxamide (**18**)

The title compound was obtained from (*S*)-5-methyl-8-(4-methylpiperazin-1-yl)-1,2,3,4-tetrahydronaphthalen-2-amine **80** and 5-phenylpicolinic acid using the general procedure A to give **18** (67%) as a white foam. HPLC 98.1%. ^1^H NMR (CDCl_3_) δ 8.78 (dd, *J* = 2.2, 0.7 Hz, 1H), 8.30 (dd, *J* = 8.1, 0.7 Hz, 1H), 8.09 (d, *J* = 8.4 Hz, 1H), 8.04 (dd, *J* = 8.1, 2.3 Hz, 1H), 7.62–7.60 (m, 2H), 7.53–7.49 (m, 2H), 7.47–7.43 (m, 1H), 7.04 (d, *J* = 8.0 Hz, 1H), 6.92 (d, *J* = 8.0 Hz, 1H), 4.43–4.41 (m, 1H), 3.37 (dd, *J* = 16.5, 4.0 Hz, 1H), 3.00–2.94 (m, 2H), 2.86–2.81 (m, 4H), 2.70–2.50 (m, 5H), 2.35 (s, 3H), 2.29–2.25 (m, 1H), 2.21 (s, 3H), 1.93–1.82 (m, 1H). ^13^C NMR (CDCl_3_) δ 163.9, 149.9, 149.0, 146.7, 139.2, 137.3, 135.8, 135.3, 132.1, 130.2, 129.5, 128.9, 128.14, 127.5, 122.5, 117.4, 55.8, 52.2, 46.3, 45.5, 32.1, 29.3, 26.3, 19.6. HRMS calcd. for C_28_H_33_N_4_O 441.2639, found 441.2649.

#### 4.1.5 N-[(2S)-5-Methyl-8-(4-methyl-1-piperazinyl)-1,2,3,4-tetrahydro-2-naphthalenyl]-5-[4-(trifluoromethoxy)phenyl]-2-pyridinecarboxamide (**20**)

The title compound was obtained from (*S*)-5-methyl-8-(4-methylpiperazin-1-yl)-1,2,3,4-tetrahydronaphthalen-2-amine **80** and 5-(4-(trifluoromethoxy)phenyl)picolinic acid using the general procedure A to give **20** (71%) as a white foam. HPLC 96.7%.^1^H NMR (CDCl_3_) 8.75 (dd, *J* = 2.2, 0.6 Hz, 1H), 8.31 (dd, *J* = 8.0, 0.7 Hz, 1H), 8.08 (br d, *J* = 8.0 Hz, 1H), 8.02 (dd, *J* = 8.1, 2.3 Hz, 1H), 7.64 (d*, J* = 8.7 Hz, 2H), 7.36 (d, *J* = 8.7 Hz, 2H), 7.05 (d, *J* = 8.0 Hz, 1H), 6.93 (d, *J* = 8.0 Hz. 1H), 4.43 (m, 1H), 3.38 (m, 1H), 3.00–2.50 (m, 10H), 2.34 (s, 3H), 2.30 (m, 2H). 2.22 (s, 3H), 1.90 (m, 1H). ^13^C NMR (CDCl_3_) δ 163.7, 149.9, 149.4, 146.6, 137.9, 135.9, 135.8, 135.3, 132.1, 130.1, 128.9, 128.1, 122.6, 121.9, 117.4, 55.8, 52.2, 46.3, 45.5, 32.1, 29.9, 26.2, 19.6. HRMS calcd. for C_29_H_32_F_3_N_4_O_2_ 525.2472, found 525.2457.

#### 4.1.6 (S)-5-(4-chloro-2-methylphenyl)-N-(5-methyl-8-(4-methylpiperazin-1-yl)-1,2,3,4-tetrahydronaphthalen-2-yl)picolinamide (**22**)

The title compound was obtained from (*S*)-5-methyl-8-(4-methylpiperazin-1-yl)-1,2,3,4-tetrahydronaphthalen-2-amine **80** and 5-(4-chloro-2-methylphenyl)picolinic acid **83** using the general procedure A to give **22** (55%) as a white foam. HPLC 98.7%. ^1^H NMR (CDCl_3_) δ 8.49 (dd, *J* = 2.0, 0.8 Hz, 1H), 8.29 (dd, *J* = 8.4, 0.8 Hz, 1H), 8.09 (d, *J* = 8.4 Hz, 1H), 7.79 (dd, *J* = 8.0, 2.0 Hz, 1H), 7.27–7.32 (m, 2H), 7.15 (d, *J* = 8.4 Hz, 1H), 7.04 (d, *J* = 8.0 Hz, 1H), 6.93 (d, *J* = 8.0 Hz, 1H), 4.37–4.47 (m, 1H), 3.38 (dd, *J* = 16.4, 4.0 Hz, 1H), 2.94–2.99 (m, 2H), 2.76–2.86 (m, 4H), 2.67 (dd, *J* = 16.4, 8.8 Hz, 1H), 2.56 (br, 4H), 2.35 (s, 3H), 2.28 (br, 1H), 2.27 (s, 3H), 2.17 (s, 3H), 1.85–1.95 (m, 1H). ^13^C NMR (CDCl_3_) δ 163.7, 149.9, 149.1, 148.3, 139.1, 138.0, 137.7, 136.0, 135.3, 134.6, 132.1, 131.2, 130.8, 130.2, 128.1, 126.6, 122.1, 117.4, 55.8, 52.3, 46.3, 45.5, 32.1, 29.3, 26.2, 20.5, 19.6. HRMS calcd. for C_29_H_33_ClN_4_O: 488.2343, found 488.2352.

#### 4.1.7 (R)-5-(4-chloro-2-methylphenyl)-N-(5-methyl-8-(4-methylpiperazin-1-yl)-1,2,3,4-tetrahydronaphthalen-2-yl)picolinamide (**22R**)

The title compound was obtained from (*R*)-5-methyl-8-(4-methylpiperazin-1-yl)-1,2,3,4-tetrahydronaphthalen-2-amine **80R** and 5-(4-chloro-2-methylphenyl)picolinic acid **83** using the general procedure A to give **22R** (32%) as a white foam. HPLC 99.6%. ^1^H NMR (CDCl_3_) δ ^1^H NMR (CDCl_3_) δ 8.49 (dd, *J* = 2.0, 0.4 Hz, 1H), 8.30 (dd, *J* = 8.4, 0.8 Hz, 1H), 8.09 (d, *J* = 8.4 Hz, 1H), 7.79 (dd, *J* = 8.0, 2.4 Hz, 1H), 7.27–7.32 (m, 2H), 7.15 (d, *J* = 8.4 Hz, 1H), 7.04 (d, *J* = 8.0 Hz, 1H), 6.93 (d, *J* = 8.0 Hz, 1H), 4.37–4.47 (m, 1H), 3.38 (dd, *J* = 16.4, 4.0 Hz, 1H), 2.94–2.99 (m, 2H), 2.76–2.86 (m, 4H), 2.67 (dd, *J* = 16.4, 8.8 Hz, 1H), 2.56 (br, 4H), 2.34 (s, 3H), 2.28 (br, 1H), 2.27 (s, 3H), 2.17 (s, 3H), 1.85–1.95 (m, 1H). ^13^C NMR (CDCl_3_) δ 163.7, 149.9, 149.1, 148.3, 139.1, 138.0, 137.7, 136.0, 135.3, 134.6, 132.1, 131.2, 130.8, 130.2, 128.1, 126.6, 122.1, 117.4, 55.8, 52.3, 46.3, 45.5, 32.1, 29.3, 26.2, 20.5, 19.6. HRMS calcd. for C_29_H_33_ClN_4_O: 488.2343, found 488.2358.

#### 4.1.8 5-[3,5-Bis(trifluoromethyl)phenyl]-N-[(2S)-5-methyl-8-(4-methyl-1-piperazinyl)-1,2,3,4-tetrahydro-2-naphthalenyl]-2-pyridinecarboxamide (**27**)

The title compound was obtained from (*S*)-5-methyl-8-(4-methylpiperazin-1-yl)-1,2,3,4-tetrahydronaphthalen-2-amine **80** and 5-(3,5-bis(trifluoromethyl)phenyl)picolinic acid using the general procedure A to give **27** (52%) as a white foam. HPLC 97.9%. ^1^H NMR (CDCl_3_) δ 8.79, (dd, *J* = 2.2, 0.6 Hz, 1H), 8.37 (dd, *J* = 8.0, 0.6 Hz, 1H), 8.04 (br s, 2H), 7.97 (br s, 1H), 7.05 (d, J = 8.0 Hz, 1H), 6.93 (d, *J* = 8.0 Hz. 1H), 4.42 (m, 1H), 3.38 (m, 1H), 3.00–2.50 (m, 10H), 2.35 (s, 3H), 2.28 (m, 2H), 2.22 (s, 3H), 1.90 (m, 1H). ^13^C NMR (CDCl_3_) δ 163.3, 150.5, 149.8, 146.7, 139.5, 136.3, 136.2, 135.2, 133.5, 133.2, 132.8, 132.5, 132.2, 130.0, 128.2, 128.6, 127.3, 124.6, 122.8, 122.6, 122.6, 121.9, 119.2, 117.5, 55.7, 52.0, 46.0, 45.6, 32.0, 29.2, 26.1, 19.6. HRMS calcd. for C_30_H_31_F_6_N_4_O 577.2399, found 577.2399.

#### 4.1.9 6-(4-Chloro-2-methylphenyl)-N-[(2S)-5-methyl-8-(4-methyl-1-piperazinyl)-1,2,3,4-tetrahydro-2-naphthalenyl]nicotinamide (**28**)

The title compound was obtained from (*S*)-5-methyl-8-(4-methylpiperazin-1-yl)-1,2,3,4-tetrahydronaphthalen-2-amine **80** and 6-(4-chloro-2-methylphenyl)nicotinic acid using the general procedure A to give **28** (83%) as a white foam. HPLC 97.4%: mp 153–156 °C. ^1^H NMR (CDCl_3_) δ 8.99, (dd, *J* = 2.2, 0.6 Hz, 1H), 8.08 (dd, *J* = 8.0, 0.8 Hz, 1H), 7.47 (dd, *J* = 8.1, 0.7 Hz, 1H), 7.37–7.27 (m. 3H), 7.05 (d, J = 8.0 Hz, 1H), 6.93 (d, *J* = 8.0 Hz. 1H), 6.15 (br d, *J* = 7.9 Hz, 1H), 4.51 (m, 1H), 3.28 (m, 1H), 2.92–2.50 (m, 10H), 2.35 (s, 3H), 2.28 (m, 2H), 2.36 (s, 3H), 2.35 (s, 3H), 2.32 (m, 1H), 2.21 (s, 3H), 1.90 (m, 1H). ^13^C NMR (CDCl_3_) δ 165.3, 161.7, 150.0, 147.6, 138.1, 135.8, 135.1, 134.8, 132.1, 131.1, 131.0, 129.3, 128.8, 128.4, 126.4, 124.0, 117.5, 55.8, 52.2, 46.3, 45.8, 31.8, 28.7, 25.5, 20.5, 19.6. HRMS calcd. for C_29_H_34_ClN_4_O 489.2416, found 489.2408.

#### 4.1.10 5-(4-Chloro-2-methylphenyl)-N-[(2S)-5-methyl-8-(4-methyl-1-piperazinyl)-1,2,3,4-tetrahydro-2-naphthalenyl]nicotinamide (**29**)

The title compound was obtained from (*S*)-5-methyl-8-(4-methylpiperazin-1-yl)-1,2,3,4-tetrahydronaphthalen-2-amine **80** and 5-(4-chloro-2-methylphenyl)nicotinic acid using the general procedure A to give **29** (69%) as a white foam. HPLC 95.3%. ^1^H NMR (CDCl_3_) δ 8.89 (d, *J* = 2.1 Hz, 1H), 8.65 (d, *J* = 2.2 Hz, 1H), 8.05 (t, *J* = 2.2 Hz, 1H), 7.30 (m, 1H), 7.17 (d, *J =* 8.1 Hz, 1H), 7.05 (d, *J* = 8.0 Hz, 1H), 6.93 (d, *J* = 8.0 Hz, 1H), 6.17 (br d, *J* = 7.7 Hz, 1H), 4.49 (m, 1H), 3.28 (m, 1H), 2.94–2.50 (m, 10H), 2.34 (s, 3H), 2.24 (s, 3H), 2.23 (m, 1H), 2.21 (s, 3H), 1.95 (m, 1H). ^13^C NMR (CDCl_3_) δ 165.2, 152.3, 150.0, 146.4, 137.7, 136.7, 135.8, 135.8, 135.0, 134.6, 132.1, 131.3, 130.8, 130.4, 129.3, 128.4, 126.6, 117.5, 55.9, 52.3, 46.4, 46.0, 31.7, 28.7, 25.5, 20.5, 19.6. HRMS calcd. for C_29_H_34_ClN_4_O 489.2416, found 489.2413.

#### 4.1.11 (S)-2-(4-Chloro-2-methylphenyl)-N-(5-methyl-8-(4-methylpiperazin-1-yl)-1,2,3,4-tetrahydronaphthalen-2-yl)pyrimidine-5-carboxamide (**30**)

**Figure:**
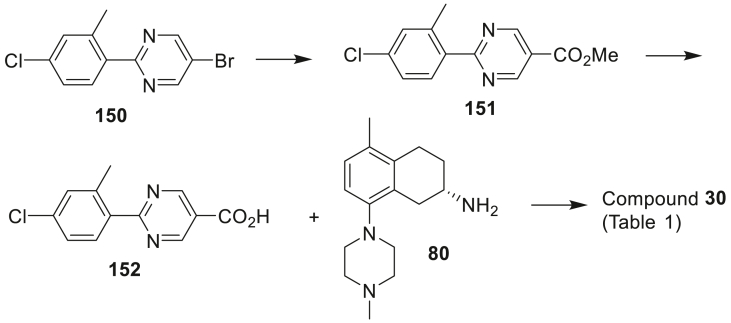

A mixture of 5-bromo-2-iodopyrimidine (1.50 g, 5.27 mmol), (4-chloro-2-methylphenyl)boronic acid (0.980 g, 5.75 mmol) and Cs_2_CO_3_ (3.42 g, 10.4 mmol) in toluene (120 mL) and water (15 mL) was purged with nitrogen. Pd(PPh_3_)_4_ (0.060 g, 0.052 mmol) was added and the mixture was refluxed under nitrogen for 3 h. Workup and chromatography on silica gave 5-bromo-2-(4-chloro-2-methylphenyl)pyrimidine **150** (0.716 g, 44%) as a white solid. mp 104–105 °C. ^1^H NMR (CDCl_3_) δ 8.87 (s, 2H), 7.83 (d, *J* = 8.2 Hz, 1H), 7.27–7.32 (m, 2H), 2.55 (s, 3H). LRMS Found: [M+H] = 283.0, 285.0, 287.0.

Triethylamine (0.60 mL, 4.3 mmol) was added to a solution of **150** (0.605 g, 2.13 mmol) in DMSO (20 mL) and MeOH (20 mL) in a Berghof pressure reactor, followed by the addition of Pd(OAc)_2_ (0.048 g, 0.21 mmol) and DPPP (0.088 g, 0.21 mmol). The reactor was evacuated and then flushed twice with carbon monoxide, then pressurised with carbon monoxide to 80 psi and heated to 80 °C for 18 h. The mixture was partitioned between EtOAc and water, the organic extracts were dried and evaporated. Chromatography on silica using 4:1 hexanes:EtOAc gave methyl 2-(4-chloro-2-methylphenyl)pyrimidine-5-carboxylate **151** (0.520 g, 93%) as a white crystalline solid. mp. 108–109 °C. ^1^H NMR (CDCl_3_) δ 9.34 (s, 2H), 7.95 (dd, *J* = 7.7, 1.2 Hz, 1H), 7.29–7.32 (m, 2H), 4.01 (s, 3H), 2.60 (s, 3H). LRMS Found: [M+H] = 263.2, 265.1.

LiOH (0.120 g, 5.01 mmol) in water (10 mL) was added to a solution of **151** (0.441 g, 1.68 mmol) in THF (20 mL) and MeOH (20 mL), then stirred at room temperature for 18 h. The solvent was evaporated and the residue was diluted with water (80 mL), 2 M HCl was added until pH 2, the resulting white precipitate was filtered and dried to give 2-(4-chloro-2-methylphenyl)pyrimidine-5-carboxylic acid **152** (0.230 g, 55%) as a white solid. mp > 230 °C. ^1^H NMR ((CD_3_)_2_SO) δ 13.84 (bs, 1H), 9.31 (s, 2H), 7.93 (d, *J* = 8.4 Hz, 1H), 7.48 (d, *J* = 2.0 Hz, 1H), 7.43 (dd, *J* = 8.4, 1.9 Hz, 1H), 2.55 (s, 3H). LRMS Found: [M+H] = 249.1, 251.1.

The title compound was obtained from (*S*)-5-methyl-8-(4-methylpiperazin-1-yl)-1,2,3,4-tetrahydronaphthalen-2-amine **80** and 2-(4-chloro-2-methylphenyl)pyrimidine-5-carboxylic acid **152** using the general procedure A to give **30** (54%) as a white foam. HPLC 97.8%. ^1^H NMR (CDCl_3_) δ 9.15 (s, 2H), 7.89 (dd, *J* = 7.7, 1.0 Hz, 1H), 7.28–7.32 (m, 2H), 7.06 (d, *J* = 8.0 Hz, 1H), 6.94 (d, *J* = 8.0 Hz, 1H), 6.14 (d, *J* = 7.7 Hz, 1H), 4.48–4.57 (m, 1H), 3.27 (dd, *J* = 16.5, 4.6 Hz, 1H), 2.89 (t, *J* = 4.7 Hz, 4H), 2.78–2.83 (m, 4H), 2.58 (s, 3H), 2.49–2.65 (br, 3H), 2.35 (s, 3H), 2.22 (s, 3H), 2.18–2.26 (br, 1H), 1.95–2.05 (m, 1H). ^13^C NMR (CDCl_3_) δ 168.5, 163.5, 155.8, 150.0, 140.3, 136.3, 135.6, 135.0, 132.5, 132.1, 131.7, 129.1, 128.5, 126.4, 125.2, 117.6, 55.8, 52.3, 46.3, 46.0, 31.7, 28.6, 25.4, 21.7, 19.6. HRMS calcd. for C_28_H_32_ClN_5_O: 489.2295, found 489.2311.

#### 4.1.12 (S)-5-(4-Chloro-2-methylphenyl)-N-(5-methyl-8-(4-methylpiperazin-1-yl)-1,2,3,4-tetrahydronaphthalen-2-yl)pyrazine-2-carboxamide (**31**)

**Figure:**
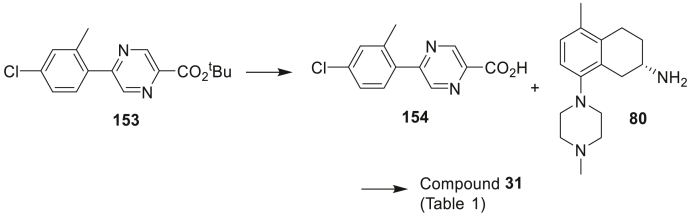

A mixture of (4-chloro-2-methylphenyl)boronic acid (3.57 g, 21.0 mmol) and *tert*-butyl 5-chloropyrazine-2-carboxylate (3.743 g, 17.4 mmol) in toluene (150 mL), MeOH (60 mL) and aqueous sodium carbonate (2 M, 30 mL, 60 mmol) was purged with nitrogen. Pd(dppf)Cl_2_.DCM (0.71 g, 0.87 mmol) was added and the mixture was refluxed under nitrogen for 45 min. The mixture was partitioned between EtOAc and water and the organic fractions were dried and evaporated. Chromatography on silica using 9:1 hexanes:EtOAc gave *tert*-butyl 5-(4-chloro-2-methylphenyl)pyrazine-2-carboxylate **153** (5.22 g, 98%) as a colourless oil. ^1^H NMR (CDCl_3_) δ 9.27 (d, *J* = 1.5 Hz, 1H), 8.78 (*J* = 1.5 Hz, 1H), 7.39 (d, *J* = 8.1 Hz, 1H), 7.28–7.33 (m, 2H), 2.39 (s, 3H), 1.66 (s, 9H). LRMS Found: [M + H–C_4_H_8_] = 249.1, 251.1.

A solution of **153** (0.523 g, 1.72 mmol) and trifluoroacetic acid (2.55 mL, 34.3 mmol) in DCM (20 mL) was stirred at room temperature for 2 h, and then at reflux for 1 h. Evaporation of the solvent gave a yellow solid, which was triturated with water and then dried to give 5-(4-chloro-2-methylphenyl)pyrazine-2-carboxylic acid **154** (0.415 g, 97%) as a white solid. mp. 211–213 °C. ^1^H NMR ((CD_3_)_2_SO) δ 13.79 (bs, 1H), 9.25 (d, *J* = 1.1 Hz, 1H), 8.98 (s, 1H), 7.59 (d, *J* = 8.3 Hz, 1H), 7.51 (d, *J* = 1.9 Hz, 1H), 7.45 (dd, *J* = 8.2, 1.9 Hz, 1H), 2.39 (s, 3H). LRMS Found: [M+H] = 249.1, 251.1.

The title compound was obtained from (*S*)-5-methyl-8-(4-methylpiperazin-1-yl)-1,2,3,4-tetrahydronaphthalen-2-amine **80** and 5-(4-chloro-2-methylphenyl)pyrazine-2-carboxylic acid **154** using the general procedure A to give **31** (40%) as a white foam. HPLC 95.5%. ^1^H NMR (CDCl_3_) δ ^1^H NMR (CDCl_3_) δ 9.48 (d, *J* = 1.5 Hz, 1H), 8.61 (d, *J* = 1.4 Hz, 1H), 7.85 (d, *J* = 8.4 Hz, 1H), 7.41 (d, *J* = 8.2 Hz, 1H), 7.31–7.35 (m, 2H), 7.05 (d, *J* = 8.0 Hz, 1H), 6.93 (d, *J* = 8.0 Hz, 1H), 4.46 (m, 1H), 3.35 (dd, J = 1 6.5, 4.4 Hz, 1H), 2.92–2.97 (m, 2H), 2.80–2.87 (m, 4H), 2.70 (dd, *J* = 16.6 Hz, 8.9 Hz, 1H), 2.56 (bs, 3H), 2.41 (s, 3H), 2.34 (s, 3H), 2.25–2.29 (m, 1H), 2.22 (s, 3H), 1.93 (m, 1H). ^13^C NMR (CDCl_3_) δ 162.7, 157.0, 150.0, 143.6, 142.5, 138.7, 135.9, 135.1, 134.8, 132.1, 131.5, 131.4, 129.8, 128.2, 126.8, 117.5, 55.9, 52.3, 46.3, 45.5, 31.9, 29.1, 26.0, 20.6, 19.6. HRMS calcd. for C_28_H_32_ClN_5_O: 489.2295, found 489.2300.

#### 4.1.13 (S)-5-(4-Chloro-2-methylphenyl)-N-(5-methyl-8-(4-methylpiperazin-1-yl)-1,2,3,4-tetrahydronaphthalen-2-yl)thiophene-2-carboxamide (**32**)

**Figure:**
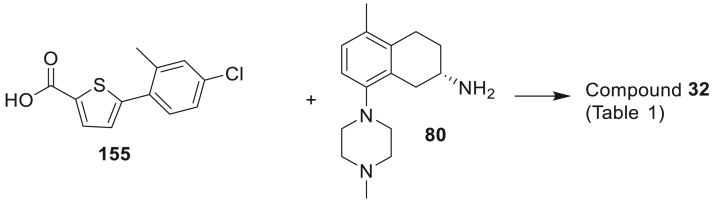

A mixture of 5-bromothiophene-2-carboxylic acid (2.29 g, 11.1 mmol), (4-chloro-2-methylphenyl)boronic acid (1.98 g, 11.6 mmol) and Cs_2_CO_3_ (7.21 g, 22.1 mmol) in DMF/toluene (1:2, 50 mL) was purged with nitrogen. Pd(PPh_3_)_4_ (0.26 g, 0.23 mmol) was added and the mixture was heated to 80 °C under nitrogen for 18 h. The mixture was partitioned between EtOAc and water, the aqueous layer was acidified to pH 2 with 2 M HCl, then extracted with EtOAc. The organic fractions were dried and then evaporated on to silica gel, chromatography on silica using EtOAc gave 5-(4-chloro-2-methylphenyl)thiophene-2-carboxylic acid **155** (1.777 g, 63%) as an off white solid. mp. 206–208 °C. ^1^H NMR ((CD_3_)_2_SO) δ 13.18 (bs, 1H), 7.73 (d, *J* = 3.9 Hz, 1H), 7.44–7.49 (m, 2H), 7.35 (dd, *J* = 8.3, 1.9 Hz, 1H), 7.29 (d, *J* = 3.8 Hz, 1H), 2.40 (s, 3H). LRMS Found: [M − H] = 251.1, 253.1.

The title compound was obtained from (*S*)-5-methyl-8-(4-methylpiperazin-1-yl)-1,2,3,4-tetrahydronaphthalen-2-amine **80** and 5-(4-chloro-2-methylphenyl)pyrazine-2-carboxylic acid **155** using the general procedure A to give **32** (77%) as a white foam. HPLC 97.4%. ^1^H NMR (CDCl_3_) δ 7.44 (d, *J* = 3.8 Hz, 1H), 7.31 (d, *J* = 8.2 Hz, 1H), 7.28 (d, *J* = 2.0 Hz, 1H), 7.21 (dd, *J* = 8.2, 2.2 Hz, 1H), 7.04 (d, *J* = 8.1 Hz, 1H), 6.99 (d, *J* = 3.8 Hz, 1H), 6.93 (d, *J* = 8.0 Hz, 1H), 5.96 (d, *J* = 7.9 Hz, 1H), 4.40–4.46 (m, 1H), 3.29 (dd, *J* = 16.4, 4.5 Hz, 1H), 2.89 (m, 4H), 2.80 (t, *J* = 6.5 Hz, 2H), 2.68 (dd, *J* = 16.4, 8.3 Hz, 1H), 2.58 (b, 4H), 2.40 (s, 3H), 2.36 (s, 3H), 2.19 (m, 1H), 2.18 (s, 3H), 1.91 (m, 1H). ^13^C NMR (CDCl_3_) δ 161.5, 150.0, 146.6, 139.1, 138.2, 135.2, 134.4, 132.1, 132.0, 131.7, 131.0, 129.6, 128.3, 128.1, 127.3, 126.4, 117.4, 55.9, 52.3, 46.4, 45.8, 31.9, 29.0, 25.7, 21.2, 19.6. HRMS calcd. for C_28_H_32_ClN_3_OS: 493.1955, found 493.1968.

#### *4.1.14* *General procedure B: (S)-1-(5-(4-Chloro-2-methylphenyl)pyridin-*2-yl*)-3-(5-methyl-8-(4-methylpiperazin-*1-yl*)-1,2,3,4-tetrahydronaphthalen-*2-yl*)urea (****36****)*

**Figure:**
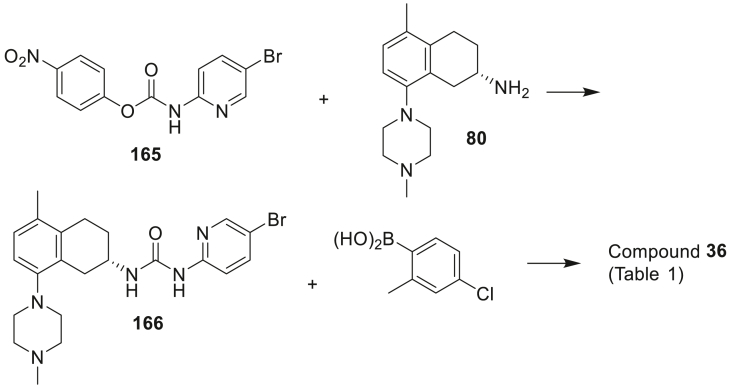

To a suspension of 5-bromopyridin-2-amine (1.00 g, 5.78 mmol) and pyridine (0.56 mL, 6.94 mmol) in DCM (10 mL) in an ice bath was added 4-nitrophenyl carbonochloridate (1.40 g, 6.94 mmol) portionwise. The mixture was stirred at room temperature overnight. The resulting precipitate was collected by filtration, washed with DCM, and dried under vacuum to give the product **165** as a white solid (1.97 g, 100%) which was used crude for the next step.

To a solution of **80** (0.260 g, 1.00 mmol) in MeCN (10 mL) and DCM (10 mL) at room temperature was added **165** (0.405 g, 1.20 mmol), followed by trimethylamine (0.70 mL, 5.00 mmol). The mixture was stirred overnight and distributed between water and ethyl acetate. The organic phase was washed with water and brine, dried over anhydrous Na_2_SO_4_. The solvent was removed to give the crude product, which was purified by Davisil® column chromatography, using gradient mixtures of MeOH and DCM (v/v = 8–15%) as eluent to give the product **166** as a white solid (0.388 g, 85%): mp 185–187 °C. ^1^H NMR (CDCl_3_, 400 MHz) δ 9.04 (br d, *J* = 6.5 Hz, 1H), 8.23 (br, 1H), 8.07 (d, *J* = 2.3 Hz, 1H), 7.64 (dd, *J* = 8.8, 2.4 Hz, 1H), 7.03 (d, *J* = 8.0 Hz, 1H), 6.90 (d, *J* = 8.0 Hz, 1H), 6.73 (d, *J* = 8.8 Hz, 1H), 4.20–4.30 (m, 1H), 3.25 (dd, *J* = 16.3, 4.2 Hz, 1H), 2.92–2.98 (m, 2H), 2.78–2.88 (m, 4H), 2.66–2.72 (m, 1H), 2.56 (br, 4H), 2.34 (s, 3H), 2.21 (s, 3H), 1.85–1.95 (m, 1H). HRMS calcd. for C_22_H_28_BrN_5_O (M + H^+^) *m/z* 458.15500, found 458.15472.

A mixture of (*S*)-1-(5-bromopyridin-2-yl)-3-(5-methyl-8-(4-methylpiperazin-1-yl)-1,2,3,4-tetrahydronaphthalen-2-yl)urea **166** (60 mg, 0.13 mmol), (4-chloro-2-methylphenyl)boronic acid (67 mg, 0.39 mmol) and aqueous sodium carbonate (2 M, 0.39 mL, 0.78 mmol) in toluene (2 mL) and EtOH (1 mL) was purged with nitrogen gas before Pd(dppf)Cl_2_-DCM (5 mg, 0.0065 mmol) was added. The resulting mixture was heated in an oil bath at 85 °C overnight. After the solvent was removed, the residue was taken in EtOAc and washed with water and brine, dried over anhydrous sodium sulphate and filtered through a pad of Celite. The solvent was removed to give the crude product, which was purified by column chromatography on silica, using mixtures of MeOH and DCM (v/v = 5–10%) as eluent, followed by recrystallisation from DCM and heptane to give **36** as a white solid (47 mg, 71%): HPLC 98.0%. mp 112–115 °C. ^1^H NMR (CDCl_3_) δ 9.51 (br d, *J* = 4.3 Hz, 1H), 9.20 (br, 1H), 7.95 (d, *J* = 2.1 Hz, 1H), 7.51 (dd, *J* = 8.5, 2.4 Hz, 1H), 7.28 (d, *J* = 2.1 Hz, 1H), 7.23 (dd, *J* = 8.2, 2.1 Hz, 1H), 7.10 (d, *J* = 8.2 Hz, 1H), 7.01 (d, *J* = 8.0 Hz, 1H), 6.96 (d, *J* = 8.4 Hz, 1H), 6.90 (d, *J* = 8.8 Hz, 1H), 4.26–4.34 (m, 1H), 3.30 (dd, *J* = 16.3, 4.2 Hz, 1H), 2.92–2.98 (m, 2H), 2.78–2.88 (m, 4H), 2.68–2.74 (m, 2H), 2.57 (br, 4H), 2.34 (s, 3H), 2.24 (s, 3H), 2.20 (s, 3H), 1.89–1.99 (m, 1H). HRMS calcd. for C_29_H_35_ClN_5_O 504.25246, found 504.25242.

#### 4.1.15 (S)-4-(4-Fluorophenyl)-N-(5-methyl-8-(4-methylpiperazin-1-yl)-1,2,3,4-tetrahydronaphthalen-2-yl)piperazine-1-carboxamide (**43**)

The title compound was obtained from (*S*)-5-methyl-8-(4-methylpiperazin-1-yl)-1,2,3,4-tetrahydronaphthalen-2-amine **80** and 1-(4-fluorophenyl)piperazine using the general procedure C to give **43** (74%) as a white solid. HPLC 95.5%. mp 189–190 °C. ^1^H NMR (CDCl_3_) δ 6.95–7.03 (m, 3H), 6.86–6.92 (m, 3H), 4.46 (d, *J* = 7.4 Hz, 1H), 4.12–4.20 (m, 1H), 3.47–3.57 (m, 4H), 3.21 (dd, *J* = 16.3, 4.5 Hz, 1H), 3.10 (apparent t, *J* = 5.1 Hz, 4H), 2.83–2.93 (m, 4H), 2.74 (t, *J* = 6.6 Hz, 2H), 2.52–2.59 (m, 5H), 2.35 (s, 3H), 2.20 (s, 3H), 2.13–2.19 (m, 1H), 1.74–1.79 (m, 1H). ^13^C NMR (CDCl_3_) δ 158.9, 157.3, 156.5, 150.0, 148.0, 148.0, 135.5, 132.0, 130.2, 128.1, 118.6, 118.6, 117.3, 116.0, 115.8, 55.9, 52.3, 50.4, 46.4, 46.4, 44.1, 32.5, 29.6, 25.9, 19.6. HRMS calcd. for C_27_H_37_FN_5_O 466.29818, found 466.29871.

#### 4.1.16 (S)-5-(4-Chloro-2-methylphenyl)-N-(8-(4-methylpiperazin-1-yl)-1,2,3,4-tetrahydronaphthalen-2-yl)picolinamide (**53**)

The title compound was obtained from (*S*)-8-(4-methylpiperazin-1-yl)-1,2,3,4-tetrahydronaphthalen-2-amine **82** and **83** using the general procedure A to give **53** (59%) as a white solid. HPLC 99.9%. mp 72–75 °C. ^1^H NMR (CDCl_3_) δ 8.49 (dd, *J* = 2.2, 0.7 Hz, 1H), 8.30 (dd, *J* = 8.0, 0.6 Hz, 1H), 8.10 (d, *J* = 8.4 Hz, 1H), 7.79 (dd, *J* = 8.0, 2.2 Hz, 1H), 7.34–7.6 (m, 2H), 7.18–7.12 (m, 2H), 6.97 (d, *J* = 7.7 Hz, 1H), 6.91 (d, *J* = 7.5 Hz, 1H), 4.49–4.40 (m, 1H), 3.34 (dd, *J* = 16.5, 4.8 Hz, 1H), 3.05–2.94 (m, 4H), 2.92–2.84 (m, 2H), 2.70–2.51 (m, 5H), 2.36 (s, 3H), 2.26 (s, 3H), 2.25–2.18 (m, 1H), 1.94–1.83 (m, 1H). ^13^C NMR (CDCl_3_) δ 163.7, 152.0, 149.1, 148.3, 139.2, 138.0, 137.7, 137.0, 136.1, 134.6, 131.2, 130.8, 130.1, 126.7, 126.6, 124.6, 122.1, 117.5, 55.8, 52.2, 46.3, 45.9, 31.8, 29.3, 28.3, 20.5. HRMS calcd. for C_28_H_31_ClN_4_O: 475.2259, found 475.2259.

#### 4.1.17 (S)-5-(4-Chloro-2-methylphenyl)-N-(8-(4-methylpiperazin-1-yl)-5-phenyl-1,2,3,4-tetrahydronaphthalen-2-yl)picolinamide (**54**)

**Figure:**
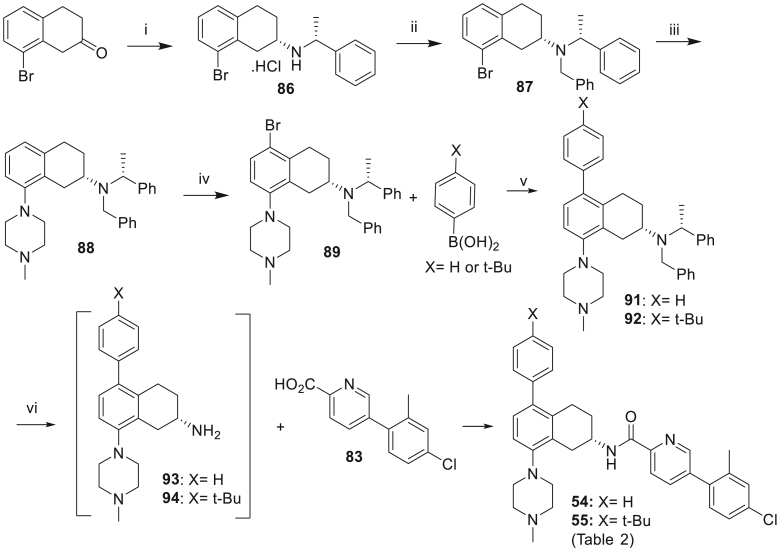

*Step i.* 8-Bromo-3,4-dihydronaphthalen-2(1*H*)-one (18.39 g, 81.7 mmol) in toluene (50 mL) was added pTSA (0.155 g, 0.817 mmol) and (*R*)-*N-*ethylphenylamine (11.59 mL, 89.9 mmol). The reaction mixture was heated to 50 °C for 2 h. The reaction mixture was cooled to 0 °C, sodium borohydride (4.95 g, 130.7 mmol) in methanol:isopropanol (2:3) was added in portions. The reaction mixture was heated at 70 °C for 18 h. The reaction mixture was quenched with water (50 mL), extracted with ethyl acetate (3 × 20 mL) and evaporated. The crude product was dissolved in ethyl acetate (20 mL), added anhydrous HCl (24.5 mL, 4 M in dioxane) dropwise. The mixture was sonicated until white precipitate forms. The precipitate was filtered, collected into a flask. The white precipitate was added ethyl acetate:ethanol (2:1, 100 mL) and heated at 50 °C for 3 h. The mixture was cooled to 5 °C for 30 min and filtered to give (*S*)-8-bromo*-N*-((*R*)-1-phenylethyl)-1,2,3,4-tetrahydronaphthalen-2-amine **86** as white solids (11.0 g, 37%). αD = −39.3°. ^1^H NMR (CDCl_3_) δ 10.43–10.23 (m, 2H), 7.78 (d, *J* = 7.2 Hz, 2H), 7.44 (t, *J* = 7.2 Hz, 2H), 7.39–7.34 (m, 1H), 7.29–7.27 (m, 1H), 6.94–6.89 (m, 2H), 4.60–4.50 (m, 1H), 3.52 (d, *J* = 13.0 Hz, 1H), 3.19–3.02 (m, 2H), 2.92 (dd, *J* = 15.1, 2.9 Hz, 1H), 2.63 (dt, *J* = 12.6, 5.4 Hz, 1H), 2.45 (dd, *J* = 12.1, 2.5 Hz, 1H), 2.24–2.12 (m, 1H), 2.08 (d, *J* = 6.8 Hz, 3H). LRMS Found: [M+H] = 330.2.
*Step ii.* (*S*)-8-Bromo-*N*-((*R*)-1-phenylethyl)-1,2,3,4-tetrahydronaphthalen-2-amine **86** (2.00 g, 5.45 mmol) in acetonitrile (30 mL) was added potassium iodide (0.045 g, 0.273 mmol), K_2_CO_3_ (1.88 g, 13.6 mmol) followed by benzyl bromide (0.78 mL, 6.54 mmol). The reaction mixture was refluxed at 150 °C in a sealed tube for 27 h. The reaction mixture was diluted with EtOAc and washed with water. The organic layer was dried over anhydrous Na_2_SO_4_, filtered and evaporated. The residue purified by silica column chromatography using hexanes:EtOAc (v/v = 2%) to give (*S*)–*N*-benzyl-8-bromo-*N*-((*R*)-1-phenylethyl)-1,2,3,4-tetrahydronaphthalen-2-amine **87** as a white foam (1.87 g, 82%). ^1^H NMR (CDCl_3_) δ 7.48 (d, *J* = 7.1 Hz, 2H), 7.39 (d, *J* = 7.1 Hz, 2H), 7.36–7.28 (m, 5H), 7.24–7.19 (m, 2H), 6.97–6.89 (m, 2H), 4.02 (q, *J* = 6.9 Hz, 1H), 3.92 (d, *J* = 15.1 Hz, 1H), 3.75 (d, *J* = 15.2 Hz, 1H), 3.18–3.09 (m, 1H), 3.02 (dd, *J* = 17.0, 5.3 Hz, 1H), 2.76–2.58 (m, 3H), 1.66–1.60 (m, 1H), 1.51–1.45 (m, 1H), 1.39 (d, *J* = 6.9 Hz, 3H). LRMS Found: [M+H] = 420.2.
*Step iii.* (*S*)–*N*-benzyl-8-bromo-*N*-((*R*)-1-phenylethyl)-1,2,3,4-tetrahydronaphthalen-2-amine **87** (1.87 g, 4.45 mmol) was dissolved in toluene (30 mL) and flushed with nitrogen for 5 min. Palladium acetate (0.04 g, 0.178 mmol), BINAP (0.22 g, 0.356 mmol) and N-methyl piperazine (0.662 g, 6.68 mmol) was added to the reaction mixture. The reaction was heated to 80 °C for 30 min. Sodium tert-butoxide (0.599 g, 6.23 mmol) was added and heated to 100 °C for a further 3 h. The reaction mixture was diluted with EtOAc and washed with water. The organic layer was dried over anhydrous Na_2_SO_4_, filtered and evaporated. The residue purified by silica column chromatography using EtOAc to give (*S*)–*N*-benzyl-8-(4-methylpiperazin-1-yl)-*N*-((*R*)-1-phenylethyl)-1,2,3,4-tetrahydronaphthalen-2-amine **88** as a white solid (1.25 g, 64%). ^1^H NMR (CDCl_3_) δ 7.48 (d, *J* = 7.1 Hz, 2H), 7.40 (d, *J* = 7.1 Hz, 2H), 7.34–7.28 (m, 4H), 7.24–7.18 (m, 2H), 7.05 (t, *J* = 7.7 Hz, 1H), 6.85 (d, *J* = 7.7 Hz, 1H), 6.76 (d, *J* = 7.4 Hz, 1H), 4.02 (q, *J* = 6.8 Hz, 1H), 3.92 (d, *J* = 15.4 Hz, 1H), 3.79 (d, *J* = 15.4 Hz, 1H), 3.10–3.02 (m, 2H), 2.99–2.92 (m, 2H), 2.81–2.73 (m, 3H), 2.67–2.45 (m, 6H), 2.39 (s, 3H), 1.76–1.68 (m, 1H), 1.58–1.51 (m, 1H), 1.40 (d, *J* = 6.8 Hz, 3H). LRMS Found: [M+H] = 440.3.
*Step iv.* (*S*)–*N*-Benzyl-8-(4-methylpiperazin-1-yl)-*N*-((*R*)-1-phenylethyl)-1,2,3,4-tetrahydronaphthalen-2-amine **88** (3.11 g, 7.07 mmol) in DMF (20 mL) was added N-bromosuccinamide (1.64 g, 9.20 mmol). The reaction was stirred at room temperature for 72 h. The reaction mixture was diluted with EtOAc and washed with water. The organic layer was dried over anhydrous Na_2_SO_4_, filtered and evaporated. The residue purified by silica column chromatography using EtOAc:MeOH (v/v = 5%) to give (*S*)–N-benzyl-5-bromo-8-(4-methylpiperazin-1-yl)-N-((R)-1-phenylethyl)-1,2,3,4-tetrahydronaphthalen-2-amine **89** as white solids (2.89 g, 79%). ^1^H NMR (CDCl_3_) δ 7.47 (d, *J* = 7.1 Hz, 2H), 7.39 (d, *J* = 7.1 Hz, 2H), 7.34–7.28 (m, 5H), 7.24–7.19 (m, 2H), 6.73 (d, *J* = 8.5 Hz, 1H), 4.03 (q, *J* = 6.7 Hz, 1H), 3.92 (d, *J* = 15.4 Hz, 1H), 3.79 (d, *J* = 15.4 Hz, 1H), 3.10–3.01 (m, 2H), 2.99–2.92 (m, 2H), 2.76–2.68 (m, 3H), 2.58–2.42 (m, 6H), 2.39 (s, 3H), 1.81–1.76 (m, 1H), 1.58–1.52 (m, 1H), 1.41 (d, *J* = 6.8 Hz, 3H). LRMS Found: [M+H] = 518.2.
*Step v.***89** (0.283 g, 0.546 mmol) was dissolved in toluene:EtOH (10:4 mL) and flushed with nitrogen for 5 min. Phenylboronic acid (0.073 g, 0.60 mmol) and K_2_CO_3_ (1.09 mL, 2 N solution, 2.18 mmol) was added to the reaction mixture. The reaction mixture was bubbled nitrogen for 5 min, followed by addition of PddppfCl_2_.DCM (0.045 g, 0.055 mmol). The reaction was heated in a sealed tube at 80 °C for 3 h. The solvent was removed, and purified by silica column chromatography using EtOAc:MeOH (v/v = 15%) to give **91** (0.084 g, 30%). ^1^H NMR (CDCl_3_) δ 7.46 (d, *J* = 7.2 Hz, 2H), 7.42–7.36 (m, 2H), 7.36–7.26 (m, 7H), 7.23–7.16 (m, 4H), 7.00 (d, *J* = 8.0 Hz, 1H), 6.93 (d, *J* = 8.1 Hz, 1H), 4.03 (q, *J* = 6.8 Hz, 1H), 3.90 (d, *J* = 15.4 Hz, 1H), 3.75 (d, *J* = 15.4 Hz, 1H), 3.18–3.03 (m, 2H), 3.02–2.92 (m, 2H), 2.91–2.78 (m, 2H), 2.71–2.43 (m, 7H), 2.39 (s, 3H), 1.72–1.65 (m, 1H), 1.49–1.42 (m, 1H), 1.41 (d, *J* = 6.8 Hz, 3H). LRMS Found: [M+H] = 516.3.
*Step vi.***91** (0.185 g, 0.36 mmol) was dissolved in MeOH (20 mL) and added AcOH (1 mL). The reaction was hydrogenated over 10% Pd–C (0.20 g) at 60 psi for 48 h. The catalyst was filtered off and the filtrate concentrated to dryness to give pure **93** as a colorless, viscous oil which was used directly for the next step. 5-(4-Chloro-2-methylphenyl)picolinic acid **83** (0.100 g, 0.43 mmol) in DMF (5 mL) was purged with nitrogen before DIPEA (0.093 g, 0.72 mmol) was added to the reaction mixture. HATU (0.177 g, 0.47 mmol) was added and stirred for 15 min. (*S*)-8-(4-methylpiperazin-1-yl)-5-phenyl-1,2,3,4-tetrahydronaphthalen-2-amine **93** (0.115 g, 0.36 mmol) was added to the reaction mixture and stirred at r.t. for 1 h. The reaction mixture was diluted with EtOAc acetate and washed with water and 2 M NaOH solution. The organic layer was dried over anhydrous Na_2_SO_4_ and filtered through a pad of Celite. The solvent was removed to give the crude product, which was purified by silica column chromatography using EtOAc:MeOH (v/v = 20%) as eluent to give **54** as a white foam (0.081 g, 41%). mp 95–98 °C. HPLC 97.4%. ^1^H NMR (CDCl_3_) δ 8.49 (dd, *J* = 2.1, 0.7 Hz, 1H), 8.30 (dd, *J* = 8.0, 0.7 Hz, 1H), 8.10 (d, *J* = 8.5 Hz, 1H), 7.79 (dd, *J* = 8.0, 2.2 Hz, 1H), 7.43–7.37 (m, 2H), 7.36–7.27 (m, 5H), 7.15 (d, *J* = 8.2 Hz, 1H), 7.10 (d, *J* = 8.1 Hz, 1H), 7.06 (d, *J* = 8.1 Hz, 1H), 4.49–4.40 (m, 1H), 3.44 (dd, *J* = 16.4, 4.7 Hz, 1H), 3.04–2.97 (m, 2H), 2.96–2.89 (m, 2H), 2.86–2.78 (m, 2H), 2.76–2.53 (m, 5H), 2.37 (s, 3H), 2.26 (s, 3H), 2.18–2.10 (m, 1H), 1.81–1.72 (m, 1H). ^13^C NMR (CDCl_3_) δ 163.7, 151.0, 149.0, 148.3, 142.1, 139.2, 138.0, 137.9, 137.7, 136.0, 134.7, 134.6, 131.2, 130.8, 130.5, 129.5, 128.3, 128.3, 126.9, 126.6, 122.1, 117.6, 55.6, 52.1, 46.1, 45.6, 32.2, 29.5, 27.5, 20.5. HRMS calcd. for C_34_H_35_ClN_4_O: 550.2499, found 550.2500.

#### 4.1.18 (S)–N-(5-Benzyl-8-(4-methylpiperazin-1-yl)-1,2,3,4-tetrahydronaphthalen-2-yl)-5-(4-chloro-2-methylphenyl)picolinamide (**56**)

**Figure:**
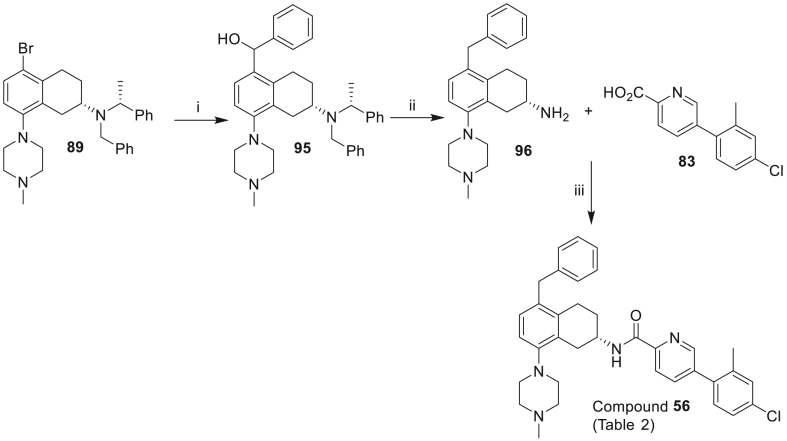

*Step i.***89** (0.868 g, 1.67 mmol) was dissolved in THF (20 mL) and cooled to −78 °C. n-BuLi (1.09 mL, 2 M solution in diethyl ether, 2.18 mmol) was added followed by benzaldehyde (0.532 g, 5.31 mmol). The reaction was stirred at −78 °C for 5 h. The reaction mixture was added water and extracted with EtOAc. The organic layer was dried over anhydrous Na_2_SO_4_, filtered and evaporated. The residue purified by silica column chromatography using EtOAc:MeOH (v/v = 5%) to give **95** (0.096 g, 11%). ^1^H NMR (CDCl_3_) δ 7.45–7.39 (m, 2H), 7.38–7.34 (m, 2H), 7.33–7.25 (m, 8H), 7.24–7.18 (m, 4H), 6.90 (d, *J* = 8.3 Hz, 1H), 5.92 (d, *J* = 10.4 Hz, 1H), 4.03 (q, *J* = 6.8 Hz, 1H), 3.88 (d, *J* = 15.6 Hz, 1H), 3.74 (d, *J* = 15.3 Hz, 1H), 3.12–3.03 (m, 1H), 3.02–2.85 (m, 4H), 2.80–2.70 (m, 3H), 2.62–2.47 (m, 5H), 2.39 (s, 3H), 1.72–1.65 (m, 1H), 1.53–1.49 (m, 1H), 1.40 (d, *J* = 6.9 Hz, 3H). LRMS Found: [M+H] = 546.3.
*Step ii.***95** (0.1 g, 0.183 mmol) was dissolved in DCM (20 mL), added TFA (0.136 mL, 1.83 mmol) followed by triethylsilane (0.059 mL, 0.366 mmol). The reaction was stirred at r.t. for 72 h. The reaction mixture was washed with sat. NaHCO_3_ solution, water and extracted with DCM. The solvent was removed and the crude reaction mixture was dissolved in MeOH (10 mL) and was hydrogenated over 10% Pd–C (0.20 g) at 55 psi for 72 h. The catalyst was filtered off and the filtrate concentrated to dryness to give pure **96** as a colorless, viscous oil which was used directly for the next step. 5-(4-chloro-2-methylphenyl)picolinic acid **83** (0.059 g, 0.25 mmol) in DMF (5 mL) was purged with nitrogen before DIPEA (0.054 g, 0.42 mmol) was added to the reaction mixture. HATU (0.103 g, 0.27 mmol) was added and stirred for 15 min. (*S*)-5-Benzyl-8-(4-methylpiperazin-1-yl)-1,2,3,4-tetrahydronaphthalen-2-amine **96** (0.07 g, 0.21 mmol) was added to the reaction mixture and stirred at r.t. for 1.5 h. The reaction mixture was diluted with EtOAc and washed with water and 2 M NaOH solution. The organic layer was dried over anhydrous Na_2_SO_4_ and filtered through a pad of Celite. The solvent was removed to give the crude product, which was purified by silica column chromatography using EtOAc/:MeOH (v/v = 20%) as eluent to give **56** as a white foamy solid (0.025 g, 21%). HPLC 95.6%. mp 77–80 °C. ^1^H NMR (CDCl_3_) δ 8.49 (dd, *J* = 2.1, 0.6 Hz, 1H), 8.28 (dd, *J* = 8.0, 0.6 Hz, 1H), 8.06 (d, *J* = 8.5 Hz, 1H), 7.79 (dd, *J* = 8.0, 2.2 Hz, 1H), 7.34–7.23 (m, 3H), 7.20–7.10 (m, 4H), 7.00–6.94 (m, 2H), 4.42–4.34 (m, 1H), 3.94 (s, 2H), 3.38 (dd, *J* = 16.4, 3.6 Hz, 1H), 3.02–2.95 (m, 2H), 2.91–2.75 (m, 4H), 2.70–2.52 (m, 5H), 2.35 (s, 3H), 2.26 (s, 3H), 2.23–2.15 (m, 1H), 1.88–1.78 (m, 1H). ^13^C NMR (CDCl_3_) δ 163.7, 150.3, 149.1, 148.3, 140.5, 139.1, 138.0, 137.7, 136.0, 135.3, 134.6, 134.3, 131., 130.8, 130.5, 128.9, 128.8, 128.6, 126.6, 126.1, 122.0, 117.5, 55.7, 52.1, 46.1, 45.4, 39.2, 32.2, 29.3, 25.9, 20.5. HRMS calcd. for C_35_H_37_ClN_4_O: 564.2656, found 564.2652.

#### 4.1.19 5-(4-Chloro-2-methylphenyl)-N-{(2S)-8-[4-(dimethylamino)-1-piperidinyl]-5-methyl-1,2,3,4-tetrahydro-2-naphthalenyl}-2-pyridinecarboxamide (**63**)

**102** was obtained from (*S*)-8-bromo-5-methyl-1,2,3,4-tetrahydronaphthalen-2-amine **101** and 4-(dimethylamino)piperidine using the general procedure D to give **102** (76%) as an oil. ^1^H NMR (CDCl_3_) δ 6.98 (d, *J* = 8.0 Hz, 1H), 6.84 (d, *J* = 8.0 Hz, 1H), 3.22 (ddd, *J* = 16.2, 4.4, 2.0 Hz, 1H), 3.16–3.02 (m, 3H), 2.89–2.74 (m, 2H), 2.71–2.60 (m, 1H), 2.44 (m, 1H), 2.32 (s, 6H), 2.25 (m, 2H), 2.18 (s, 3H), 2.04 (m, 1H), 1.87 (m, 2H), 1.64 (m). LRMS Found: [M+H] = 288.

The title compound was obtained from **102** and 5-(4-chloro-2-methylphenyl)picolinic acid **83** using the general procedure A to give **63** (86%) as a white foam. HPLC 95.0%. ^1^H NMR (CDCl_3_) δ 8.49, (dd, *J* = 2.2, 0.7 Hz, 1H), 8.29 (dd, *J* = 8.7, 0.7 Hz, 1H), 8.08 (br d, *J* = 8.4 Hz, 1H), 7.79 (dd, *J* = 8.7, 2.2 Hz, 1H), 7.33–7.28 (m, 2H), 7.16 (d, *J* = 8.2 Hz, 1H), 7.03 (d, *J* = 8.0 Hz, 1H), 6.89 (d, *J* = 8.0 Hz, 1H), 4.42 (m, 1H), 3.37 (dd, *J* = 16.4, 4.0 Hz, 1H), 3.07 (m, 1H), 2.92–2.50 (m, 5H), 2.31 (s, 6H), 2.26 (s, 3H), 2.21 (s, 3H), 2.23 (m, 1H), 1.95 (m, 1H). ^13^C NMR (CDCl_3_) δ 163.8, 163.7, 150.4, 149.2, 149.1, 149.0, 148.3, 148.3, 139.2, 139.1, 138.0, 138.0, 137.7, 136.1, 136.0, 135.4, 135.3, 134.6, 134.5, 132.5, 131.8, 131.2, 130.8, 130.8, 130.2, 130.1, 128.1, 128.0, 126.6, 126.6, 122.0, 117.3, 117.2, 62.5, 56.1, 55.2, 52.8, 52.3, 52.1, 51.5, 45.6, 45.5, 41.7, 41.5, 32.1, 32.0, 29.4, 29.3, 29.3, 29.2, 29.0, 28.4, 28.1, 27.8, 26.3, 24.2, 20.5, 19.6, 19.6. HRMS calcd. for C_31_H_38_ClN_4_O; 517.2734, found 517.2730.

#### 4.1.20 5-(4-Chloro-2-methylphenyl)-N-[(2S)-5-methyl-8-(4-pyridinyl)-1,2,3,4-tetrahydro-2-naphthalenyl]-2-pyridinecarboxamide (**71**)

**115** was obtained from (*S*)-8-bromo-5-methyl-1,2,3,4-tetrahydronaphthalen-2-amine **101** and pyridin-4-ylboronic acid using the general procedure B to give crude **115** which was used directly for the next step.

The title compound was obtained from **115** and 5-(4-chloro-2-methylphenyl)picolinic acid **83** using the general procedure A to give **71** (88%) as a white foam. HPLC 96%. ^1^H NMR (CDCl_3_) δ 8.61 (d, *J* = 5.9 Hz, 2H), 8.45, (dd, *J* = 2.2, 0.8 Hz, 1H), 8.22 (dd, *J* = 8.0, 0.8 Hz, 1H), 8.01 (br d, *J* = 8.4 Hz, 1H), 7.75 (dd, *J* = 8.6, 2.2 Hz, 1H), 7.32–7.28 (m, 2H), 7.23 (d, *J* = 5.9 Hz, 2H), 7.13 (m, 2H), 6.98 (d, *J* = 6.5 Hz, 1H), 4.38 (m, 1H), 3.02–2.82 (m, 3H), 2.70 (dd, *J* = 16.4, 6.4 Hz, 1H), 2.32 (s and m, 4H), 2.29 (m, 1H), 2.23 (s, 3H), 1.91 (m, 1H). ^13^C NMR (CDCl_3_) δ 163.7, 150.0, 149.8, 148.8, 148.3, 139.2, 138.0, 137.7, 137.7, 137.2, 136.0, 135.0, 134.6, 131.6, 131.2, 130.8, 128.0, 127.0, 126.6, 126.6, 124.7, 122.0, 45.6, 38.8, 35.3, 29.2, 26.4, 20.5, 20.1. HRMS calcd. for C_29_H_26_ClN_3_O 468.1854, found 468.1848.

#### 4.1.21 5-(4-Chloro-2-methylphenyl)-N-[(2S)-5-methyl-8-(3-pyridinyl)-1,2,3,4-tetrahydro-2-naphthalenyl]-2-pyridinecarboxamide (**72**)

**116** was obtained from (*S*)-8-bromo-5-methyl-1,2,3,4-tetrahydronaphthalen-2-amine **101** and pyridin-3-ylboronic acid using the general procedure B to give **116** (65%) as an oil. ^1^H NMR (CDCl_3_) δ 1.95 (m, 2H), 7.62 (m, 1H), 7.33 (m, 1H), 7.11 (d, *J* = 7.6 Hz, 1H), 6.99 (d, *J* = 7.6 Hz, 1H), 3.08 (m, 1H), 2.91 (m, 1H), 2.73 (m, 2H), 2.45 (m, 1H), 2.29 (s, 3H), 2.08 (m, 1H), 1.65 (m, 1H). Found: [M+H] = 239.

The title compound was obtained from **116** and 5-(4-chloro-2-methylphenyl)picolinic acid **83** using the general procedure A to give **72** (62%) as a white foam. HPLC 91%.^1^H NMR (CDCl_3_+ drop of ((CD_3_)_2_SO)) δ 8.55 (m, 2H), 8.49, (br s, 1H), 8.27 (m, 2H), 7.78 (m, 1H), 7.65 (m, 1H), 7.39–7.30 (m, 2H), 7.27 (d, *J* = 7.6 Hz, 1H),7.14 (m, 2H), 7.00 (d, *J* = 7.6 Hz, 1H), 4.35 (br, 1H), 3.02–2.80 (m), 2.75 (dd, *J* = 16.4, 9.8 Hz, 1H), 2.32 (s, 3H), 2.29 (m, 1H), 2.25 (s, 3H), 1.98 (m, 1H). ^13^C NMR (CDCl_3_) δ 163.7, 150.2, 148.8, 148.3, 148.3, 139.2, 138.0, 137.7, 137.0, 136.8, 136.7, 136.0, 135.0, 134.6, 132.3, 131.2, 130.8, 128.0, 127.8, 126.6, 123.3, 122.0, 45.6, 35.5, 29.2, 26.3, 20.5, 20.1. HRMS calcd. for C_29_H_26_ClN_3_O 468.1854, found 468.1856.

#### 4.1.22 (S)-5-(4-Chloro-2-methylphenyl)-N-(5-methyl-8-(1H-pyrazol-5-yl)-1,2,3,4-tetrahydronaphthalen-2-yl)picolinamide (**77**)

**126** was obtained from **97** and (1*H*-pyrazol-5-yl)boronic acid **121** using the general procedure B to give **126** (78%) as a crude product. This was immediately dissolved in MeOH (40 mL) and hydrogenated over 10% Pd–C (0.30 g) at 60 psi for 72 h. The catalyst was filtered off and the filtrate concentrated to dryness to give pure **131** as a colorless, viscous oil which was used directly for the next step.

The title compound was obtained from **131** and 5-(4-chloro-2-methylphenyl)picolinic acid **83** using the general procedure A to give **77** (46%) as a white foam. HPLC 98.7%. mp 147–150 °C. ^1^H NMR (CDCl_3_) δ 8.45 (d, *J* = 1.5 Hz, 1H), 8.23 (d, *J* = 8.1 Hz, 1H), 8.06 (d, *J* = 8.1 Hz, 1H), 7.77 (dd, *J* = 8.0, 2.1 Hz, 1H), 7.60 (d, *J* = 2.0 Hz, 1H), 7.33–7.24 (m, 2H), 7.22 (d, *J* = 7.7 Hz, 1H), 7.17–7.10 (m, 2H), 6.38 (d, *J* = 1.9 Hz, 1H), 4.45–4.36 (m, 1H), 3.26 (dd, *J* = 16.4, 4.2 Hz, 1H), 2.97–2.82 (m, 3H), 2.33–2.25 (m, 1H), 2.30 (s, 3H), 2.23 (s, 3H), 1.98–1.88 (m, 1H) (NH not observed). ^13^C NMR (CDCl_3_) δ 163.8, 148.8, 148.2, 139.1, 138.0, 137.7, 137.2, 136.0, 135.0, 134.5, 132.9, 131.2, 130.8, 129.8, 127.9, 127.4, 126.6, 122.0, 106.1, 45.6, 35.1, 28.9, 26.1, 20.5, 20.1. LRMS Found: [M+H] = 457.2. HRMS calcd. for C_27_H_25_ClN_4_O (M + H^+^) *m/z*: 456.1717 found 456.1730.

#### 4.1.23 (S)-5-(4-Chloro-2-methylphenyl)-N-(5-methyl-8-(1-methyl-1H-pyrazol-4-yl)-1,2,3,4-tetrahydronaphthalen-2-yl)picolinamide (**79**)

**128** was obtained from **97** and (1-methyl-1*H*-pyrazol-4-yl)boronic acid **123** using the general procedure B to give **128** (43%) as a crude product. This was immediately dissolved in MeOH (40 mL) and hydrogenated over 10% Pd–C (0.30 g) at 60 psi for 72 h. The catalyst was filtered off and the filtrate concentrated to dryness to give pure **133** as a colorless, viscous oil which was used directly for the next step.

The title compound was obtained from **133** and 5-(4-chloro-2-methylphenyl)picolinic acid **83** using the general procedure A to give **79** (74%) as a white foam. HPLC 98.7%. mp 76–79 °C. ^1^H NMR (CDCl_3_) δ 8.47 (dd, *J* = 2.1, 0.7 Hz, 1H), 8.24 (dd, *J* = 8.0, 0.6 Hz, 1H), 8.07 (d, *J* = 8.3 Hz, 1H), 7.77 (dd, *J* = 8.0, 2.2 Hz, 1H), 7.52 (d, *J* = 0.5 Hz, 1H), 7.38 (s, 1H), 7.33–7.26 (m, 2H), 7.14 (d, *J* = 8.2 Hz, 1H), 7.11–7.05 (m, 2H), 4.45–4.36 (m, 1H), 3.93 (s, 3H), 3.23 (dd, *J* = 16.0, 4.1 Hz, 1H), 2.93–2.76 (m, 3H), 2.33–2.26 (m, 1H), 2.29 (s, 3H), 2.24 (s, 3H), 1.98–1.88 (m, 1H). ^13^C NMR (CDCl_3_) δ 163.7, 148.9, 148.3, 139.4, 139.2, 138.0, 137.7, 136.0, 135.6, 134.8, 134.6, 132.3, 131.2, 130.9, 130.8, 129.1, 127.8, 127.5, 126.7, 122.0, 121.9, 45.7, 39.2, 35.6, 29.0, 26.2, 20.5, 20.0. LRMS Found: [M+H] = 471.1. HRMS calcd. for C_28_H_27_ClN_4_O: 470.1873, found 470.1885.

## Declaration of competing interest

The authors declare that they have no known competing financial interests or personal relationships that could have appeared to influence the work reported in this paper.
