# Supplementary material for: Synthesis and structure-activity relationships for a new class of tetrahydronaphthalene amide inhibitors of Mycobacterium tuberculosis
Source: Eur J Med Chem. 2022 Feb 5;229:114059. doi: 10.1016/j.ejmech.2021.114059 (PMC8811485; doi:10.1016/j.ejmech.2021.114059)
Supplement: Multimedia component 1 [file mmc1.docx]

**Supplementary Data**

Synthesis and structure-activity relationships for a new class of tetrahydronaphthalene amide inhibitors of *Mycobacterium tuberculosis*

Hamish S. Sutherland^1,2^, Guo-Liang Lu^1^, Amy S.T. Tong^1^, Daniel Conole^1^, Scott G. Franzblau^3^, Anna M. Upton^4^, Manisha U. Lotlikar^4^, Christopher B. Cooper^4^, Brian D. Palmer^1,2^, Peter J. Choi^1,2^*, William A. Denny^1,2^

^1^ Auckland Cancer Society Research Centre, School of Medical Sciences, University of Auckland, Private Bag 92019, Auckland 1142, New Zealand

^2^ Maurice Wilkins Centre, University of Auckland, Private Bag 92019, Auckland 1142, New Zealand

^3^ Institute for Tuberculosis Research, College of Pharmacy, University of Illinois at Chicago, 833 South Wood Street, Chicago, Illinois 60612, USA

^4^ Global Alliance for TB Drug Development, 40 Wall St, New York 10005, USA

***** *Corresponding author*.

Email address: [p.choi@auckland.ac.nz](mailto:p.choi@auckland.ac.nz)

**CONTENTS**

Fig S1 2

Fig S2 3

General Procedure and experimental 4 - 49

^1^H and ^13^C NMR spectra of final compounds 50 -103


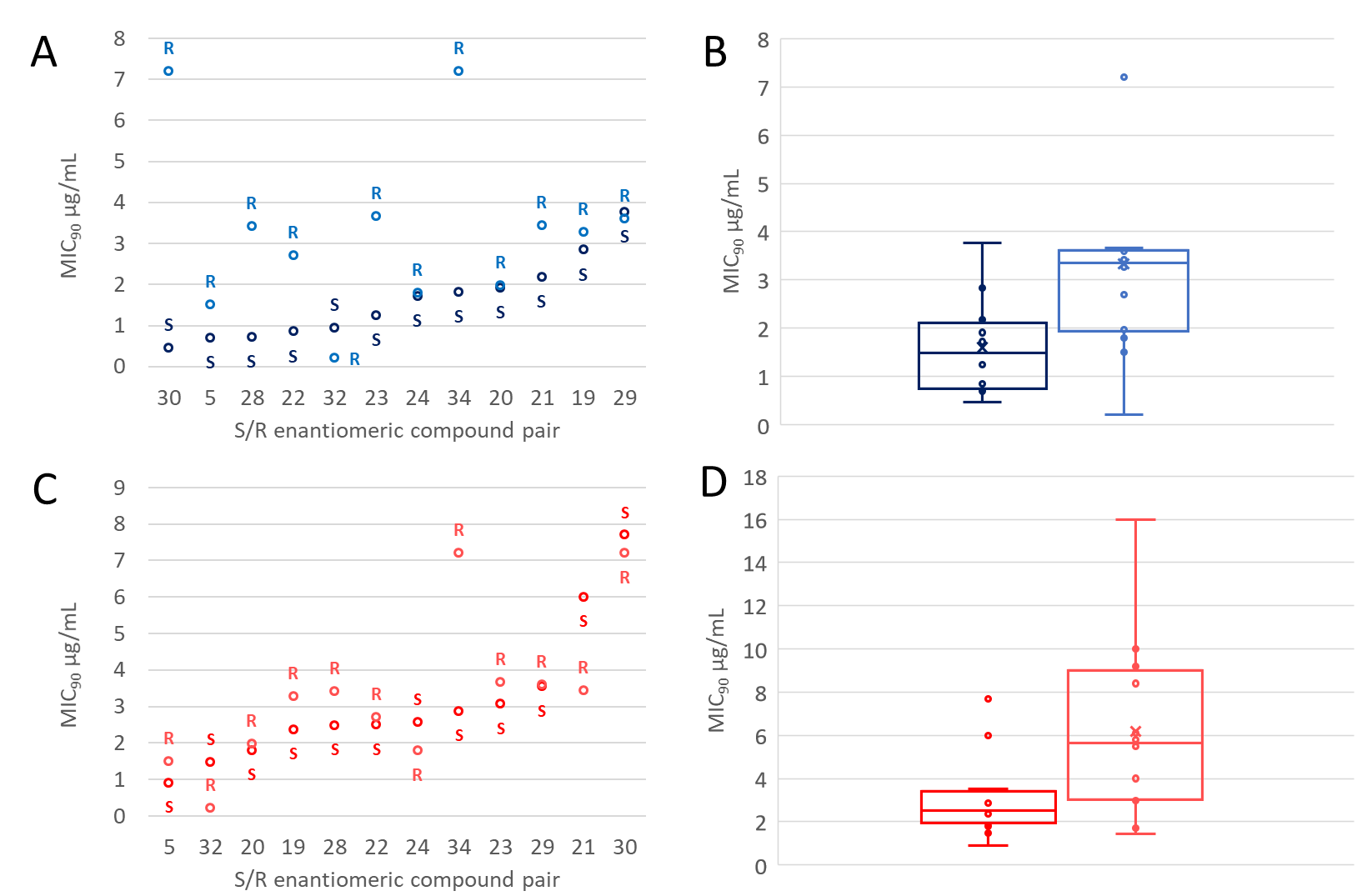


**Fig. S1:** ***S* stereochemical configuration for THNAs offers slightly increased *M.tb* inhibition compared with *R*.** Figure depicts the MIC_90_ values (µg/mL) (MABA (blue, **A** and **B**) and LORA (red, **C** and **D**)) of 12 enantiomeric *R*/*S* THNA pairs (*R* = light blue/red, *S* = dark blue/red) as measures of their M.tb inhibition activity. Panels **A** and **C** display the MIC_90_ values for individual matched pairs, whereas panels **B** and **D** show the box and whisker plots representative of the general activity trend for R and S stereochemical configurations. Mean values are noted by an X on the box and whisker plot.


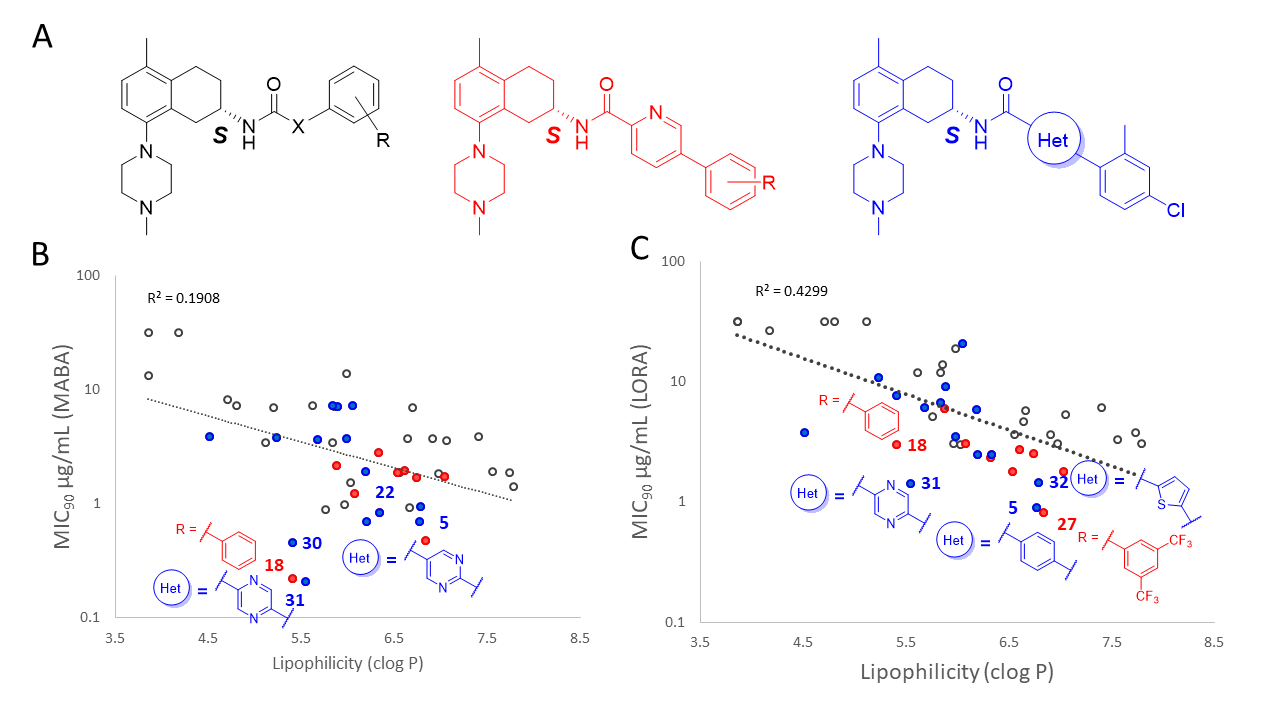


**Fig. S2:** **A** – Colour code for structural analogues and matched pairs investigated in Table 1. THNAs increased in MABA (**B**) and LORA (**C**) *M.tb* inhibition potency with elevated lipophilicity, with the exceptions of **5**, **18**, **27**, **30**, **31** and **32**.

**4. Experimental section**

***4.1. General information***

Final products were analysed by reverse-phase HPLC (Alltima C18 5 µm column, 15 × 3.2 mm; Alltech Associated, Inc., Deerfield, IL) using an Agilent HP1100 equipped with a diode-array detector. Mobile phases were gradients of 80% CH_3_CN/20% H_2_O (v/v) in 45 mM NH_4_HCO_2_ at pH 3.5 and 0.5 mL/min. Purity was determined by monitoring at 330 ± 50 nm and was ≥95% for all final products. Melting points were determined on an Electrothermal 9100 melting point apparatus. NMR spectra were obtained on a Bruker Avance 400 spectrometer at 400 MHz for ^1^H. Low-resolution atmospheric pressure chemical ionization (APCI) mass spectra were measured for solutions on a ThermoFinnigan Surveyor MSQ mass spectrometer, connected to a Gilson autosampler. High resolution mass spectra were obtained using an Agilent G6530B Q-TOF spectrometer, and are reported for M+H.

*4.1.1. General procedure A:* *(S)-5-(4-Chloro-2-methylphenyl)-N-(5-methyl-8-(4-methylpiperazin-1-yl)-1,2,3,4-tetrahydronaphthalen-2-yl)picolinamide (****5****)*

A solution of 5-(4-chloro-2-methylphenyl)picolinic acid **83** (0.184 g, 0.746 mmol) in DMF (15 mL) was purged with nitrogen before DIPEA (0.48 ml, 2.8 mmol) was added to the reaction mixture. HATU (0.285 g, 0.750 mmol) was added and stirred for 15 mins. (*S*)-5-methyl-8-(4-methylpiperazin-1-yl)-1,2,3,4-tetrahydronaphthalen-2-amine **80** (0.259 g, 0.679 mmol) was added to the reaction mixture and stirred at r.t. for 40 h. The reaction mixture was diluted with EtOAc and washed with water and 2M NaOH solution. The organic layer was dried over anhydrous Na_2_SO_4_ and filtered through a pad of Celite. The solvent was removed to give the crude product, which was purified by silica column chromatography using MeOH (0-5% v/v) in EtOAc as eluent to give **5** (0.287 g, 87%) as a white foam. HPLC 99.1%. ^1^H NMR (CDCl_3_) δ 7.81 (ap d, J = 8.4 Hz, 2H), 7.35 (ap d, J = 8.4 Hz, 2H), 7.28 (d, J = 2.0 Hz, 1H), 7.23 (dd, J = 7.9, 1.7 Hz, 1H), 7.13 (d, J = 8.2 Hz, 1H), 7.04 (d, J = 8.0 Hz, 1H), 6.93 (d, J = 8.0 Hz, 1H), 6.13 (d, J = 7.8 Hz, 1H), 4.45-4.54 (m, 1H), 3.29 (dd, J = 16.5, 4.5 Hz, 1H), 2.85-2.94 (m, 4H), 2.81 (t, J = 6.7 Hz, 2H), 2.72 (dd, J = 16.5, 8.0 Hz, 1H), 2.57 (br, 4H), 2.35 (s, 3H), 2.23 (s, 3H), 2.22 (s, 3H), 2.22 (br, 1H), 1.89-1.99 (m, 1H). ^13^C NMR (CDCl_3_) δ 167.0, 150.0, 144.2, 139.5, 137.4, 135.2, 133.9, 133.7, 132.1, 131.0, 130.5, 129.7, 129.5, 128.2, 127.1, 126.2, 117.4, 55.8, 52.3, 46.3, 45.6, 31.9, 28.8, 25.6, 20.5, 19.6. HRMS calcd. for C_30_H_34_ClN_3_O: 487.2390, found 487.2405.

*4.1.2. (R)-5-(4-Chloro-2-methylphenyl)-N-(5-methyl-8-(4-methylpiperazin-1-yl)-1,2,3,4-tetrahydronaphthalen-2-yl)picolinamide (****5R****)*

The title compound was obtained from (*R*)-5-methyl-8-(4-methylpiperazin-1-yl)-1,2,3,4-tetrahydronaphthalen-2-amine **80R** and **83** using the general procedure A to give **5*R*** (69%) as a white foam. HPLC 98.5%. ^1^H NMR (CDCl_3_) δ 7.81 (ap d, J = 8.4 Hz, 2H), 7.35 (ap d, J = 8.4 Hz, 2H), 7.28 (d, J = 2.0 Hz, 1H), 7.23 (ddd, J = 8.2, 2.0, 0.4 Hz, 1H), 7.13 (d, J = 8.2 Hz, 1H), 7.04 (d, J = 8.0 Hz, 1H), 6.93 (d, J = 8.0 Hz, 1H), 6.14 (d, J = 7.8 Hz, 1H), 4.45-4.54 (m, 1H), 3.29 (dd, J = 16.5, 4.6 Hz, 1H), 2.85-2.94 (m, 4H), 2.81 (t, J = 6.7 Hz, 2H), 2.72 (dd, J = 16.5, 8.0 Hz, 1H), 2.57 (br, 4H), 2.35 (s, 3H), 2.23 (s, 3H), 2.22 (s, 3H), 2.22 (br, 1H), 1.89-1.99 (m, 1H). ^13^C NMR (CDCl_3_) δ 167.0, 150.0, 144.2, 139.5, 137.4, 135.2, 133.9, 133.7, 132.1, 131.0, 130.5, 129.7, 129.5, 128.2, 127.1, 126.2, 117.4, 55.8, 52.3, 46.3, 45.6, 31.9, 28.8, 25.6, 20.5, 19.6. HRMS calcd. for C_30_H_34_ClN_3_O: 487.2390, found 487.2400.

*4.1.3. (S)-2-(4'-(2-Methoxyethoxy)-2'-methyl-[1,1'-biphenyl]-4-yl)-N-(5-methyl-8-(4-methylpiperazin-1-yl)-1,2,3,4-tetrahydronaphthalen-2-yl)acetamide (****6****)*

A mixture of (4-(methoxycarbonyl)phenyl)boronic acid (2.90 g, 16.1 mmol), 4-bromo-3-methylphenol (3.00 g, 16.0 mmol) and Cs_2_CO_3_ (10.5 g, 32.2 mmol) in anhydrous DMF (50 mL) was purged with nitrogen. Pd(dppf)Cl_2_.DCM (0.655 g, 0.80 mmol) was added and the mixture was heated to 75 °C under nitrogen in a sealable tube for 2 h. The mixture was partitioned between EtOAc and water, the organic fraction was dried and evaporated. Column chromatography (0-5% EtOAc:DCM) gave methyl 4ˈ-hydroxy-2ˈ-methyl-[1,1ˈ-biphenyl]-4-carboxylate (**134**) (2.89 g, 74%) as a tan solid. mp 166-167 °C. ^1^H NMR (CDCl_3_) δ 8.08 (ap d, J = 8.5 Hz, 2H), 7.39 (ap d, J = 8.5 Hz, 2H), 7.13 (d, J = 8.2 Hz, 1H), 6.80 (d, J = 2.6 Hz, 1H), 6.74 (dd, J = 8.2, 2.6 Hz, 1H), 5.22 (s, 1H), 3.96 (s, 3H), 2.25 (s, 3H). LRMS Found: [M+H]= 243.1.

Bromo-2-methoxyethane (0.47 mL, 50.0 mmol) was added to a mixture of **134** (1.018 g, 4.20 mmol) and K_2_CO_3_ (91.45 g, 10.5 mmol) in anhydrous DMF (20 mL). The mixture was stirred for 16 h, and then partitioned between EtOAc and water. The organic fraction was dried and evaporated, silica column chromatography (2:1 hexanes:DCM) gave methyl 4ˈ-(2-methoxyethoxy)-2ˈ-methyl-[1,1ˈ-biphenyl]-4-carboxylate (**135**) (1.136 g, 90%) as a colourless oil. ^1^H NMR (CDCl_3_) δ 8.06 (ap d, J = 8.5 Hz, 2H), 7.37 (ap d, J = 8.6 Hz, 2H), 7.15 (d, J = 8.4 Hz, 1H), 6.86 (d, J = 2.7 Hz, 1H), 6.83 (dd, J = 8.4, 2.5 Hz, 1H), 4.14-4.17 (m, 2H), 3.94 (s, 3H), 3.76-3.79 (m, 2H), 3.47 (s, 3H), 2.25 (s, 3H). LRMS Found: [M+H]= 301.1.

A solution of **135** (0.701 g, 2.45 mmol) in THF (20 mL), MeOH (20 mL) and water (10 mL) was treated with LiOH (0.76 g, 32 mmol). The solution was stirred at room temperature for 16 h, LiOH (0.76 g, 32 mmol) was added and stirring was continued for another 2 h. The solvent was evaporated and the residue was dissolved in water (50 mL), 2M HCl was added until pH 2 was reached, the resulting white precipitate was filtered, washed with water and dried to give 4ˈ-(2-methoxyethoxy)-2ˈ-methyl-[1,1ˈ-biphenyl]-4-carboxylic acid (**136**) (0.655 g, 98%) as a white solid. mp 130-131 ^o^C. ^1^H NMR ((CD_3_)_2_SO) δ 12.92 (bs, 1H), 7.98 (ap d, J = 8.4 Hz, 2H), 7.43 (ap d, J =8.4 Hz, 2H), 7.15 (d, J = 8.4 Hz, 1H), 6.91 (d, J = 2.5 Hz, 1H), 6.86 (dd, J = 8.4, 2.5 Hz, 1H), 4.11-4.13 (m, 2H), 3.66-3.88 (m, 2H), 2.22 (s, 3H). LRMS Found: [M-H]= 285.1.

The title compound was obtained from (*S*)-5-methyl-8-(4-methylpiperazin-1-yl)-1,2,3,4-tetrahydronaphthalen-2-amine **80** and **136** using the general procedure A to give **6** (85%) as a white foam. ^1^H NMR (CDCl_3_) δ 7.79 (ap d, J = 8.4 Hz, 2H), 7.36 (ap d, J = 8.4 Hz, 2H), 7.12 (d, J = 8.4 Hz, 1H), 7.04 (d, J = 8.1 Hz, 1H), 6.92 (d, J = 8.0 Hz, 1H), 6.86 (d, J = 2.6 Hz, 1H), 6.82 (dd, J = 8.4, 2.6 Hz, 1H), 6.14 (d, J = 7.8 Hz, 1H), 4.47-4.51 (m, 1H), 4.14-4.17 (m, 2H), 3.76-3.78 (m, 2H), 3.47 (s, 3H), 3.28 (dd, J = 16.4, 4.6 Hz, 1H), 2.87 (m, 4H), 2.81 (t, J = 6.6 Hz, 2H), 2.71 (dd, J = 16.5, 8.1 Hz, 1H), 2.57 (bs, 4H), 2.35 (s, 3H), 2.24 (s, 3H), 2.22 (s, 4H), 2.21 (s, 3H), 1.93 (m, 1H). ^13^C NMR (CDCl_3_) δ 167.1, 158.5, 150.0, 145.1, 136.8, 135.2, 133.9, 133.2, 132.0, 130.9, 129.8, 129.8, 129.7, 128.2, 126.9, 117.4, 116.9, 112.1, 71.3, 67.5, 59.5, 55.9, 52.3, 46.4, 45.6, 31.9, 28.9, 25.6, 20.9, 19.6. HRMS calcd. for C_33_H_41_N_3_O_3_: 527.3148, found 527.3161.

*4.1.4. (S)-2-(4'-Chloro-2'-methyl-[1,1'-biphenyl]-4-yl)-N-(5-methyl-8-(4-methylpiperazin-1-yl)-1,2,3,4-tetrahydronaphthalen-2-yl)acetamide (****7****)*

A mixture 2-(4-bromophenyl)acetic acid (0.406 g, 1.89 mmol), (4-chloro-2-methylphenyl)boronic acid (0.482 g, 2.83 mmol) and Cs_2_CO_3_ (0.922 g, 2.83 mmol) in toluene (20 mL) was purged with nitrogen. Pd(PPh_3_)_4_ (0.065 g, 0.057 mmol) was added, the mixture was purged with nitrogen then heated to 95 °C under nitrogen for 20 h. The reaction was partitioned between EtOAc and water and the organic fraction was dried and evaporated. Column chromatography with EtOAc:hexanes (1:9) to give 2-(4'-chloro-2'-methyl-[1,1'-biphenyl]-4-yl)acetic acid **137** (0.182 g, 37%). mp 124-127°C. ^1^H NMR ((CD_3_)_2_SO) 400 MHz) δ 12.4 (s, 1H), 7.39 (d, J = 8.3 Hz, 1H), 7.36-7.31 (m, 3H), 7.30-7.26 (m, 3H), 7.21 (d, J = 8.2 Hz, 1H), 3.56 (s, 2H), 2.23 (s, 3H). LRMS Found: [M-H]^-^= 259.1.

The title compound was obtained from (*S*)-5-methyl-8-(4-methylpiperazin-1-yl)-1,2,3,4-tetrahydronaphthalen-2-amine **80** and **137** using the general procedure A to give **7** (77%) as a white foam. HPLC 97.1%. mp 94-96°C. ^1^H NMR (CDCl_3_) δ 7.30-7.19 (m, 6H), 7.12 (d, J = 8.2 Hz, 1H), 7.00 (d, J = 8.1 Hz, 1H), 6.87 (d, J = 8.0 Hz, 1H), 5.47 (d, J = 7.8 Hz, 1H), 4.32-4.23 (m, 1H), 3.59 (s, 2H), 3.08 (dd, J = 16.4, 4.4 Hz, 1H), 2.87-2.84 (m, 4H), 2.75-2.53 (m, 7H), 2.38 (s, 3H), 2.21 (s, 3H), 2.15 (s, 3H), 2.08-1.98 (m, 1H), 1.83-1.75 (m, 1H). HRMS calcd. for C_31_H_36_ClN_3_O: 501.2547, found 501.2555.

*4.1.5. (S)-2-(3',5'-Bis(trifluoromethyl)-[1,1'-biphenyl]-4-yl)-N-(5-methyl-8-(4-methylpiperazin-1-yl)-1,2,3,4-tetrahydronaphthalen-2-yl)acetamide (****8****)*

2-(4-Bromophenyl)acetic acid (0.534 g, 2.48 mmol), (3,5-bis(trifluoromethyl)phenyl)boronic acid (1.28 g, 4.97 mmol), Cs_2_CO_3_ (1.21 g, 3.73 mmol) in toluene (20 mL) was purged with nitrogen. Pd(PPh_3_)_4_ (0.086 g, 0.075 mmol) was added, the mixture was purged with nitrogen then heated to 95 °C under nitrogen for 17 h. The reaction was partitioned between EtOAc and water and the organic fraction was dried and evaporated. Column chromatography with EtOAc:hexanes (1:9) to give 2-(3',5'-bis(trifluoromethyl)-[1,1'-biphenyl]-4-yl)acetic acid **138** (23%). mp 155-158°C. ^1^H NMR ((CD_3_)_2_SO) 400 MHz) δ 12.4 (s, 1H), 8.32 (s, 2H), 8.08 (s, 1H), 7.82 (d, J = 8.3 Hz, 2H), 7.42 (d, J = 8.3 Hz, 2H), 3.66 (s, 2H).

The title compound was obtained from (*S*)-5-methyl-8-(4-methylpiperazin-1-yl)-1,2,3,4-tetrahydronaphthalen-2-amine **80** and **138** using the general procedure A to give **8** (77%) as a white foam. HPLC 93.8%. mp 125-128°C. ^1^H NMR (CDCl_3_) δ 7.97 (s, 2H), 7.87 (s, 1H), 7.53 (d, J = 8.3 Hz, 2H), 7.39 (d, J = 8.3 Hz, 2H), 6.96 (d, J = 8.1 Hz, 1H), 6.81 (d, J = 8.0 Hz, 1H), 5.52 (d, J = 8.0 Hz, 1H), 4.28-4.20 (m, 1H), 3.61 (s, 2H), 3.08 (dd, J = 16.5, 4.6 Hz, 1H), 2.95-2.73 (m, 8H), 2.68-2.51 (m, 3H), 2.53 (s, 3H), 2.12 (s, 3H), 2.08-1.98 (m, 1H), 1.82-1.71 (m, 1H). HRMS calcd. for [M+H] C_32_H_34_F_6_N_3_O: 590.2601, found 590.2585.

*4.1.6. (S)-N-(5-Methyl-8-(4-methylpiperazin-1-yl)-1,2,3,4-tetrahydronaphthalen-2-yl)-4-phenoxybenzamide (****9****)*

The title compound was obtained from (*S*)-5-methyl-8-(4-methylpiperazin-1-yl)-1,2,3,4-tetrahydronaphthalen-2-amine **80** and 4-phenoxybenzoic acid using the general procedure A to give **9** (79%) as a white foam. HPLC 97.3%. mp 208-211°C. ^1^H NMR (CDCl_3_) δ 7.76-7.71 (m, 2H), 7.39-7.33 (m, 2H), 7.19-7.14 (m, 1H), 7.06-6.98 (m, 5H), 6.91 (d, J = 8.0 Hz, 1H), 6.03 (d, J = 7.8 Hz, 1H), 4.49-4.41 (m, 1H), 3.27 (dd, J = 16.5, 4.6 Hz, 1H), 2.95-2.86 (m, 4H), 2.81-2.58 (m, 7H), 2.41 (s, 3H), 2.23-2.17 (m, 1H), 2.21 (s, 3H), 1.95-1.85 (m, 1H). HRMS calcd. for C_29_H_33_N_3_O_2_: 455.2573, found 455.2583.

*4.1.7. (S)-4-(4-Fluoro-2-methylphenoxy)-N-(5-methyl-8-(4-methylpiperazin-1-yl)-1,2,3,4-tetrahydronaphthalen-2-yl)benzamide (****10****)*

The title compound was obtained from (*S*)-5-methyl-8-(4-methylpiperazin-1-yl)-1,2,3,4-tetrahydronaphthalen-2-amine **80** and 4-(4-fluoro-2-methylphenoxy)benzoic acid using the general procedure A to give **10** (81%) as a white foam. HPLC 97.3%. mp 192-194°C. ^1^H NMR (CDCl_3_) δ 7.73-7.68 (m, 2H), 7.03 (d, J = 8.0 Hz, 1H), 6.98 (dd, J = 8.8, 2.5 Hz, 1H), 6.94-6.89 (m, 3H), 6.88-6.83 (m, 2H), 6.00 (d, J = 7.8 Hz, 1H), 4.49-4.40 (m, 1H), 3.24 (dd, J = 16.5, 4.5 Hz, 1H), 2.43-2.35 (m, 4H), 2.78 (t, J = 6.6 Hz, 2H), 2.72-2.50 (m, 5H), 2.37 (s, 3H), 2.22-2.13 (m, 1H), 2.20 (s, 3H), 2.26 (s, 3H), 1.95-1.85 (m, 1H). HRMS calcd. for C_30_H_34_FN_3_O_2_: 487.2635, found 487.2661.

*4.1.8. (S)-4-(4-Chloro-2-methylphenoxy)-N-(5-methyl-8-(4-methylpiperazin-1-yl)-1,2,3,4-tetrahydronaphthalen-2-yl)benzamide (****11****)*

The title compound was obtained from (*S*)-5-methyl-8-(4-methylpiperazin-1-yl)-1,2,3,4-tetrahydronaphthalen-2-amine **80** and 4-(4-chloro-2-methylphenoxy)benzoic acid using the general procedure A to give **11** (62%) as a white foam. HPLC 99.3%. mp 204-207°C. ^1^H NMR (CDCl_3_, 400 MHz) δ 7.73-7.68 (m, 2H), 7.16 (dd, J = 8.6, 2.6 Hz, 1H), 7.03 (d, J = 8.1 Hz, 1H), 6.94-6.85 (m, 5H), 6.00 (d, J = 7.8 Hz, 1H), 4.47-4.42 (m, 1H), 3.24 (dd, J = 16.6, 4.8 Hz, 1H), 2.93-2.83 (m, 4H), 2.78 (t, J = 6.6 Hz, 2H), 2.72-2.50 (m, 5H), 2.34 (s, 3H), 2.22-2.16 (m, 1H), 2.20 (s, 3H), 2.17 (s, 3H), 1.95-1.87 (m, 1H). HRMS calcd. for C_30_H_34_ClN_3_O_2_: 503.2340, found 503.2353.

*4.1.9. (S)-4-(2-Fluorophenoxy)-N-(5-methyl-8-(4-methylpiperazin-1-yl)-1,2,3,4-tetrahydronaphthalen-2-yl)benzamide (****12****)*

The title compound was obtained from (*S*)-5-methyl-8-(4-methylpiperazin-1-yl)-1,2,3,4-tetrahydronaphthalen-2-amine **80** and 4-(2-fluorophenoxy)benzoic acid using the general procedure A to give **12** (79%) as a white foam. HPLC 97.3%. mp 220-222°C. ^1^H NMR (CDCl_3_, 400 MHz) δ 7.74-7.69 (m, 2H), 7.22-7.09 (m, 4H), 7.03 (d, J = 8.1 Hz, 1H), 6.99-6.89 (m, 3H), 6.00 (d, J = 7.7 Hz, 1H), 4.49-4.41 (m, 1H), 3.24 (dd, J = 16.5, 4.7 Hz, 1H), 2.93-2.83 (m, 4H), 2.78 (t, J = 6.6 Hz, 2H), 2.72-2.50 (m, 5H), 2.36 (s, 3H), 2.23-2.16 (m, 1H), 2.20 (s, 3H), 1.95-1.85 (m, 1H). HRMS calcd. for C_29_H_32_FN_3_O_2_: 473.2479, found 473.2500.

*4.1.10. (S)-4-(3,5-Bis(trifluoromethyl)phenoxy)-N-(5-methyl-8-(4-methylpiperazin-1-yl)-1,2,3,4-tetrahydronaphthalen-2-yl)benzamide (****13****)*

The title compound was obtained from (*S*)-5-methyl-8-(4-methylpiperazin-1-yl)-1,2,3,4-tetrahydronaphthalen-2-amine **80** and 4-(3,5-bis(trifluoromethyl)phenoxy)benzoic acid using the general procedure A to give **13** (74%) as a white foam. HPLC 98.4%. mp 191-193°C. ^1^H NMR (CDCl_3_, 400 MHz) δ 7.86-7.81 (m, 2H), 7.62 (s, 1H), 7.42 (s, 2H), 7.11-7.03 (m, 3H), 6.92 (J = 8.0 Hz, 1H), 6.08 (d, J = 7.8 Hz, 1H), 4.51-4.43 (m, 1H), 3.28 (dd, J = 16.4, 4.5 Hz, 1H), 2.96-2.83 (m, 4H), 2.80 (t, J = 6.7 Hz, 2H), 2.75-2.53 (m, 5H), 2.38 (s, 3H), 2.25-2.19 (m, 1H), 2.21 (s, 3H), 1.98-1.88 (m, 1H). HRMS calcd. for C_31_H_31_FN_3_O_2_: 591.2321, found 591.2352.

*4.1.11. (S)-4-((4-Chlorophenyl)thio)-N-(5-methyl-8-(4-methylpiperazin-1-yl)-1,2,3,4-tetrahydronaphthalen-2-yl)benzamide (****14****)*

The title compound was obtained from (*S*)-5-methyl-8-(4-methylpiperazin-1-yl)-1,2,3,4-tetrahydronaphthalen-2-amine **80** and 4-((4-chlorophenyl)thio)benzoic acid using the general procedure A to give **14** (46%) as a white foam. HPLC 99.4%. mp 87-90°C. ^1^H NMR (CDCl_3_, 400 MHz) δ 7.65 (d, J = 8.4 Hz, 2H), 7.36-7.30 (m, 4H), 7.28-7.23 (m, 2H), 7.03 (d, J = 8.1 Hz, 1H), 6.91 (d, J = 8.0 Hz, 1H), 6.07 (d, J = 7.8 Hz, 1H), 4.48-4.39 (m , 1H), 3.24 (dd, J = 16.4, 4.5 Hz, 1H), 2.98-2.88 (m, 4H), 2.78 (t, J = 6.7 Hz, 2H), 2.77-2.62 (m, 5H), 2.44 (s, 3H), 2.22-2.15 (m, 1H), 2.20 (s, 3H), 1.95-1.84 (m, 1H). HRMS calcd. for C_29_H_32_ClN_3_OS: 505.1955, found 505.1972.

*4.1.12. (S)-4-((2,4-Dimethylphenyl)thio)-N-(5-methyl-8-(4-methylpiperazin-1-yl)-1,2,3,4-tetrahydronaphthalen-2-yl)benzamide (****15****)*

2,4-Dimethylbenzenethiol (0.60 mL, 4.43 mmol), 4-fluorobenzonitrile (0.537 g, 4.43 mmol) and K_2_CO_3_ (0.919 g, 6.65 mmol) in DMF (20 mL) was heated at 80 ^o^C for 3 h. The reaction was partitioned between EtOAc and water and the organic fraction was dried was dried with Na_2_SO_4_ and evaporated. Column chromatography with 5:95 EtOAc/hexanes gave an impure product which was rechromatographed using a gradient of hexanes:DCM(2:1) to hexanes:DCM(1:1) to give 4-((2,4-dimethylphenyl)thio)benzonitrile **138** (0.694 g, 65%). mp 155-158 ^o^C. ^1^H NMR (CDCl_3_, 400 MHz) δ 7.46-7.40 (m, 3H), 7.18 (s, 1H), 7.08 (d, J = 7.8 Hz, 1H), 7.03-7.00 (m, 2H), 2.38 (s, 3H), 2.31 (s, 3H).

4-((2,4-Dimethylphenyl)thio)benzonitrile **138** (0.285 g, 1.19 mmol) was added to KOH (0.50 g, 8.91 mmol) in a mixture of EtOH:water (1:1 mixture, 4 mL). The reaction was refluxed for 3 h. The organic solvent was evaporated to give a crude mixture. Residue was dissolved in water, acidified to pH1 using 2M HCl (aq) solution. The resulting white precipitate was collected and washed with water to give 4-((2,4-dimethylphenyl)thio)benzoic acid **139** (0.084g, 24%). mp 175-177 ^o^C. 1H NMR ((CD_3_)_2_SO) 400 MHz) δ 12.9 (s, 1H), 7.83-7.79 (m, 2H), 7.41 (d, J = 7.8 Hz, 1H), 7.27 (s, 1H), 7.14-7.11 (m, 1H), 7.07-7.02 (m, 2H), 2.33 (s, 3H), 2.26 (s, 3H). LRMS Found: [M-H]-= 257.1.

The title compound was obtained from (*S*)-5-methyl-8-(4-methylpiperazin-1-yl)-1,2,3,4-tetrahydronaphthalen-2-amine **80** and 4-((2,4-dimethylphenyl)thio)benzonitrile **139** using the general procedure A to give **15** (60%) as a white foam. HPLC 96.8%. mp 203-205°C. ^1^H NMR (CDCl_3_, 400 MHz) δ 7.60-7.56 (m, 2H), 7.38 (d, J = 7.8 Hz, 1H), 7.14 (s, 1H), 7.08-7.00 (m, 4H), 6.90 (d, J = 8.0 Hz, 1H), 6.00 (d, J = 7.8 Hz, 1H), 4.48-4.38 (m , 1H), 3.24 (dd, J = 16.6, 4.5 Hz, 1H), 2.98-2.85 (m, 4H), 2.78 (t, J = 6.6 Hz, 2H), 2.70-2.50 (m, 5H), 2.36 (s, 6H), 2.31 (s, 3H), 2.19 (s, 3H), 2.19-2.14 (m, 1H), 1.93-1.84 (m, 1H). HRMS calcd. for C_31_H_37_N_3_OS: 499.2679, found 499.2657.

*4.1.13. (S)-4-((4-Chlorophenyl)sulfonyl)-N-(5-methyl-8-(4-methylpiperazin-1-yl)-1,2,3,4-tetrahydronaphthalen-2-yl)benzamide (****16****)*

The title compound was obtained from (*S*)-5-methyl-8-(4-methylpiperazin-1-yl)-1,2,3,4-tetrahydronaphthalen-2-amine **80** and 4-((4-chlorophenyl)sulfonyl)benzonitrile using the general procedure A to give **16** (83%) as a white foam. HPLC 99.3%. mp 97-100°C. ^1^H NMR (CDCl_3_, 400 MHz) δ 7.99-7.95 (m, 2H), 7.89-7.82 (m, 4H), 7.51-7.46 (m, 2H), 7.03 (d, J = 8.0 Hz, 1H), 6.91 (d, J = 8.0 Hz, 1H), 6.08 (d, J = 7.8 Hz, 1H), 4.48-4.39 (m , 1H), 3.24 (dd, J = 16.4, 4.5 Hz, 1H), 2.89-2.83 (m, 4H), 2.78 (t, J = 6.7 Hz, 2H), 2.73-2.48 (m, 5H), 2.32 (s, 3H), 2.22-2.15 (m, 1H), 2.20 (s, 3H), 1.96-1.88 (m, 1H). HRMS calcd. for C_29_H_32_ClN_3_O_3_S: 537.1871, found 537.1853.

*4.1.14. (S)-4-((2,4-Dimethylphenyl)sulfonyl)-N-(5-methyl-8-(4-methylpiperazin-1-yl)-1,2,3,4-tetrahydronaphthalen-2-yl)benzamide (****17****)*

4-((2,4-Dimethylphenyl)thio)benzonitrile (0.371 g, 1.55 mmol) was added *m*CPBA (0.803 g, 4.65 mmol) in DCM (5 mL). The reaction was stirred at r.t. for 4 h. The reaction was partitioned between DCM and sat. NaHCO_3_ solution. The aqueous layer was extracted with DCM (3 x 10 mL) and the organic fraction was dried with Na_2_SO_4_ and evaporated to give 4-((2,4-dimethylphenyl)sulfonyl)benzonitrile **140** (0.348 g, 83%). mp 99-10 ^o^C. ^1^H NMR (CDCl_3_, 400 MHz) δ 8.10 (d, J = 8.1 Hz, 1H), 7.96-7.93 (m, 2H), 7.80-7.76 (m, 2H), 7.23 (d, J = 8.1 Hz, 1H), 7.08 (s, 1H), 2.39 (s, 3H), 2.38 (s, 3H). LRMS Found: [M-H]-= 270.1.

4-((2,4-Dimethylphenyl)sulfonyl)benzonitrile **140** (338 g, 1.25 mmol) was added NaOH (0.498 g, 12.5 mmol) in a mixture of EtOH:water (1:1 mixture, 4 mL). The reaction was refluxed for 1.5 h. The organic solvent was evaporated to give a crude mixture. Residue was dissolved in water, acidified to pH1 using 2M HCl (aq) solution. The resulting white precipitate was collected and washed with water to give 4-((2,4-dimethylphenyl)sulfonyl)benzoic acid **141** (0.342g, 95%). mp 206-208 ^o^C. ^1^H NMR ((CD_3_)_2_SO) 400 MHz) δ 13.5 (s, 1H), 8.13-8.09 (m, 2H), 8.02 (d, J = 8.1 Hz, 1H), 7.92 (d, J = 8.5 Hz, 2H), 7.34 (d, J = 8.1 Hz, 1H), 7.21 (s, 1H), 2.35 (s, 3H), 2.31 (s, 3H). LRMS Found: [M-H]^+^= 291.

The title compound was obtained from (*S*)-5-methyl-8-(4-methylpiperazin-1-yl)-1,2,3,4-tetrahydronaphthalen-2-amine **80** and 4-((2,4-dimethylphenyl)sulfonyl)benzoic acid **141** using the general procedure A to give **17** (80%) as a white foam. HPLC 98.8%. mp 126-129°C. ^1^H NMR (CDCl_3_, 400 MHz) δ 8.09 (d, J = 8.1 Hz, 1H), 7.90-7.81 (m, 4H), 7.20 (d, J = 8.1 Hz, 1H), 7.06-7.01 (m, 2H), 6.91 (d, J = 8.0 Hz, 1H), 6.14 (d, J = 7.8 Hz, 1H), 4.48-4.39 (m , 1H), 3.24 (dd, J = 16.4, 4.6 Hz, 1H), 2.92-2.83 (m, 4H), 2.78 (t, J = 6.6 Hz, 2H), 2.73-2.47 (m, 5H), 2.38 (s, 6H), 2.33 (s, 3H), 2.22-2.16 (m, 1H), 2.20 (s, 3H), 1.96-1.87 (m, 1H). HRMS calcd. for C_31_H_37_N_3_O_3_S: 531.2574, found 531.2556.

*4.1.15. N-[(2S)-5-Methyl-8-(4-methyl-1-piperazinyl)-1,2,3,4-tetrahydro-2-naphthalenyl]-5-phenyl-2-pyridinecarboxamide (****18****)*

The title compound was obtained from (*S*)-5-methyl-8-(4-methylpiperazin-1-yl)-1,2,3,4-tetrahydronaphthalen-2-amine **80** and 5-phenylpicolinic acid using the general procedure A to give **18** (67%) as a white foam. HPLC 98.1%. ^1^H NMR (CDCl_3_) δ 8.78 (dd, J = 2.2, 0.7 Hz, 1H), 8.30 (dd, J = 8.1, 0.7 Hz, 1H), 8.09 (d, J = 8.4 Hz, 1H), 8.04 (dd, J = 8.1, 2.3 Hz, 1H), 7.62-7.60 (m, 2H), 7.53-7.49 (m, 2H), 7.47-7.43 (m, 1H), 7.04 (d, J = 8.0 Hz, 1H), 6.92 (d, J = 8.0 Hz, 1H), 4.43-4.41 (m, 1H), 3.37 (dd, J = 16.5, 4.0 Hz, 1H), 3.00-2.94 (m, 2H), 2.86-2.81 (m, 4H), 2.70-2.50 (m, 5H), 2.35 (s, 3H), 2.29-2.25 (m, 1H), 2.21 (s, 3H), 1.93-1.82 (m, 1H). ^13^C NMR (CDCl_3_) δ 163.9, 149.9, 149.0, 146.7, 139.2, 137.3, 135.8, 135.3, 132.1, 130.2, 129.5, 128.9, 128.14, 127.5, 122.5, 117.4, 55.8, 52.2, 46.3, 45.5, 32.1, 29.3, 26.3, 19.6. HRMS calcd. for C_28_H_33_N_4_O 441.2639, found 441.2649.

*4.1.16. N-[(2S)-5-Methyl-8-(4-methyl-1-piperazinyl)-1,2,3,4-tetrahydro-2-naphthalenyl]-5-[4-(trifluoromethyl)phenyl]-2-pyridinecarboxamide (****19****)*

The title compound was obtained from (*S*)-5-methyl-8-(4-methylpiperazin-1-yl)-1,2,3,4-tetrahydronaphthalen-2-amine **80** and 5-(4-(trifluoromethyl)phenyl)picolinic acid using the general procedure A to give **19** (62%) as a white foam. HPLC 95.1%.^1^H NMR (CDCl_3_) δ 8.78 (dd, J = 2.2, 0.6 Hz, 1H), 8.34 (dd, J = 8.1, 0.7 Hz, 1H), 8.09 (br d, J = 8.0 Hz, 1H), 8.06 (dd, J = 8.2, 2.3 Hz, 1H), 7.78 (d, J = 8.3 Hz, 1H), 7.73 (d, J = 8.3 Hz, 1H), 7.05 (d, J = 8.0 Hz, 1H), 6.93 (d, J = 8.0 Hz. 1H), 4.43 (m, 1H), 3.38 (m, 1H), 3.00-2.50 (m, 10H), 2.35 (s, 3H), 2.30 (m, 2H). 2.22 (s, 3H), 1.90 (m, 1H). HRMS calcd. for C_29_H_32_F_3_N_4_O 509.2528, found 509.2509.

*4.1.17. N-[(2R)-5-Methyl-8-(4-methyl-1-piperazinyl)-1,2,3,4-tetrahydro-2-naphthalenyl]-5-[4-(trifluoro)phenyl]-2-pyridinecarboxamide (****19R****)*

The title compound was obtained from (*R*)-5-methyl-8-(4-methylpiperazin-1-yl)-1,2,3,4-tetrahydronaphthalen-2-amine **80R** and 5-(4-(trifluoromethyl)phenyl)picolinic acid using the general procedure A to give **19** (89%) as a white foam. HPLC 99.6%. ^1^H NMR (CDCl_3_) δ 8.78 (dd, J = 2.2, 0.6 Hz, 1H), 8.34 (dd, J = 8.1, 0.6 Hz, 1H), 8.05-8.13 (m, 2H), 7.78 (d, J = 8.4 Hz, 2H), 7.72 (d, J = 8.3 Hz, 2H), 7.04 (d, J = 8.0 Hz, 1H), 6.93 (d, J = 8.0 Hz, 1H), 4.39-4.47 (m, 1H), 3.36 (dd, J = 16.4, 4.1 Hz, 1H), 2.93-2.98 (m, 2H), 2.83-2.86 (m, 4H), 2.68 (dd, J = 16.6, 9.0 Hz, 1H), 2.56 (br, 4H), 2.34 (s, 3H), 2.25-2.28 (m, 1H), 2.22 (s, 3H), 1.88-1.94 (m, 1H). HRMS calcd. for C_29_H_31_F_3_N_4_O: 508.2450, found 508.2464.

*4.1.18. N-[(2S)-5-Methyl-8-(4-methyl-1-piperazinyl)-1,2,3,4-tetrahydro-2-naphthalenyl]-5-[4-(trifluoromethoxy)phenyl]-2-pyridinecarboxamide (****20****)*

The title compound was obtained from (*S*)-5-methyl-8-(4-methylpiperazin-1-yl)-1,2,3,4-tetrahydronaphthalen-2-amine **80** and 5-(4-(trifluoromethoxy)phenyl)picolinic acid using the general procedure A to give **20** (71%) as a white foam. HPLC 96.7%.^1^H NMR (CDCl_3_) 8.75 (dd, J = 2.2, 0.6 Hz, 1H), 8.31 (dd, J = 8.0, 0.7 Hz, 1H), 8.08 (br d, J = 8.0 Hz, 1H), 8.02 (dd, J = 8.1, 2.3 Hz, 1H), 7.64 (d*,* J = 8.7 Hz, 2H), 7.36 (d, J = 8.7 Hz, 2H), 7.05 (d, J = 8.0 Hz, 1H), 6.93 (d, J = 8.0 Hz. 1H), 4.43 (m, 1H), 3.38 (m, 1H), 3.00-2.50 (m, 10H), 2.34 (s, 3H), 2.30 (m, 2H). 2.22 (s, 3H), 1.90 (m, 1H). ^13^C NMR (CDCl_3_) δ 163.7, 149.9, 149.4, 146.6, 137.9, 135.9, 135.8, 135.3, 132.1, 130.1, 128.9, 128.1, 122.6, 121.9, 117.4, 55.8, 52.2, 46.3, 45.5, 32.1, 29.9, 26.2, 19.6. HRMS calcd. for C_29_H_32_F_3_N_4_O_2_ 525.2472, found 525.2457.

*4.1.19. N-[(2R)-5-Methyl-8-(4-methyl-1-piperazinyl)-1,2,3,4-tetrahydro-2-naphthalenyl]-5-[4-(trifluoromethoxy)phenyl]-2-pyridinecarboxamide (****20R****)*

The title compound was obtained from (*R*)-5-methyl-8-(4-methylpiperazin-1-yl)-1,2,3,4-tetrahydronaphthalen-2-amine **80R** and 5-(4-(trifluoromethoxy)phenyl)picolinic acid using the general procedure A to give **20R** (59%) as a white foam. HPLC 92.6%. ^1^H NMR (CDCl_3_) δ 8.75 (dd, J = 2.3, 0.8 Hz, 1H), 8.31 (dd, J = 8.1, 0.8 Hz, 1H), 8.08 (d, J = 8.4 Hz, 1H), 8.02 (dd, J = 8.1, 2.3 Hz, 1H), 7.64 (ap d, J = 8.8 Hz, 2H), 7.36 (dd, J = 8.8, 0.8 Hz, 2H), 7.04 (d, J = 8.0 Hz, 1H), 6.93 (d, J = 8.0 Hz, 1H), 4.38-4.46 (m, 1H), 3.38 (dd, J = 16.5, 4.1 Hz, 1H), 2.93-2.99 (m, 2H), 2.76-2.88 (m, 4H), 2.68 (dd, J = 16.5, 9.1 Hz, 1H), 2.56 (br, 4H), 2.34 (s, 3H), 2.24-2.29 (m, 1H), 2.22 (s, 3H), 1.84-1.95 (m, 1H). HRMS calcd. for C_29_H_31_F_3_N_4_O_2_: 524.2399, found 524.2412.

*4.1.20. N-[(2S)-5-Methyl-8-(4-methyl-1-piperazinyl)-1,2,3,4-tetrahydro-2-naphthalenyl]-5-[4-(difluoromethoxy)phenyl]-2-pyridinecarboxamide (****21****)*

A mixture of methyl 5-bromopicolinate (1.03 g, 4.79 mmol), (4-(difluoromethoxy)phenyl)boronic acid (0.902 g, 4.80 mmol) and Cs_2_CO_3_ (3.10 g, 9.51 mmol) in anhydrous DMF (24 mL) was purged with nitrogen. Pd(PPh_3_)_4_ (0.110 g, 0.095 mmol) was added, the mixture was purged with nitrogen and then heated to 80 °C for 3 h under nitrogen in a sealable tube. The mixture was partitioned between EtOAc and water, workup and chromatography on silica using a gradient of 10-50% EtOAc:hexanes gave methyl 5-(4-(difluoromethoxy)phenyl)picolinate **142** (0.823 g, 62%) as a white solid. mp. 107-108 °C ^1^H NMR (CDCl_3_) δ 8.94 (dd, J = 2.3, 0.8 Hz, 1H), 8.22 (dd, J = 8.2, 0.8 Hz, 1H), 8.00 (dd, J = 8.2, 2.3 Hz, 1H), 7.63 (ap d, J = 8.8 Hz, 2H), 7.28 (ap d, J = 8.8 Hz, 2H), 6.58 (t, J = 73.3 Hz, 1H), 4.04 (s, 3H). LRMS Found: [M+H] = 280.1.

LiOH (0.223 g, 9.31 mmol) in water (15 mL) was added to a solution of **142** (0.868 g, 3.11 mmol) in THF (15 mL) and methanol (15 mL), the solution was stirred at room temperature for 18 h. The solvent was evaporated and the residue was diluted with water (30 mL), the acidified to pH 3 with 2M HCl. The precipitate was filtered, washed with water and extracted into EtOAc, the organic extract was dried and evaporated to give 5-(4-(difluoromethoxy)phenyl)picolinic acid **143** (0.580 g, 70%) as a white solid. mp.190-193 °C. ^1^H NMR ((CD_3_)_2_SO)) δ 8.91 (s, 1H), 8.18 (dd, J = 8.1, 1.8 Hz, 1H), 8.05 (d, J = 8.1 Hz, 1H), 7.85 (d, J = 8.6, 2H), 7.33 (t, J = 73.9 Hz, 1H), 7.33 (d, J = 8.6 Hz, 2H), OH exchanged. LRMS Found: [M+H]= 266.1.

The title compound was obtained from (*S*)-5-methyl-8-(4-methylpiperazin-1-yl)-1,2,3,4-tetrahydronaphthalen-2-amine **80** and 5-(4-(difluoromethoxy)phenyl)picolinic acid **143** using the general procedure A to give **21** (87%) as a white foam. HPLC 97.8%. ^1^H NMR (CDCl_3_) δ 8.74 (dd, J = 2.3 Hz, 0.8 Hz, 1H), 8.30 (dd, J = 8.1, 0.8 Hz, 1H), 8.08 (bd, J = 8.3 Hz, 1H), 8.01 (dd, J = 8.1, 2.3 Hz, 1H), 7.61 (ap d, J = 8.8 Hz, 2H), 7.27 (d, J = 8.8 Hz, 2H), 7.04 (d, J = 8.0 Hz, 1H), 6.93 (d, J = 8.0 Hz, 1H), 6.58 (t, J = 73.5 Hz, 1H), 4.38-4.45 (m, 2H), 3.38 (dd, J = 16.8, 4.2 Hz, 1H), 2.94-2.99 (m, 2H), 2.76-2.88 (m, 4H), 2.67 (dd, J = 16.6, 9.1 Hz, 1H), 2.57 (br, 4H), 2.34 (s, 3H), 2.19-2.30 (m, 1H), 2.22 (s, 3H), 1.86-1.95 (m, 1H). HRMS calcd. for C_29_H_32_F_2_N_4_O_2_: 506.2493, found 506.2499.

*4.1.21. N-[(2R)-5-Methyl-8-(4-methyl-1-piperazinyl)-1,2,3,4-tetrahydro-2-naphthalenyl]-5-[4-(difluoromethoxy)phenyl]-2-pyridinecarboxamide (****21R****)*

The title compound was obtained from (*R*)-5-methyl-8-(4-methylpiperazin-1-yl)-1,2,3,4-tetrahydronaphthalen-2-amine **80R** and 5-(4-(difluoromethoxy)phenyl)picolinic acid **143** using the general procedure A to give **21R** (56%) as a white foam. HPLC 99.1%. ^1^H NMR (CDCl_3_) δ ^1^H NMR (CDCl_3_) δ 8.74 (dd, J = 2.3 Hz, 0.8 Hz, 1H), 8.30 (dd, J = 8.1, 0.8 Hz, 1H), 8.08 (bd, J = 8.3 Hz, 1H), 8.01 (dd, J = 8.1, 2.3 Hz, 1H), 7.61 (ap d, J = 8.8 Hz, 2H), 7.27 (d, J = 8.8 Hz, 2H), 7.04 (d, J = 8.0 Hz, 1H), 6.93 (d, J = 8.0 Hz, 1H), 6.58 (t, J = 73.5 Hz, 1H), 4.38-4.45 (m, 2H), 3.38 (dd, J = 16.8, 4.2 Hz, 1H), 2.94-2.99 (m, 2H), 2.76-2.88 (m, 4H), 2.67 (dd, J = 16.6, 9.1 Hz, 1H), 2.57 (br, 4H), 2.34 (s, 3H), 2.19-2.30 (m, 1H), 2.22 (s, 3H), 1.86-1.95 (m, 1H). HRMS calcd. for C_29_H_32_ClF_2_N_4_O_2_: 506.2493, found 506.2496.

*4.1.22. (S)-5-(4-chloro-2-methylphenyl)-N-(5-methyl-8-(4-methylpiperazin-1-yl)-1,2,3,4-tetrahydronaphthalen-2-yl)picolinamide (****22****)*

The title compound was obtained from (*S*)-5-methyl-8-(4-methylpiperazin-1-yl)-1,2,3,4-tetrahydronaphthalen-2-amine **80** and 5-(4-chloro-2-methylphenyl)picolinic acid **83** using the general procedure A to give **22** (55%) as a white foam. HPLC 98.7%. ^1^H NMR (CDCl_3_) δ 8.49 (dd, J = 2.0, 0.8 Hz, 1H), 8.29 (dd, J = 8.4, 0.8 Hz, 1H), 8.09 (d, J = 8.4 Hz, 1H), 7.79 (dd, J = 8.0, 2.0 Hz, 1H), 7.27-7.32 (m, 2H), 7.15 (d, J = 8.4 Hz, 1H), 7.04 (d, J = 8.0 Hz, 1H), 6.93 (d, J = 8.0 Hz, 1H), 4.37-4.47 (m, 1H), 3.38 (dd, J = 16.4, 4.0 Hz, 1H), 2.94-2.99 (m, 2H), 2.76-2.86 (m, 4H), 2.67 (dd, J = 16.4, 8.8 Hz, 1H), 2.56 (br, 4H), 2.35 (s, 3H), 2.28 (br, 1H), 2.27 (s, 3H), 2.17 (s, 3H), 1.85-1.95 (m, 1H). ^13^C NMR (CDCl_3_) δ 163.7, 149.9, 149.1, 148.3, 139.1, 138.0, 137.7, 136.0, 135.3, 134.6, 132.1, 131.2, 130.8, 130.2, 128.1, 126.6, 122.1, 117.4, 55.8, 52.3, 46.3, 45.5, 32.1, 29.3, 26.2, 20.5, 19.6. HRMS calcd. for C_29_H_33_ClN_4_O: 488.2343, found 488.2352.

*4.1.23. (R)-5-(4-chloro-2-methylphenyl)-N-(5-methyl-8-(4-methylpiperazin-1-yl)-1,2,3,4-tetrahydronaphthalen-2-yl)picolinamide (****22R****)*

The title compound was obtained from (*R*)-5-methyl-8-(4-methylpiperazin-1-yl)-1,2,3,4-tetrahydronaphthalen-2-amine **80R** and 5-(4-chloro-2-methylphenyl)picolinic acid **83** using the general procedure A to give **22R** (32%) as a white foam. HPLC 99.6%. ^1^H NMR (CDCl_3_) δ ^1^H NMR (CDCl_3_) δ 8.49 (dd, J = 2.0, 0.4 Hz, 1H), 8.30 (dd, J = 8.4, 0.8 Hz, 1H), 8.09 (d, J = 8.4 Hz, 1H), 7.79 (dd, J = 8.0, 2.4 Hz, 1H), 7.27-7.32 (m, 2H), 7.15 (d, J = 8.4 Hz, 1H), 7.04 (d, J = 8.0 Hz, 1H), 6.93 (d, J = 8.0 Hz, 1H), 4.37-4.47 (m, 1H), 3.38 (dd, J = 16.4, 4.0 Hz, 1H), 2.94-2.99 (m, 2H), 2.76-2.86 (m, 4H), 2.67 (dd, J = 16.4, 8.8 Hz, 1H), 2.56 (br, 4H), 2.34 (s, 3H), 2.28 (br, 1H), 2.27 (s, 3H), 2.17 (s, 3H), 1.85-1.95 (m, 1H). ^13^C NMR (CDCl_3_) δ 163.7, 149.9, 149.1, 148.3, 139.1, 138.0, 137.7, 136.0, 135.3, 134.6, 132.1, 131.2, 130.8, 130.2, 128.1, 126.6, 122.1, 117.4, 55.8, 52.3, 46.3, 45.5, 32.1, 29.3, 26.2, 20.5, 19.6. HRMS calcd. for C_29_H_33_ClN_4_O: 488.2343, found 488.2358.

*4.1.24. (S)-5-(2-Methyl-4-(difluoromethoxy)phenyl)-N-(5-methyl-8-(4-methylpiperazin-1-yl)-1,2,3,4-tetrahydronaphthalen-2-yl)picolinamide (****23****)*

A mixture of *tert*-butyl 5-(4,4,5,5-tetramethyl-1,3,2-dioxaborolan-2-yl)picolinate (0.558 g, 1.83 mmol) and 1-bromo-2-methyl-4-(difluoromethoxy)benzene (0.520 g, 2.19 mmol) in toluene (25 mL), MeOH (10 mL) and aqueous sodium carbonate (2M, 5 mL, 10 mmol) was purged with nitrogen. PdCl_2_(dppf).DCM (0.075 g, 0.092 mmol) was added and the mixture was heated to reflux under nitrogen for 45 min. The mixture was partitioned between EtOAc and water, the organic fractions were dried and evaporated, chromatography on silica using hexanes:EtOAc (9:1) as an eluent gave *tert*-butyl 5-(2-methyl-4-(difluoromethoxy)phenyl)picolinate **146** (0.344 g, 47%) as a white solid. mp. 106-107 °C. ^1^H NMR (CDCl_3_) δ 8.69 (dd, J = 2.2, 0.8 Hz, 1H), 8.12 (dd, J = 8.0, 0.8 Hz, 1H), 7.74 (dd, J = 8.0, 2.2 Hz, 1H), 7.21 (d, J = 8.3 Hz, 1H), 7.04-7.09 (m, 2H), 6.56 (t, J = 73.7 Hz, 1H), 2.27 (s, 3H), 1.67 (s, 9H). LRMS Found: [M+H] = 336.2.

A solution of **146** (0.392 g, 1.17 mmol) and trifluoroacetic acid (1.75 mL, 23.6 mmol) in DCM (20 mL) was refluxed for 1 h. The solvent was evaporated and the residue was diluted with water (20 mL), the pH was adjusted to 3 with sat. aq. NaHCO_3_, and then extracted with EtOAc. The organic fractions were dried and evaporated to give 5-(2-methyl-4-(difluoromethoxy)phenyl)picolinic acid **147** (0.300 g, 97%) as a white solid.. 1H NMR ((CD_3_)_2_SO)) δ 7.94-8.13 (m, 3H), 7.28 (t, J = 74 Hz, 1H), 7.08-7.23 (m, 3H), 2.03 (s, 3H), OH exchanged. LRMS Found: [M+H] = 280.1.

The title compound was obtained from (*S*)-5-methyl-8-(4-methylpiperazin-1-yl)-1,2,3,4-tetrahydronaphthalen-2-amine **80** and 5-(2-methyl-4-(difluoromethoxy)phenyl)picolinic acid **147** using the general procedure A to give **23** (40%) as a white foam. HPLC 89.6%. ^1^H NMR (CDCl_3_) δ 8.50 (dd, J = 2.2, 0.7 Hz, 1H), 8.30 (dd, J = 8.0, 0.7 Hz, 1H), 8.09 (d, J = 8.4 Hz, 1H), 7.80 (dd, J = 8.0, 2.2 Hz, 1H), 7.21 (d, J = 8.2 Hz, 1H), 7.03-7.11 (m, 3H), 6.93 (d, J = 8.0 Hz, 1H), 6.56 (t, J = 73.7 Hz, 1H), 4.38-4.47 (m, 1H), 3.38 (dd, J = 16.4, 4.0 Hz, 1H), 2.93-2.98 (m, 2H), 2.76-2.90 (m, 4H), 2.67 (dd, J = 16.4, 9.2 Hz, 1H), 2.50 (br, 4H), 2.34 (s, 3H), 2.28 (s, 3H), 2.23-2.28 (m, 1H), 2.22 (s, 3H), 1.85-1.95 (m, 1H). HRMS calcd. for C_30_H_34_F_2_N_4_O_2_: 520.2650, found 520.2657.

*4.1.25. (R)-5-(2-Methyl-4-(difluoromethoxy)phenyl)-N-(5-methyl-8-(4-methylpiperazin-1-yl)-1,2,3,4-tetrahydronaphthalen-2-yl)picolinamide (****23R****)*

The title compound was obtained from (*R*)-5-methyl-8-(4-methylpiperazin-1-yl)-1,2,3,4-tetrahydronaphthalen-2-amine **80R** and 5-(2-methyl-4-(difluoromethoxy)phenyl)picolinic acid **147** using the general procedure A to give **23R** (45%) as a white foam. HPLC 96.8%.  ^1^H NMR (CDCl_3_) δ 8.50 (dd, J = 2.2, 0.7 Hz, 1H), 8.29 (dd, J = 8.0, 0.8 Hz, 1H), 8.09 (d, J = 8.4 Hz, 1H), 7.80 (dd, J = 8.0, 2.2 Hz, 1H), 7.21 (d, J = 8.3 Hz, 1H), 7.03-7.11 (m, 3H), 6.93 (d, J = 8.0 Hz, 1H), 6.56 (t, J = 73.7 Hz, 1H), 4.38-4.47 (m, 1H), 3.38 (dd, J = 16.4, 4.0 Hz, 1H), 2.96-3.00 (m, 2H), 2.76-2.88 (m, 4H), 2.67 (dd, J = 16.4, 9.2 Hz, 1H), 2.50 (br, 4H), 2.34 (s, 3H), 2.28 (s, 3H), 2.23-2.28 (m, 1H), 2.22 (s, 3H), 1.85-1.95 (m, 1H). HRMS calcd. for C_30_H_34_F_2_N_4_O_2_: 520.2650, found 520.2668

*4.1.26. (S)-5-(2-Methyl-4-(trifluoromethoxy)phenyl)-N-(5-methyl-8-(4-methylpiperazin-1-yl)-1,2,3,4-tetrahydronaphthalen-2-yl)picolinamide (****24****)*

A mixture of *tert*-butyl 5-(4,4,5,5-tetramethyl-1,3,2-dioxaborolan-2-yl)picolinate (2.00 g, 6.55 mmol) and 1-bromo-2-methyl-4-(trifluoromethoxy)benzene (2.16 g, 8.47 mmol) in toluene (25 mL), MeOH (10 mL) and aqueous sodium carbonate (2M, 5 mL, 10 mmol) was purged with nitrogen. PdCl_2_(dppf).DCM (0.320 g, 0.392 mmol) was added and the mixture was heated to reflux under nitrogen for 1 h. The mixture was partitioned between EtOAc and water, the organic fractions were dried and evaporated, chromatography on silica using 9:1 hexanes:EtOAc as an eluent gave *tert*-butyl 5-(2-methyl-4-(trifluoromethoxy)phenyl)picolinate **148** (0.660 g, 29%) as a white solid. mp 106-107 °C. ^1^H NMR (CDCl_3_) δ 8.70 (dd, J = 2.3, 0.8 Hz, 1H), 8.13 (dd, J = 8.1, 0.8 Hz, 1H), 7.75 (dd, J = 8.1, 2.3 Hz, 1H), 7.23 (d, J = 8.2 Hz, 1H), 7.13-7.19 (m, 2H), 2.28 (s, 3H), 1.67 (s, 9H). LRMS Found: [M+H] = 354.1.

A solution of **148** (0.564 g, 1.60 mmol) and trifluoroacetic acid (2.4 mL, 32.3 mmol) in DCM (25 mL) was refluxed for 3 h. The solvent was evaporated and the residue was diluted with water (30 mL), the pH was adjusted to 3 with sat. aq. NaHCO_3_, and then extracted with EtOAc. The organic fractions were dried and evaporated to give 5-(2-methyl-4-(trifluoromethoxy)phenyl)picolinic acid **149** (0.422 g, 89%) as a white solid. ^1^H NMR ((CD_3_)_2_SO)) δ 8.72 (dd, J =2.2, 0.7 Hz, 1H), 8.12 (dd, J = 8.0, 0.7 Hz, 1H), 8.02 (dd, J = 8.0, 2.2 Hz, 1H), 7.44 (d, J = 8.4 Hz, 1H), 7.41 (s, 1H), 7.33 (d, J = 8.4 Hz, 1H), 2.29 (s, 3H), OH exchanged. LRMS Found: [M+H] = 298.1

The title compound was obtained from (*S*)-5-methyl-8-(4-methylpiperazin-1-yl)-1,2,3,4-tetrahydronaphthalen-2-amine **80** and 5-(2-methyl-4-(trifluoromethoxy)phenyl)picolinic acid **149** using the general procedure A to give **24** (75%) as a white foam. HPLC 97.2%. ^1^H NMR (CDCl_3_) δ 8.50 (dd, J = 2.1, 0.7 Hz, 1H), 8.31 (dd, J = 8.0, 0.7 Hz, 1H), 8.09 (d, J = 8.4 Hz, 1H), 7.80 (dd, J = 8.0, 2.2 Hz, 1H), 7.24 (d, J = 8.3 Hz, 1H), 7.13-7.19 (m, 2H), 7.04 (d, J =8.0 Hz, 1H), 6.93 (d, J = 8.0 Hz, 1H), 4.39-4.46 (m, 1H), 3.38 (dd, J = 16.6, 4.1 Hz, 1H), 2.93-2.98 (m, 2H), 2.77-2.89 (m, 4H), 2.68 (dd, J = 16.5, 9.2 Hz, 1H), 2.56 (br, 4H), 2.34 (s, 3H), 2.29 (s, 3H), 2.23-2.28 (m, 1H), 2.22 (s, 3H), 1.86-1.95 (m, 1H). HRMS calcd. for C_30_H_33_F_3_N_4_O_2_: 538.2556, found 538.2567.

*4.1.27. (R)-5-(2-Methyl-4-(trifluoromethoxy)phenyl)-N-(5-methyl-8-(4-methylpiperazin-1-yl)-1,2,3,4-tetrahydronaphthalen-2-yl)picolinamide (****24R****)*

The title compound was obtained from (*R*)-5-methyl-8-(4-methylpiperazin-1-yl)-1,2,3,4-tetrahydronaphthalen-2-amine **80R** and 5-(2-methyl-4-(trifluoromethoxy)phenyl)picolinic acid **149** using the general procedure A to give **24R** (66%) as a white foam. HPLC 96.5%.  ^1^H NMR (CDCl_3_) δ 8.50 (dd, J = 2.1, 0.6 Hz, 1H), 8.30 (dd, J = 8.0, 0.5 Hz, 1H), 8.09 (d, J = 8.4 Hz, 1H), 7.80 (dd, J = 8.0, 2.2 Hz, 1H), 7.24 (d, J = 8.3 Hz, 1H), 7.13-7.19 (m, 2H), 7.04 (d, J =8.0 Hz, 1H), 6.93 (d, J = 8.0 Hz, 1H), 4.39-4.48 (m, 1H), 3.38 (dd, J = 16.6, 4.0 Hz, 1H), 2.93-3.00 (m, 2H), 2.77-2.89 (m, 4H), 2.67 (dd, J = 16.5, 9.2 Hz, 1H), 2.56 (br, 4H), 2.34 (s, 3H), 2.29 (s, 3H), 2.23-2.28 (m, 1H), 2.22 (s, 3H), 1.86-1.95 (m, 1H). HRMS calcd. for C_30_H_33_F_3_N_4_O_2_: 538.2556, found 538.2566.

*4.1.28. 5-(2,4-Dichlorophenyl)-N-[(2S)-5-methyl-8-(4-methyl-1-piperazinyl)-1,2,3,4-tetrahydro-2-naphthalenyl]-2-pyridinecarboxamide (****25****)*

The title compound was obtained from (*S*)-5-methyl-8-(4-methylpiperazin-1-yl)-1,2,3,4-tetrahydronaphthalen-2-amine **80** and 5-(2,4-dichlorophenyl)picolinic acid using the general procedure A to give **25** (59%) as an oil. HPLC 95.9%. ^1^H NMR (CDCl_3_) δ 8.60, (dd, J = 2.2, 0.6 Hz, 1H), 8.31 (dd, J = 8.0, 0.8 Hz, 1H), 8.09 (br d, J = 8.0 Hz, 1H), 7.92 (dd. J = 8.0, 2.2 Hz, 1H), 7.56 (d, J = 2.0 Hz, 1H), 7.38 (dd, J = 8.2, 2.1 Hz, 1H), 7.29 (d, J = 8.3 Hz, 1H), 7.05 (d, J *=* 8.0 Hz, 1H), 6.93 (d, J = 8.0 Hz, 1H), 4.42 (m, 1H), 3.38 (m, 1H), 3.00-2.50 (m, 10H), 2.35 (s, 3H), 2.28 (m, 2H). 2.22 (s, 3H), 1.90 (m, 1H). HRMS calcd. for C_28_H_31_Cl_2_N_4_O 509.1869, found 509.1875.

*4.1.29. 5-[4-Chloro-3-(trifluoromethyl)phenyl]-N-[(2S)-5-methyl-8-(4-methyl-1-piperazinyl)-1,2,3,4-tetrahydro-2-naphthalenyl]-2-pyridinecarboxamide (****26****)*

The title compound was obtained from (*S*)-5-methyl-8-(4-methylpiperazin-1-yl)-1,2,3,4-tetrahydronaphthalen-2-amine **80** and 5-(4-chloro-3-(trifluoromethyl)phenyl)picolinic acid using the general procedure A to give **26** (88%) as a white foam. HPLC 95.5 %. ^1^H NMR (CDCl_3_) δ 8.76, (dd, J = 2.2, 0.6 Hz, 1H), 8.34 (dd, *J* = 8.0, 0.8 Hz, 1H), 8.08 (br d, J = 8.0 Hz, 1H), 8.04 (dd, J = 8.1, 2.3 Hz, 1H), 7.92 (d. *J* = 2.1 Hz, 1H), 7.72 (dd, J = 8.3, 2.0 Hz, 1H), 7.66 (d, J = 8.8.3 Hz, 1H), 7.05 (d, J *=* 8.0 Hz, 1H), 6.93 (d, J = 8.0 Hz, 1H), 4.42 (m, 1H), 3.38 (m, 1H), 3.00-2.50 (m, 10H), 2.35 (s, 3H), 2.28 (m, 2H). 2.22 (s, 3H), 1.90 (m, 1H). HRMS calcd. for C_29_H_31_ClF_3_N_4_O 543.2133, found 543.2124.

*4.1.30. 5-[3,5-Bis(trifluoromethyl)phenyl]-N-[(2S)-5-methyl-8-(4-methyl-1-piperazinyl)-1,2,3,4-tetrahydro-2-naphthalenyl]-2-pyridinecarboxamide (****27****)*

The title compound was obtained from (*S*)-5-methyl-8-(4-methylpiperazin-1-yl)-1,2,3,4-tetrahydronaphthalen-2-amine **80** and 5-(3,5-bis(trifluoromethyl)phenyl)picolinic acid using the general procedure A to give **27** (52%) as a white foam. HPLC 97.9 %. ^1^H NMR (CDCl_3_) δ 8.79, (dd, J = 2.2, 0.6 Hz, 1H), 8.37 (dd, J = 8.0, 0.6 Hz, 1H), 8.04 (br s, 2H), 7.97 (br s, 1H), 7.05 (d, J *=* 8.0 Hz, 1H), 6.93 (d, J = 8.0 Hz. 1H), 4.42 (m, 1H), 3.38 (m, 1H), 3.00-2.50 (m, 10H), 2.35 (s, 3H), 2.28 (m, 2H), 2.22 (s, 3H), 1.90 (m, 1H). ^13^C NMR (CDCl_3_) δ 163.3, 150.5, 149.8, 146.7, 139.5, 136.3, 136.2, 135.2, 133.5, 133.2, 132.8, 132.5, 132.2, 130.0, 128.2, 128.6, 127.3, 124.6, 122.8, 122.6, 122.6, 121.9, 119.2, 117.5, 55.7, 52.0, 46.0, 45.6, 32.0, 29.2, 26.1, 19.6. HRMS calcd. for C_30_H_31_F_6_N_4_O 577.2399, found 577.2399.

*4.1.31. 6-(4-Chloro-2-methylphenyl)-N-[(2S)-5-methyl-8-(4-methyl-1-piperazinyl)-1,2,3,4-tetrahydro-2-naphthalenyl]nicotinamide (****28****)*

The title compound was obtained from (*S*)-5-methyl-8-(4-methylpiperazin-1-yl)-1,2,3,4-tetrahydronaphthalen-2-amine **80** and 6-(4-chloro-2-methylphenyl)nicotinic acid using the general procedure A to give **28** (83%) as a white foam. HPLC 97.4 %: mp 153-156 ^o^C. ^1^H NMR (CDCl_3_) δ 8.99, (dd, J = 2.2, 0.6 Hz, 1H), 8.08 (dd, J = 8.0, 0.8 Hz, 1H), 7.47 (dd, J = 8.1, 0.7 Hz, 1H), 7.37-7.27 (m. 3H), 7.05 (d, J *=* 8.0 Hz, 1H), 6.93 (d, J = 8.0 Hz. 1H), 6.15 (br d, J = 7.9 Hz, 1H), 4.51 (m, 1H), 3.28 (m, 1H), 2.92-2.50 (m, 10H), 2.35 (s, 3H), 2.28 (m, 2H), 2.36 (s, 3H), 2.35 (s, 3H), 2.32 (m, 1H), 2.21 (s, 3H), 1.90 (m, 1H). ^13^C NMR (CDCl_3_) δ 165.3, 161.7, 150.0, 147.6, 138.1, 135.8, 135.1, 134.8, 132.1, 131.1, 131.0, 129.3, 128.8, 128.4, 126.4, 124.0, 117.5, 55.8, 52.2, 46.3, 45.8, 31.8, 28.7, 25.5, 20.5, 19.6. HRMS calcd. for C_29_H_34_ClN_4_O 489.2416, found 489.2408.

*4.1.32. 6-(4-Chloro-2-methylphenyl)-N-[(2S)-5-methyl-8-(4-methyl-1-piperazinyl)-1,2,3,4-tetrahydro-2-naphthalenyl]nicotinamide (****28R****)*

The title compound was obtained from (*R*)-5-methyl-8-(4-methylpiperazin-1-yl)-1,2,3,4-tetrahydronaphthalen-2-amine **80** and 6-(4-chloro-2-methylphenyl)nicotinic acid using the general procedure A to give **28R** (72%) as a white foam. HPLC 96.4%. ^1^H NMR (CDCl_3_) δ 8.99 (dd, J = 2.3, 0.7 Hz, 1H), 8.17 (dd, J = 8.1, 2.3 Hz, 1H), 7.46 (dd, J = 8.1, 0.8 Hz, 1H), 7.34 (d, J = 8.1 Hz, 1H), 7.25-7.31 (m, 2H), 7.05 (d, J = 8.0 Hz, 1H), 6.93 (d, J = 8.0 Hz, 1H), 6.16 (d, J = 7.8 Hz, 1H), 4.47-4.55 (m, 1H), 3.28 (dd, J = 16.4, 4.6 Hz, 1H), 2.87-2.96 (m, 4H), 2.81 (t, J = 6.6 Hz, 2H), 2.76 (dd, J = 16.6, 7.9 Hz, 1H), 2.58 (br, 4H), 2.36 (s, 3H), 2.35 (s, 3H), 2.19-2.27 (br, 1H), 2.22 (s, 3H), 1.93-2.04 (m, 1H). HRMS calcd. for C_30_H_33_F_3_N_4_O_2_: 488.2343, found 488.2348.

*4.1.33. 5-(4-Chloro-2-methylphenyl)-N-[(2S)-5-methyl-8-(4-methyl-1-piperazinyl)-1,2,3,4-tetrahydro-2-naphthalenyl]nicotinamide (****29****)*

The title compound was obtained from (*S*)-5-methyl-8-(4-methylpiperazin-1-yl)-1,2,3,4-tetrahydronaphthalen-2-amine **80** and 5-(4-chloro-2-methylphenyl)nicotinic acid using the general procedure A to give **29** (69%) as a white foam. HPLC 95.3 %. ^1^H NMR (CDCl_3_) δ 8.89 (d, J = 2.1 Hz, 1H), 8.65 (d, J = 2.2 Hz, 1H), 8.05 (t, J = 2.2 Hz, 1H), 7.30 (m, 1H), 7.17 (d, *J =* 8.1 Hz, 1H), 7.05 (d, J = 8.0 Hz, 1H), 6.93 (d, J = 8.0 Hz, 1H), 6.17 (br d, J = 7.7 Hz, 1H), 4.49 (m, 1H), 3.28 (m, 1H), 2.94-2.50 (m, 10H), 2.34 (s, 3H), 2.24 (s, 3H), 2.23 (m, 1H), 2.21 (s, 3H), 1.95 (m, 1H). ^13^C NMR (CDCl_3_) δ 165.2, 152.3, 150.0, 146.4, 137.7, 136.7, 135.8, 135.8, 135.0, 134.6, 132.1, 131.3, 130.8, 130.4, 129.3, 128.4, 126.6, 117.5, 55.9, 52.3, 46.4, 46.0, 31.7, 28.7, 25.5, 20.5, 19.6. HRMS calcd. for C_29_H_34_ClN_4_O 489.2416, found 489.2413.

*4.1.34. 5-(4-Chloro-2-methylphenyl)-N-[(2R)-5-methyl-8-(4-methyl-1-piperazinyl)-1,2,3,4-tetrahydro-2-naphthalenyl]nicotinamide (****29R****)*

The title compound was obtained from (*R*)-5-methyl-8-(4-methylpiperazin-1-yl)-1,2,3,4-tetrahydronaphthalen-2-amine **80** and 5-(4-chloro-2-methylphenyl)nicotinic acid using the general procedure A to give **29R** (90%) as a white foam. HPLC 93.7%. ^1^H NMR (CDCl_3_) δ 8.89 (d, J = 2.2 Hz, 1H), 8.66 (d, J = 2.2 Hz, 1H), 8.06 (t, J = 2.2 Hz, 1H), 7.30 (m, 1H), 7.15 (d, J = 8.1 Hz, 1H), 7.05 (d, J = 8.0 Hz, 1H), 6.93 (d, J = 8.0 Hz, 1H), 6.16 (d, J = 7.8 Hz, 1H), 4.46-4.52 (m, 1H), 3.27 (dd, J = 16.3, 4.7 Hz, 1H), 2.85-2.94 (m, 4H), 2.81 (t, J = 6.7 Hz, 2H), 2.74 (dd, J = 16.5, 8.1 Hz, 1H), 2.56 (br, 4H), 2.35 (s, 3H), 2.25 (s, 3H), 2.21 (s, 3H), 2.20-2.26 (br, 1H), 1.91-2.05 (m, 1H). HRMS calcd. for C_29_H_34_ClN_4_O 489.2416, found 489.2418.

*4.1.35. (S)-2-(4-Chloro-2-methylphenyl)-N-(5-methyl-8-(4-methylpiperazin-1-yl)-1,2,3,4-tetrahydronaphthalen-2-yl)pyrimidine-5-carboxamide (****30****)*

A mixture of 5-bromo-2-iodopyrimidine (1.50 g, 5.27 mmol), (4-chloro-2-methylphenyl)boronic acid (0.980 g, 5.75 mmol) and Cs_2_CO_3_ (3.42 g, 10.4 mmol) in toluene (120 mL) and water (15 mL) was purged with nitrogen. Pd(PPh_3_)_4_ (0.060 g, 0.052 mmol) was added and the mixture was refluxed under nitrogen for 3 h. Workup and chromatography on silica gave 5-bromo-2-(4-chloro-2-methylphenyl)pyrimidine **150** (0.716 g, 44%) as a white solid. mp 104-105 °C. ^1^H NMR (CDCl_3_) δ 8.87 (s, 2H), 7.83 (d, J = 8.2 Hz, 1H), 7.27-7.32 (m, 2H), 2.55 (s, 3H). LRMS Found: [M+H]= 283.0, 285.0, 287.0.

Triethylamine (0.60 mL, 4.3 mmol) was added to a solution of **150** (0.605 g, 2.13 mmol) in DMSO (20 mL) and MeOH (20 mL) in a Berghof pressure reactor, followed by the addition of Pd(OAc)_2_ (0.048 g, 0.21 mmol) and DPPP (0.088 g, 0.21 mmol). The reactor was evacuated and then flushed twice with carbon monoxide, then pressurised with carbon monoxide to 80 psi and heated to 80 °C for 18 h. The mixture was partitioned between EtOAc and water, the organic extracts were dried and evaporated. Chromatography on silica using 4:1 hexanes:EtOAc gave methyl 2-(4-chloro-2-methylphenyl)pyrimidine-5-carboxylate **151** (0.520 g, 93%) as a white crystalline solid. mp. 108-109 °C. ^1^H NMR (CDCl_3_) δ 9.34 (s, 2H), 7.95 (dd, J = 7.7, 1.2 Hz, 1H), 7.29-7.32 (m, 2H), 4.01 (s, 3H), 2.60 (s, 3H). LRMS Found: [M+H]= 263.2, 265.1.

LiOH (0.120 g, 5.01 mmol) in water (10 mL) was added to a solution of **151** (0.441 g, 1.68 mmol) in THF (20 mL) and MeOH (20 mL), then stirred at room temperature for 18 h. The solvent was evaporated and the residue was diluted with water (80 mL), 2M HCl was added until pH 2, the resulting white precipitate was filtered and dried to give 2-(4-chloro-2-methylphenyl)pyrimidine-5-carboxylic acid **152** (0.230 g, 55%) as a white solid. mp >230 °C. ^1^H NMR ((CD_3_)_2_SO) δ 13.84 (bs, 1H), 9.31 (s, 2H), 7.93 (d, J = 8.4 Hz, 1H), 7.48 (d, J = 2.0 Hz, 1H), 7.43 (dd, J = 8.4, 1.9 Hz, 1H), 2.55 (s, 3H). LRMS Found: [M+H]= 249.1, 251.1.

The title compound was obtained from (*S*)-5-methyl-8-(4-methylpiperazin-1-yl)-1,2,3,4-tetrahydronaphthalen-2-amine **80** and 2-(4-chloro-2-methylphenyl)pyrimidine-5-carboxylic acid **152** using the general procedure A to give **30** (54%) as a white foam. HPLC 97.8%. ^1^H NMR (CDCl_3_) δ 9.15 (s, 2H), 7.89 (dd, J = 7.7, 1.0 Hz, 1H), 7.28-7.32 (m, 2H), 7.06 (d, J = 8.0 Hz, 1H), 6.94 (d, J = 8.0 Hz, 1H), 6.14 (d, J =7.7 Hz, 1H), 4.48-4.57 (m, 1H), 3.27 (dd, J = 16.5, 4.6 Hz, 1H), 2.89 (t, J = 4.7 Hz, 4H), 2.78-2.83 (m, 4H), 2.58 (s, 3H), 2.49-2.65 (br, 3H), 2.35 (s, 3H), 2.22 (s, 3H), 2.18-2.26 (br, 1H), 1.95-2.05 (m, 1H). ^13^C NMR (CDCl_3_) δ 168.5, 163.5, 155.8, 150.0, 140.3, 136.3, 135.6, 135.0, 132.5, 132.1, 131.7, 129.1, 128.5, 126.4, 125.2, 117.6, 55.8, 52.3, 46.3, 46.0, 31.7, 28.6, 25.4, 21.7, 19.6. HRMS calcd. for C_28_H_32_ClN_5_O: 489.2295, found 489.2311.

*4.1.36. (R)-2-(4-Chloro-2-methylphenyl)-N-(5-methyl-8-(4-methylpiperazin-1-yl)-1,2,3,4-tetrahydronaphthalen-2-yl)pyrimidine-5-carboxamide (****30R****)*

The title compound was obtained from (*R*)-5-methyl-8-(4-methylpiperazin-1-yl)-1,2,3,4-tetrahydronaphthalen-2-amine **80R** and 2-(4-chloro-2-methylphenyl)pyrimidine-5-carboxylic acid **152** using the general procedure A to give **30R** (56%) as a white foam. HPLC 99.3%. ^1^H NMR (CDCl_3_) δ 9.15 (s, 2H), 7.89 (dd, J = 7.7, 1.0 Hz, 1H), 7.28-7.31 (m, 2H), 7.06 (d, J = 8.0 Hz, 1H), 6.94 (d, J = 8.0 Hz, 1H), 6.14 (d, J =7.7 Hz, 1H), 4.48-4.56 (m, 1H), 3.27 (dd, J = 16.5, 4.6 Hz, 1H), 2.90 (t, J = 4.7 Hz, 4H), 2.78-2.83 (m, 4H), 2.58 (s, 3H), 2.49-2.65 (br, 3H), 2.35 (s, 3H), 2.22 (s, 3H), 2.18-2.26 (br, 1H), 1.95-2.05 (m, 1H). HRMS calcd. for C_28_H_32_ClN_5_O: 489.2295, found 489.2303.

*4.1.37. (S)-5-(4-Chloro-2-methylphenyl)-N-(5-methyl-8-(4-methylpiperazin-1-yl)-1,2,3,4-tetrahydronaphthalen-2-yl)pyrazine-2-carboxamide (****31****)*

A mixture of (4-chloro-2-methylphenyl)boronic acid (3.57 g, 21.0 mmol) and *tert*-butyl 5-chloropyrazine-2-carboxylate (3.743 g, 17.4 mmol) in toluene (150 mL), MeOH (60 mL) and aqueous sodium carbonate (2M, 30 mL, 60 mmol) was purged with nitrogen. Pd(dppf)Cl_2_.DCM (0.71 g, 0.87 mmol) was added and the mixture was refluxed under nitrogen for 45 min. The mixture was partitioned between EtOAc and water and the organic fractions were dried and evaporated. Chromatography on silica using 9:1 hexanes:EtOAc gave *tert*-butyl 5-(4-chloro-2-methylphenyl)pyrazine-2-carboxylate **153** (5.22 g, 98%) as a colourless oil. ^1^H NMR (CDCl_3_) δ 9.27 (d, J = 1.5 Hz, 1H), 8.78 (J = 1.5 Hz, 1H), 7.39 (d, J = 8.1 Hz, 1H), 7.28-7.33 (m, 2H), 2.39 (s, 3H), 1.66 (s, 9H). LRMS Found: [M+H-C_4_H_8_] = 249.1, 251.1.

A solution of **153** (0.523 g, 1.72 mmol) and trifluoroacetic acid (2.55 mL, 34.3 mmol) in DCM (20 mL) was stirred at room temperature for 2 h, and then at reflux for 1 h. Evaporation of the solvent gave a yellow solid, which was triturated with water and then dried to give 5-(4-chloro-2-methylphenyl)pyrazine-2-carboxylic acid **154** (0.415 g, 97%) as a white solid. mp. 211-213 °C. ^1^H NMR ((CD_3_)_2_SO) δ 13.79 (bs, 1H), 9.25 (d, J = 1.1 Hz, 1H), 8.98 (s, 1H), 7.59 (d, J = 8.3 Hz, 1H), 7.51 (d, J = 1.9 Hz, 1H), 7.45 (dd, J = 8.2, 1.9 Hz, 1H), 2.39 (s, 3H). LRMS Found: [M+H]= 249.1, 251.1.

The title compound was obtained from (*S*)-5-methyl-8-(4-methylpiperazin-1-yl)-1,2,3,4-tetrahydronaphthalen-2-amine **80** and 5-(4-chloro-2-methylphenyl)pyrazine-2-carboxylic acid **154** using the general procedure A to give **31** (40%) as a white foam. HPLC 95.5%. ^1^H NMR (CDCl_3_) δ ^1^H NMR (CDCl_3_) δ 9.48 (d, J = 1.5 Hz, 1H), 8.61 (d, J = 1.4 Hz, 1H), 7.85 (d, J = 8.4 Hz, 1H), 7.41 (d, J = 8.2 Hz, 1H), 7.31-7.35 (m, 2H), 7.05 (d, J = 8.0 Hz, 1H), 6.93 (d, J = 8.0 Hz, 1H), 4.46 (m, 1H), 3.35 (dd, J =1 6.5, 4.4 Hz, 1H), 2.92-2.97 (m, 2H), 2.80-2.87 (m, 4H), 2.70 (dd, J = 16.6 Hz, 8.9 Hz, 1H), 2.56 (bs, 3H), 2.41 (s, 3H), 2.34 (s, 3H), 2.25-2.29 (m, 1H), 2.22 (s, 3H), 1.93 (m, 1H). ^13^C NMR (CDCl_3_) δ 162.7, 157.0, 150.0, 143.6, 142.5, 138.7, 135.9, 135.1, 134.8, 132.1, 131.5, 131.4, 129.8, 128.2, 126.8, 117.5, 55.9, 52.3, 46.3, 45.5, 31.9, 29.1, 26.0, 20.6, 19.6. HRMS calcd. for C_28_H_32_ClN_5_O: 489.2295, found 489.2300.

*4.1.38. (S)-5-(4-Chloro-2-methylphenyl)-N-(5-methyl-8-(4-methylpiperazin-1-yl)-1,2,3,4-tetrahydronaphthalen-2-yl)thiophene-2-carboxamide (****32****)*

A mixture of 5-bromothiophene-2-carboxylic acid (2.29 g, 11.1 mmol), (4-chloro-2-methylphenyl)boronic acid (1.98 g, 11.6 mmol) and Cs_2_CO_3_ (7.21 g, 22.1 mmol) in DMF/toluene (1:2, 50 mL) was purged with nitrogen. Pd(PPh_3_)_4_ (0.26 g, 0.23 mmol) was added and the mixture was heated to 80 °C under nitrogen for 18 h. The mixture was partitioned between EtOAc and water, the aqueous layer was acidified to pH 2 with 2M HCl, then extracted with EtOAc. The organic fractions were dried and then evaporated on to silica gel, chromatography on silica using EtOAc gave 5-(4-chloro-2-methylphenyl)thiophene-2-carboxylic acid **155** (1.777 g, 63%) as an off white solid. mp. 206-208 °C. ^1^H NMR ((CD_3_)_2_SO) δ 13.18 (bs, 1H), 7.73 (d, J = 3.9 Hz, 1H), 7.44-7.49 (m, 2H), 7.35 (dd, J = 8.3, 1.9 Hz, 1H), 7.29 (d, J = 3.8 Hz, 1H), 2.40 (s, 3H). LRMS Found: [M-H]= 251.1, 253.1.

The title compound was obtained from (*S*)-5-methyl-8-(4-methylpiperazin-1-yl)-1,2,3,4-tetrahydronaphthalen-2-amine **80** and 5-(4-chloro-2-methylphenyl)pyrazine-2-carboxylic acid **155** using the general procedure A to give **32** (77%) as a white foam. HPLC 97.4%. ^1^H NMR (CDCl_3_) δ 7.44 (d, J = 3.8 Hz, 1H), 7.31 (d, J = 8.2 Hz, 1H), 7.28 (d, J = 2.0 Hz, 1H), 7.21 (dd, J = 8.2, 2.2 Hz, 1H), 7.04 (d, J = 8.1 Hz, 1H), 6.99 (d, J = 3.8 Hz, 1H), 6.93 (d, J = 8.0 Hz, 1H), 5.96 (d, J = 7.9 Hz, 1H), 4.40-4.46 (m, 1H), 3.29 (dd, J = 16.4, 4.5 Hz, 1H), 2.89 (m, 4H), 2.80 (t, J = 6.5 Hz, 2H), 2.68 (dd, J = 16.4, 8.3 Hz, 1H), 2.58 (b, 4H), 2.40 (s, 3H), 2.36 (s, 3H), 2.19 (m, 1H), 2.18 (s, 3H), 1.91 (m, 1H). ^13^C NMR (CDCl_3_) δ 161.5, 150.0, 146.6, 139.1, 138.2, 135.2, 134.4, 132.1, 132.0, 131.7, 131.0, 129.6, 128.3, 128.1, 127.3, 126.4, 117.4, 55.9, 52.3, 46.4, 45.8, 31.9, 29.0, 25.7, 21.2, 19.6. HRMS calcd. for C_28_H_32_ClN_3_OS: 493.1955, found 493.1968.

*4.1.39. (R)-5-(4-Chloro-2-methylphenyl)-N-(5-methyl-8-(4-methylpiperazin-1-yl)-1,2,3,4-tetrahydronaphthalen-2-yl)thiophene-2-carboxamide (****32R****)*

The title compound was obtained from (*R*)-5-methyl-8-(4-methylpiperazin-1-yl)-1,2,3,4-tetrahydronaphthalen-2-amine **80R** and 5-(4-chloro-2-methylphenyl)pyrazine-2-carboxylic acid **155** using the general procedure A to give **32R** (42%) as a white foam. HPLC 98.3%. ^1^H NMR (CDCl_3_) δ 7.44 (d, J = 3.8 Hz, 1H), 7.31 (d, J = 8.2 Hz, 1H), 7.28 (d, J = 2.0 Hz, 1H), 7.21 (dd, J = 8.2, 2.2 Hz, 1H), 7.04 (d, J = 8.1 Hz, 1H), 6.99 (d, J = 3.8 Hz, 1H), 6.93 (d, J = 8.0 Hz, 1H), 5.96 (d, J = 7.9 Hz, 1H), 4.40-4.46 (m, 1H), 3.29 (dd, J = 16.4, 4.5 Hz, 1H), 2.89 (m, 4H), 2.80 (t, J = 6.5 Hz, 2H), 2.68 (dd, J = 16.4, 8.3 Hz, 1H), 2.58 (b, 4H), 2.40 (s, 3H), 2.36 (s, 3H), 2.19 (m, 1H), 2.18 (s, 3H), 1.91 (m, 1H). ^13^C NMR (CDCl_3_) δ 161.5, 150.0, 146.6, 139.1, 138.2, 135.2, 134.4, 132.1, 132.0, 131.7, 131.0, 129.6, 128.3, 128.1, 127.3, 126.4, 117.4, 55.9, 52.3, 46.4, 45.8, 31.9, 29.0, 25.7, 21.2, 19.6. HRMS calcd. for C_28_H_32_ClN_3_OS: 493.1955, found 493.1968.

*4.1.40. (S)-1-(4-Chloro-2-methylphenyl)-N-(5-methyl-8-(4-methylpiperazin-1-yl)-1,2,3,4-tetrahydronaphthalen-2-yl)-1H-pyrazole-3-carboxamide (****33****)*

NaNO_2_ (2.9 g, 42 mmol) in water (11 mL) was added to 4-chloro-2-methylaniline (5.42 g, 38.3 mmol) in HCl (35 mL, 12 M, 0.42 mol) at 0 °C, then stirred at 0 °C for 0.5 h. The solution was filtered into a solution of NaBF_4_ (9.5 g, 87 mmol) in water 11 mL. The solid was extracted into EtOAc, dried and rotary evaporation at 20 °C gave 4-chloro-2-methylbenzenediazonium tetrafluoroborate **156** (2.20 g, 24%) as an off-white solid. ^1^H NMR ((CD_3_)_2_SO) δ 8.67 (d, J = 8.9 Hz, 1H), 8.06 (m, 1H), 7.94 (ddd, J = 8.9, 2.2, 0.4 Hz, 1H), 2.73 (s, 3H).

**156** (2.151 g, 8.95 mmol) was added to a solution of ethyl 2-chloro-3-oxobutanoate 1.30 mL, 9.40 mmol) in pyridine (6 mL) and water (5 mL) at -5 °C, after stirring at -5 °C for 30 min the yellow precipitate was filtered and washed with water. Recrystallisation from EtOH/water gave ethyl 2-chloro-2-(2-(4-chloro-2-methylphenyl)hydrazineylidene)acetate **157** (1.502 g, 67%) as pale yellow needles.. mp. 107-108 °C. ^1^H NMR (CDCl_3_) δ 8.20 (s, 1H), 7.47 (d, J = 8.6 Hz, 1H), 7.20 (dd, J = 8.7, 2.4 Hz, 1H), 7.14 (d, J =2 .0 Hz, 1H), 4.40 (q, J = 7.1 Hz, 2H), 2.29 (s, 3H), 1.41 (t, J = 7.1 Hz, 3H). LRMS Found: [M-H] = 273.1, 275.1.

A solution of **157** (1.496 g, 5.44 mmol), Et_3_N (2.27 mL, 16.3 mmol) and norbornadiene (2.76 mL, 27.1 mmol) in toluene (30 mL) was heated to 70 °C for 2 h. The mixture was filtered and the solvent evaporated to give crude **158**, which was used directly. A solution of **158** in xylenes (50 mL) was refluxed for 2 h, solvent was periodically removed by distillation and replenished with new xylenes to remove cyclopentadiene. The solution was cooled and evaporated, chromatography on silica using a gradient of 10-20% EtOAc:hexanes gave ethyl 1-(4-chloro-2-methylphenyl)-1*H*-pyrazole-3-carboxylate **159** (1.153 g, 80%) as a pale yellow solid. mp. 109-110 °C. ^1^H NMR (CDCl_3_) δ 7.58 (d, J = 2.4 Hz, 1H), 7.25-7.33 (m, 3H), 6.99 (d, J = 2.4 Hz, 1H), 4.4 (q, J = 7.1 Hz, 2H), 2.20 (s, 3H), 1.41 (t, J = 7.1 Hz, 3H). LRMS Found: [M+H]= 265.1, 267.1.

A solution of LiOH (0.303 g, 12.7 mmol) and **159** (1.18 g, 4.22 mmol) in THF (20 mL), MeOH (20 mL) and water (20 mL) was stirred at room temperature for 16 h. The organic solvent was removed and the residue was diluted with water (50 mL), acidification to pH 2 with conc. HCl gave a white precipitate which was filtered, washed with water and dried to give 1-(4-chloro-2-methylphenyl)-1*H*-pyrazole-3-carboxylic acid **160** (0.901 g, 90%) as a white solid. mp. 197-199 °C. ^1^H NMR ((CD_3_)_2_SO) δ 12.90 (bs, 1H), 8.16 (d, J = 2.4 Hz, 1H), 7.56 (s, 1H), 7.44 (m, 2H), 6.92 (d, J = 2.4 Hz, 1H), 2.19 (s, 3H). LRMS Found: [M+H] = 237.1, 239.1.

The title compound was obtained from (*S*)-5-methyl-8-(4-methylpiperazin-1-yl)-1,2,3,4-tetrahydronaphthalen-2-amine **80** and 1-(4-chloro-2-methylphenyl)-1*H*-pyrazole-3-carboxylic acid **160** using the general procedure A to give **33** (98%) as a white foam. HPLC 92.7%. ^1^H NMR (CDCl_3_) δ 7.58 (d, J = 2.4 Hz, 1H), 7.35 (bd, J = 0.6 Hz, 1H), 7.30 (d, J = 1.4 Hz, 1H), 7.02 (d, J = 2.4 Hz, 1H), 7.01 (d, J = 7.2 Hz, 1H), 6.88-6.95 (m, 2H), 4.32-4.42 (m, 1H), 3.35 (dd, J = 16.5, 3.8 Hz, 1H), 2.90-2.97 (m, 2H), 2.73-2.86 (m, 4H), 2.57 (dd, J = 16.5, 9.4 Hz, 1H), 2.54 (br, 4H), 2.33 (s, 3H), 2.22-2.28 (m, 1H), 2.22 (s, 3H), 2.17 (s, 3H), 1.77-1.87 (m, 1H). HRMS calcd. for C_27_H_32_ClN_5_O: 477.2295, found 477.2308.

*4.1.41. (S)-1-(4-Chloro-2-methylphenyl)-N-(5-methyl-8-(4-methylpiperazin-1-yl)-1,2,3,4-tetrahydronaphthalen-2-yl)-1H-1,2,3-triazole-4-carboxamide (****34****)*

Cu(OAc)_2_.H_2_O (0.119 g, 0.596 mmol) and CuI (0.11 g, 0.58 mmol) were added to a solution of 1-azido-4-chloro-2-methylbenzene (2.00 g, 11.9 mmol) and *tert*-butyl propiolate (1.15 g, 12.0 mmol) in THF (40 mL), the mixture was heated to 40 °C in a sealable tube for 16 h. The mixture was partitioned between Et_2_O and water. the organic fractions were dried and evaporated. Silica chromatography (DCM) gave *tert*-butyl 1-(4-chloro-2-methylphenyl)-1*H*-1,2,3-triazole-4-carboxylate **161** (2.543 g, 73%) as white solid. m.p. 117-119 °C. ^1^H NMR (CDCl_3_) δ 8.17 (s, 1H), 7.40 (d, J = 2.2 Hz, 1H), 7.34 (ddd, J = 8.5, 2.2, 0.4 Hz, 1H), 7.28 (d, J = 8.5 Hz, 1H), 2.21 (s, 3H), 1.65 (s, 9H). LRMS Found: [M+H]= 294.1, 296.1.

Trifluoroacetic acid (5.0 mL, 67 mmol) was added to a solution of **161** (2.00 g, 6.81 mmol) in DCM (30 mL), the solution was stirred at room temperature for 2 h, then at reflux for 1 h. The solvent was removed and the residue was triturated with water, the precipitate was filtered and washed with water to give 1-(4-chloro-2-methylphenyl)-1*H*-1,2,3-triazole-4-carboxylic acid **162** (1.404 g, 87%) as a white solid. mp. 142-144 °C. ^1^H NMR ((CD_3_)_2_SO) δ 13.29 (bs, 1H), 9.09 (s, 1H), 7.64 (dd, J = 1.7, 0.6 Hz, 1H), 7.55 (d, J = 8.4 Hz, 1H), 7.51 (dd, J = 8.5, 0.4 Hz, 1H), 2.16 (s, 3H). LRMS Found: [M+H]= 238.1, 240.1.

The title compound was obtained from (*S*)-5-methyl-8-(4-methylpiperazin-1-yl)-1,2,3,4-tetrahydronaphthalen-2-amine **80** and 1-(4-chloro-2-methylphenyl)-1*H*-1,2,3-triazole-4-carboxylic acid **162** using the general procedure A to give **34** (75%) as a white foam. HPLC 98.8%. ^1^H NMR (CDCl_3_) δ 8.26 (s, 1H), 7.41 (d, J = 2.0 Hz, 1H), 7.36 (dd, J = 8.4, 2.0 Hz, 1H), 7.28 (d, J = 8.4 Hz, 1H), 7.20 (d, J = 8.0 Hz, 1H), 6.92 (d, J = 8.0 Hz, 1H), 4.39-4.49 (m, 1H), 3.35 (dd, J = 16.4 Hz, 4.4 Hz, 1H), 2.92-2.99 (m, 2H), 2.77-2.89 (m, 4H), 2.70 (dd, J = 16.5, 8.8 Hz, 1H), 2.57 (br, 4H), 2.35 (s, 3H), 2.20-2.30 (m, 1H), 2.22 (s, 3H), 2.21 (s, 3H), 1.87-1.97 (m, 1H). HRMS calcd. for C_26_H_31_ClN_6_O: 478.2248, found 478.2257.

*4.1.42. (R)-1-(4-Chloro-2-methylphenyl)-N-(5-methyl-8-(4-methylpiperazin-1-yl)-1,2,3,4-tetrahydronaphthalen-2-yl)-1H-1,2,3-triazole-4-carboxamide (****34R****)*

The title compound was obtained from (*R*)-5-methyl-8-(4-methylpiperazin-1-yl)-1,2,3,4-tetrahydronaphthalen-2-amine **80R** and 1-(4-chloro-2-methylphenyl)-1*H*-1,2,3-triazole-4-carboxylic acid **162** using the general procedure A to give **34R** (76%) as a white foam. HPLC 99.6%. ^1^H NMR (CDCl_3_) δ 8.25 (s, 1H), 7.41 (d, J = 2.2 Hz, 1H), 7.36 (ddd, J = 8.4, 2.3, 0.5 Hz, 1H), 7.29 (d, J = 8.4 Hz, 1H), 7.04 (d, J = 8.0 Hz, 1H), 6.92 (d, J = 8.0 Hz, 1H), 4.39-4.49 (m, 1H), 3.35 (dd, J = 16.5, 4.5 Hz, 1H), 2.92-2.99 (m, 2H), 2.77-2.89 (m, 4H), 2.70 (dd, J = 16.6, 8.7 Hz, 1H), 2.58 (br, 4H), 2.35 (s, 3H), 2.20-2.30 (m, 1H), 2.22 (s, 3H), 2.21 (s, 3H), 1.87-1.97 (m, 1H). HRMS calcd. for C_26_H_31_ClN_6_O: 478.2248, found 478.2260.

*4.1.43. (S)-2-(4-Chloro-2-methylphenyl)-N-(5-methyl-8-(4-methylpiperazin-1-yl)-1,2,3,4-tetrahydronaphthalen-2-yl)-2H-tetrazole-5-carboxamide (****35****)*

Glyoxylic acid (1.30 g, 14.1 mmol) was added to a slurry of (4-chloro-2-methylphenyl)hydrazine hydrochloride (2.50 g, 12.9 mmol) in water (25 mL) and HCl (1.5 mL, 12 M, 18 mmol). The suspension was stirred at room temperature for 1 h, then filtered and dried to give (*E*)-2-(2-(4-chloro-2-methylphenyl)hydrazineylidene)acetic acid **163** (2.642 g, 92%) as a pale yellow solid. ^1^H NMR ((CD_3_)_2_SO) δ 12.35 (s, 1H), 10.45 (s, 1H), 7.43 (d, J = 1.0 Hz, 1H), 7.40 (d, J = 9.4 Hz, 1H), 7.16-7.21 (m, 2H), 2.22 (s, 3H). LRMS Found: [M-CO_2_]= 168.2, 170.0.

Sodium (0.324 g, 14.1 mmol) was reacted with EtOH (15 mL), **163** (1.51 g, 7.10 mmol) and 2-azido-1,3,5-tribromobenzene (2.64 g, 7.42 mmol) were added and the mixture was refluxed under nitrogen for 4 h. The solvent was evaporated, the residue was dissolved in water (40 mL) and then filtered. The filtrate was acidified to pH 2 with conc. HCl and the precipitate was extracted with EtOAc, the organic fractions were dried and evaporated. Chromatography on silica using a gradient (75-0% hexanes:EtOAc) gave 2-(4-chloro-2-methylphenyl)-2*H*-tetrazole-5-carboxylic acid **164** (1.043 g, 55%) as a white solid. mp. 171-173 °C. ^1^H NMR ((CD_3_)_2_SO) δ 7.76 (d, J = 8.6 Hz, 1H), 7.72 (J = 2.0 Hz, 1H), 7.59 (ddd, J = 8.6, 2.0, 0.5 Hz, 1H), 2.29 (s, 3H), OH exchanged. LRMS Found: [M-NCCO_2_H]= 165.1, 167.0.

The title compound was obtained from (*S*)-5-methyl-8-(4-methylpiperazin-1-yl)-1,2,3,4-tetrahydronaphthalen-2-amine **80** and 2-(4-chloro-2-methylphenyl)-2*H*-tetrazole-5-carboxylic acid **164** using the general procedure A to give **35** (51%) as a white foam. HPLC 93.6%. ^1^H NMR (CDCl_3_) δ 7.60 (d, J = 8.5 Hz, 1H), 7.43 (d, J = 2.0 Hz, 1H), 7.38 (dd, J = 8.5, 2.2 Hz, 1H), 7.22 (d, J = 8.0 Hz, 1H), 7.04 (d, J = 8.0 Hz, 1H), 6.93 (d, J = 8.0 Hz, 1H), 4.48-4.55 (m, 1H), 3.36 (dd, J = 16.4, 4.4 Hz, 1H), 2.91-2.96 (m, 2H), 2.80-2.88 (m, 4H), 2.74 (dd, J = 16.5, 8.5 Hz, 1H), 2.56 (br, 4H), 2.40 (s, 3H), 2.35 (s, 3H), 2.22-2.32 (m, 1H), 2.21 (s, 3H), 1.94-2.01 (m, 1H). HRMS calcd. for C_25_H_30_ClN_7_O: 479.2200, found 479.2210.

*4.1.44. General procedure B:* *(S)-1-(5-(4-Chloro-2-methylphenyl)pyridin-2-yl)-3-(5-methyl-8-(4-methylpiperazin-1-yl)-1,2,3,4-tetrahydronaphthalen-2-yl)urea (****36****)*

To a suspension of 5-bromopyridin-2-amine (1.00 g, 5.78 mmol) and pyridine (0.56 mL, 6.94 mmol) in DCM (10 mL) in an ice bath was added 4-nitrophenyl carbonochloridate (1.40 g, 6.94 mmol) portionwise. The mixture was stirred at room temperature overnight. The resulting precipitate was collected by filtration, washed with DCM, and dried under vacuum to give the product **165** as a white solid (1.97 g, 100%) which was used crude for the next step.

To a solution of **80** (0.260 g, 1.00 mmol) in MeCN (10 mL) and DCM (10 mL) at room temperature was added **165** (0.405 g, 1.20 mmol), followed by trimethylamine (0.70 mL, 5.00 mmol). The mixture was stirred overnight and distributed between water and ethyl acetate. The organic phase was washed with water and brine, dried over anhydrous Na_2_SO_4_. The solvent was removed to give the crude product, which was purified by Davisil® column chromatography, using gradient mixtures of MeOH and DCM (v/v=8~15%) as eluent to give the product **166** as a white solid (0.388 g, 85%): mp 185-187°C. ^1^H NMR (CDCl_3_, 400 MHz) δ 9.04 (br d, *J* = 6.5 Hz, 1H), 8.23 (br, 1H), 8.07 (d, *J* = 2.3 Hz, 1H), 7.64 (dd, *J* = 8.8, 2.4 Hz, 1H), 7.03 (d, *J* = 8.0 Hz, 1H), 6.90 (d, *J* = 8.0 Hz, 1H), 6.73 (d, *J* = 8.8 Hz, 1H), 4.20-4.30 (m, 1H), 3.25 (dd, *J* = 16.3, 4.2 Hz, 1H), 2.92-2.98 (m, 2H), 2.78-2.88 (m, 4H), 2.66-2.72 (m, 1H), 2.56 (br, 4H), 2.34 (s, 3H), 2.21 (s, 3H), 1.85-1.95 (m, 1H). HRMS calcd. for C_22_H_28_BrN_5_O (M+H^+^) *m/z* 458.15500, found 458.15472.

A mixture of (*S*)-1-(5-bromopyridin-2-yl)-3-(5-methyl-8-(4-methylpiperazin-1-yl)-1,2,3,4-tetrahydronaphthalen-2-yl)urea **166** (60 mg, 0.13 mmol), (4-chloro-2-methylphenyl)boronic acid (67 mg, 0.39 mmol) and aqueous sodium carbonate (2M, 0.39 mL, 0.78 mmol) in toluene (2 mL) and EtOH (1 mL) was purged with nitrogen gas before Pd(dppf)Cl_2_-DCM (5 mg, 0.0065 mmol) was added. The resulting mixture was heated in an oil bath at 85°C overnight. After the solvent was removed, the residue was taken in EtOAc and washed with water and brine, dried over anhydrous sodium sulphate and filtered through a pad of Celite. The solvent was removed to give the crude product, which was purified by column chromatography on silica, using mixtures of MeOH and DCM (v/v=5-10%) as eluent, followed by recrystallisation from DCM and heptane to give **36** as a white solid (47 mg, 71%): HPLC 98.0%. mp 112-115°C. ^1^H NMR (CDCl_3_) δ 9.51 (br d, J = 4.3 Hz, 1H), 9.20 (br, 1H), 7.95 (d, J = 2.1 Hz, 1H), 7.51 (dd, J = 8.5, 2.4 Hz, 1H), 7.28 (d, J = 2.1 Hz, 1H), 7.23 (dd, J = 8.2, 2.1 Hz, 1H), 7.10 (d, J = 8.2 Hz, 1H), 7.01 (d, J = 8.0 Hz, 1H), 6.96 (d, J = 8.4 Hz, 1H), 6.90 (d, J = 8.8 Hz, 1H), 4.26-4.34 (m, 1H), 3.30 (dd, J = 16.3, 4.2 Hz, 1H), 2.92-2.98 (m, 2H), 2.78-2.88 (m, 4H), 2.68-2.74 (m, 2H), 2.57 (br, 4H), 2.34 (s, 3H), 2.24 (s, 3H), 2.20 (s, 3H), 1.89-1.99 (m, 1H). HRMS calcd. for C_29_H_35_ClN_5_O 504.25246, found 504.25242.

*4.1.45. (S)-1-(5-(4-Chloro-2-methylphenyl)pyrazin-2-yl)-3-(5-methyl-8-(4-methylpiperazin-1-yl)-1,2,3,4-tetrahydronaphthalen-2-yl)urea (****37****)*

To a suspension of 5-bromopyrazin-2-amine (0.50 g, 2.87 mmol) and pyridine (0.35 mL, 4.31 mmol) in DCM (10 mL) in an ice bath was added 4-nitrophenyl carbonochloridate (0.70 g, 4.31 mmol) portionwise. The mixture was stirred at room temperature overnight. The resulting precipitate was collected by filtration, washed with DCM, and dried under vacuum to give the product **167** as a white solid (0.920 g, 94%) which was used crude for the next step.

To a solution of **80** (0.250 g, 0.96 mmol) in MeCN (5 mmol) and and DCM (5 mL) at room temperature was added **167** (0.392 g, 1.16 mmol), followed by trimethylamine (0.67 mL, 4.82 mmol). The mixture was stirred overnight and distributed between water and ethyl acetate. The organic phase was washed with water and brine, dried over anhydrous Na_2_SO_4_. The solvent was removed to give the crude product, which was purified by by Davisil® column chromatography, using gradient mixtures of MeOH and DCM (v/v=5~15%) as eluent to give the product **168** as a white solid (0.388 g, 88%): ^1^H NMR (CDCl_3_, 400 MHz) δ 8.99 (br, 1H), 8.37 (br, 1H), 8.18 (s, 1H), 8.06 (d, *J* = 1.3 Hz, 1H), 7.05 (d, *J* = 8.1 Hz, 1H), 6.91 (d, *J* = 8.0 Hz, 1H), 4.20-4.30 (m, 1H), 3.22 (dd, *J* = 16.3, 4.2 Hz, 1H), 2.85-2.95 (m, 4H), 2.77-2.80 (m, 2H), 2.66-2.75 (m, 1H), 2.57 (br, 4H), 2.35 (s, 3H), 2.20 (s, 3H), 1.87-1.95 (m, 1H). HRMS calcd. for C_21_H_27_BrN_6_O (M+H^+^) *m/z* 459.15049, found 459.15085.

The title compound was obtained from (*S*)-1-(5-bromopyrazin-2-yl)-3-(5-methyl-8-(4-methylpiperazin-1-yl)-1,2,3,4-tetrahydronaphthalen-2-yl)urea **168** and (4-chloro-2-methylphenyl)boronic acid using the general procedure B to give **37** (79%) as a white solid. HPLC 97.6%. mp 112-115°C. ^1^H NMR (CDCl_3_) δ 8.90 (br d, J = 7.3 Hz, 1H), 8.81 (br, 1H), 8.38 (d, J = 2.3 Hz, 1H), 8.04 (d, J = 1.3 Hz, 1H), 7.27-7.32 (m, 3H), 7.03 (d, J = 8.0 Hz, 1H), 6.90 (d, J = 8.0 Hz, 1H), 4.26-4.35 (m, 1H), 3.27 (dd, J = 16.4, 4.3 Hz, 1H), 2.71-2.95 (m, 8H), 2.57 (br, 4H), 2.36 (s, 3H), 2.33(s, 3H), 2.20 (s, 3H), 1.90-2.00 (m, 1H). HRMS calcd. for C_28_H_34_ClN_6_O 505.24771, found 505.24768.

*4.1.46. (S)-1-(5-(3,5-Bis(trifluoromethyl)phenyl)pyrazin-2-yl)-3-(5-methyl-8-(4-methylpiperazin-1-yl)-1,2,3,4-tetrahydronaphthalen-2-yl)urea (****38****)*

A mixture of 5-bromopyrazin-2-amine (1.50 g, 8.62 mmol), (3,5-bis(trifluoromethyl)phenyl)boronic acid (2.45 g, 9.48 mmol) and potassium carbonate (4.76 g, 34.5 mmol) in toluene (10 mL) and EtOH (4 mL) was purged with nitrogen gas before Pd(dppf)Cl_2_-DCM (0.352 g, 0.431 mmol) was added. The resulting mixture was heated in an oil bath at 85°C for 2 h. After the solvent was removed, the residue was taken in EtOAc and washed with water and brine, dried over anhydrous sodium sulphate and filtered through a pad of Celite. The solvent was removed to give the crude product, which was purified by column chromatography on silica, using mixtures of hexanes and EtOAc (1:1) as eluent to give **169** as a white solid (2.0 g, 76%). ^1^H NMR (CDCl_3_) δ 8.54 (d, J = 1.5 Hz, 1H), 8.35 (s, 2H), 8.09 (d, J = 1.5 Hz, 1H), 7.85 (s, 1H), 4.79 (s, 2H). LRMS Found: [M+H]= 308.1.

A mixture of **169** (0.60 g, 1.95 mmol) in DCM (10 mL) was cooled to 0 °C and added pyridine (0.185 g, 2.34 mmol) followed by 4-nitrophenyl chloroformate (0.472 g, 2.34 mmol). The reaction was stirred at 0 °C for 2 h. The white precipitate was filtered to give **81** as white solid (0.407 g, 66%) which was used directly for the next step. ^1^H NMR (CDCl_3_) δ 9.43 (d, J = 1.5 Hz, 1H), 8.82 (d, J = 1.5 Hz, 1H), 8.48 (s, 2H), 8.36-8.32 (m, 2H), 7.96 (s, 1H), 7.89 (s, 1H), 7.49-7.44 (m, 2H). LRMS Found: [M+H]= 473.1.

Under nitrogen, a mixture of (*S*)-5-methyl-8-(4-methylpiperazin-1-yl)-1,2,3,4-tetrahydronaphthalen-2-amine **80** (60 mg, 0.23 mmol), 4-nitrophenyl (5-(3,5-bis(trifluoromethyl)phenyl)pyrazin-2-yl)carbamate **81** (100 mg, 0.21 mmol) and triethylamine (0.044 mL, 0.32 mmol) in toluene (4 mL) was heated in an oil bath at 75°C overnight. After the solvent was removed, the residue was taken in EtOAc and washed with 5% cold ammonia three times, dried over anhydrous sodium sulphate and filtered through a pad of alumina. The solvent was removed to give the crude product, which was purified by column chromatography on alumina (the same as above), using a mixture of MeOH and EtOAc (v/v=5%) as eluent, followed by recrystallisation from DCM and heptane to give **38** as a white solid (74 mg, 59%): HPLC 99.6%. mp 218-220°C. ^1^H NMR (CDCl_3_) δ 9.50 (br, 1H), 8.86 (br, 1H), 8.56 (d, J = 1.1 Hz, 1H), 8.50 (s, 1H), 8.39 (s, 2H), 7.90 (s, 1H), 7.05 (d, J = 8.0 Hz, 1H), 6.93 (d, J = 8.0 Hz, 1H), 4.25-4.33 (m, 1H), 3.35 (dd, J = 16.3, 4.2 Hz, 1H), 2.95-3.0 (m, 2H), 2.80-2.90 (m, 5H), 2.60-2.70 (m, 5H), 2.35 (s, 3H), 2.22 (s, 3H), 1.85-1.95 (m, 1H). HRMS calcd. for C_29_H_31_F_6_N_6_O 593.24540, found 593.24610.

*4.1.47. (S)-1-(5-(4-Chloro-2-methylphenyl)pyrimidin-2-yl)-3-(5-methyl-8-(4-methylpiperazin-1-yl)-1,2,3,4-tetrahydronaphthalen-2-yl)urea (****39****)*

To a suspension of 5-bromopyrimidin-2-amine (0.50 g, 2.87 mmol) and pyridine (0.35 mL, 4.31 mmol) in DCM (10 mL) in an ice bath was added 4-nitrophenyl carbonochloridate (0.70 g, 4.31 mmol) portionwise. The mixture was stirred at room temperature overnight. The resulting precipitate was collected by filtration, washed with DCM, and dried under vacuum to give the product **170** as a white solid (970 mg, 100%) which was used crude for the next step.

To a solution of **80** (0.250 g, 0.96 mmol) in MeCN (10 mmol) and and DCM (10 mL) at room temperature was added **170** (0.392 g, 1.16 mmol), followed by trimethylamine (0.67 mL, 4.82 mmol). The mixture was stirred overnight and distributed between water and ethyl acetate. The organic phase was washed with water and brine, dried over anhydrous Na_2_SO_4_. The solvent was removed to give the crude product, which was purified by by Davisil® column chromatography, using gradient mixtures of MeOH and DCM (v/v=5~10%) as eluent to give the product **171** as a white solid (0.321 g, 72%): ^1^H NMR (CDCl_3_, 400 MHz) δ 8.74 (br d, *J* = 7.9 Hz, 1H), 8.43 (s, 2H), 7.71 (s, 1H), 7.12 (d, *J* = 8.0 Hz, 1H), 6.90 (d, *J* = 8.0 Hz, 1H), 4.20-4.30 (m, 1H), 3.24 (dd, *J* = 16.3, 4.2 Hz, 1H), 2.83-2.96 (m, 4H), 2.76-2.80 (m, 2H), 2.66-2.72 (m, 1H), 2.57 (br, 4H), 2.35 (s, 3H), 2.20 (s, 3H), 1.83-1.93 (m, 1H). HRMS calcd. for C_21_H_27_BrN_6_O (M+H^+^) *m/z* 459.15049, found 459.15014.

The title compound was obtained from (*S*)-1-(5-bromopyrimidin-2-yl)-3-(5-methyl-8-(4-methylpiperazin-1-yl)-1,2,3,4-tetrahydronaphthalen-2-yl)urea **171** and (4-chloro-2-methylphenyl)boronic acid using the general procedure B to give **39** (80%) as a white solid. HPLC 95.5%. mp 95-98°C. ^1^H NMR (CDCl_3_) δ 9.07 (d, J = 7.9 Hz, 1H), 8.35 (s, 2H), 7.72-7.78 (br, 1H), 7.32 (d, *J* = 2.0 Hz, 1H), 7.31 (dd, *J* = 8.0, 2.0 Hz, 1H), 7.09 (d, J = 8.0 Hz, 1H), 7.01 (d, J = 8.0 Hz, 1H), 6.89 (d, J = 8.0 Hz, 1H), 4.24-4.33 (m, 1H), 3.26 (dd, J = 16.4, 4.3 Hz, 1H), 2.69-2.94 (m, 8H), 2.56 (br, 4H), 2.34 (s, 3H), 2.27(s, 3H), 2.19 (s, 3H), 1.89-1.95 (m, 1H). HRMS calcd. for C_28_H_34_ClN_6_O 505.24771, found 505.24778.

*4.1.48. (S)-1-(5-(3,5-Bis(trifluoromethyl)phenyl)pyrimidin-2-yl)-3-(5-methyl-8-(4-methylpiperazin-1-yl)-1,2,3,4-tetrahydronaphthalen-2-yl)urea (****40****)*

The title compound was obtained from (*S*)-1-(5-bromopyrimidin-2-yl)-3-(5-methyl-8-(4-methylpiperazin-1-yl)-1,2,3,4-tetrahydronaphthalen-2-yl)urea **171** and (3,5-bis(trifluoromethyl)phenyl)boronic acid using the general procedure B to give **40** (78%) as a white solid. HPLC 99.0%. mp 198-201°C. ^1^H NMR (CDCl_3_) δ 8.98 (d, J = 7.8 Hz, 1H), 8.70 (s, 2H), 7.93 (apparent s, 4H), 7.03 (d, J = 8.0 Hz, 1H), 6.91 (d, J = 8.0 Hz, 1H), 4.22-4.32 (m, 1H), 3.32 (dd, J = 16.4, 4.1 Hz, 1H), 2.90-2.98 (m, 2H), 2.78-2.88 (m, 4H), 2.64-2.71 (m, 1H), 2.57 (br, 4H), 2.34 (s, 3H), 2.21-2.27(m, 1H), 2.21(s, 3H), 1.84-1.94 (m, 1H). HRMS calcd. for C_29_H_31_F_6_N_6_O 593.24581, found 593.24671.

*4.1.49. General Procedure C: (S)-N-(5-Methyl-8-(4-methylpiperazin-1-yl)-1,2,3,4-tetrahydronaphthalen-2-yl)-4-phenylpiperazine-1-carboxamide (****41****)*

To a solution of (*S*)-5-methyl-8-(4-methylpiperazin-1-yl)-1,2,3,4-tetrahydronaphthalen-2-amine **80** (60 mg, 0.23 mmol) in DCM (4 mL) over an ice bath, trimethylamine (0.097 mL, 0.69 mmol) was added followed by 4-nitrophenyl chloroformate (54 mg, 0.27 mmol). The resulting solution of 4-nitrophenyl (*S*)-(5-methyl-8-(4-methylpiperazin-1-yl)-1,2,3,4-tetrahydronaphthalen-2-yl)carbamate was allowed to warm up to room temperature and stirred for 3 hours, before 1-phenylpiperazine (49 mg, 0.30 mmol) was added followed by more trimethylamine (0.161 mL, 1.16 mmol). The resulting mixture was stirred at room temperature overnight. After the solvent was removed, the residue was taken in EtOAc and washed with 5% cold ammonia three times, dried over anhydrous sodium sulphate and filtered through a pad of alumina. The solvent was removed to give the crude product, which was purified by column chromatography on silica using a mixture of MeOH and DCM (v/v=5~10%) as eluent, followed by recrystallisation from DCM and heptane to give **41** as a white solid (80 mg, 77%): HPLC 99.2%. mp 190-191°C. ^1^H NMR (CDCl_3_) δ 7.28-7.30 (m, 2H), 7.02 (d, J = 8.0 Hz, 1H), 6.88-6.94 (m, 4H), 4.47 (d, J = 7.4 Hz, 1H), 4.13-4.19 (m, 1H), 3.48-3.56 (m, 4H), 3.14-3.24 (m, 5H), 2.83-2.94 (m, 4H), 2.75 (t, J = 6.5 Hz, 2H), 2.52-2.58 (m, 5H), 2.36 (s, 3H), 2.20 (s, 3H), 2.13-2.17 (m, 1H), 1.72-1.81 (m, 1H). HRMS calcd. for C_27_H_38_N_5_O 448.30823, found 448.30819.

*4.1.50. (S)-4-(4-Methoxyphenyl)-N-(5-methyl-8-(4-methylpiperazin-1-yl)-1,2,3,4-tetrahydronaphthalen-2-yl)piperazine-1-carboxamide (****42****)*

The title compound was obtained from (*S*)-5-methyl-8-(4-methylpiperazin-1-yl)-1,2,3,4-tetrahydronaphthalen-2-amine **80** and 1-(4-methoxyphenyl)piperazine using the general procedure C to give **42** (82%) as a white foam. HPLC 95.5%. mp 182-185°C. ^1^H NMR (CDCl_3_) δ 7.02 (d, J = 8.0 Hz, 1H), 6.89-6.92 (m, 3H), 6.78 (AA’BB’ pattern, J = 9.2 Hz, 2H), 4.46 (d, J = 7.4 Hz, 1H), 4.13-4.20 (m, 1H), 3.77 (s, 3H), 3.48-3.55 (m, 4H), 3.21 (dd, J = 16.3, 4.4 Hz, 1H), 3.06 (apparent t, J = 5.1 Hz, 4H), 2.83-2.93 (m, 4H), 2.74 (t, J = 6.5 Hz, 2H), 2.52-2.58 (m, 5H), 2.36 (s, 3H), 2.20 (s, 3H), 2.13-2.18 (m, 1H), 1.73-1.80 (m, 1H). HRMS calcd. for C_28_H_40_N_5_O_2_ 478.31879, found 478.31864.

*4.1.51. (S)-4-(4-Fluorophenyl)-N-(5-methyl-8-(4-methylpiperazin-1-yl)-1,2,3,4-tetrahydronaphthalen-2-yl)piperazine-1-carboxamide (****43****)*

The title compound was obtained from (*S*)-5-methyl-8-(4-methylpiperazin-1-yl)-1,2,3,4-tetrahydronaphthalen-2-amine **80** and 1-(4-fluorophenyl)piperazine using the general procedure C to give **43** (74%) as a white solid. HPLC 95.5%. mp 189-190°C. ^1^H NMR (CDCl_3_) δ 6.95-7.03 (m, 3H), 6.86-6.92 (m, 3H), 4.46 (d, J = 7.4 Hz, 1H), 4.12-4.20 (m, 1H), 3.47-3.57 (m, 4H), 3.21 (dd, J = 16.3, 4.5 Hz, 1H), 3.10 (apparent t, J = 5.1 Hz, 4H), 2.83-2.93 (m, 4H), 2.74 (t, J = 6.6 Hz, 2H), 2.52-2.59 (m, 5H), 2.35 (s, 3H), 2.20 (s, 3H), 2.13-2.19 (m, 1H), 1.74-1.79 (m, 1H). ^13^C NMR (CDCl_3_) δ 158.9, 157.3, 156.5, 150.0, 148.0, 148.0, 135.5, 132.0, 130.2, 128.1, 118.6, 118.6, 117.3, 116.0, 115.8, 55.9, 52.3, 50.4, 46.4, 46.4, 44.1, 32.5, 29.6, 25.9, 19.6. HRMS calcd. for C_27_H_37_FN_5_O 466.29818, found 466.29871.

*4.1.52. (S)-4-(4-Trifluoromethylphenyl)-N-(5-methyl-8-(4-methylpiperazin-1-yl)-1,2,3,4-tetrahydronaphthalen-2-yl)piperazine-1-carboxamide (****44****)*

The title compound was obtained from (*S*)-5-methyl-8-(4-methylpiperazin-1-yl)-1,2,3,4-tetrahydronaphthalen-2-amine **80** and 1-(4-trifluoromethylphenyl)piperazine using the general procedure C to give **44** (75%) as a white solid. HPLC 95.5%. mp 168-170°C. ^1^H NMR (CDCl_3_) δ 7.50 (d, J = 8.6 Hz, 2H), 7.02 (d, J = 8.0 Hz, 1H), 6.91 (apparent d, J = 8.2 Hz, 3H), 4.45 (d, J = 7.4 Hz, 1H), 4.12-4.21 (m, 1H), 3.51-3.56 (m, 4H), 3.31 (apparent t, J = 5.2 Hz, 4H), 3.21 (dd, J = 16.5, 4.5 Hz, 1H), 2.83-2.93 (m, 4H), 2.75 (t, J = 6.6 Hz, 2H), 2.53-2.59 (m, 5H), 2.35 (s, 3H), 2.20 (s, 3H), 2.13-2.17 (m, 1H), 1.74-1.81 (m, 1H). HRMS calcd. for C_28_H_37_F_3_N_5_O 516.29447, found 516.29506.

*4.1.53. (S)-4-(4-Bromophenyl)-N-(5-methyl-8-(4-methylpiperazin-1-yl)-1,2,3,4-tetrahydronaphthalen-2-yl)piperazine-1-carboxamide (****45****)*

The title compound was obtained from (*S*)-5-methyl-8-(4-methylpiperazin-1-yl)-1,2,3,4-tetrahydronaphthalen-2-amine **80** and 1-(4-bromophenyl)piperazine using the general procedure C to give **45** (36%) as a white solid. HPLC 97.95%. mp 174-176°C. ^1^H NMR (CDCl_3_) δ 7.36 (AA’BB’ pattern, *J* = 9.0 Hz, 2H), 7.02 (d, J = 8.0 Hz, 1H), 6.91 (d, J = 8.0 Hz, 1H), 6.78 (AA’BB’ pattern, J = 9.0 Hz, 2H), 4.45 (d, *J* = 7.4 Hz, 1H), 4.13-4.20 (m, 1H), 3.52 (apparent dd, J = 6.5, 4.1 Hz, 4H), 3.14-3.23 (m, 5H), 2.83-2.94 (m, 4H), 2.75 (t, J = 6.6 Hz, 2H), 2.52-2.59 (m, 5H), 2.36 (s, 3H), 2.20 (s, 3H), 2.10-2.18 (m, 1H), 1.60-1.70 (m, 1H). HRMS calcd. for C_27_H_37_BrN_5_O 526.21760, found 526.21897.

*4.1.54. (S)-N-(5-Methyl-8-(4-methylpiperazin-1-yl)-1,2,3,4-tetrahydronaphthalen-2-yl)-4-(pyridin-4-yl)piperazine-1-carboxamide (****46****)*

The title compound was obtained from (*S*)-5-methyl-8-(4-methylpiperazin-1-yl)-1,2,3,4-tetrahydronaphthalen-2-amine **80** and 1-(pyridin-4-yl)piperazine using the general procedure C to give **46** (64%) as a white solid. HPLC 97.8%. mp 88°C. ^1^H NMR (CDCl_3_) δ 8.29 (AA’XX’ pattern, J = 6.6 Hz, 2H), 7.02 (d, J = 8.0 Hz, 1H), 6.91 (d, J = 8.0 Hz, 1H), 6.64 (AA’XX’ pattern, J = 6.6 Hz, 2H), 4.45 (d, J = 7.4 Hz, 1H), 4.13-4.20 (m, 1H), 3.52 (apparent dd, J = 6.5, 4.0 Hz, 4H), 3.40 (apparent t, J = 5.8 Hz, 4H), 3.20 (dd, J = 16.4, 4.5 Hz, 1H), 2.83-2.94 (m, 4H), 2.74 (t, J = 6.6 Hz, 2H), 2.53-2.59 (m, 5H), 2.35 (s, 3H), 2.20 (s, 3H), 2.10-2.18 (m, 1H), 1.63-1.73 (m, 1H). HRMS calcd. for C_26_H_37_N_6_O 449.30348, found 449.30437.

*4.1.55. (S)-N-(5-Methyl-8-(4-methylpiperazin-1-yl)-1,2,3,4-tetrahydronaphthalen-2-yl)-4-(pyridin-2-yl)piperazine-1-carboxamide (****47****)*

The title compound was obtained from (*S*)-5-methyl-8-(4-methylpiperazin-1-yl)-1,2,3,4-tetrahydronaphthalen-2-amine **80** and 1-(pyridin-2-yl)piperazine using the general procedure C to give **47** (67%) as a white solid. HPLC 99.6%. mp 192-194°C. ^1^H NMR (CDCl_3_) δ 8.18-8.20 (m, 1H), 7.03-7.52 (m, 1H), 7.02 (d, J = 8.0 Hz, 1H), 6.91 (d, J = 8.0 Hz, 1H), 6.63-6.67 (m, 1H), 4.45 (d, J = 7.4 Hz, 1H), 4.13-4.20 (m, 1H), 3.58-3.62 (m, 4H), 3.50-3.55 (m, 4H), 3.22 (dd, J = 16.4, 4.5 Hz, 1H), 2.82-2.93 (m, 4H), 2.75 (t, J = 6.4 Hz, 2H), 2.52-2.58 (m, 5H), 2.35 (s, 3H), 2.20 (s, 3H), 2.12-2.18 (m, 1H), 1.70-1.80 (m, 1H). HRMS calcd. for C_26_H_37_N_6_O 449.30348, found 449.30381.

*4.1.56. (S)-4-(4-Chloro-2-methylphenyl)-N-(5-methyl-8-(4-methylpiperazin-1-yl)-1,2,3,4-tetrahydronaphthalen-2-yl)piperazine-1-carboxamide (****48****)*

The title compound was obtained from (*S*)-5-methyl-8-(4-methylpiperazin-1-yl)-1,2,3,4-tetrahydronaphthalen-2-amine **80** and 1-(4-chloro-2-methylphenyl)piperazine using the general procedure C to give **48** (65%) as a white solid. HPLC 98.8%. mp 193-196°C. 1H NMR (CDCl_3_) δ 7.17 (d, J = 2.4 Hz, 1H), 7.13 (dd, J = 8.5, 2.5 Hz, 1H), 7.02 (d, J = 8.0 Hz, 1H), 6.91 (d, J = 8.5 Hz, 1H), 4.47 (d, J = 7.4 Hz, 1H), 4.12-4.21 (m, 1H), 3.45-3.55 (m, 4H), 3.21 (dd, J = 16.5, 4.6 Hz, 1H), 2.82-2.94 (m, 8H), 2.75 (t, J = 6.6 Hz, 2H), 2.52-2.59 (m, 5H), 2.36 (s, 3H), 2.28 (s, 3H), 2.20 (s, 3H), 2.12-2.19 (m, 1H), 1.72-1.80 (m, 1H). HRMS calcd. for C_28_H_39_ClN_5_O 496.28377, found 496.28576.

*4.1.57. (S)-4-(3,5-Bis(trifluoromethyl)phenyl)-N-(5-methyl-8-(4-methylpiperazin-1-yl)-1,2,3,4-tetrahydronaphthalen-2-yl)piperazine-1-carboxamide (****49****)*

The title compound was obtained from (*S*)-5-methyl-8-(4-methylpiperazin-1-yl)-1,2,3,4-tetrahydronaphthalen-2-amine **80** and 1-(3,5-bis(trifluoromethyl)phenyl)piperazine using the general procedure C to give **49** (60%) as a white solid. HPLC 99.3%. mp 211-214°C. ^1^H NMR (CDCl_3_) δ 7.31 (s, 1H), 7.23 (s, 2H), 7.03 (d, J = 8.0 Hz, 1H), 6.91 (d, J = 8.0 Hz, 1H), 4.49 (d, J = 7.4 Hz, 1H), 4.13-4.21 (m, 1H), 3.56-3.58 (m, 4H), 3.33 (apparent t, J = 5.2 Hz, 4H), 3.21 (dd, J = 16.5, 4.6 Hz, 1H), 2.83-2.93 (m, 4H), 2.75 (t, J = 6.6 Hz, 2H), 2.54-2.61 (m, 5H), 2.35 (s, 3H), 2.20 (s, 3H), 2.10-2.20 (m, 1H), 1.74-1.83 (m, 1H). HRMS calcd. for C_29_H_36_F_6_N_5_O 584.28316, found 584.28327.

*4.1.58. (S)-N-(5-Methyl-8-(4-methylpiperazin-1-yl)-1,2,3,4-tetrahydronaphthalen-2-yl)-4-(4-(trifluoromethyl)phenyl)piperazine-1-carboxamide (****50****)*

The title compound was obtained from (*S*)-5-methyl-8-(4-methylpiperazin-1-yl)-1,2,3,4-tetrahydronaphthalen-2-amine **80** and *N*-(4-(trifluoromethyl)-phenyl)piperidin-4-amine using the general procedure C to give **50** (47%) as a white solid. HPLC 98.9%. mp 92-95 ^o^C. ^1^H NMR (CDCl_3_) δ 7.40 (d, J = 8.5 Hz, 2H), 7.02 (d, J = 8.0 Hz, 1H), 6.91 (d, J = 8.0 Hz, 1H), 6.60 (d, J = 8.5 Hz, 2H), 4.46 (d, *J* = 7.4 Hz, 1H), 4.09-4.18 (m, 1H), 3.85-3.95 (m, 3H), 3.45-3.55 (m, 1H), 3.20 (dd, J = 16.4, 4.5 Hz, 1H), 2.83-3.02 (m, 6H), 2.74 (t, J = 6.6 Hz, 2H), 2.50-2.57 (m, 5H), 2.36 (s, 3H), 2.19 (s, 3H), 2.11-2.16 (m, 1H), 2.04-2.09 (m, 2H), 1.71-1.77 (m, 1H), 1.38-1.44 (m, 2H). HRMS calcd. for C_29_H_39_F_3_N_5_O 530.31114, found 530.31131.

*4.1.59. (S)-4-((4-Chloro-2-methylphenyl)amino)-N-(5-methyl-8-(4-methylpiperazin-1-yl)-1,2,3,4-tetrahydronaphthalen-2-yl)piperidine-1-carboxamide (****51****)*

The title compound was obtained from (*S*)-5-methyl-8-(4-methylpiperazin-1-yl)-1,2,3,4-tetrahydronaphthalen-2-amine **80** and *N*-(4-chloro-2-methylphenyl)piperidin-4-amine using the general procedure C to give **51** (70%) as a white solid. HPLC 99.7%. mp 84-87 ^o^C. ^1^H NMR (CDCl_3_) δ 7.00-7.07 (m, 3H), 6.90 (d, J = 8.0 Hz, 1H), 6.53 (d, J = 8.4 Hz, 1H), 4.46 (d, J = 7.4 Hz, 1H), 4.09-4.18 (m, 1H), 3.84-3.93 (m, 2H), 3.43 (br, 1H), 3.26 (br, 1H), 3.20 (dd, J = 16.4, 4.5 Hz, 1H), 2.84-3.03 (m, 6H), 2.74 (t, J = 6.6 Hz, 2H), 2.50-2.56 (m, 5H), 2.36 (s, 3H), 2.19 (s, 3H), 2.05-2.18 (m, 6H), 1.70-1.79 (m, 1H), 1.38-1.48 (m, 2H). HRMS calcd. for C_29_H_41_ClN_5_O 510.29942, found 510.30013.

*4.1.60. (S)-4-((3,5-Bis(trifluoromethyl)phenyl)amino)-N-(5-methyl-8-(4-methylpiperazin-1-yl)-1,2,3,4-tetrahydronaphthalen-2-yl)piperidine-1-carboxamide (****52****)*

The title compound was obtained from (*S*)-5-methyl-8-(4-methylpiperazin-1-yl)-1,2,3,4-tetrahydronaphthalen-2-amine **80** and *N*-(2,4-bis(trifluoromethyl)phenyl)piperidin-4-amine using the general procedure C to give **52** (62%) as a white solid. HPLC 99.3%. mp 104-107 °C. ^1^H NMR (CDCl_3_) δ 7.14 (s, 1H), 7.02 (d, *J* = 8.0 Hz, 1H), 6.90-6.92 (m, 3H), 4.47 (d, J = 7.4 Hz, 1H), 4.10-4.18 (m, 1H), 4.01 (d, J = 7.8 Hz, 1H), 3.89-3.96 (m, 2H), 3.45-3.55 (m, 1H), 3.20 (dd, J = 16.4, 4.5 Hz, 1H), 2.97-3.05 (m, 2H), 2.82-2.92 (m, 4H), 2.74 (t, J = 6.6 Hz, 2H), 2.51-2.57 (m, 5H), 2.36 (s, 3H), 2.19 (s, 3H), 2.06-2.16 (m, 3H), 1.70-1.80 (m, 1H), 1.38-1.50 (m, 2H). HRMS calcd. for C_30_H_38_F_6_N_5_O 598.29802, found 598.29824.

*4.1.61. (S)-5-(4-Chloro-2-methylphenyl)-N-(8-(4-methylpiperazin-1-yl)-1,2,3,4-tetrahydronaphthalen-2-yl)picolinamide (****53****)*

The title compound was obtained from (*S*)-8-(4-methylpiperazin-1-yl)-1,2,3,4-tetrahydronaphthalen-2-amine **82** and **83** using the general procedure A to give **53** (59%) as a white solid. HPLC 99.9%. mp 72-75°C. ^1^H NMR (CDCl_3_) δ 8.49 (dd, J = 2.2, 0.7 Hz, 1H), 8.30 (dd, J = 8.0, 0.6 Hz, 1H), 8.10 (d, J = 8.4 Hz, 1H), 7.79 (dd, J = 8.0, 2.2 Hz, 1H), 7.34-7.6 (m, 2H), 7.18-7.12 (m, 2H), 6.97 (d, J = 7.7 Hz, 1H), 6.91 (d, J = 7.5 Hz, 1H), 4.49-4.40 (m, 1H), 3.34 (dd, J = 16.5, 4.8 Hz, 1H), 3.05-2.94 (m, 4H), 2.92-2.84 (m, 2H), 2.70-2.51 (m, 5H), 2.36 (s, 3H), 2.26 (s, 3H), 2.25-2.18 (m, 1H), 1.94-1.83 (m, 1H). ^13^C NMR (CDCl_3_) δ 163.7, 152.0, 149.1, 148.3, 139.2, 138.0, 137.7, 137.0, 136.1, 134.6, 131.2, 130.8, 130.1, 126.7, 126.6, 124.6, 122.1, 117.5, 55.8, 52.2, 46.3, 45.9, 31.8, 29.3, 28.3, 20.5. HRMS calcd. for C_28_H_31_ClN_4_O: 475.2259, found 475.2259.

*4.1.62. (S)-5-(4-Chloro-2-methylphenyl)-N-(8-(4-methylpiperazin-1-yl)-5-phenyl-1,2,3,4-tetrahydronaphthalen-2-yl)picolinamide (****54****)*

*Step i*. 8-Bromo-3,4-dihydronaphthalen-2(1*H*)-one (18.39 g, 81.7 mmol) in toluene (50 mL) was added pTSA (0.155 g, 0.817 mmol) and (*R*)-*N-*ethylphenylamine (11.59 mL, 89.9 mmol). The reaction mixture was heated to 50 °C for 2 h. The reaction mixture was cooled to 0 °C, sodium borohydride (4.95 g, 130.7 mmol) in methanol:isopropanol (2:3) was added in portions. The reaction mixture was heated at 70 °C for 18 h. The reaction mixture was quenched with water (50 mL), extracted with ethyl acetate (3 x 20 mL) and evaporated. The crude product was dissolved in ethyl acetate (20 mL), added anhydrous HCl (24.5 mL, 4M in dioxane) dropwise. The mixture was sonicated until white precipitate forms. The precipitate was filtered, collected into a flask. The white precipitate was added ethyl acetate:ethanol (2:1, 100 mL) and heated at 50 °C for 3 h. The mixture was cooled to 5 °C for 30 min and filtered to give (*S*)-8-bromo*-N*-((*R*)-1-phenylethyl)-1,2,3,4-tetrahydronaphthalen-2-amine **86** as white solids (11.0 g, 37%). αD= -39.3 °. ^1^H NMR (CDCl_3_) δ 10.43-10.23 (m, 2H), 7.78 (d, J = 7.2 Hz, 2H), 7.44 (t, J = 7.2 Hz, 2H), 7.39-7.34 (m, 1H), 7.29-7.27 (m, 1H), 6.94-6.89 (m, 2H), 4.60-4.50 (m, 1H), 3.52 (d, J = 13.0 Hz, 1H), 3.19-3.02 (m, 2H), 2.92 (dd, J = 15.1, 2.9 Hz, 1H), 2.63 (dt, J = 12.6, 5.4 Hz, 1H), 2.45 (dd, J = 12.1, 2.5 Hz, 1H), 2.24-2.12 (m, 1H), 2.08 (d, J = 6.8 Hz, 3H). LRMS Found: [M+H]= 330.2.

*Step ii*. (*S*)-8-Bromo-*N*-((*R*)-1-phenylethyl)-1,2,3,4-tetrahydronaphthalen-2-amine **86** (2.00 g, 5.45 mmol) in acetonitrile (30 mL) was added potassium iodide (0.045 g, 0.273 mmol), K_2_CO_3_ (1.88 g, 13.6 mmol) followed by benzyl bromide (0.78 mL, 6.54 mmol). The reaction mixture was refluxed at 150 °C in a sealed tube for 27 h. The reaction mixture was diluted with EtOAc and washed with water. The organic layer was dried over anhydrous Na_2_SO_4_, filtered and evaporated. The residue purified by silica column chromatography using hexanes:EtOAc (v/v=2%) to give (*S*)-*N*-benzyl-8-bromo-*N*-((*R*)-1-phenylethyl)-1,2,3,4-tetrahydronaphthalen-2-amine **87** as a white foam (1.87 g, 82%). ^1^H NMR (CDCl_3_) δ 7.48 (d, J = 7.1 Hz, 2H), 7.39 (d, J = 7.1 Hz, 2H), 7.36-7.28 (m, 5H), 7.24-7.19 (m, 2H), 6.97-6.89 (m, 2H), 4.02 (q, J = 6.9 Hz, 1H), 3.92 (d, J = 15.1 Hz, 1H), 3.75 (d, J = 15.2 Hz, 1H), 3.18-3.09 (m, 1H), 3.02 (dd, J = 17.0, 5.3 Hz, 1H), 2.76-2.58 (m, 3H), 1.66-1.60 (m, 1H), 1.51-1.45 (m, 1H), 1.39 (d, J = 6.9 Hz, 3H). LRMS Found: [M+H]= 420.2.

*Step iii*. (*S*)-*N*-benzyl-8-bromo-*N*-((*R*)-1-phenylethyl)-1,2,3,4-tetrahydronaphthalen-2-amine **87** (1.87 g, 4.45 mmol) was dissolved in toluene (30 mL) and flushed with nitrogen for 5 min. Palladium acetate (0.04 g, 0.178 mmol), BINAP (0.22 g, 0.356 mmol) and N-methyl piperazine (0.662 g, 6.68 mmol) was added to the reaction mixture. The reaction was heated to 80 °C for 30 min. Sodium tert-butoxide (0.599 g, 6.23 mmol) was added and heated to 100 °C for a further 3 h. The reaction mixture was diluted with EtOAc and washed with water. The organic layer was dried over anhydrous Na_2_SO_4_, filtered and evaporated. The residue purified by silica column chromatography using EtOAc to give (*S*)-*N*-benzyl-8-(4-methylpiperazin-1-yl)-*N*-((*R*)-1-phenylethyl)-1,2,3,4-tetrahydronaphthalen-2-amine **88** as a white solid (1.25 g, 64%). ^1^H NMR (CDCl_3_) δ 7.48 (d, J = 7.1 Hz, 2H), 7.40 (d, J = 7.1 Hz, 2H), 7.34-7.28 (m, 4H), 7.24-7.18 (m, 2H), 7.05 (t, J = 7.7 Hz, 1H), 6.85 (d, J = 7.7 Hz, 1H), 6.76 (d, J = 7.4 Hz, 1H), 4.02 (q, J = 6.8 Hz, 1H), 3.92 (d, J = 15.4 Hz, 1H), 3.79 (d, J = 15.4 Hz, 1H), 3.10-3.02 (m, 2H), 2.99-2.92 (m, 2H), 2.81-2.73 (m, 3H), 2.67-2.45 (m, 6H), 2.39 (s, 3H), 1.76-1.68 (m, 1H), 1.58-1.51 (m, 1H), 1.40 (d, J = 6.8 Hz, 3H). LRMS Found: [M+H]= 440.3.

*Step iv*. (*S*)-*N*-Benzyl-8-(4-methylpiperazin-1-yl)-*N*-((*R*)-1-phenylethyl)-1,2,3,4-tetrahydronaphthalen-2-amine **88** (3.11 g, 7.07 mmol) in DMF (20 mL) was added N-bromosuccinamide (1.64 g, 9.20 mmol). The reaction was stirred at room temperature for 72 h. The reaction mixture was diluted with EtOAc and washed with water. The organic layer was dried over anhydrous Na_2_SO_4_, filtered and evaporated. The residue purified by silica column chromatography using EtOAc:MeOH (v/v=5%) to give (*S*)-N-benzyl-5-bromo-8-(4-methylpiperazin-1-yl)-N-((R)-1-phenylethyl)-1,2,3,4-tetrahydronaphthalen-2-amine **89** as white solids (2.89 g, 79%). ^1^H NMR (CDCl_3_) δ 7.47 (d, J = 7.1 Hz, 2H), 7.39 (d, J = 7.1 Hz, 2H), 7.34-7.28 (m, 5H), 7.24-7.19 (m, 2H), 6.73 (d, J = 8.5 Hz, 1H), 4.03 (q, J = 6.7 Hz, 1H), 3.92 (d, J = 15.4 Hz, 1H), 3.79 (d, J = 15.4 Hz, 1H), 3.10-3.01 (m, 2H), 2.99-2.92 (m, 2H), 2.76-2.68 (m, 3H), 2.58-2.42 (m, 6H), 2.39 (s, 3H), 1.81-1.76 (m, 1H), 1.58-1.52 (m, 1H), 1.41 (d, J = 6.8 Hz, 3H). LRMS Found: [M+H]= 518.2.

*Step v*. **89** (0.283 g, 0.546 mmol) was dissolved in toluene:EtOH (10:4 mL) and flushed with nitrogen for 5 min. Phenylboronic acid (0.073 g, 0.60 mmol) and K_2_CO_3_ (1.09 mL, 2N solution, 2.18 mmol) was added to the reaction mixture. The reaction mixture was bubbled nitrogen for 5 min, followed by addition of PddppfCl_2_.DCM (0.045 g, 0.055 mmol). The reaction was heated in a sealed tube at 80 °C for 3 h. The solvent was removed, and purified by silica column chromatography using EtOAc:MeOH (v/v=15%) to give **91** (0.084 g, 30%). ^1^H NMR (CDCl_3_) δ 7.46 (d, J = 7.2 Hz, 2H), 7.42-7.36 (m, 2H), 7.36-7.26 (m, 7H), 7.23-7.16 (m, 4H), 7.00 (d, J = 8.0 Hz, 1H), 6.93 (d, J = 8.1 Hz, 1H), 4.03 (q, J = 6.8 Hz, 1H), 3.90 (d, J = 15.4 Hz, 1H), 3.75 (d, J = 15.4 Hz, 1H), 3.18-3.03 (m, 2H), 3.02-2.92 (m, 2H), 2.91-2.78 (m, 2H), 2.71-2.43 (m, 7H), 2.39 (s, 3H), 1.72-1.65 (m, 1H), 1.49-1.42 (m, 1H), 1.41 (d, J = 6.8 Hz, 3H). LRMS Found: [M+H]= 516.3.

*Step vi*. **91** (0.185 g, 0.36 mmol) was dissolved in MeOH (20 mL) and added AcOH (1 mL). The reaction was hydrogenated over 10% Pd-C (0.20 g) at 60 psi for 48 h. The catalyst was filtered off and the filtrate concentrated to dryness to give pure **93** as a colorless, viscous oil which was used directly for the next step. 5-(4-Chloro-2-methylphenyl)picolinic acid **83** (0.100 g, 0.43 mmol) in DMF (5 mL) was purged with nitrogen before DIPEA (0.093 g, 0.72 mmol) was added to the reaction mixture. HATU (0.177 g, 0.47 mmol) was added and stirred for 15 mins. (*S*)-8-(4-methylpiperazin-1-yl)-5-phenyl-1,2,3,4-tetrahydronaphthalen-2-amine **93** (0.115 g, 0.36 mmol) was added to the reaction mixture and stirred at r.t. for 1 h. The reaction mixture was diluted with EtOAc acetate and washed with water and 2M NaOH solution. The organic layer was dried over anhydrous Na_2_SO_4_ and filtered through a pad of Celite. The solvent was removed to give the crude product, which was purified by silica column chromatography using EtOAc:MeOH (v/v=20%) as eluent to give **54** as a white foam (0.081 g, 41%). mp 95-98°C. HPLC 97.4%. ^1^H NMR (CDCl_3_) δ 8.49 (dd, J = 2.1, 0.7 Hz, 1H), 8.30 (dd, J = 8.0, 0.7 Hz, 1H), 8.10 (d, J = 8.5 Hz, 1H), 7.79 (dd, J = 8.0, 2.2 Hz, 1H), 7.43-7.37 (m, 2H), 7.36-7.27 (m, 5H), 7.15 (d, J = 8.2 Hz, 1H), 7.10 (d, J = 8.1 Hz, 1H), 7.06 (d, J = 8.1 Hz, 1H), 4.49-4.40 (m, 1H), 3.44 (dd, J = 16.4, 4.7 Hz, 1H), 3.04-2.97 (m, 2H), 2.96-2.89 (m, 2H), 2.86-2.78 (m, 2H), 2.76-2.53 (m, 5H), 2.37 (s, 3H), 2.26 (s, 3H), 2.18-2.10 (m, 1H), 1.81-1.72 (m, 1H). ^13^C NMR (CDCl_3_) δ 163.7, 151.0, 149.0, 148.3, 142.1, 139.2, 138.0, 137.9, 137.7, 136.0, 134.7, 134.6, 131.2, 130.8, 130.5, 129.5, 128.3, 128.3, 126.9, 126.6, 122.1, 117.6, 55.6, 52.1, 46.1, 45.6, 32.2, 29.5, 27.5, 20.5. HRMS calcd. for C_34_H_35_ClN_4_O: 550.2499, found 550.2500.

*4.1.63. (S)-N-(5-(4-(tert-Butyl)phenyl)-8-(4-methylpiperazin-1-yl)-1,2,3,4-etrahydronaphthalen-2-yl)-5-(4-chloro-2-methylphenyl)picolinamide (****55****)*

*Step v* as preparation of **91** but using (*S*)-N-benzyl-5-bromo-8-(4-methylpiperazin-1-yl)-*N*-((*R*)-1-phenylethyl)-1,2,3,4-tetrahydronaphthalen-2-amine **89** and (4-(tert-butyl)phenyl)boronic acid to give **92** (49%) as white solids. ^1^H NMR (CDCl_3_) δ 7.46 (d, J = 7.2 Hz, 2H), 7.41-7.34 (m, 4H), 7.33-7.26 (m, 4H), 7.23-7.16 (m, 2H), 7.15 (d, J = 8.3 Hz, 2H), 7.01 (d, J = 8.1 Hz, 1H), 6.93 (d, J = 8.1 Hz, 1H), 4.03 (q, J = 7.0 Hz, 1H), 3.90 (d, J = 15.4 Hz, 1H), 3.75 (d, J = 15.4 Hz, 1H), 3.17-3.03 (m, 2H), 3.02-2.94 (m, 2H), 2.89-2.81 (m, 2H), 2.69-2.43 (m, 7H), 2.40 (s, 3H), 1.72-1.66 (m, 1H), 1.50-1.42 (m, 1H), 1.40 (d, J = 6.8 Hz, 3H). LRMS Found: [M+H]= 572.4.

*Step vi* as preparation of **54** but using **92** and 5-(4-chloro-2-methylphenyl)picolinic acid **82** to give **55** (28%) as white foam. HPLC 99.4%. mp 117-120°C. ^1^H NMR (CDCl_3_) δ 8.49 (dd, J = 2.1, 0.7 Hz, 1H), 8.30 (dd, J = 8.0, 0.7 Hz, 1H), 8.10 (d, J = 8.5 Hz, 1H), 7.79 (dd, J = 8.0, 2.2 Hz, 1H), 7.43-7.38 (m, 2H), 7.32 (d, J = 2.0 Hz, 1H), 7.29-7.25 (m, 1H), 7.25-7.21 (m, 2H), 7.15 (d, J = 8.2 Hz, 1H), 7.11 (d, J = 8.1 Hz, 1H), 7.04 (d, J = 8.1 Hz, 1H), 4.49-4.40 (m, 1H), 3.43 (dd, J = 16.7, 5.3 Hz, 1H), 3.04-2.97 (m, 2H), 2.96-2.90 (m, 2H), 2.86-2.78 (m, 2H), 2.76-2.53 (m, 5H), 2.36 (s, 3H), 2.26 (s, 3H), 2.16-2.10 (m, 1H), 1.79-1.71 (m, 1H), 1.36 (s, 9H). HRMS calcd. for C_38_H_43_ClN_4_O: 606.3125, found 606.3129.

*4.1.64. (S)-N-(5-Benzyl-8-(4-methylpiperazin-1-yl)-1,2,3,4-tetrahydronaphthalen-2-yl)-5-(4-chloro-2-methylphenyl)picolinamide (****56****)*

*Step i*. **89** (0.868 g, 1.67 mmol) was dissolved in THF (20 mL) and cooled to -78 °C. n-BuLi (1.09 mL, 2M solution in diethyl ether, 2.18 mmol) was added followed by benzaldehyde (0.532 g, 5.31 mmol). The reaction was stirred at -78 °C for 5 h. The reaction mixture was added water and extracted with EtOAc. The organic layer was dried over anhydrous Na_2_SO_4_, filtered and evaporated. The residue purified by silica column chromatography using EtOAc:MeOH (v/v=5%) to give **95** (0.096 g, 11%). ^1^H NMR (CDCl_3_) δ 7.45-7.39 (m, 2H), 7.38-7.34 (m, 2H), 7.33-7.25 (m, 8H), 7.24-7.18 (m, 4H), 6.90 (d, J = 8.3 Hz, 1H), 5.92 (d, J = 10.4 Hz, 1H), 4.03 (q, J = 6.8 Hz, 1H), 3.88 (d, J = 15.6 Hz, 1H), 3.74 (d, J = 15.3 Hz, 1H), 3.12-3.03 (m, 1H), 3.02-2.85 (m, 4H), 2.80-2.70 (m, 3H), 2.62-2.47 (m, 5H), 2.39 (s, 3H), 1.72-1.65 (m, 1H), 1.53-1.49 (m, 1H), 1.40 (d, J = 6.9 Hz, 3H). LRMS Found: [M+H]= 546.3.

*Step ii*. **95** (0.1 g, 0.183 mmol) was dissolved in DCM (20 mL), added TFA (0.136 mL, 1.83 mmol) followed by triethylsilane (0.059 mL, 0.366 mmol). The reaction was stirred at r.t. for 72 h. The reaction mixture was washed with sat. NaHCO_3_ solution, water and extracted with DCM. The solvent was removed and the crude reaction mixture was dissolved in MeOH (10 mL) and was hydrogenated over 10% Pd-C (0.20 g) at 55 psi for 72 h. The catalyst was filtered off and the filtrate concentrated to dryness to give pure **96** as a colorless, viscous oil which was used directly for the next step. 5-(4-chloro-2-methylphenyl)picolinic acid **83** (0.059 g, 0.25 mmol) in DMF (5 mL) was purged with nitrogen before DIPEA (0.054 g, 0.42 mmol) was added to the reaction mixture. HATU (0.103 g, 0.27 mmol) was added and stirred for 15 mins. (*S*)-5-Benzyl-8-(4-methylpiperazin-1-yl)-1,2,3,4-tetrahydronaphthalen-2-amine **96** (0.07 g, 0.21 mmol) was added to the reaction mixture and stirred at r.t. for 1.5 h. The reaction mixture was diluted with EtOAc and washed with water and 2M NaOH solution. The organic layer was dried over anhydrous Na_2_SO_4_ and filtered through a pad of Celite. The solvent was removed to give the crude product, which was purified by silica column chromatography using EtOAc/:MeOH (v/v=20%) as eluent to give **56** as a white foamy solid (0.025 g, 21%). HPLC 95.6%. mp 77-80°C. ^1^H NMR (CDCl_3_) δ 8.49 (dd, J = 2.1, 0.6 Hz, 1H), 8.28 (dd, J = 8.0, 0.6 Hz, 1H), 8.06 (d, J = 8.5 Hz, 1H), 7.79 (dd, J = 8.0, 2.2 Hz, 1H), 7.34-7.23 (m, 3H), 7.20-7.10 (m, 4H), 7.00-6.94 (m, 2H), 4.42-4.34 (m, 1H), 3.94 (s, 2H), 3.38 (dd, J = 16.4, 3.6 Hz, 1H), 3.02-2.95 (m, 2H), 2.91-2.75 (m, 4H), 2.70-2.52 (m, 5H), 2.35 (s, 3H), 2.26 (s, 3H), 2.23-2.15 (m, 1H), 1.88-1.78 (m, 1H). ^13^C NMR (CDCl_3_) δ 163.7, 150.3, 149.1, 148.3, 140.5, 139.1, 138.0, 137.7, 136.0, 135.3, 134.6, 134.3, 131., 130.8, 130.5, 128.9, 128.8, 128.6, 126.6, 126.1, 122.0, 117.5, 55.7, 52.1, 46.1, 45.4, 39.2, 32.2, 29.3, 25.9, 20.5. HRMS calcd. for C_35_H_37_ClN_4_O: 564.2656, found 564.2652.

*4.1.65. (S)-N-(5-Bromo-8-(4-methylpiperazin-1-yl)-1,2,3,4-tetrahydronaphthalen-2-yl)-5-(4-chloro-2-methylphenyl)picolinamide (****57****)*

A solution of **53** (0.048 g, 0.10 mmol) in DMF (5 mL) was cooled to 0 °C and NBS (0.023 g, 0.13 mmol) was added. The mixture was stirred at r.t. for 28 h, then diluted with EtOAc, washed with water and further extracted with EtOAc (3 X 20 mL). The combined organic layer was dried over anhydrous Na_2_SO_4_, filtered through Celite and evaporated, and the residue was purified by silica column chromatography using (EtOAc/MeOH (v/v=20%) to give **57** as a white foam (0.014 g, 25%). HPLC 95.4%. mp 121-124°C. ^1^H NMR (CDCl_3_) δ 8.49 (dd, J = 2.1, 0.7 Hz, 1H), 8.29 (dd, J = 8.0, 0.7 Hz, 1H), 8.08 (d, J = 8.3 Hz, 1H), 7.80 (dd, J = 8.0, 2.2 Hz, 1H), 7.43 (d, J = 8.5 Hz, 1H), 7.34-7.26 (m, 2H), 7.14 (d, J = 8.2 Hz, 1H), 6.89 (d, J = 8.6 Hz, 1H), 4.45-4.35 (m, 1H), 3.35 (dd, J = 16.3, 4.5 Hz, 1H), 3.15-2.62 (m, 11H), 2.49 (s, 3H), 2.26 (s, 3H), 2.23-2.20 (m, 1H), 1.96-1.89 (m, 1H). HRMS calcd. for C_28_H_30_BrClN_4_O: 552.1292, found 552.1291.

*4.1.66. (S)-5-(4-Chloro-2-methylphenyl)-N-(8-(4-methylpiperazin-1-yl)-5-nitro-1,2,3,4-tetrahydronaphthalen-2-yl)picolinamide (****58****)*

(*S*)-8-(4-Methylpiperazin-1-yl)-1,2,3,4-tetrahydronaphthalen-2-amine **82** (0.075 g, 0.31 mmol) was dissolved in conc. sulfuric acid (2 mL) and cooled to 0 °C. A 1:1 mixture of nitric acid (0.018 mL, 0.43 mmol, 69%) and sulfuric acid (0.018 mL) was added dropwise at 0 °C. The reaction was stirred at 0 °C for 45 min. The reaction was quenched with water and extracted with DCM. The organic layer was washed with sat. NaHCO_3_ solution and dried over anhydrous Na_2_SO_4_. The solvent was removed to give an inseparable mixture of **84** 5-NO_2_ and 7-NO_2_ isomers (1:2 ratio) which was used directly for the next step.

The title compound was obtained from crude mixture of (*S*)-8-(4-methylpiperazin-1-yl)-5-nitro-1,2,3,4-tetrahydronaphthalen-2-amine **84** and 5-(4-chloro-2-methylphenyl)picolinic acid **83** using the general procedure A to give **58** (15%) as a white foam. HPLC 97.3%. mp 106-109°C. ^1^H NMR (CDCl_3_) δ 8.49 (dd, J = 2.2, 0.8 Hz, 1H), 8.29 (dd, J = 8.0, 0.8 Hz, 1H), 8.10 (d, J = 8.4 Hz, 1H), 7.87 (d, J = 8.8 Hz, 1H), 7.80 (dd, J = 8.0, 2.2 Hz, 1H), 7.34-7.25 (m, 2H), 7.15 (d, J = 8.2 Hz, 1H), 7.03-6.99 (m, 1H), 4.45-4.38 (m, 1H), 3.38 (dd, J = 16.2, 4.1 Hz, 1H), 3.32-3.25 (m, 2H), 3.09-3.02 (m, 2H), 2.98-2.90 (m, 2H), 2.72-2.55 (m, 5H), 2.37 (s, 3H), 2.29-2.21 (m, 1H), 2.26 (s, 3H), 1.96-1.88 (m, 1H). HRMS calcd. for C_28_H_30_ClN_5_O_3_: 519.2037, found 519.2037.

*4.1.67. (S)-5-(4-Chloro-2-methylphenyl)-N-(5-((2-(dimethylamino)ethyl)amino)-8-(4- methylpiperazin-1-yl)-1,2,3,4-tetrahydronaphthalen-2-yl)picolinamide (****59****)*

A solution of **57** (0.062 g, 0.112 mmol) and *N,N*-dimethylethane-1,2-diamine (0.037 mL, 0.34 mmol) in toluene (5 mL) was purged with nitrogen for 5 min. XPhos (0.011 g, 0.022 mmol) and Pd_2_(dba)_3_ (0.01 g, 0.011 mmol) were added and the mixture was stirred at 100 °C for 30 min. Sodium tert-butoxide (0.022 g, 0.22 mmol) was then added and stirring was continued at 100 °C for 6 h. The cooled reaction mixture was diluted with EtOAc and washed with water, EtOAc and washed with water and extracted with EtOAc (3 X 20 mL). The combined organic layer was dried over anhydrous sNa_2_SO_4_, filtered through Celite and evaporated, The residue was purified by silica column chromatography using EtOAc/MeOH (v/v=20%) as eluent to give **59** as a yellow oil (0.008 g, 13%). HPLC 95.6%. ^1^H NMR (CDCl_3_) δ 8.48 (dd, J = 2.1, 0.7 Hz, 1H), 8.29 (dd, J = 8.0, 0.7 Hz, 1H), 8.08 (d, J = 8.5 Hz, 1H), 7.79 (dd, J = 8.0, 2.2 Hz, 1H), 7.33-7.25 (m, 2H), 7.14 (d, J = 8.2 Hz, 1H), 7.0 (d, J = 8.5 Hz, 1H), 6.51 (d, J = 8.6 Hz, 1H), 4.49-4.39 (m, 1H), 3.50 (s, 1H), 3.37 (dd, J = 16.5, 4.3 Hz, 1H), 3.15 (t, J = 6.0 Hz, 2H), 2.93-2.87 (m, 2H), 2.85-2.78 (m, 2H), 2.73-2.55 (m, 9H), 2.33 (s, 3H), 2.31-2.28 (m, 1H), 2.27 (s, 3H), 2.25 (s, 6H), 1.97-1.89 (m, 1H). HRMS calcd. for C_32_H_41_ClN_6_O: 560.3030, found 560.3025.

*4.1.68. (S)-N-(5,8-Bis(4-methylpiperazin-1-yl)-1,2,3,4-tetrahydronaphthalen-2-yl)-5-(4-chloro-2-methylphenyl)picolinamide (****60****)*

*Step i*. **89** (0.30 g, 0.579 mmol) in toluene (20 mL) was added palladium acetate (0.005 g, 0.023 mmol), BINAP (0.029 g, 0.046 mmol) and N-methylpiperazine (0.086 g, 0.869 mmol). The reaction mixture was heated at 100 °C for 3 h. The cooled reaction mixture was diluted with EtOAc and water. The pH of the mixture was adjusted to ~5 using AcOH (~ 0.3 mL) and was extracted with EtOAc. The aqueous layer was filtered through a plug of celite, and was hydrogenated over 10% Pd-C (0.20 g) at 55 psi for 43 h. The catalyst was filtered off and the filtrate concentrated to dryness to give pure **85** as a colorless, viscous oil which was used directly for the next step.

*Step ii*. The title compound was obtained from crude mixture of (*S*)-5,8-bis(4-methylpiperazin-1-yl)-1,2,3,4-tetrahydronaphthalen-2-amine **85** and 5-(4-chloro-2-methylphenyl)picolinic acid **83** using the general procedure A to give **60** (30%) as a white foam. HPLC 96.3%. mp 136-139°C. ^1^H NMR (CDCl_3_) δ 8.49 (dd, J = 2.2, 0.7 Hz, 1H), 8.30 (dd, J = 8.0, 0.7 Hz, 1H), 8.09 (d, J = 8.4 Hz, 1H), 7.80 (dd, J = 8.1, 2.3 Hz, 1H), 7.33-7.26 (m, 2H), 7.15 (d, J = 8.1 Hz, 1H), 7.03-6.98 (m, 2H), 4.49-4.38 (m, 1H), 3.38 (dd, J = 16.6, 4.8 Hz, 1H), 3.12-2.53 (m, 19H), 2.41 (s, 6H), 2.26 (s, 3H), 2.25-2.18 (m, 1H), 1.85-1.75 (m, 1H). HRMS calcd. for C_33_H_41_ClN_6_O: 572.3030, found 572.3030.

*4.1.69. (S)-5-(4-Chloro-2-methylphenyl)-N-(5-cyano-8-(4-methylpiperazin-1-yl)-1,2,3,4-tetrahydronaphthalen-2-yl)picolinamide (****61****)*

A solution of **57** (0.189 g, 0.34 mmol) in DMF (5 mL) was treated with Zn powder (0.002 g, 0.034 mmol), tris(dibenzylideneacetone)dipalladium (0.031 g, 0.034 mmol), tri(*o*-tolyl)phosphine (0.021 g, 0.068 mmol) and heated to 50 °C for 10 min. Zn(CN)_2_ (0.028 g, 0.24 mmol) was added and the reaction mixture was stirred at 50 °C for 1 h, then diluted with EtOAc and washed with water. The organic layer was dried over anhydrous Na_2_SO_4_, filtered through a pad of Celite and evaporated. The crude product, which was purified by silica column chromatography using EtOAc/MeOH (v/v=10%) to give **61** as a white solid (0.054 g, 32%). HPLC 96.4%. mp 132-135°C. ^1^H NMR (CDCl_3_) δ 8.49 (dd, J = 2.1, 0.7 Hz, 1H), 8.29 (dd, J = 8.0, 0.6 Hz, 1H), 8.10 (d, J = 8.4 Hz, 1H), 7.80 (dd, J = 8.1, 2.2 Hz, 1H), 7.51 (d, J = 8.3 Hz, 1H), 7.34-7.26 (m, 2H), 7.14 (d, J = 8.2 Hz, 1H), 6.95 (d, J = 8.3 Hz, 1H), 4.46-4.37 (m, 1H), 3.32-3.08 (m, 3H), 3.06-3.00 (m, 2H), 2.93-2.87 (m, 2H), 2.67-2.50 (m, 5H), 2.35 (s, 3H), 2.31-2.28 (m, 1H), 2.26 (s, 3H), 2.02-1.93 (m, 1H). HRMS calcd. for C_29_H_30_ClN_5_O: 499.2139, found 499.2141.

*4.1.70. General Procedure D. (S)-5-(4-Chloro-2-methylphenyl)-N-(5-methyl-8-(piperazin-1-yl)-1,2,3,4-tetrahydronaphthalen-2-yl)picolinamide (****62****)*

*Step i*. A solution of (*S*)-8-bromo-5-methyl-*N*-((*R*)-1-phenylethyl)-1,2,3,4-tetrahydronaphthalen-2-amine **97** (0.30 g, 0.788 mmol), *tert*-butyl piperazine-1-carboxylate **98** (0.40 g, 1.18 mmol), NaOtBu (0.151 g, 1.58 mmol) in toluene (10 mL) was flushed with N2 for 5 min. BINAP (0.104 g, 0.16 mmol) and Pd2dba3 (0.072 g, 0.079 mmol) was added and heated to 110 °C for 3 h. The catalyst was filtered off and the filtrate concentrated to dryness. The crude product, which was purified by silica column chromatography using EtOAc to give **99** (0.27 g, 76%) as a viscous oil which was hydrogenated over 10% Pd-C (0.20 g) at 55 psi for 24 h. The catalyst was filtered off and the filtrate concentrated to dryness to give pure **100** (53%) as a colorless, viscous oil which was used directly for the next step.

*Step ii*. The title compound was obtained from *tert-*butyl (*S*)-4-(7-amino-4-methyl-5,6,7,8-tetrahydronaphthalen-1-yl)piperazine-1-carboxylate **100** and 5-(4-chloro-2-methylphenyl)picolinic acid **83** using the general procedure A to give *N*-Boc **62** (53%) as a white foam. HPLC 99.1%. mp 163-164°C. ^1^H NMR (CDCl_3_) δ 8.49 (dd, J = 2.1, 0.6 Hz, 1H), 8.30 (dd, J = 8.0, 0.6 Hz, 1H), 8.08 (d, J = 8.4 Hz, 1H), 7.79 (dd, J = 8.0, 2.2 Hz, 1H), 7.34-7.25 (m, 2H), 7.14 (d, J = 8.2 Hz, 1H), 7.04 (d, J = 8.0 Hz, 1H), 6.86 (d, J = 8.0 Hz, 1H), 4.46-4.37 (m, 1H), 3.42 (dd, J = 16.6, 4.4 Hz, 1H), 2.90-2.70 (m, 10H), 2.71-2.62 (m, 1H), 2.31-2.25 (m, 1H), 2.26 (s, 3H), 2.22 (s, 3H), 1.95-1.85 (m, 1H) (NH not observed). HRMS calcd. for C_33_H_39_ClN_4_O_3_: 574.2711, found 574.2737.

A solution of carboxylate *N*-Boc **62** (0.10 g, 0.17 mmol) dissolved in DCM (3 mL) was added TFA (3 mL) dropwise. Reaction was stirred at r.t. for 1 h and solvent was removed. The reaction mixture was diluted with DCM and washed with 0.5 M NaOH solution and extracted with DCM (3 X 10 mL). The organic layer was dried over anhydrous Na_2_SO_4_ and filtered through a pad of Celite. The solvent was removed to give the crude product, which was purified by silica column chromatography using EtOAc/MeOH (v/v=10%) as eluent to give **62** as a white solid (0.089 g, 100%). HPLC 99.3%. mp 106-109 °C. ^1^H NMR (CDCl_3_) δ 8.49 (dd, J = 2.2, 0.8 Hz, 1H), 8.29 (dd, J = 8.0, 0.8 Hz, 1H), 8.08 (d, J = 8.4 Hz, 1H), 7.79 (dd, J = 8.0, 2.2 Hz, 1H), 7.33-7.25 (m, 2H), 7.15 (d, J = 8.2 Hz, 1H), 7.04 (d, J = 8.0 Hz, 1H), 6.91 (d, J = 8.0 Hz, 1H), 4.43-4.38 (m, 1H), 3.40 (dd, J = 16.5, 4.1 Hz, 1H), 3.04-2.93 (m, 4H), 2.92-2.80 (m, 4H), 2.80-2.73 (m, 2H), 2.71-2.63 (m, 1H), 2.31-2.26 (m, 1H), 2.26 (s, 3H), 2.21 (s, 3H), 1.95-1.85 (m, 1H) (NH not observed). HRMS calcd. for C_28_H_31_ClN_4_O. 474.2186, found 474.2201.

*4.1.71. 5-(4-Chloro-2-methylphenyl)-N-{(2S)-8-[4-(dimethylamino)-1-piperidinyl]-5-methyl-1,2,3,4-tetrahydro-2-naphthalenyl}-2-pyridinecarboxamide (****63****)*

**102** was obtained from (*S*)-8-bromo-5-methyl-1,2,3,4-tetrahydronaphthalen-2-amine **101** and 4-(dimethylamino)piperidine using the general procedure D to give **102** (76%) as an oil. ^1^H NMR (CDCl_3_) δ 6.98 (d, J = 8.0 Hz, 1H), 6.84 (d, J = 8.0 Hz, 1H), 3.22 (ddd, J = 16.2, 4.4, 2.0 Hz, 1H), 3.16-3.02 (m, 3H), 2.89-2.74 (m, 2H), 2.71-2.60 (m, 1H), 2.44 (m, 1H), 2.32 (s, 6H), 2.25 (m, 2H), 2.18 (s, 3H), 2.04 (m, 1H), 1.87 (m, 2H), 1.64 (m). LRMS Found: [M+H] =288.

The title compound was obtained from **102** and 5-(4-chloro-2-methylphenyl)picolinic acid **83** using the general procedure A to give **63** (86%) as a white foam. HPLC 95.0 %. ^1^H NMR (CDCl_3_) δ 8.49, (dd, J = 2.2, 0.7 Hz, 1H), 8.29 (dd, J = 8.7, 0.7 Hz, 1H), 8.08 (br d, J = 8.4 Hz, 1H), 7.79 (dd, J = 8.7, 2.2 Hz, 1H), 7.33-7.28 (m, 2H), 7.16 (d, J = 8.2 Hz, 1H), 7.03 (d, J = 8.0 Hz, 1H), 6.89 (d, J = 8.0 Hz, 1H), 4.42 (m, 1H), 3.37 (dd, J = 16.4, 4.0 Hz, 1H), 3.07 (m, 1H), 2.92-2.50 (m, 5H), 2.31 (s, 6H), 2.26 (s, 3H), 2.21 (s, 3H), 2.23 (m, 1H), 1.95 (m, 1H). ^13^C NMR (CDCl_3_) δ 163.8, 163.7, 150.4, 149.2, 149.1, 149.0, 148.3, 148.3, 139.2, 139.1, 138.0, 138.0, 137.7, 136.1, 136.0, 135.4, 135.3, 134.6, 134.5, 132.5, 131.8, 131.2, 130.8, 130.8, 130.2, 130.1, 128.1, 128.0, 126.6, 126.6, 122.0, 117.3, 117.2, 62.5, 56.1, 55.2, 52.8, 52.3, 52.1, 51.5, 45.6, 45.5, 41.7, 41.5, 32.1, 32.0, 29.4, 29.3, 29.3, 29.2, 29.0, 28.4, 28.1, 27.8, 26.3, 24.2, 20.5, 19.6, 19.6. HRMS calcd. for C_31_H_38_ClN_4_O. 517.2734, found 517.2730.

*4.1.72. 5-(4-Chloro-2-methylphenyl)-N-{(2S)-8-[3-(dimethylamino)-1-piperidinyl]-5-methyl-1,2,3,4-tetrahydro-2-naphthalenyl}-2-pyridinecarboxamide (****64****)*

**103** was obtained from (*S*)-8-bromo-5-methyl-1,2,3,4-tetrahydronaphthalen-2-amine **101** and 3-(dimethylamino)piperidine using the general procedure D to give **103** (87%) as an oil (1:1 mixture of diastereomers). ^1^H NMR (CDCl_3_) δ 6.99 (d, J = 8.0 Hz, 1H), 6.85 (d, J = 8.0 Hz, 1H), 3.26-3.15 (m, 2H), 3.13-3.02 (m, 1H), 3.01-2.89 (m, 1H), 2.85-2.76 (m, 1H), 2.63 (m, 2H), 2.46 (m, 1H), 2.32, 2.30 (2s, 6H), 2.18 (s, 3H), 2.03 (m, 2H), 1.87-1.49 (m), 1.28 (m, 1H). Found: [M+H]=288

The title compound was obtained from **103** and 5-(4-chloro-2-methylphenyl)picolinic acid **83** using the general procedure A to give **64** (78%) as a white foam (3:2 mixture of diastereomers). HPLC 94.7 %. 1H NMR (CDCl_3_) δ 8.49, (dd, J = 2.2, 0.7 Hz, 1H), 8.29 (dd, J = 8.7, 0.7 Hz, 1H), 8.10 (m, 1H), 7.79 (dd, J = 8.7, 2.2 Hz, 1H), 7.30 (dd, J = 14.0, 2.0 Hz,1H), 7.16 (dd, J = 8.2, 1.8 Hz, 1H), 7.03 (d, J = 8.0 Hz, 1H), 6.89 (d, J = 8.0 Hz, 1H), 4.42 (m, 1H), 3.37 (m, 1H), 3.20 (m,1H), 2.92-2.50 (m, 5H), 2.32 (s, NMe2), 2.29 (s, NMe2), 2.26 (s, 3H), 2.22 (s, 3H), 2.23 (m), 1.95 (m). HRMS calcd. for C_31_H_38_ClN_4_O. 517.2734, found 517.2746

*4.1.73. 5-(4-Chloro-2-methylphenyl)-N-[(2R)-5-methyl-8-(4-piperidinyl)-1,2,3,4-tetrahydro-2-naphthalenyl]-2-pyridinecarboxamide (****65****)*

**110** was obtained from **97** and *tert*-butyl 4-(4,4,5,5-tetramethyl-1,3,2-dioxaborolan-2-yl)-3,6-dihydropyridine-1(2*H*)-carboxylate **108** using the general procedure B to give **110** (74%) as an oil. This was immediately dissolved in MeOH (40 mL) and hydrogenated over 10% Pd-C (0.50 g) at 60 psi for 24 h. The catalyst was filtered off and the filtrate concentrated to dryness to give pure **112** as a colorless, viscous oil (100%). ^1^H NMR (CDCl_3_) δ 7.02 (d, J = 7.9 Hz, 1H), 6.97 (d, J = 7.9 Hz, 1H), 4.24 (br, 2H), 3.12 (m, 2H), 2.83 (m, 4H), 2.66 (m, 1H), 2.47 (dd, J = 15.4, 9.1 Hz, 1H), 2.20 (s, 3H), 2.06 (m, 1H), 1.61 (m), 1.48 (s, 9H), 1.45 (m, 1H) . LRMS Found: [M+H] =345.

The title compound was obtained from **112** and 5-(4-chloro-2-methylphenyl)picolinic acid **83** using the general procedure A to give **114** as a white foam followed by Boc group removal using *step ii* for preparation of **62** to give **65** (63%) as white foam. HPLC 97 %. ^1^H NMR (CDCl_3_) δ 8.49, (dd, J = 2.2, 0.7 Hz, 1H), 8.29 (dd, J = 8.7, 0.7 Hz, 1H), 8.13 (br d, J = 8.3 Hz, 1H), 7.79 (dd, J = 8.7, 2.2 Hz, 1H), 7.33-7.28 (m, 2H), 7.15 (d, J = 8.2 Hz, 1H), 7.09 (d, J = 7.9 Hz, 1H), 7.06 (d, J = 7.9 Hz, 1H), 4.48 (m, 1H), 3.31 (dd, J = 16.0, 5.2 Hz, 1H), 3.19 (m, 2H), 2.93-2.69 (m, 6H), 2.25 (s, 3H), 2.24 (s, 3H), 1.90 (m), 1.74 (m). HRMS calcd. for C_29_H_33_ClN_3_O 474.2307, found 474.2309.

*4.1.74. 5-(4-Chloro-2-methylphenyl)-N-[(2S)-5-methyl-8-(1-methyl-4-piperidinyl)-1,2,3,4-tetrahydro-2-naphthalenyl]-2-pyridinecarboxamide (****66****)*

**111** was obtained from **97** and 1-methyl-4-(4,4,5,5-tetramethyl-1,3,2-dioxaborolan-2-yl)-1,2,3,6-tetrahydropyridine **109** using the general procedure B to give **111** (84%) as an oil. This was immediately dissolved in MeOH (40 mL) and hydrogenated over 10% Pd-C (0.50 g) at 60 psi for 24 h. The catalyst was filtered off and the filtrate concentrated to dryness to give pure **113** (92%) as a colorless, viscous oil. ^1^H NMR (CDCl3) δ 7.06 (d, J = 7.8 Hz, 1H), 7.01 (d, J = 7.8 Hz, 1H), 3.17-2.98 (m, 4H), 2.82 (m, 1H), 2.69 (m, 2H), 2.44 (dd, J = 14.9, 8.6 Hz, 1H), 2.35 (s, 3H), 2.20 (s, 3H), 2.03 (m), 1.85 (m), 1.57 (m). Found: [M+H]= 259.

The title compound was obtained from **113** and 5-(4-chloro-2-methylphenyl)picolinic acid **83** using the general procedure A to give **66** (82%) as a white foam. HPLC 96 %. 1H NMR (CDCl_3_) δ 8.49, (dd, J = 2.1, 0.7 Hz, 1H), 8.29 (dd, J = 8.7, 0.7 Hz, 1H), 8.13 (br d, J = 8.2 Hz, 1H), 7.79 (dd, J = 8.7, 2.2 Hz, 1H), 7.33-7.27 (m, 2H), 7.15 (d, J = 8.2 Hz, 1H), 7.09 (d, J = 7.9 Hz, 1H), 7.06 (d, J = 7.9 Hz, 1H), 4.41 (m, 1H), 3.31 (dd, J = 16.0, 5.2 Hz, 1H), 3.19 (m, 2H), 2.93-2.63 (m, 6H), 2.30 (s, 3H), 2.26 (s, 3H), 2.25 (s, 3H), 2.02 (m,), 1.80 (m). HRMS calcd. for C_30_H_35_ClN_3_O 488.2463, found 488.2469.

*4.1.75. (S)-5-(4-Chloro-2-methylphenyl)-N-(5-methyl-8-(piperidin-1-yl)-1,2,3,4-tetrahydronaphthalen-2-yl)picolinamide (****67****)*

**104** was obtained from (*S*)-8-bromo-5-methyl-1,2,3,4-tetrahydronaphthalen-2-amine **101** and piperidine using the general procedure D to give **104** (47%) as white solid. ^1^H NMR ((CD_3_)_2_SO) δ 7.89 (d, J = 8.0 Hz, 2H), 7.41 (t, J = 8.0 Hz, 1H), 7.36 (d, J = 8.0 Hz, 2H), 6.95 (d, J = 8.01 Hz, 1H), 6.82 (d, J = 8.01 Hz, 1H), 3.21 (dd, J = 16.4, 2.5 Hz, 2H), 3.10 (m, 2H), 2.86 (m, 2H), 2.74 (m, 1H), 2.62 (m, 3H), 2.40 (m, 4H), 2.20 (s, 3H), 2.06 (m, 1H), 1.62 (m, 1H). Found: [M+H] =245.

The title compound was obtained from **104** and 5-(4-chloro-2-methylphenyl)picolinic acid **83** using the general procedure A to give **67** (84%) as a white solid. HPLC 95.0 %. mp 95-98 °C. ^1^H NMR (CDCl3) δ 8.49, (dd, J = 2.1, 0.7 Hz, 1H), 8.29 (dd, J = 8.7, 0.7 Hz, 1H), 8.09 (br d, J = 8.4 Hz, 1H), 7.79 (dd, J = 8.7, 2.2 Hz, 1H), 7.33-7.27 (m, 2H), 7.15 (d, J = 8.2 Hz, 1H), 7.03 (d, J = 8.0 Hz, 1H), 6.88 (d, J = 8.0 Hz, 1H), 4.41 (m, 1H), 3.35 (dd, J = 16.0, 5.2 Hz, 1H), 2.87-2.65 (m, 6H), 2.27 (s, 3H), 2.21 (s, 3H), 1.78 (m), 1.68 (m). HRMS calcd. for C_29_H_33_ClN_3_O 474.2307, found 474.2305.

*4.1.76. 5-(4-Chloro-2-methylphenyl)-N-[(2S)-5-methyl-8-(4-morpholinyl)-1,2,3,4-tetrahydro-2-naphthalenyl]-2-pyridinecarboxamide (****68****)*

**105** was obtained from (*S*)-8-bromo-5-methyl-1,2,3,4-tetrahydronaphthalen-2-amine **101** and morpholine using the general procedure D to give **105** (34%) as white crystals. ^1^H NMR ((CD_3_)_2_SO) δ 7.88 (d, J = 8.1 Hz, 2H), 7.41 (t, J = 8.1 Hz, 1H), 7.34 (t, J = 8.1 Hz, 2H), 6.98 (d, J = 8.0 Hz, 1H), 6.83 (d, J = 8.0 Hz, 1H), 3.68 (m, 4H), 3.24 (m, 1H), 3.14 (m, 1H), 2.85 (m, 2H), 2.76 (m, 1H), 2.61 (m, 3H), 2.43 (m, 1H), 2.12 (s, 3H), 2.07 (m, 1H), 1.65 (m, 1H). Found: [M+H] =247.

The title compound was obtained from **105** and 5-(4-chloro-2-methylphenyl)picolinic acid **83** using the general procedure A to give **68** (92%) as a white foam. HPLC 96 %. ^1^H NMR (CDCl_3_) δ 8.49, (dd, J = 2.1, 0.7 Hz, 1H), 8.29 (dd, J = 8.7, 0.7 Hz, 1H), 8.09 (br d, J = 8.4 Hz, 1H), 7.79 (dd, J = 8.7, 2.2 Hz, 1H), 7.34-7.27 (m, 2H), 7.15 (d, J = 8.2 Hz, 1H), 7.06 (d, J = 8.0 Hz, 1H), 6.92 (d, J = 8.0 Hz, 1H), 4.41 (m, 1H), 3.83 (m, 4H), 3.41 (dd, J = 15.8, 3.8 Hz, 1H), 2.97-2.77 (m, 6H), 2.67 (dd, J = 16.0, 9.2 Hz, 1H), 2.28 (m, 1H), 2.27 (s, 3H), 2.22 (s, 3H), 1.90 (m), 1.68 (m). HRMS calcd. for C_28_H_31_ClN_3_O_2_ 476.2099, found 476.2101.

*4.1.77. (S)-N-(8-(4-Aminophenyl)-5-methyl-1,2,3,4-tetrahydronaphthalen-2-yl)-5-(4-chloro-2-methylphenyl)picolinamide (****69****)*

**106** was obtained from (*S*)-8-bromo-5-methyl-1,2,3,4-tetrahydronaphthalen-2-amine **101** and 4-((tert-butoxycarbonyl)amino)phenyl)boronic acid using the general procedure D give **106** (64%) as white foam. ^1^H NMR (CDCl_3_) δ 7.29 (d, J = 8.4 Hz, 2H), 7.19 (d, J = 8.4 Hz, 2H), 7.06 (d, J = 7.6 Hz, 1H), 6.98 (d, J =7.6 Hz, 1H), 6.59 (br s, 1H), 3.16 (m, 1H), 2.96 (m, 1H), 2.86-2.52 (m, 4H), 2.32 (m), 2.27 (s, 3H), 1.86 (m, 1H), 1.52 (s, 9H). Found: [M+H]=353.

The title compound was obtained from **106** and 5-(4-chloro-2-methylphenyl)picolinic acid **83** using the general procedure A by Boc group removal using *step ii* for preparation of **62** to give **69** (32%) as white foam. HPLC 92.5 %. ^1^H NMR (CDCl_3_) δ 8.44, (dd, J = 2.2, 0.8 Hz, 1H), 8.22 (dd, J = 8.0, 0.8 Hz, 1H), 7.99 (br d, J = 8.3 Hz, 1H), 7.75 (dd, J = 8.7, 2.2 Hz, 1H), 7.32-7.27 (m, 2H), 7.13 (d, J = 8.1 Hz, 1H), 7.10-7.04 (m, 3H), 7.00 (d, J = 8.0 Hz, 1H), 6.70 (d, J = 6.5 Hz, 2H), 4.37 (m, 1H), 3.68 (br, 2H),) 3.06 (dd, J =16.5, 4.1 Hz, 1H), 2.98-2.70 (m, 2H), 2.71 (dd, J = 16.5, 9.6 Hz, 1H), 2.30 (s, 3H), 2.23 (s, 3H), 1.89 (m, 1H). HRMS calcd. for C_30_H_29_ClN_3_O 482.1994, found 482.2006.

*4.1.78. (S)-5-(4-Chloro-2-methylphenyl)-N-(8-(4-(dimethylamino)phenyl)-5-methyl-1,2,3,4-tetrahydronaphthalen-2-yl)picolinamide (****70****)*

**107** was obtained from (*S*)-8-bromo-5-methyl-1,2,3,4-tetrahydronaphthalen-2-amine **101** and 4-(dimethylamino)phenyl)boronic acid using the general procedure B to give **107** (81%) as white crystals. ^1^H NMR ((CD_3_)_2_SO) δ 7.07 (d, J = 8.8 Hz, 2H), 6.98 (d, J = 7.6 Hz, 1H), 6.84 (d, J = 7.6 Hz, 1H), 6.75 (d, J = 8.8 Hz, 2H), 3.30 (br, 2H), 2.92 (s, 6H), 2.78 (m, 2H), 2.67 (m, 2H), 2.36 (m, 1H), 2.18 (s, 3H), 1.94 (m, 1H), 1.47 (m, 1H). Found: [M+H] =281.

The title compound was obtained from **107** and 5-(4-chloro-2-methylphenyl)picolinic acid **83** using the general procedure A to give **70** (85%) as a white foam. HPLC 99%. ^1^H NMR (CDCl_3_) δ 8.44, (dd, J = 2.2, 0.8 Hz, 1H), 8.22 (dd, J = 8.0, 0.8 Hz, 1H), 7.99 (br d, J = 8.3 Hz, 1H), 7.74 (dd, J = 8.7, 2.2 Hz, 1H), 7.32-7.27 (m, 2H), 7.19-7.07 (m, 4H), 7.10-7.04 (m, 4H), 7.02 (d, J = 8.0 Hz, 1H), 6.75 (d, J = 6.5 Hz, 2H), 4.38 (m, 1H), 3.07 (dd, J = 16.5, 4.0 Hz, 1H), 2.98 (s, 6H), 2.95-2.76 (m, 1H), 2.75 (dd, J = 16.4, 9.5 Hz, 1H), 2.29 (s, 3H), 2.23 (s, 3H), 1.89 (m, 1H). HRMS calcd. for C_32_H_33_ClN_3_O 510.2307, found 510.2310.

*4.1.79. 5-(4-Chloro-2-methylphenyl)-N-[(2S)-5-methyl-8-(4-pyridinyl)-1,2,3,4-tetrahydro-2-naphthalenyl]-2-pyridinecarboxamide (****71****)*

**115** was obtained from (*S*)-8-bromo-5-methyl-1,2,3,4-tetrahydronaphthalen-2-amine **101** and pyridin-4-ylboronic acid using the general procedure B to give crude **115** which was used directly for the next step.

The title compound was obtained from **115** and 5-(4-chloro-2-methylphenyl)picolinic acid **83** using the general procedure A to give **71** (88%) as a white foam. HPLC 96%. ^1^H NMR (CDCl_3_) δ 8.61 (d, J = 5.9 Hz, 2H), 8.45, (dd, J = 2.2, 0.8 Hz, 1H), 8.22 (dd, J = 8.0, 0.8 Hz, 1H), 8.01 (br d, J = 8.4 Hz, 1H), 7.75 (dd, J = 8.6, 2.2 Hz, 1H), 7.32-7.28 (m, 2H), 7.23 (d, J = 5.9 Hz, 2H), 7.13 (m, 2H), 6.98 (d, J = 6.5 Hz, 1H), 4.38 (m, 1H), 3.02-2.82 (m, 3H), 2.70 (dd, J = 16.4, 6.4 Hz, 1H), 2.32 (s and m, 4H), 2.29 (m, 1H), 2.23 (s, 3H), 1.91 (m, 1H). ^13^C NMR (CDCl_3_) δ 163.7, 150.0, 149.8, 148.8, 148.3, 139.2, 138.0, 137.7, 137.7, 137.2, 136.0, 135.0, 134.6, 131.6, 131.2, 130.8, 128.0, 127.0, 126.6, 126.6, 124.7, 122.0, 45.6, 38.8, 35.3, 29.2, 26.4, 20.5, 20.1. HRMS calcd. for C_29_H_26_ClN_3_O 468.1854, found 468.1848

*4.1.80. 5-(4-Chloro-2-methylphenyl)-N-[(2S)-5-methyl-8-(3-pyridinyl)-1,2,3,4-tetrahydro-2-naphthalenyl]-2-pyridinecarboxamide (****72****)*

**116** was obtained from (*S*)-8-bromo-5-methyl-1,2,3,4-tetrahydronaphthalen-2-amine **101** and pyridin-3-ylboronic acid using the general procedure B to give **116** (65%) as an oil.  ^1^H NMR (CDCl_3_) δ 1.95 (m, 2H), 7.62 (m, 1H), 7.33 (m, 1H), 7.11 (d, J = 7.6 Hz, 1H), 6.99 (d, J = 7.6 Hz, 1H), 3.08 (m, 1H), 2.91 (m, 1H), 2.73 (m, 2H), 2.45 (m, 1H), 2.29 (s, 3H), 2.08 (m, 1H), 1.65 (m, 1H). Found: [M+H] =239.

The title compound was obtained from **116** and 5-(4-chloro-2-methylphenyl)picolinic acid **83** using the general procedure A to give **72** (62%) as a white foam. HPLC 91%.^1^H NMR (CDCl_3_+ drop of ((CD_3_)_2_SO)) δ 8.55 (m, 2H), 8.49, (br s, 1H), 8.27 (m, 2H), 7.78 (m, 1H), 7.65 (m, 1H), 7.39-7.30 (m, 2H), 7.27 (d, J = 7.6 Hz, 1H),7.14 (m, 2H), 7.00 (d, J = 7.6 Hz, 1H), 4.35 (br, 1H), 3.02-2.80 (m), 2.75 (dd, J = 16.4, 9.8 Hz, 1H), 2.32 (s, 3H), 2.29 (m, 1H), 2.25 (s, 3H), 1.98 (m, 1H). ^13^C NMR (CDCl_3_) δ 163.7, 150.2, 148.8, 148.3, 148.3, 139.2, 138.0, 137.7, 137.0, 136.8, 136.7, 136.0, 135.0, 134.6, 132.3, 131.2, 130.8, 128.0, 127.8, 126.6, 123.3, 122.0, 45.6, 35.5, 29.2, 26.3, 20.5, 20.1. HRMS calcd. for C_29_H_26_ClN_3_O 468.1854, found 468.1856.

*4.1.81. 5-(4-Chloro-2-methylphenyl)-N-[(2S)-8-(6-methoxy-3-pyridinyl)-5-methyl-1,2,3,4-tetrahydro-2-naphthalenyl]-2-pyridinecarboxamide (****73****)*

**117** was obtained from (*S*)-8-bromo-5-methyl-1,2,3,4-tetrahydronaphthalen-2-amine **101** and (6-methoxypyridin-3-yl)boronic acid using the general procedure B to give **117** (65%) as an oil.  ^1^H NMR (CDCl_3_) δ 8.09 (dd, J = 2.4, 0.5 Hz, 1H), 7.51 (dd, J = 5.5, 2.4 Hz, 1H), 7.08 (d, J = 7.6 Hz, 1H), 6.97 (d, J = 7.6 Hz, 1H), 6.78 (dd, *J* = 5.5, 0.5 Hz, 1H), 3.97 (s, 3H), 3.09 (m, 1H), 2.90 (m, 1H), 2.80 (m, 1H), 2.71 (m, 1H), 2.48 (dd, J = 16.2, 6.8 Hz, 1H), 2.27 (s, 3H), 2.09 (m, 1H), 1.67 (m, 1H). Found: [M+H] =269.

The title compound was obtained from **117** and 5-(4-chloro-2-methylphenyl)picolinic acid **83** using the general procedure A to give **73** (80%) as a white foam. HPLC 96%. ^1^H NMR (CDCl_3_) δ 8.45 (dd, J = 2.08, 0.6 Hz, 1H), 8.22, (dd, J = 8.0, 0.6 Hz, 1H), 8.09 (d, J = 2.0 Hz, 1H), 8.01 (br d, J = 8.4 Hz, 1H), 7.76 (dd, J = 8.0, 2.2 Hz, 1H), 7.51 (dd, J = 8.5, 2.4 Hz, 1H), 7.32 (d, J = 1.9 Hz, 1H), 7.13 (m, 2H), 6.99 (d, J = 7.6 Hz, 1H), 6.77 (dd, J = 8.4, 0.4 Hz, 1H), 4.36 (m, 1H), 3.96 (s, 3H), 3.04-2.82 (m, 3H), 2.70 (dd, J = 16.4, 6.4 Hz, 1H), 2.31 (s and m, 4H), 2.23 (s, 3H), 1.91 (m, 1H). HRMS calcd. for C_30_H_29_ClN_3_O_2_ 498.1943, found 498.1967.

*4.1.82. (S)-5-(4-Chloro-2-methylphenyl)-N-(8-(6-(dimethylamino)pyridin-3-yl)-5-methyl-1,2,3,4-tetrahydronaphthalen-2-yl)picolinamide) (****74****)*

**118** was obtained from (*S*)-8-bromo-5-methyl-1,2,3,4-tetrahydronaphthalen-2-amine **101** and (6-(dimethylamino)pyridin-3-yl)boronic acid using the general procedure B to give **118** (68%) as an oil.  ^1^H NMR (CDCl_3_) δ 8.11 (dd, J = 2.4, 0.5 Hz, 1H), 7.41 (dd, J = 8.7, 2.4 Hz, 1H), 7.07 (d, J = 7.6 Hz, 1H), 6.98 (d, J = 7.6 Hz, 1H), 6.56 (dd, J = 8.7, 0.5 Hz, 1H), 3.12 (s, 3H), 3.01-2.79 (m, 4H), 2.69 (m, 2H), 2.50 (m,1H), 2.27 (s, 3H), 2.19 (m), 1.61 (m). Found: [M+H] =282.

The title compound was obtained from **118** and 5-(4-chloro-2-methylphenyl)picolinic acid **83** using the general procedure A to give **74** (63%) as a white foam. HPLC 96%. ^1^H NMR (CDCl_3_) δ 8.45 (dd, J = 2.08, 0.6 Hz, 1H), 8.22, (dd, J = 8.0, 0.6 Hz, 1H), 8.12 (d, J = 2.0 Hz, 1H), 8.01 (br d, J = 8.4 Hz, 1H), 7.75 (dd, J = 8.0, 2.2 Hz, 1H), 7.41 (dd, J = 8.7, 2.4 Hz, 1H), 7.28 (d, J = 1.9 Hz, 1H), 7.12 (m, 2H), 70.0 (d, J = 7.6 Hz, 1H), 6.55 (d, J = 8.7 Hz, 1H), 4.38 (m, 1H), 3.11 (s, 6H), 3.07 (dd, J = 16.5, 4.2 Hz, 1H), 2.99-2.87 (m, 2H), 2.73 (dd, J = 16.4, 6.4 Hz, 1H), 2.30 (s and m, 4H), 2.23 (s, 3H), 1.90 (m, 1H). HRMS calcd. for C_31_H_32_ClN_4_O 511.2259, found 511.2271.

*4.1.83. (S)-5-(4-Chloro-2-methylphenyl)-N-(8-(2-methoxypyrimidin-5-yl)-5-methyl-1,2,3,4-tetrahydronaphthalen-2-yl)picolinamide (****75****)*

**124** was obtained from **97** and (2-methoxypyrimidin-5-yl)boronic acid **119** using the general procedure B to give **124** (35%) as a crude product. This was immediately dissolved in MeOH (40 mL) and hydrogenated over 10% Pd-C (0.30 g) at 60 psi for 72 h. The catalyst was filtered off and the filtrate concentrated to dryness to give pure **129** as a colorless, viscous oil which was used directly for the next step.

The title compound was obtained from **129** and 5-(4-chloro-2-methylphenyl)picolinic acid **83** using the general procedure A to give **75** (22%) as a white foam. HPLC 98.3%. mp 91-94°C. ^1^H NMR (CDCl_3_) δ 8.48-8.45 (m, 3H), 8.22 (dd, J = 8.0, 0.7 Hz, 1H), 8.03 (d, J = 8.4 Hz, 1H), 7.77 (dd, J = 8.0, 2.2 Hz, 1H), 7.32-7.28 (m, 2H), 7.17-7.12 (m, 2H), 6.99 (d, J = 7.6 Hz, 1H), 4.41-4.35 (m, 1H), 4.03 (s, 3H), 3.02-2.85 (m, 3H), 2.73-2.66 (m, 1H), 2.35-2.28 (m, 1H), 2.32 (s, 3H), 2.24 (s, 3H), 1.97-1.87 (m, 1H).. HRMS calcd. for C_29_H_27_ClN_4_O_2_: 498.1823, found 498.1850.

*4.1.84. (S)-5-(4-Chloro-2-methylphenyl)-N-(8-(2-(dimethylamino)pyrimidin-5-yl)-5-methyl-1,2,3,4-tetrahydronaphthalen-2-yl)picolinamide (****76****)*

**125** was obtained from **97** and (2-(dimethylamino)pyrimidin-5-yl)boronic acid **120** using the general procedure B to give **125** (51%) as a white solid. ^1^H NMR (CDCl_3_) δ 8.21 (s, 2H), 7.27-7.21 (m, 4H), 7.18-7.13 (m, 1H), 7.03 (d, J = 7.6 Hz, 1H), 6.89 (d, J = 7.6 Hz, 1H), 3.98 (q, J = 6.6 Hz, 1H), 3.26 (s, 6H), 2.87-2.79 (m, 1H), 2.67-2.40 (m, 4H), 2.22 (s, 3H), 2.20-2.17 (m, 1H), 1.61-1.51 (m, 1H), 1.43-1.36 (m, 1H), 1.32 (d, J = 6.6 Hz, 3H). LRMS Found: [M+H]= 387.3.

This was immediately dissolved in MeOH (40 mL) and hydrogenated over 10% Pd-C (0.30 g) at 60 psi for 72 h. The catalyst was filtered off and the filtrate concentrated to dryness to give pure **130** as a colorless, viscous oil which was used directly for the next step.

The title compound was obtained from **130** and 5-(4-chloro-2-methylphenyl)picolinic acid **83** using the general procedure A to give **76** (53%) as a white foam. HPLC 96.0%. mp 107-110°C. ^1^H NMR (CDCl_3_) δ 8.46 (d, J = 1.5 Hz, 1H), 8.28 (s, 2H), 8.22 (dd, J = 8.0, 0.5 Hz, 1H), 8.02 (d, J = 8.2 Hz, 1H), 7.76 (dd, J = 8.0, 2.2 Hz, 1H), 7.32-7.25 (m, 2H), 7.15-7.11 (m, 2H), 6.99 (d, J = 7.6 Hz, 1H), 4.41-4.32 (m, 1H), 3.21 (s, 6H), 3.05 (dd, J = 17.0, 4.5 Hz, 1H), 2.99-2.70 (m, 3H), 2.35-2.28 (m, 1H), 2.31 (s, 3H), 2.26 (s, 3H), 1.96-1.86 (m, 1H). LRMS Found: [M+H]= 512.3. HRMS calcd. for C_30_H_30_ClN_5_O: 511.2139, found 511.2163.

*4.1.85. (S)-5-(4-Chloro-2-methylphenyl)-N-(5-methyl-8-(1H-pyrazol-5-yl)-1,2,3,4-tetrahydronaphthalen-2-yl)picolinamide (****77****)*

**126** was obtained from **97** and (1*H*-pyrazol-5-yl)boronic acid **121** using the general procedure B to give **126** (78%) as a crude product. This was immediately dissolved in MeOH (40 mL) and hydrogenated over 10% Pd-C (0.30 g) at 60 psi for 72 h. The catalyst was filtered off and the filtrate concentrated to dryness to give pure **131** as a colorless, viscous oil which was used directly for the next step.

The title compound was obtained from **131** and 5-(4-chloro-2-methylphenyl)picolinic acid **83** using the general procedure A to give **77** (46%) as a white foam. HPLC 98.7%. mp 147-150°C. ^1^H NMR (CDCl_3_) δ 8.45 (d, J = 1.5 Hz, 1H), 8.23 (d, J = 8.1 Hz, 1H), 8.06 (d, J = 8.1 Hz, 1H), 7.77 (dd, J = 8.0, 2.1 Hz, 1H), 7.60 (d, J = 2.0 Hz, 1H), 7.33-7.24 (m, 2H), 7.22 (d, J = 7.7 Hz, 1H), 7.17-7.10 (m, 2H), 6.38 (d, J = 1.9 Hz, 1H), 4.45-4.36 (m, 1H), 3.26 (dd, J = 16.4, 4.2 Hz, 1H), 2.97-2.82 (m, 3H), 2.33-2.25 (m, 1H), 2.30 (s, 3H), 2.23 (s, 3H), 1.98-1.88 (m, 1H) (NH not observed). ^13^C NMR (CDCl_3_) δ 163.8, 148.8, 148.2, 139.1, 138.0, 137.7, 137.2, 136.0, 135.0, 134.5, 132.9, 131.2, 130.8, 129.8, 127.9, 127.4, 126.6, 122.0, 106.1, 45.6, 35.1, 28.9, 26.1, 20.5, 20.1. LRMS Found: [M+H]= 457.2. HRMS calcd. for C_27_H_25_ClN_4_O (M+H^+^) *m/z*: 456.1717 found 456.1730.

*4.1.86. (S)-5-(4-Chloro-2-methylphenyl)-N-(5-methyl-8-(1-methyl-1H-pyrazol-5-yl)-1,2,3,4-tetrahydronaphthalen-2-yl)picolinamide (****78****)*

**127** was obtained from **97** and (1-methyl-1*H*-pyrazol-5-yl)boronic acid **122** using the general procedure B to give **127** (75%) as a crude product. This was immediately dissolved in MeOH (40 mL) and hydrogenated over 10% Pd-C (0.30 g) at 60 psi for 72 h. The catalyst was filtered off and the filtrate concentrated to dryness to give pure **132** as a colorless, viscous oil which was used directly for the next step.

The title compound was obtained from **132** and 5-(4-chloro-2-methylphenyl)picolinic acid **83** using the general procedure A to give **78** (10%) as a white foam. HPLC 99.6%. mp 161-163°C. ^1^H NMR (CDCl_3_) δ 8.45 (dd, J = 2.1, 0.8 Hz, 1H), 8.23 (dd, J = 8.0, 0.7 Hz, 1H), 7.99 (d, J = 8.4 Hz, 1H), 7.77 (dd, J = 8.0, 2.2 Hz, 1H), 7.51 (d, J = 1.8 Hz, 1H), 7.33-7.24 (m, 2H), 7.17-7.10 (m, 2H), 7.01 (d, J = 7.7 Hz, 1H), 6.17 (d, J = 1.9 Hz, 1H), 4.42-4.34 (m, 1H), 3.66 (s, 3H), 2.99-2.83 (m, 3H), 2.57-2.48 (m, 1H), 2.36-2.29 (m, 1H), 2.32 (s, 3H), 2.24 (s, 3H), 1.92-1.83 (m, 1H). LRMS Found: [M+H]= 471.2. HRMS calcd. for C_28_H_27_ClN_4_O 471.1946 found 471.1930.

*4.1.87. (S)-5-(4-Chloro-2-methylphenyl)-N-(5-methyl-8-(1-methyl-1H-pyrazol-4-yl)-1,2,3,4-tetrahydronaphthalen-2-yl)picolinamide (****79****)*

**128** was obtained from **97** and (1-methyl-1*H*-pyrazol-4-yl)boronic acid **123** using the general procedure B to give **128** (43%) as a crude product. This was immediately dissolved in MeOH (40 mL) and hydrogenated over 10% Pd-C (0.30 g) at 60 psi for 72 h. The catalyst was filtered off and the filtrate concentrated to dryness to give pure **133** as a colorless, viscous oil which was used directly for the next step.

The title compound was obtained from **133** and 5-(4-chloro-2-methylphenyl)picolinic acid **83** using the general procedure A to give **79** (74%) as a white foam. HPLC 98.7%. mp 76-79°C. ^1^H NMR (CDCl_3_) δ 8.47 (dd, J = 2.1, 0.7 Hz, 1H), 8.24 (dd, J = 8.0, 0.6 Hz, 1H), 8.07 (d, J = 8.3 Hz, 1H), 7.77 (dd, J = 8.0, 2.2 Hz, 1H), 7.52 (d, J = 0.5 Hz, 1H), 7.38 (s, 1H), 7.33-7.26 (m, 2H), 7.14 (d, J = 8.2 Hz, 1H), 7.11-7.05 (m, 2H), 4.45-4.36 (m, 1H), 3.93 (s, 3H), 3.23 (dd, J = 16.0, 4.1 Hz, 1H), 2.93-2.76 (m, 3H), 2.33-2.26 (m, 1H), 2.29 (s, 3H), 2.24 (s, 3H), 1.98-1.88 (m, 1H). ^13^C NMR (CDCl_3_) δ 163.7, 148.9, 148.3, 139.4, 139.2, 138.0, 137.7, 136.0, 135.6, 134.8, 134.6, 132.3, 131.2, 130.9, 130.8, 129.1, 127.8, 127.5, 126.7, 122.0, 121.9, 45.7, 39.2, 35.6, 29.0, 26.2, 20.5, 20.0. LRMS Found: [M+H]= 471.1. HRMS calcd. for C_28_H_27_ClN_4_O: 470.1873, found 470.1885.

**^1^H and ^13^C NMR Spectra** **(for compounds that progressed to advanced testing):**

Compound **5**

Compound **5R**


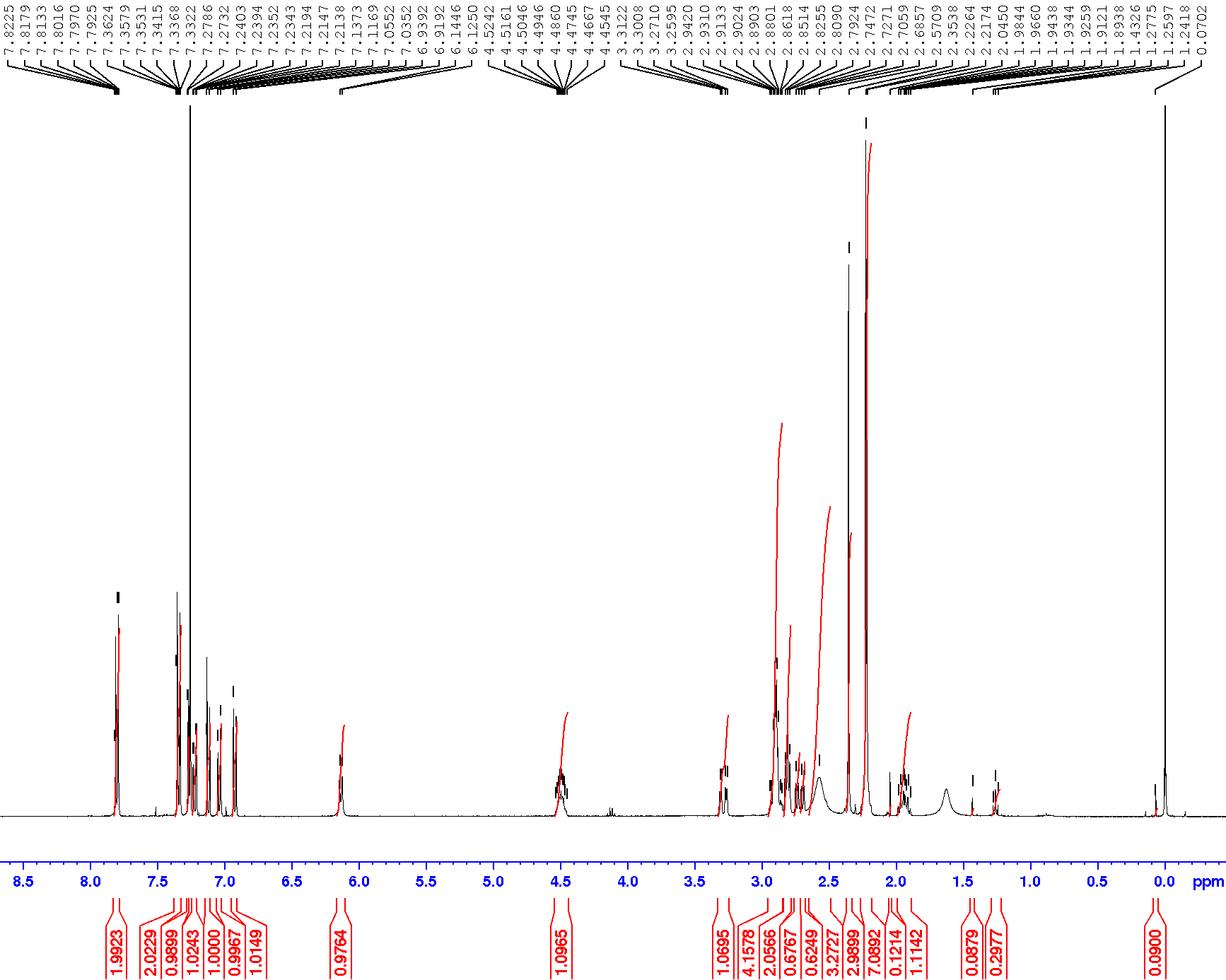


Compound **6**


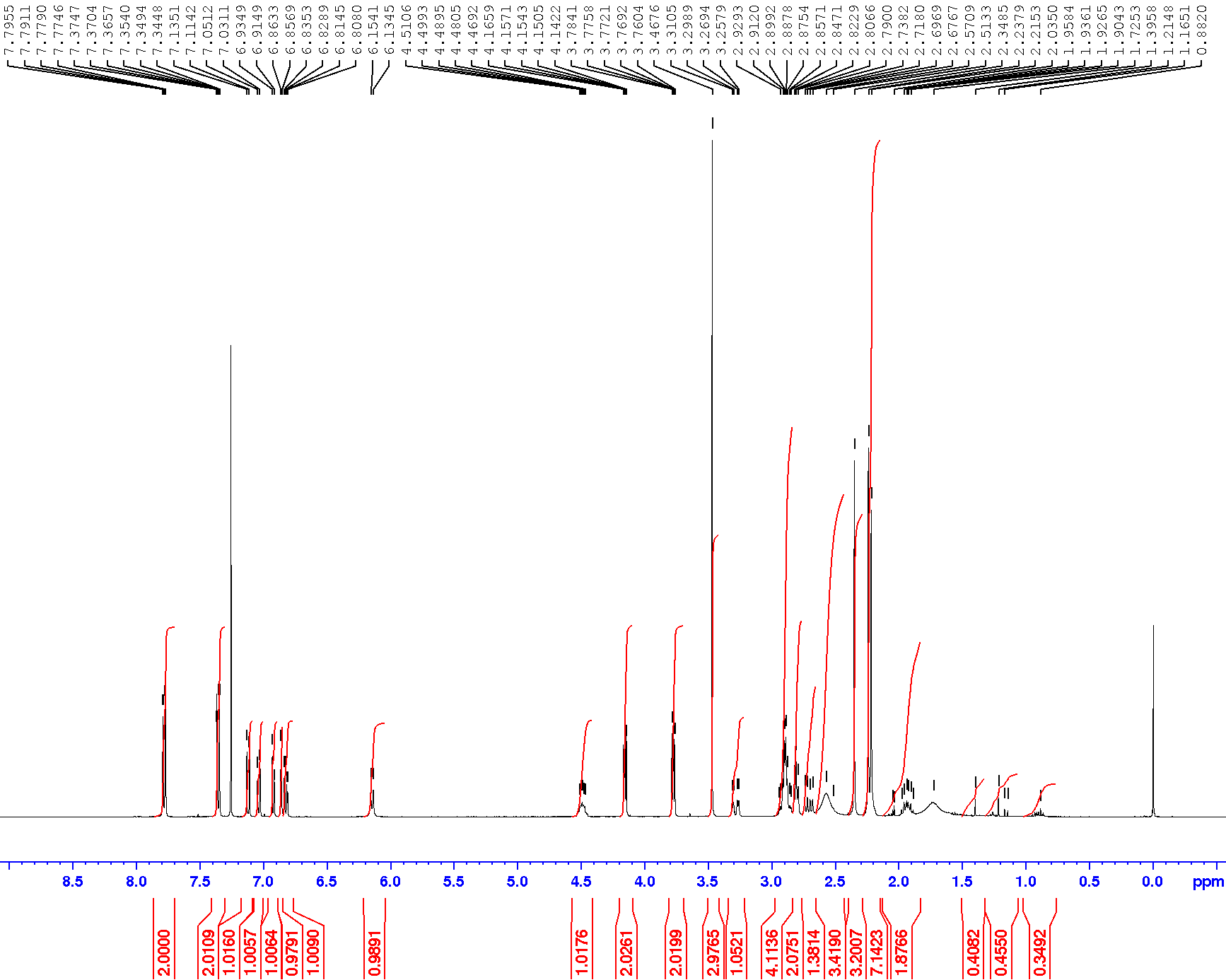

Compound **7**


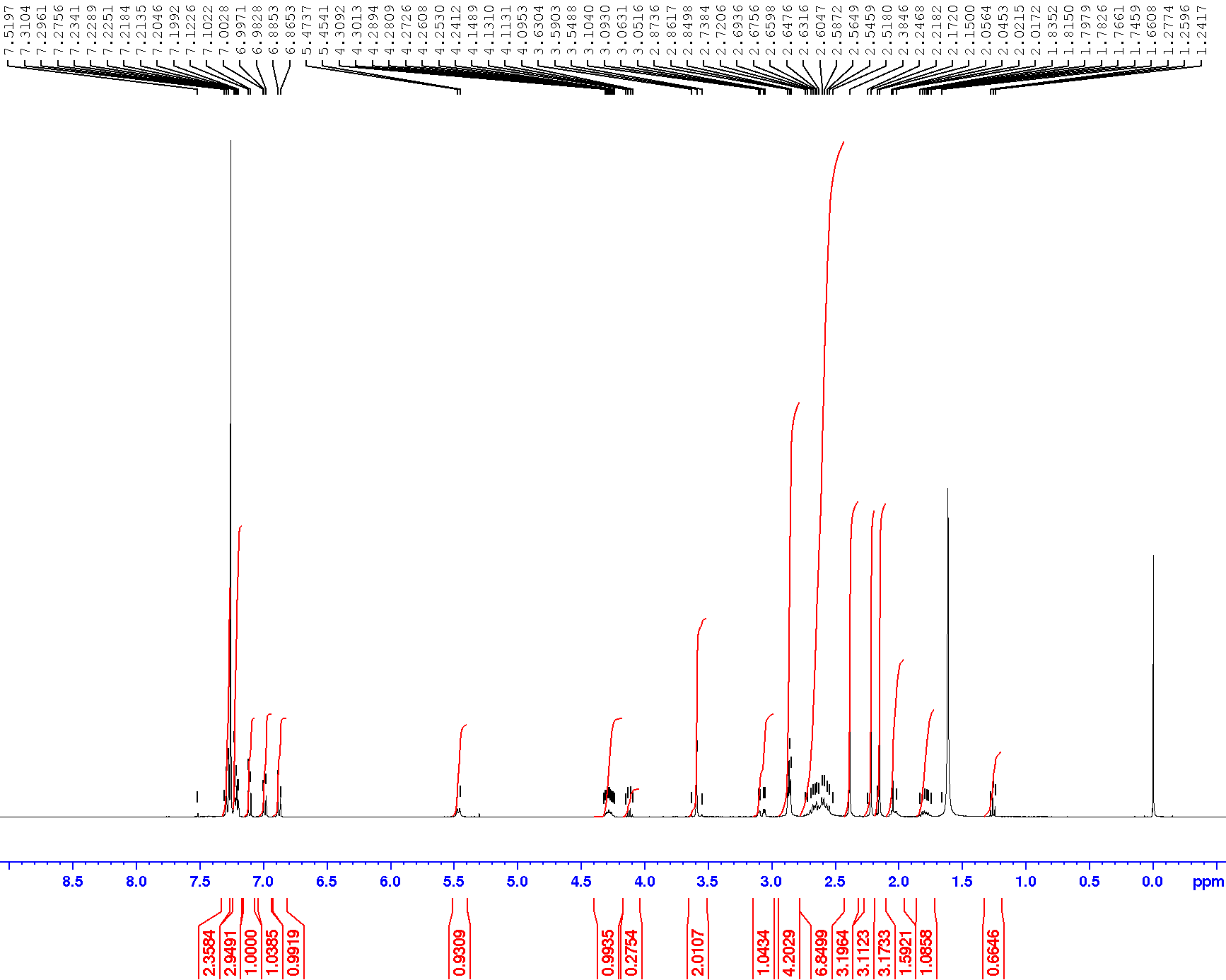


Compound **8**


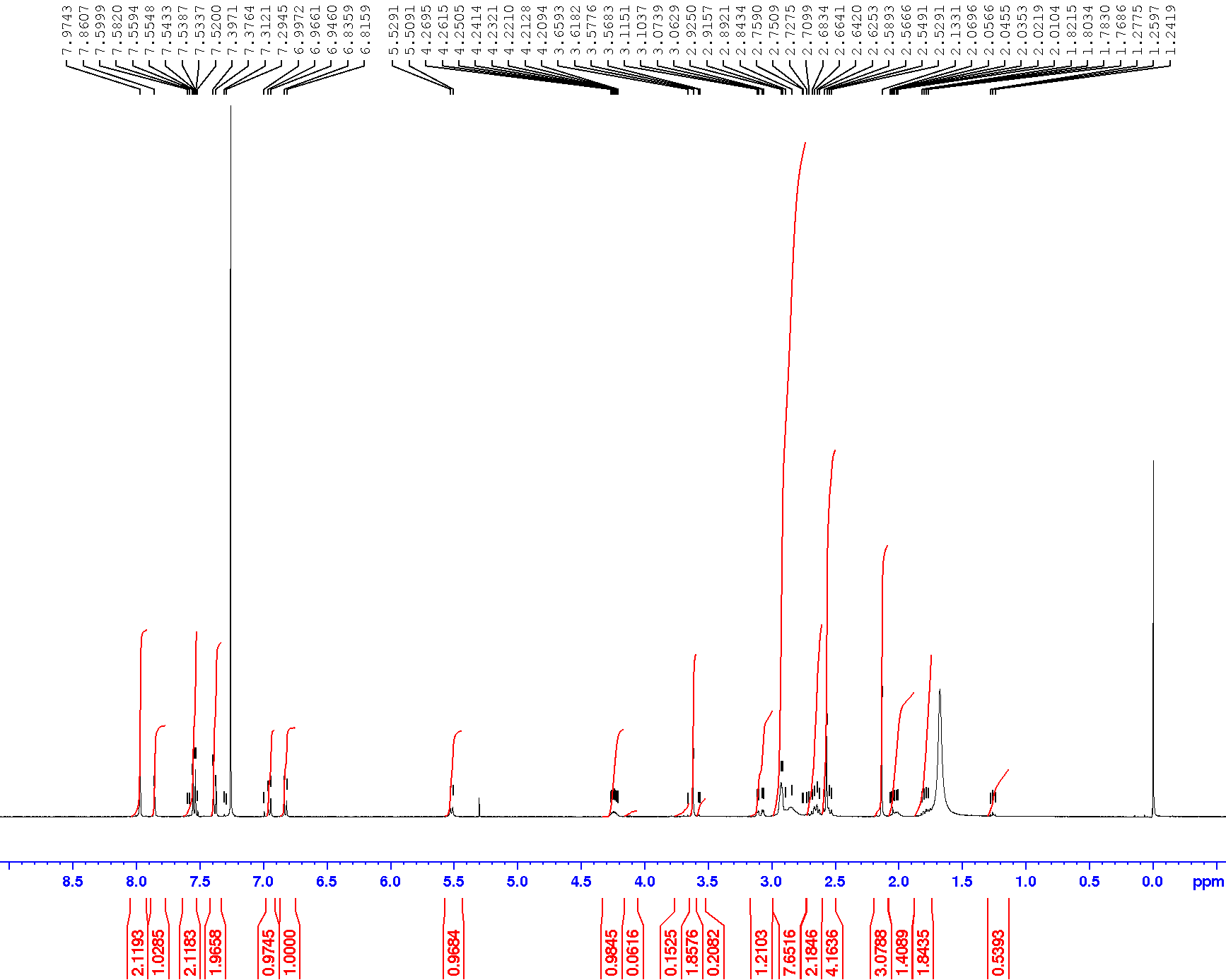


Compound **9**


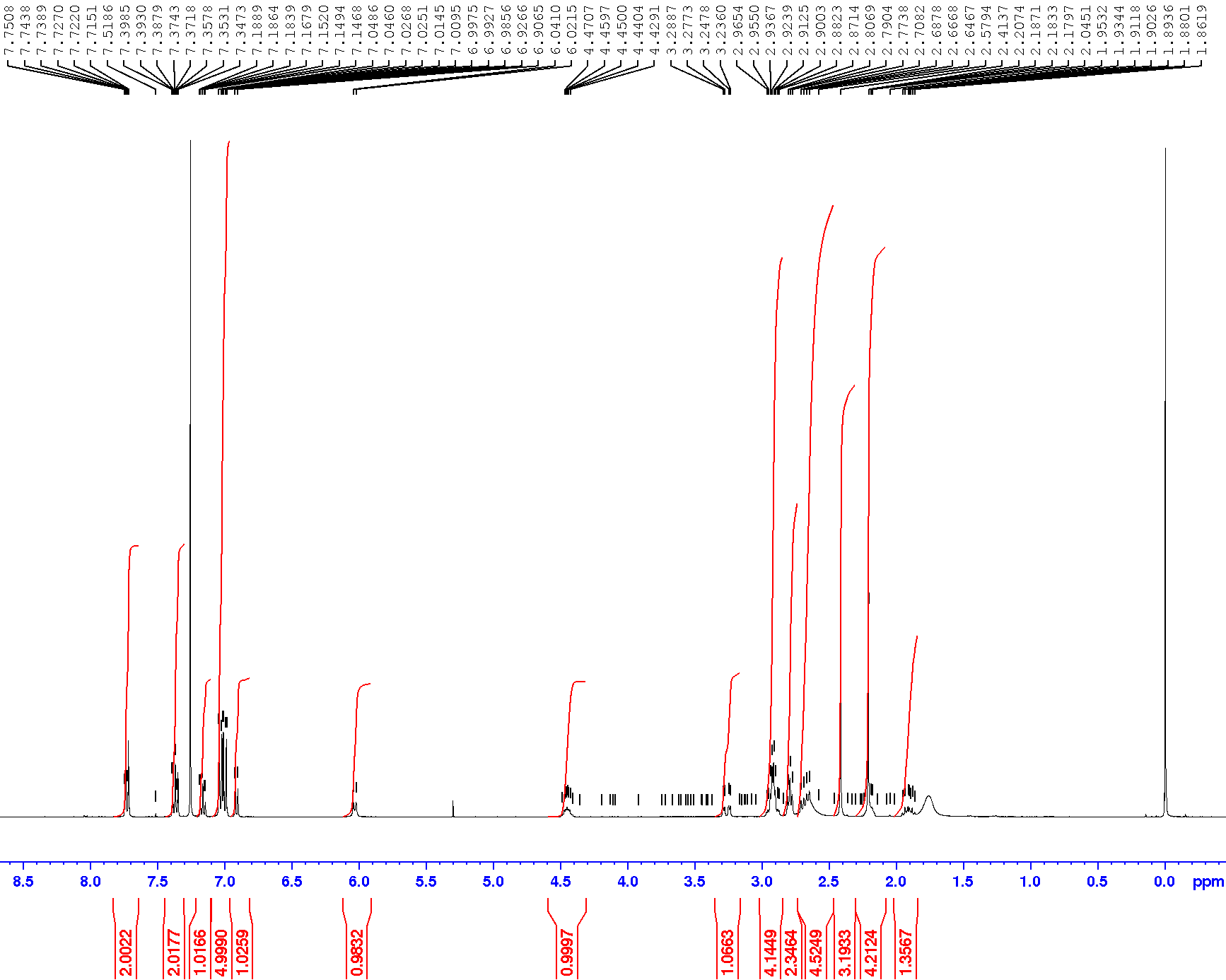


Compound **10**


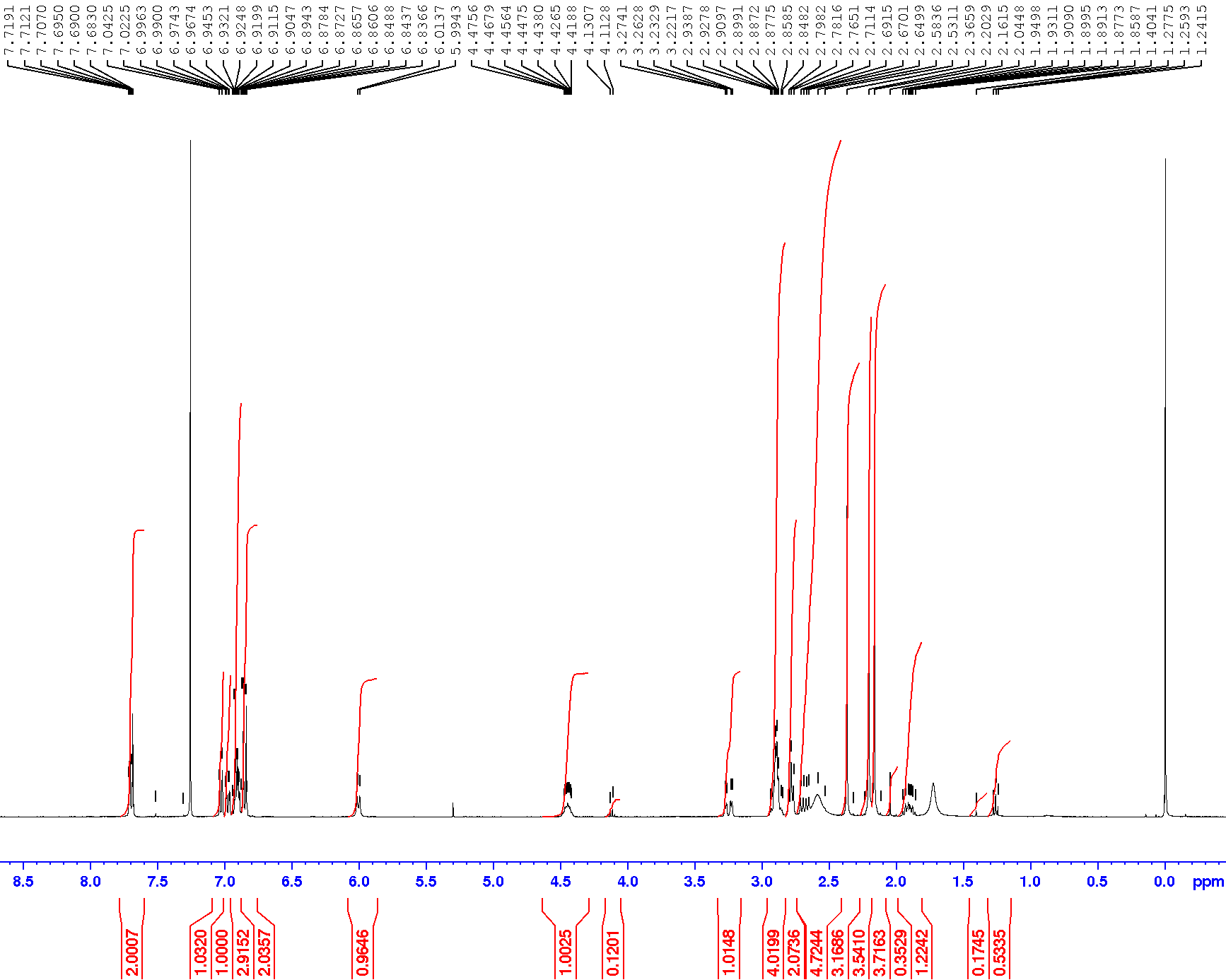


Compound **11**


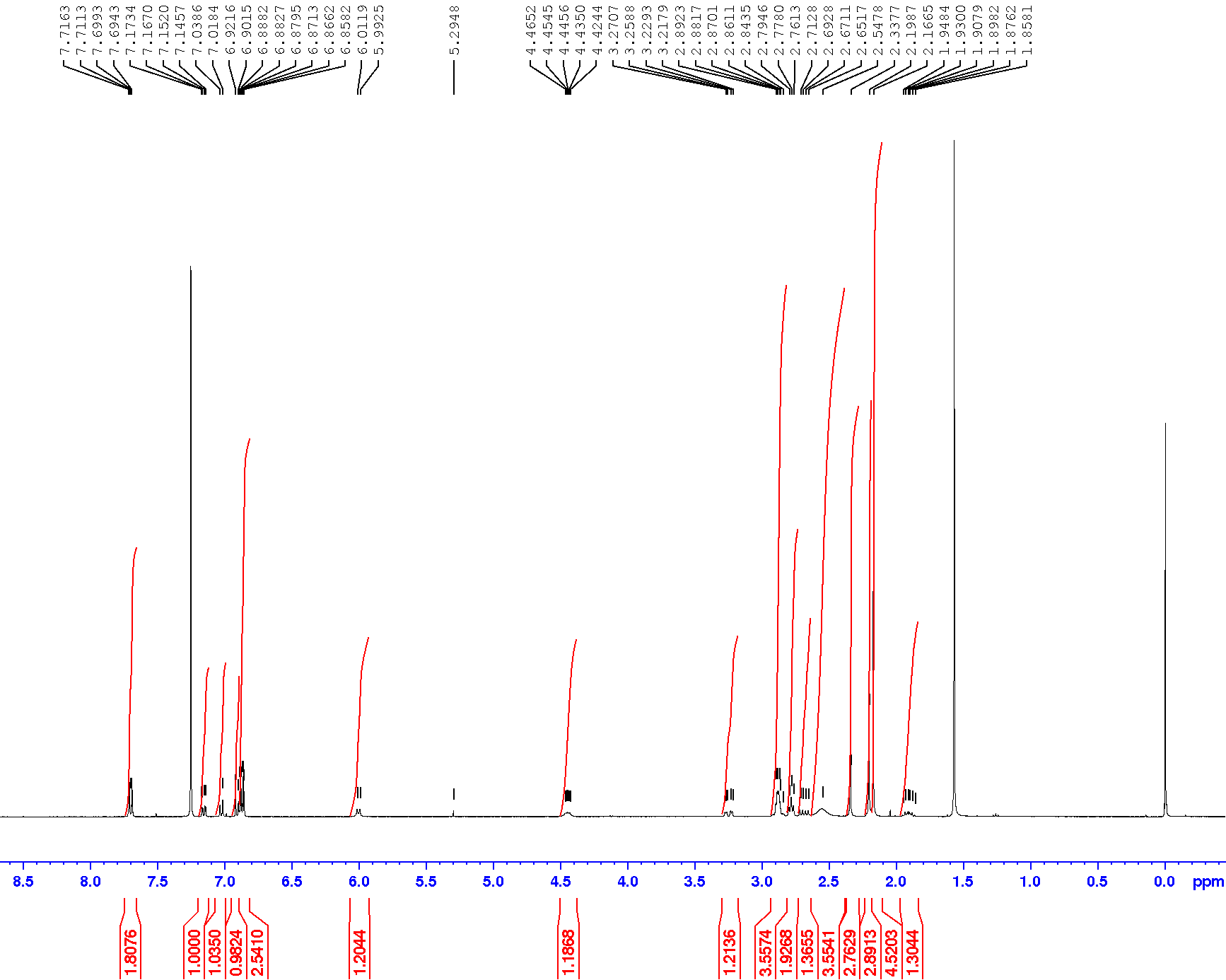


Compound **12**


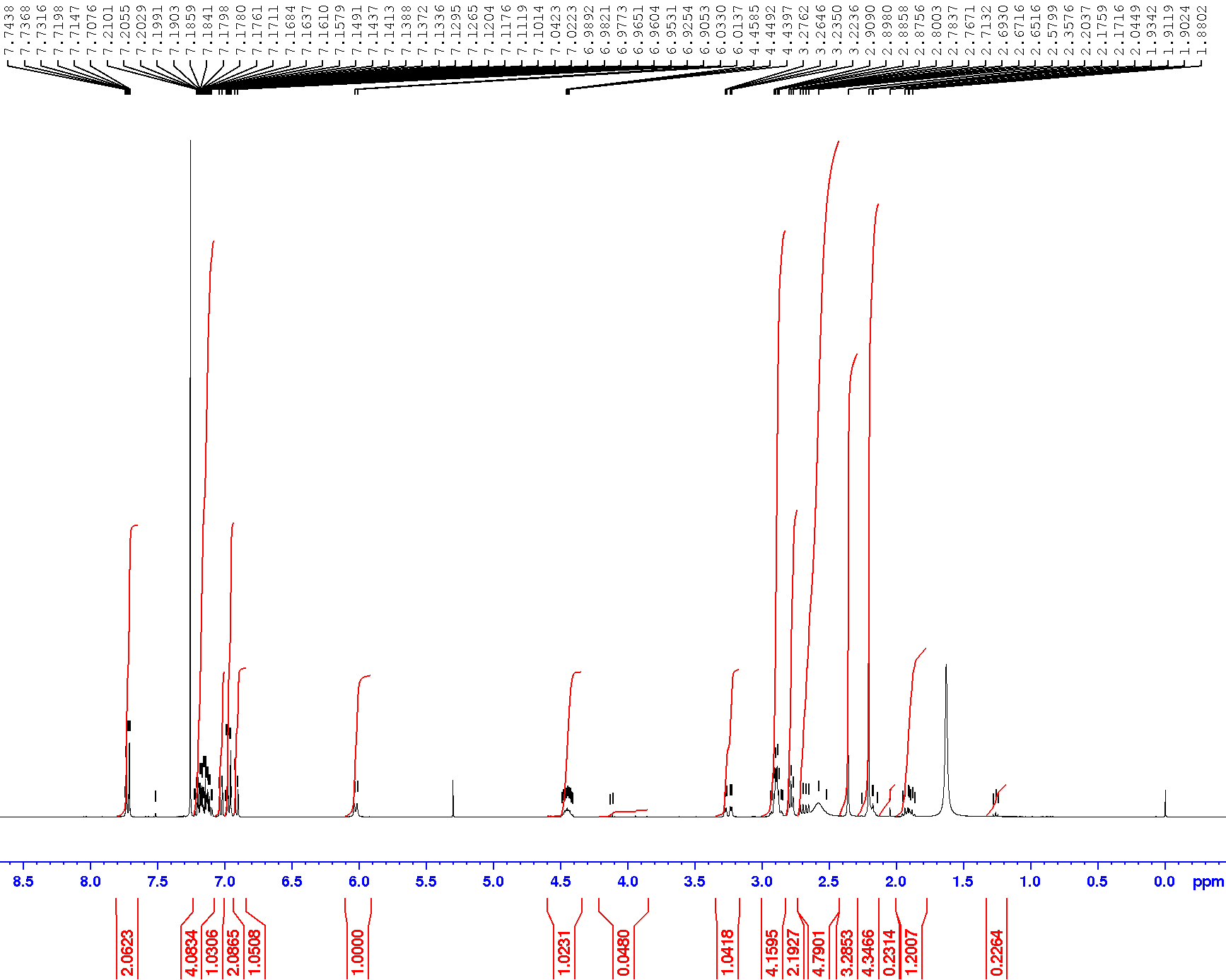


Compound **13**


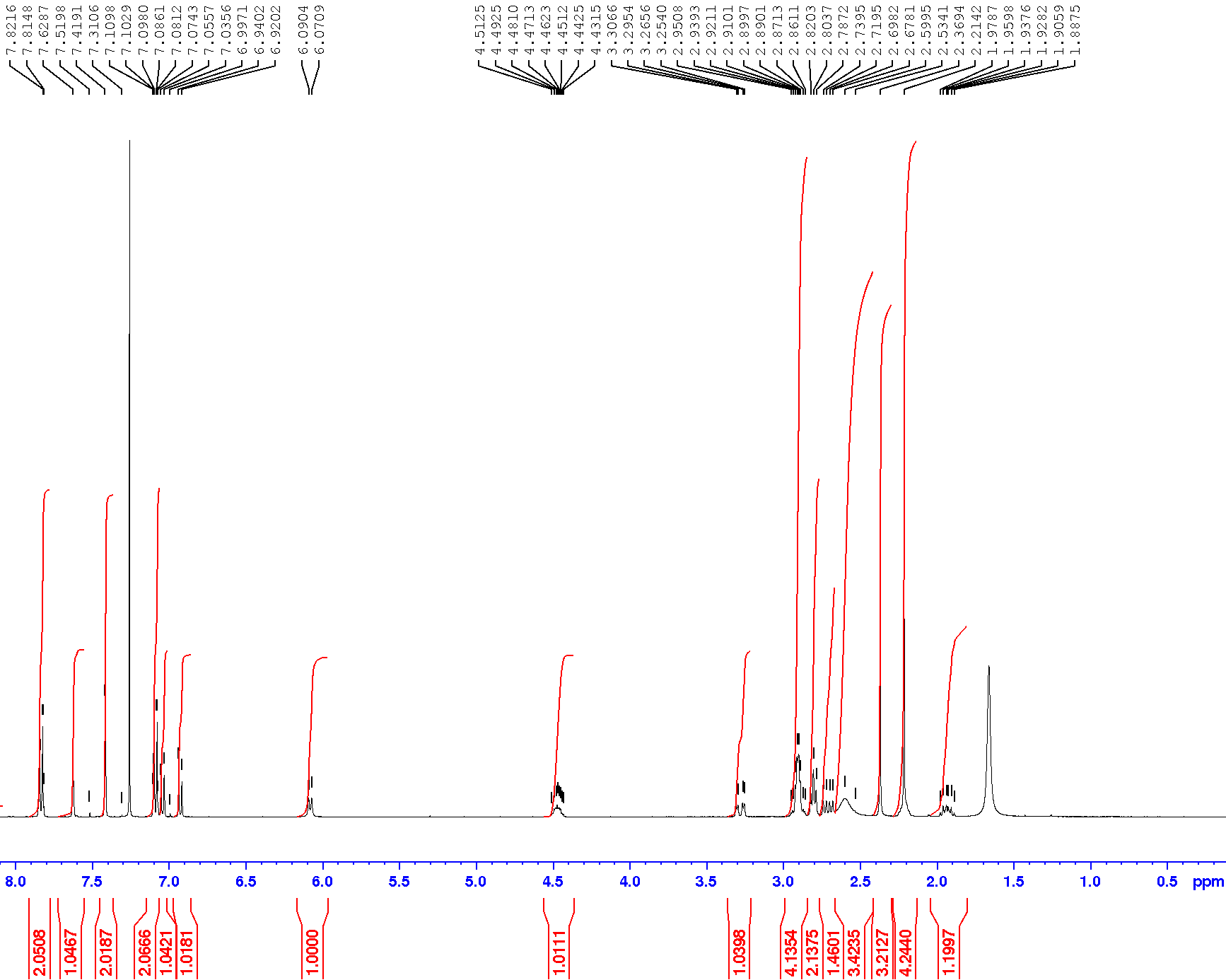


Compound **14**


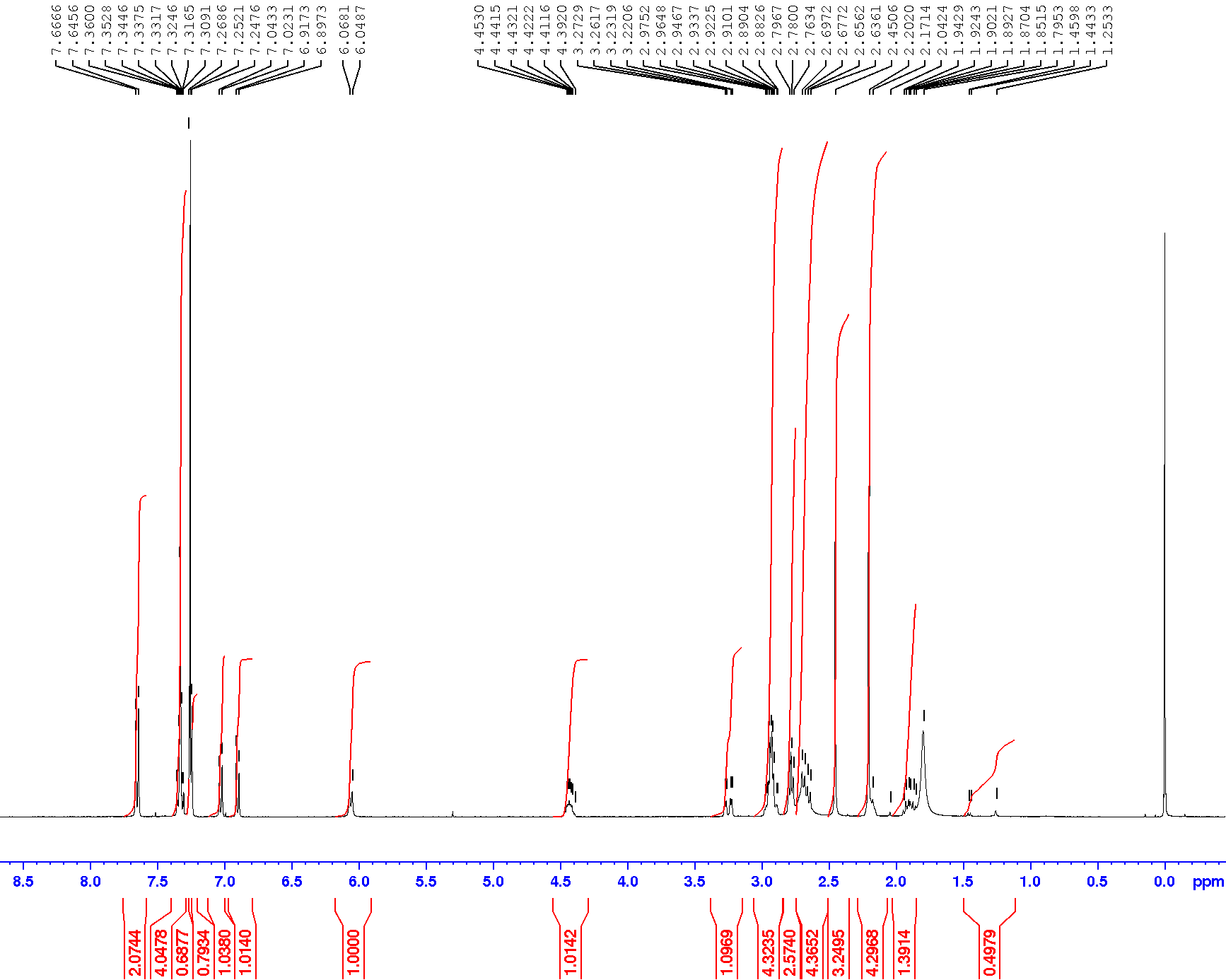


Compound **15**


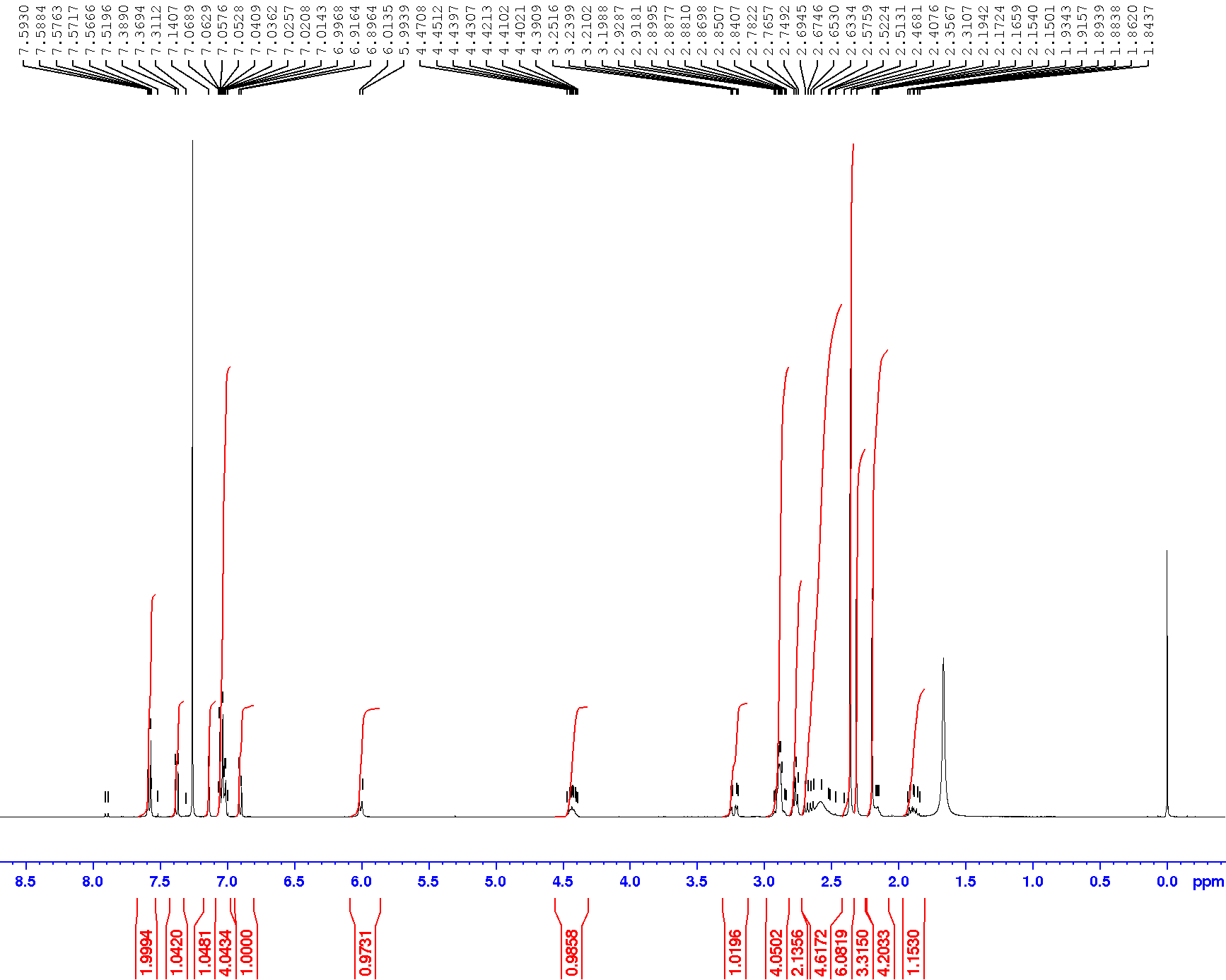


Compound **16**


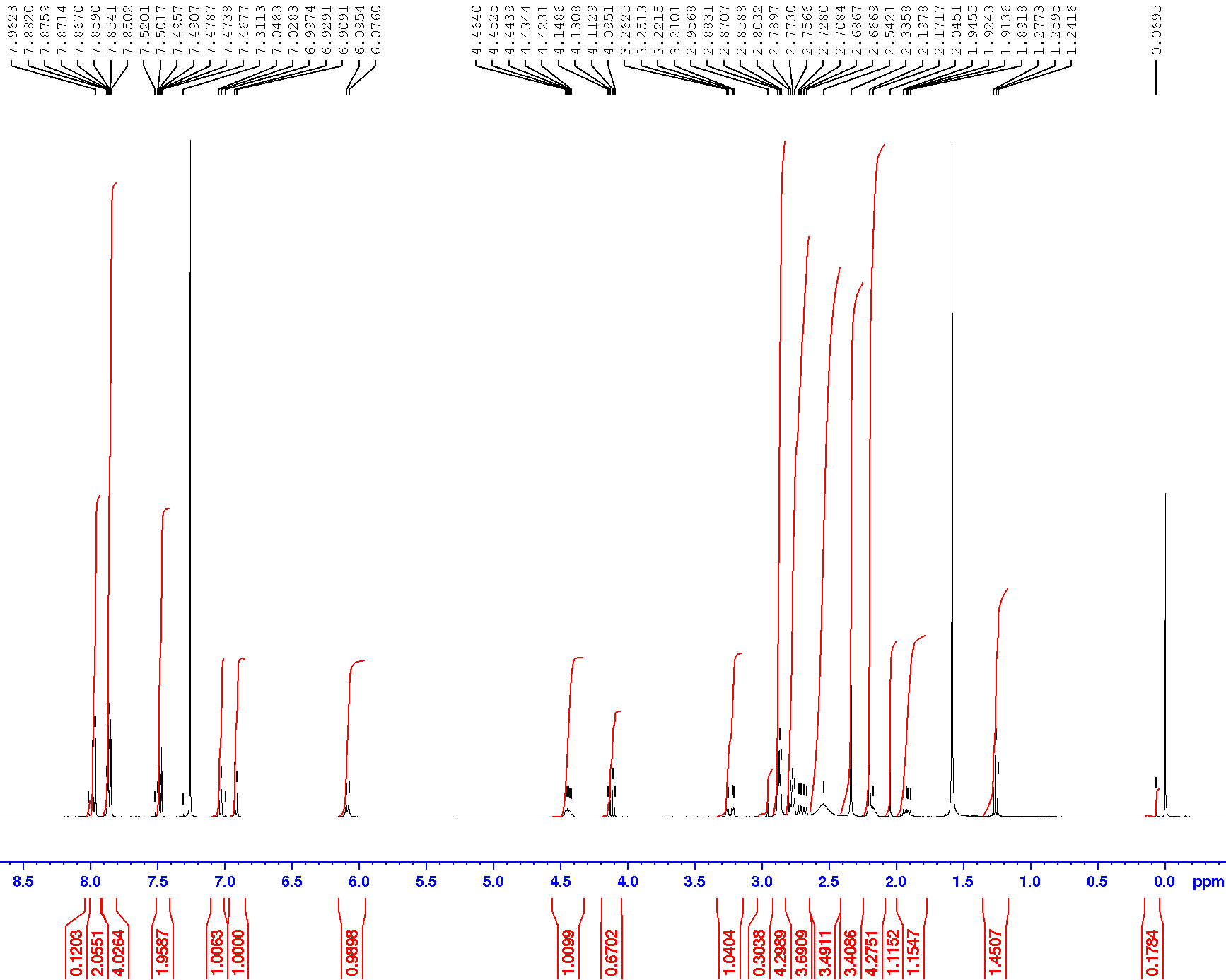


Compound **17**


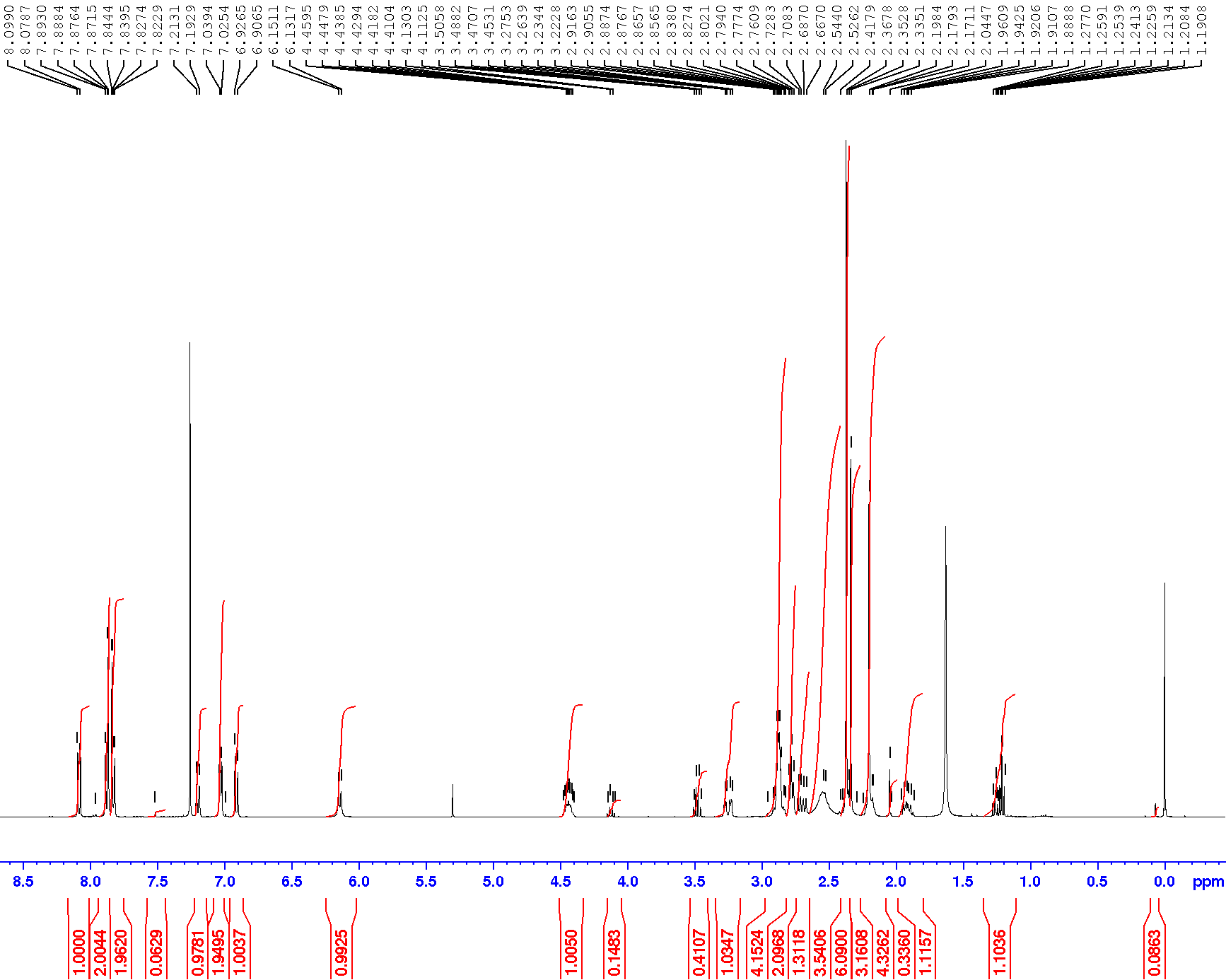


Compound **18**

Compound **19**


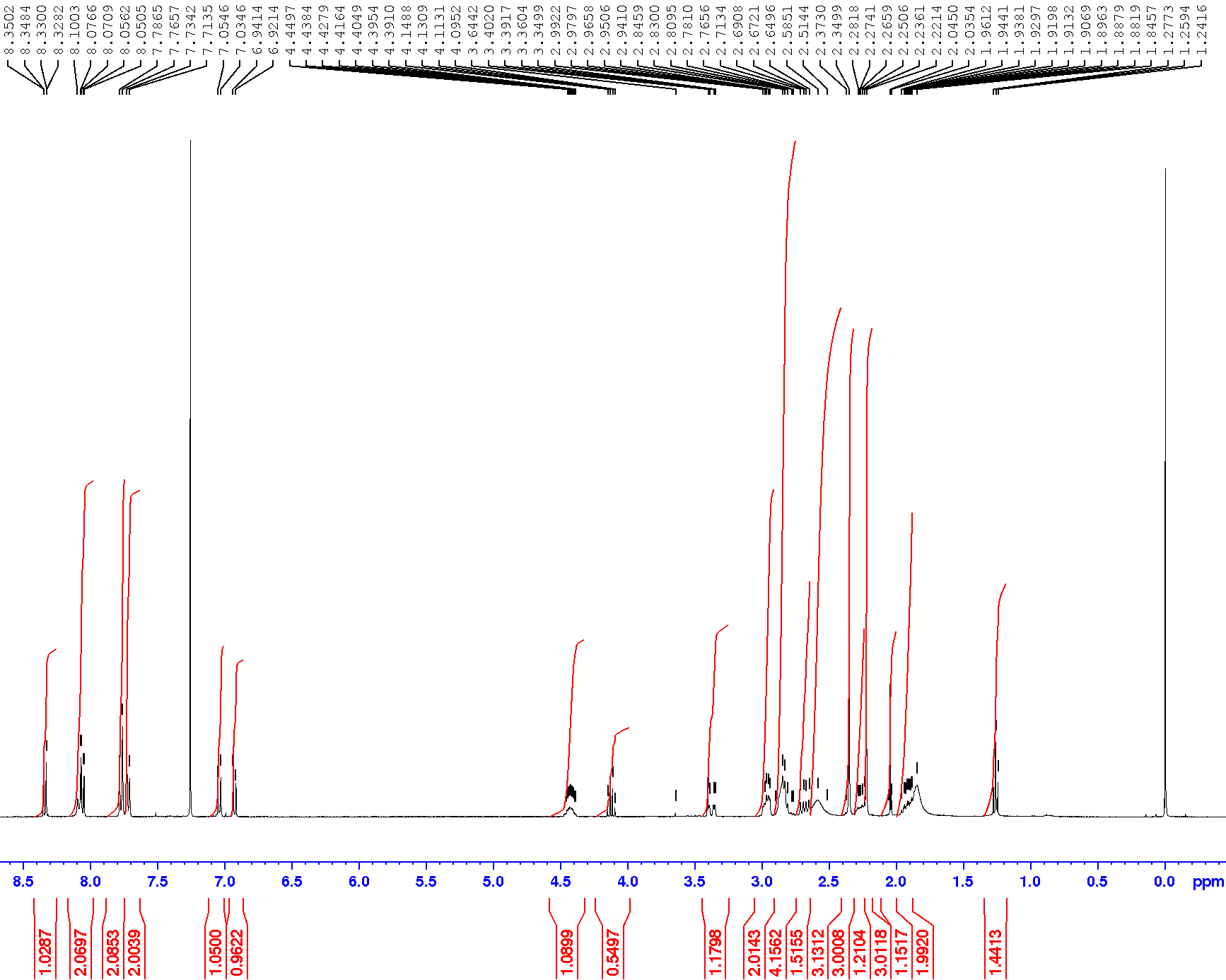


Compound **19R**


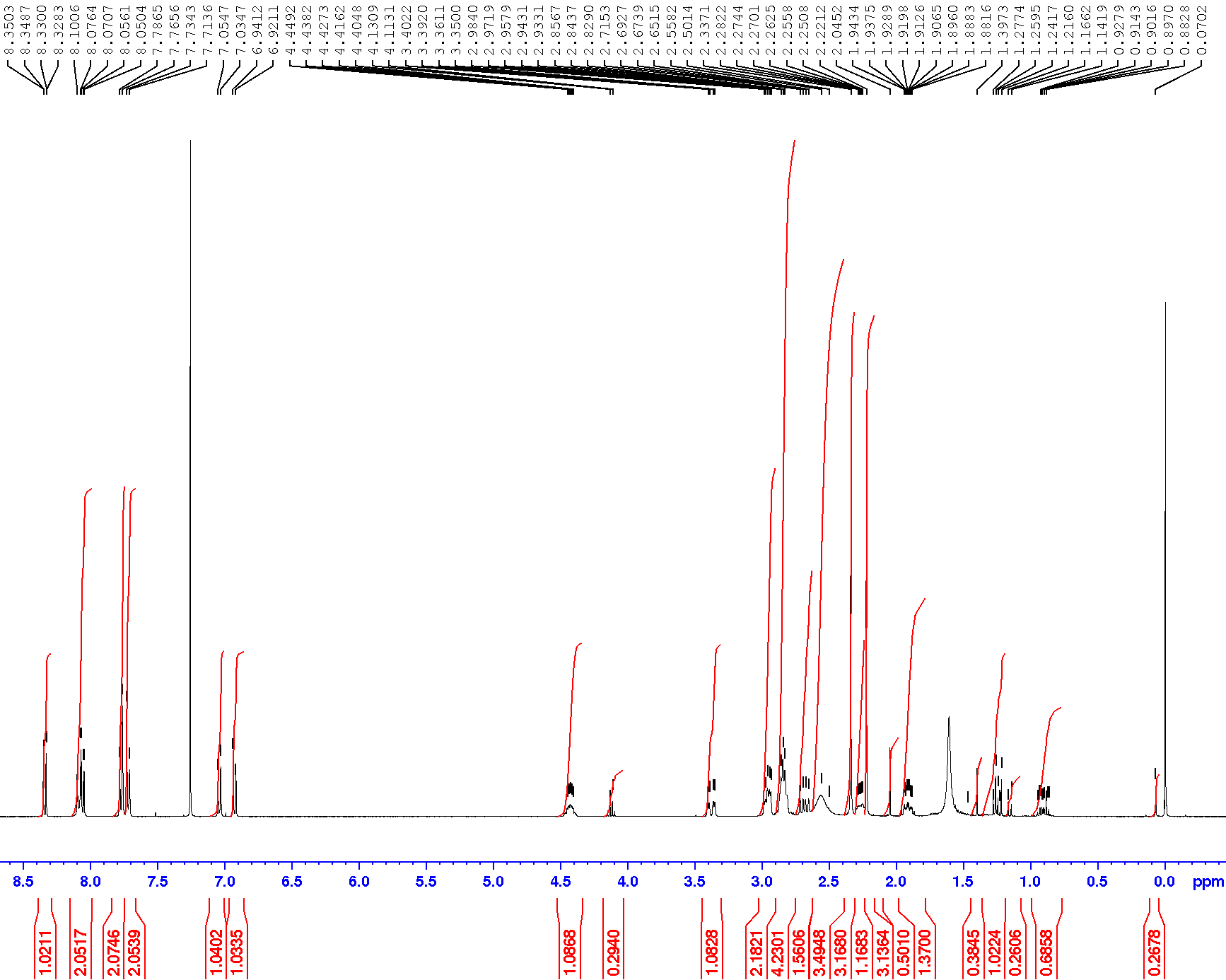


Compound **20**


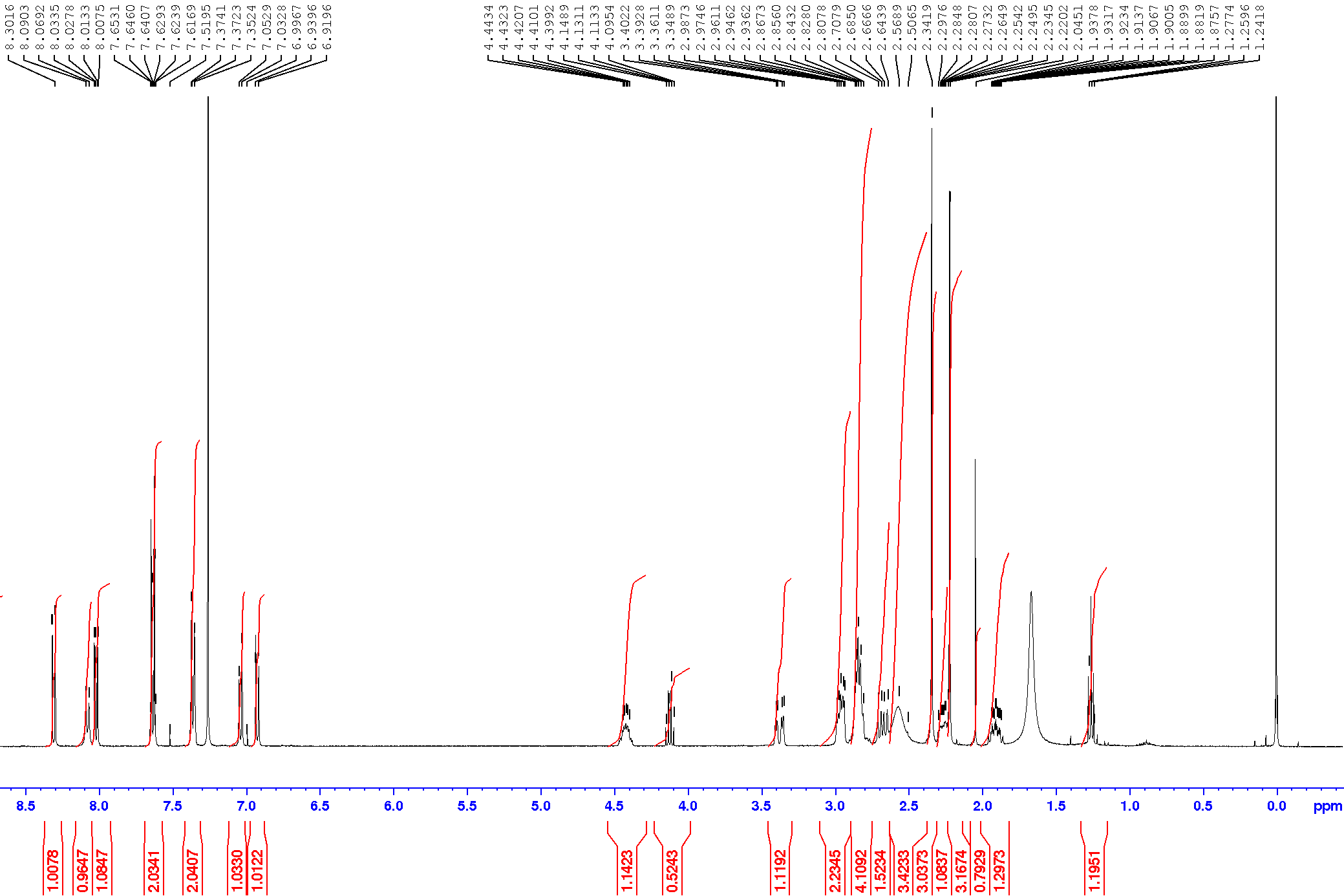

Compound **20R**


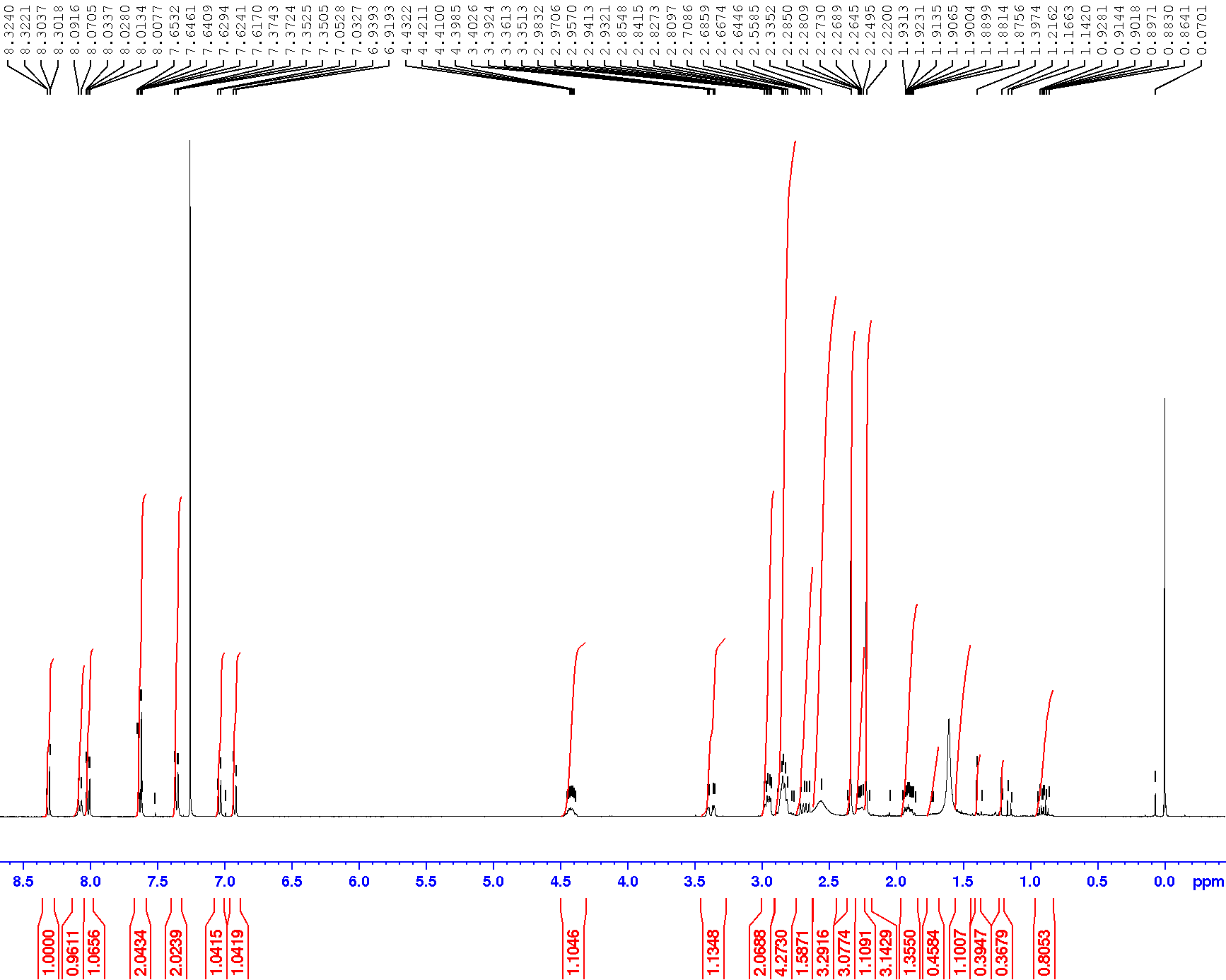


Compound **21**


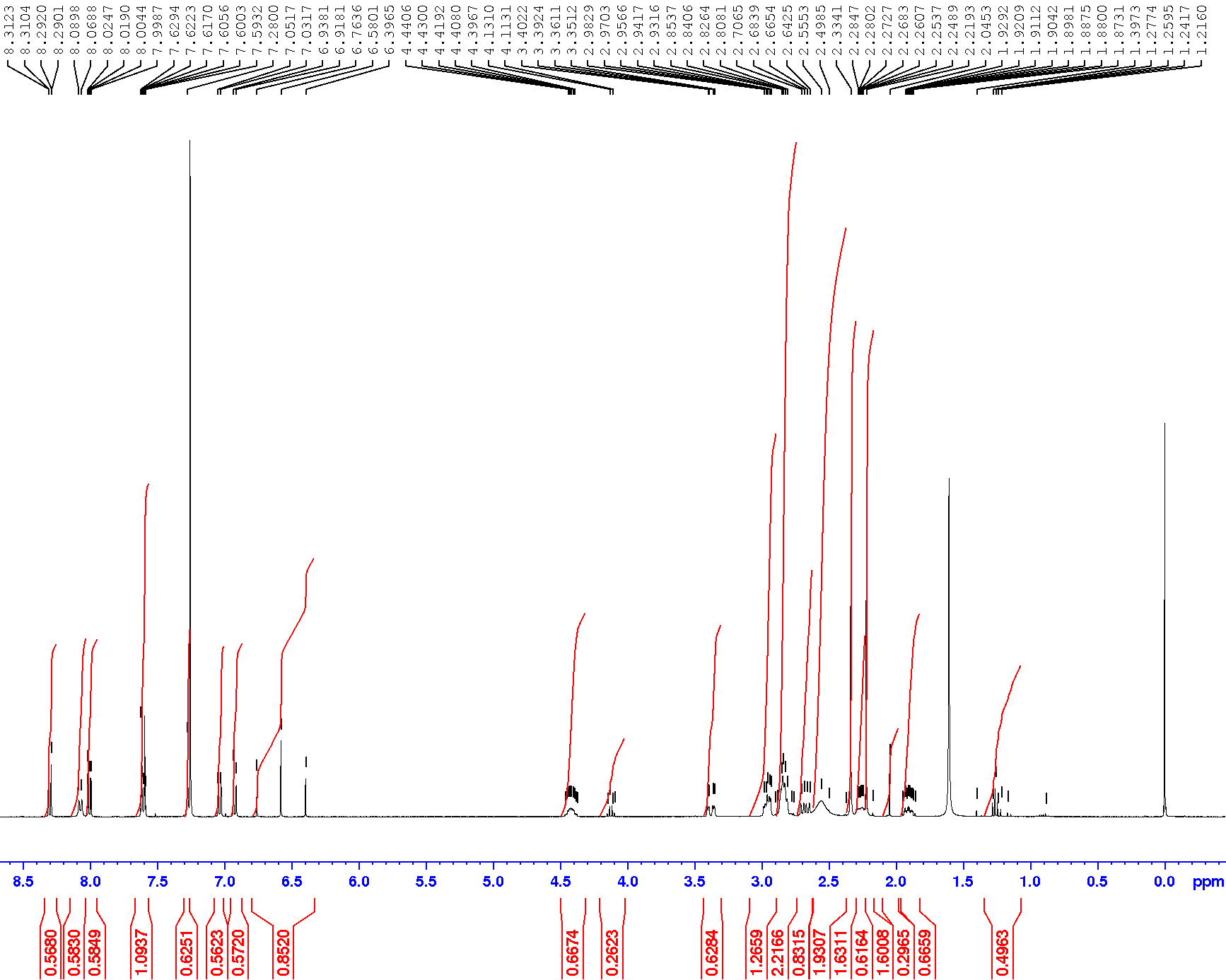


Compound **21R**


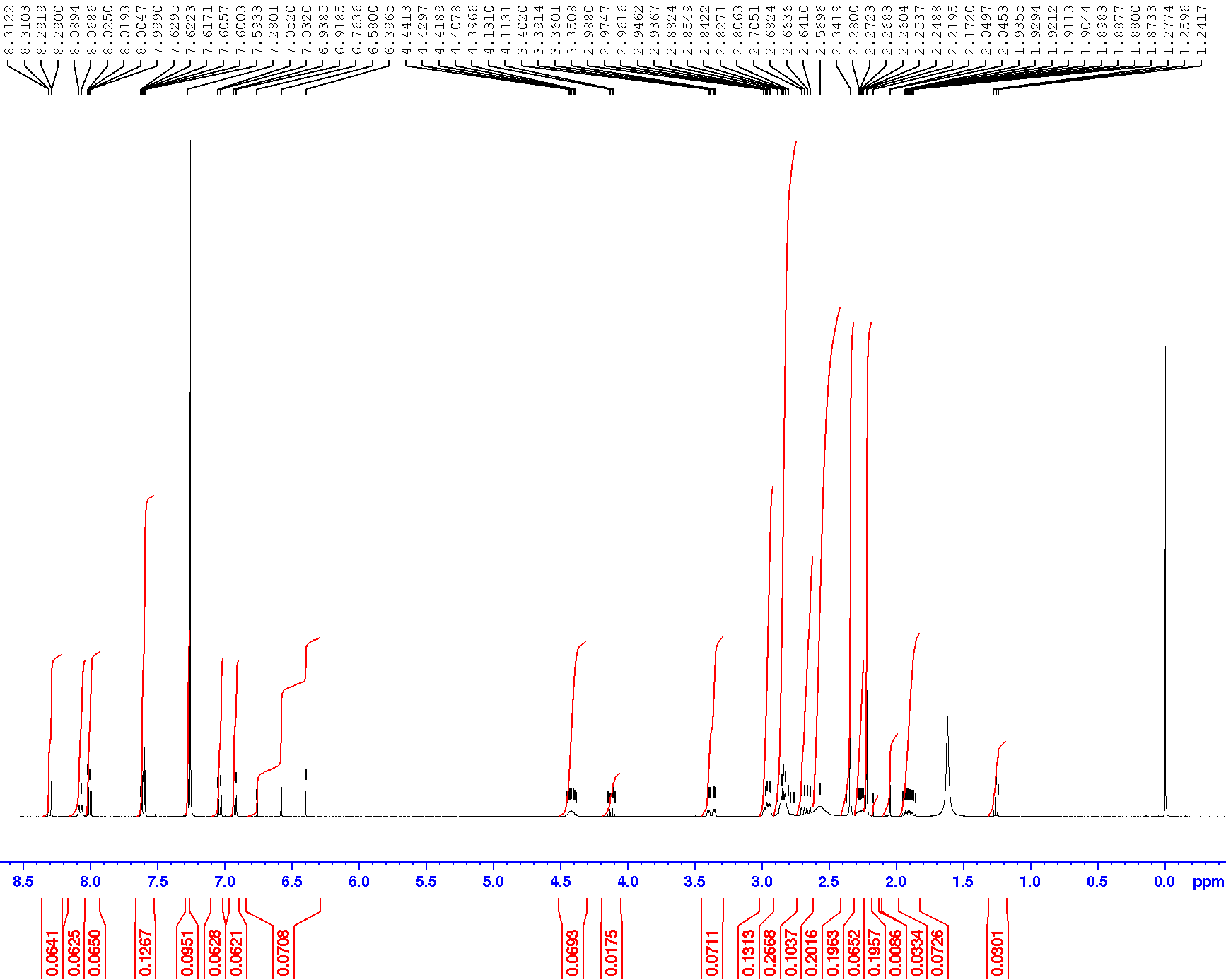


Compound **22**

Compound **22R**


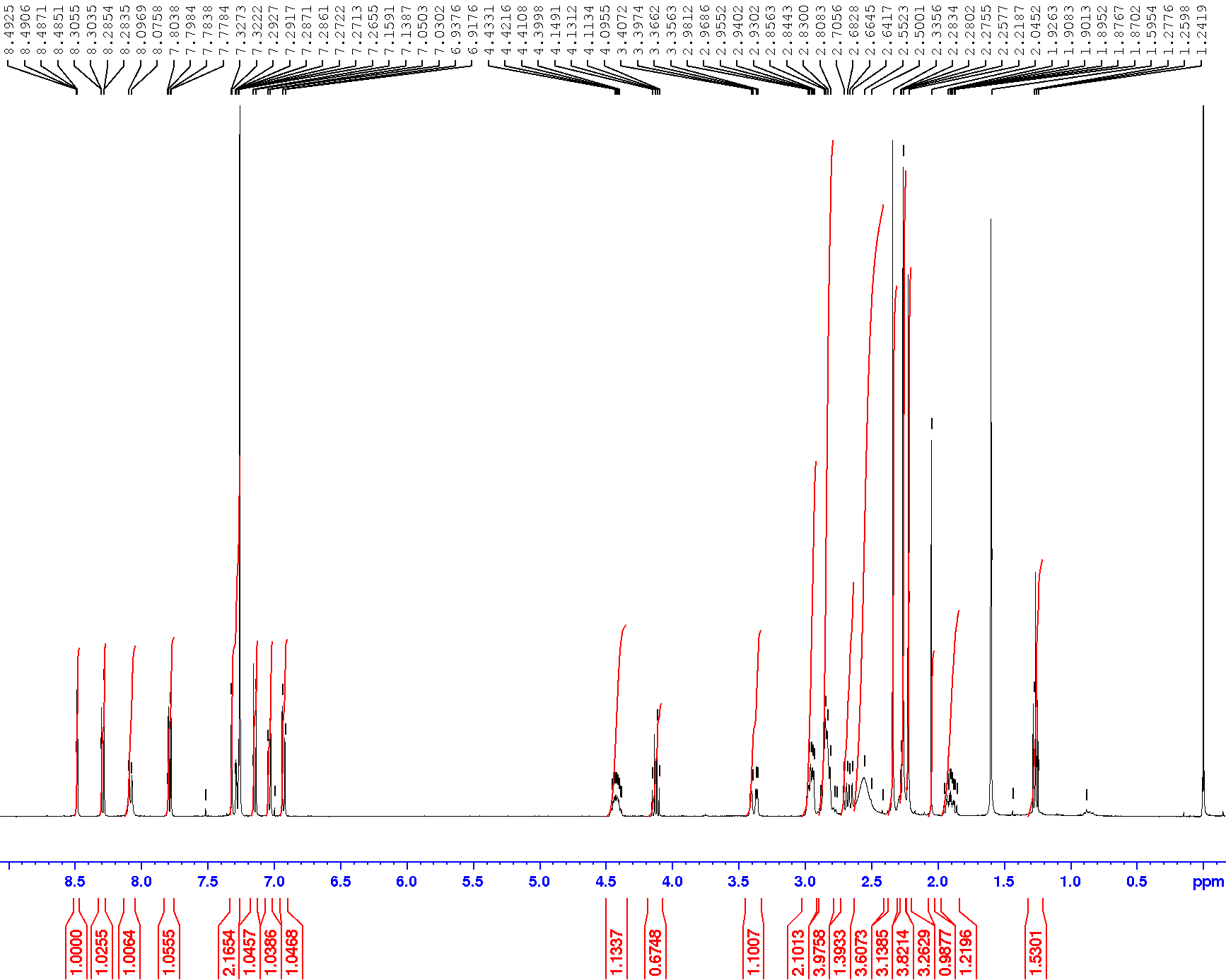


Compound **23**


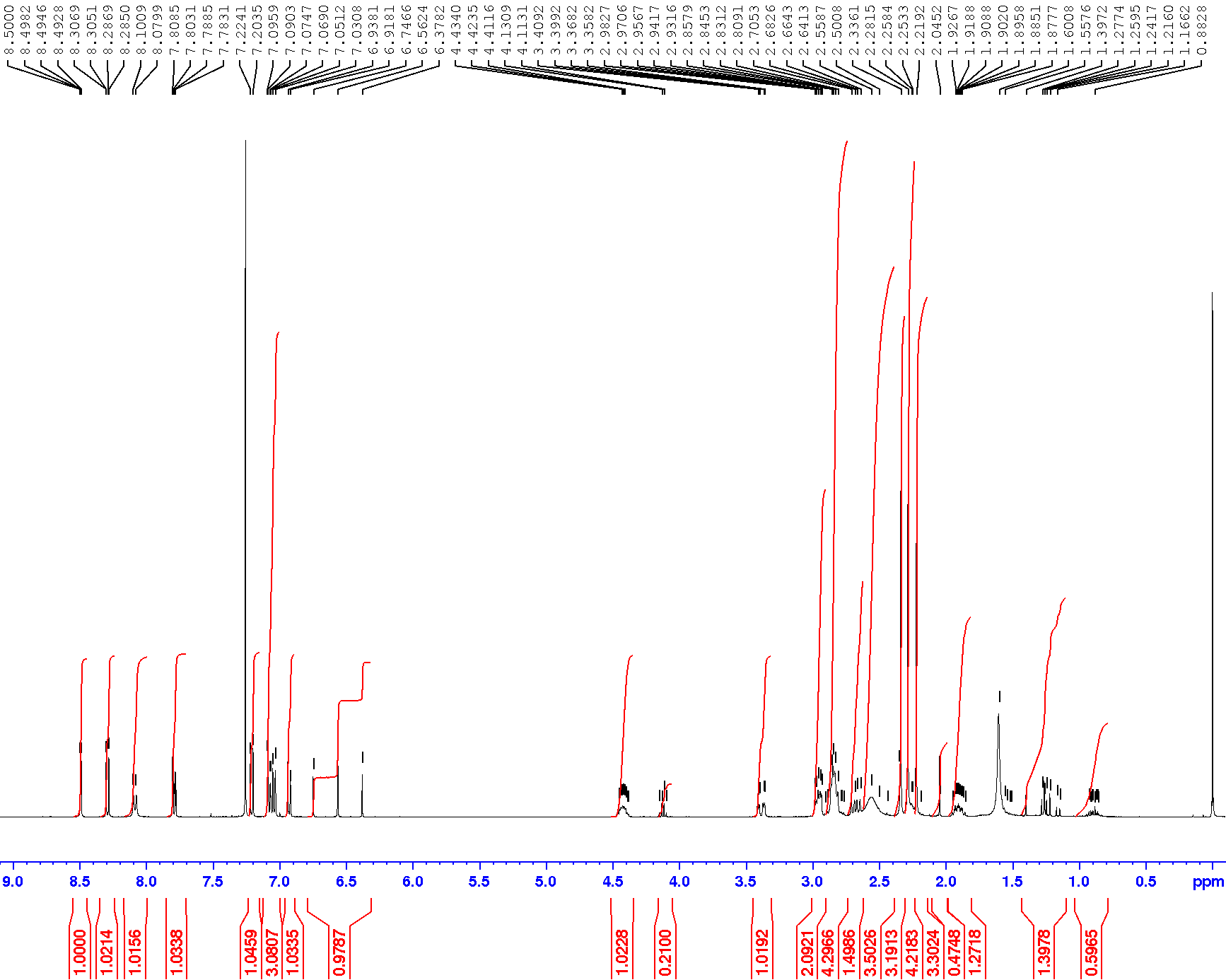


Compound **23R**


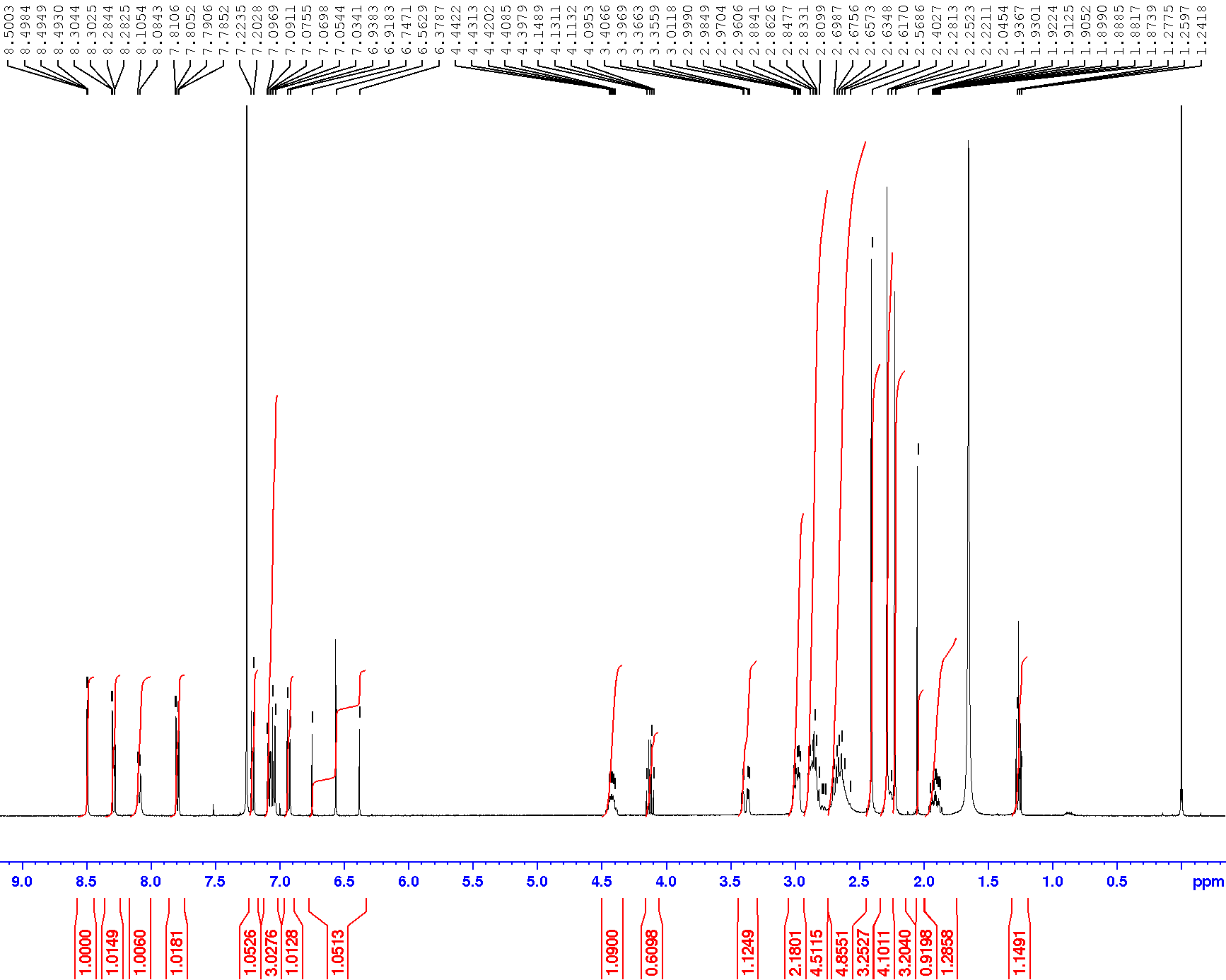


Compound **24**


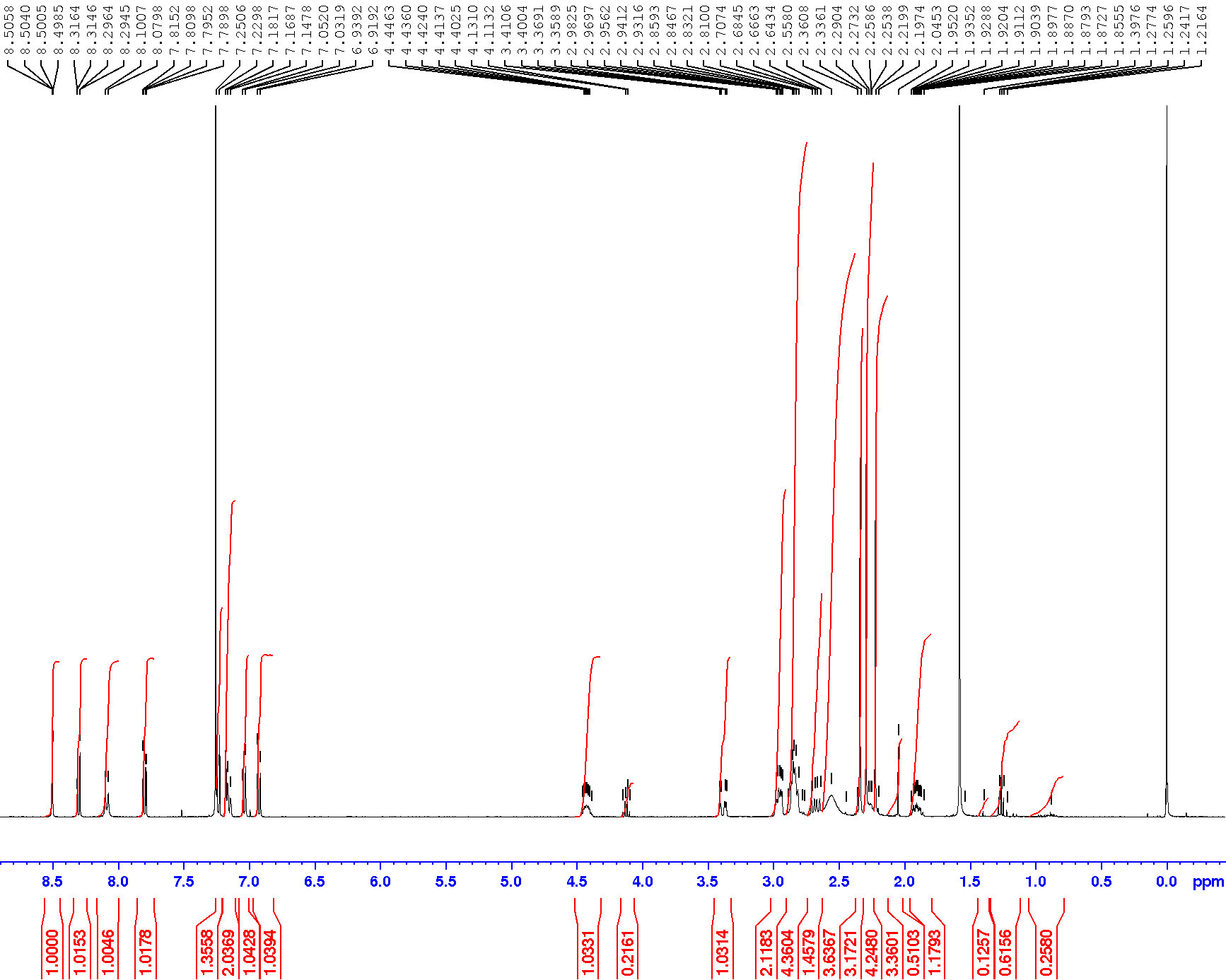


Compound **24R**


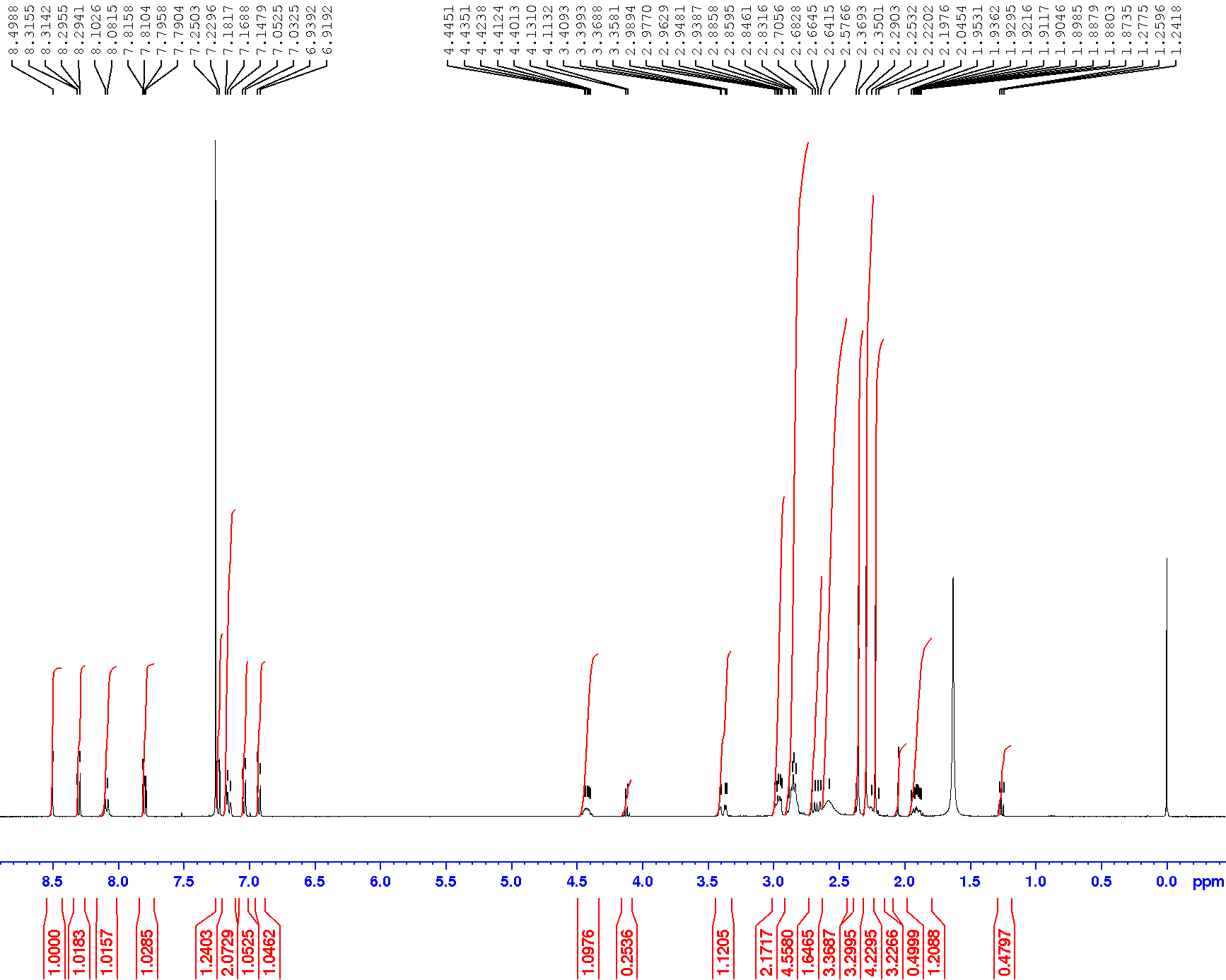


Compound **25**


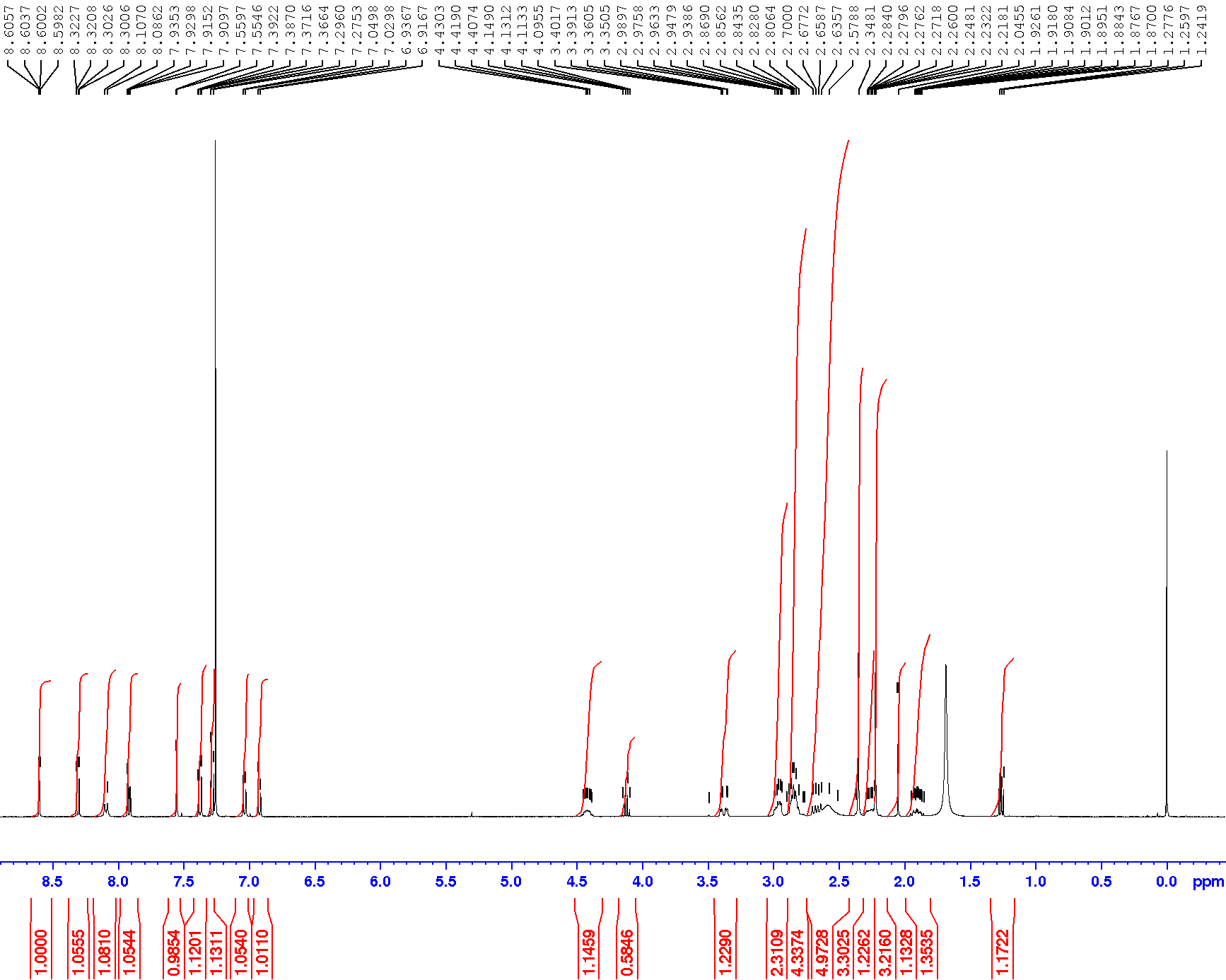


Compound **26**


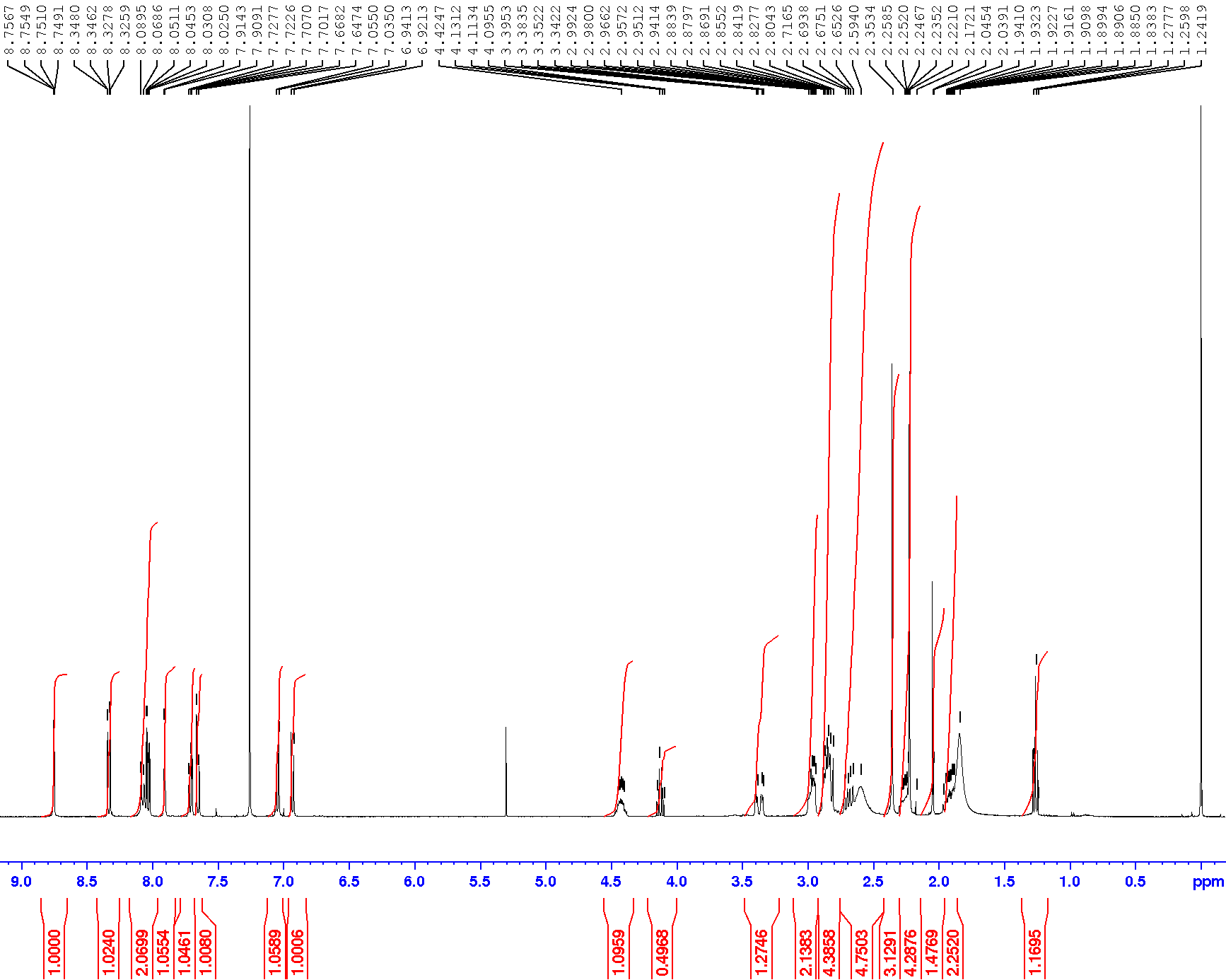


Compound **27**


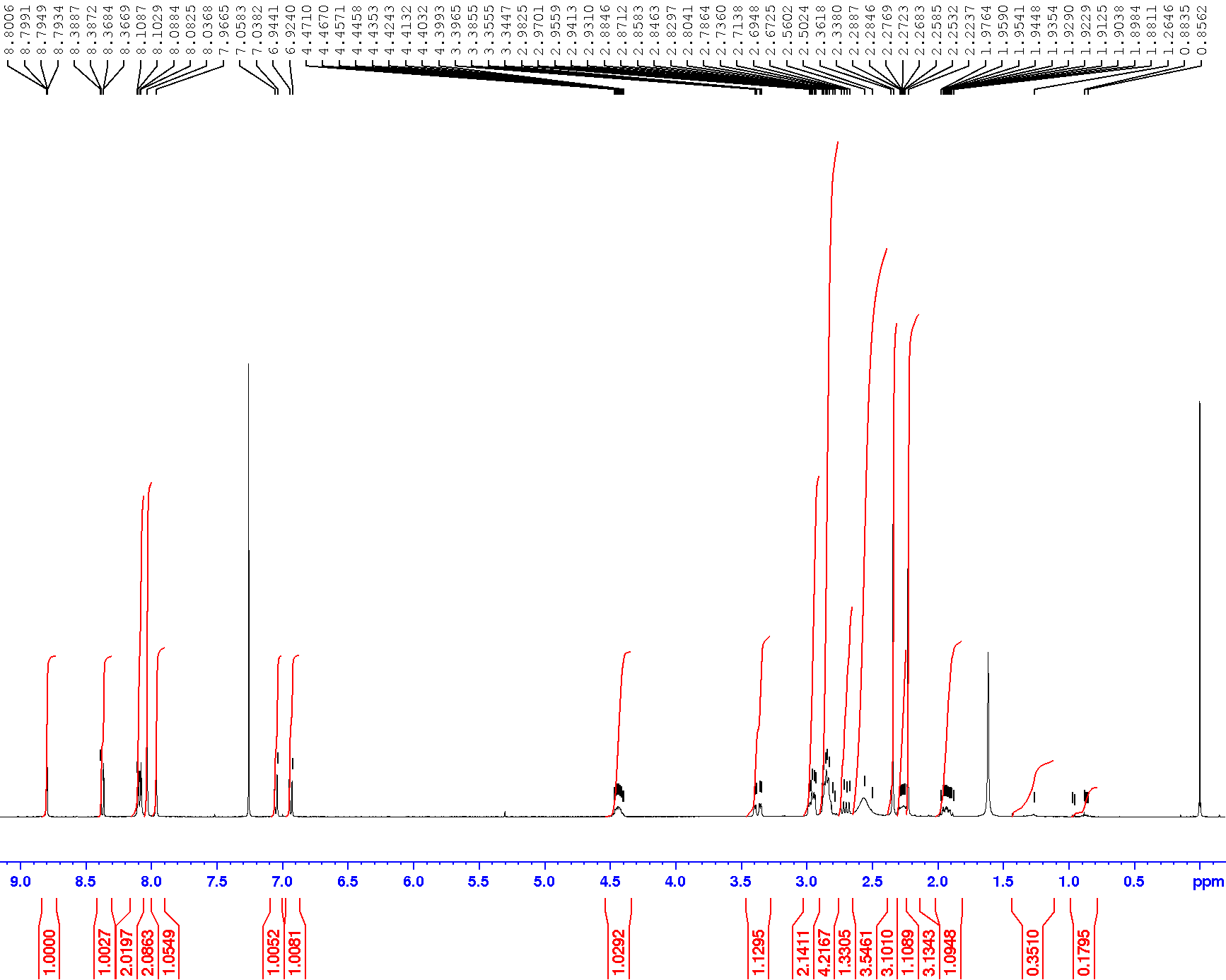

Compound **28**


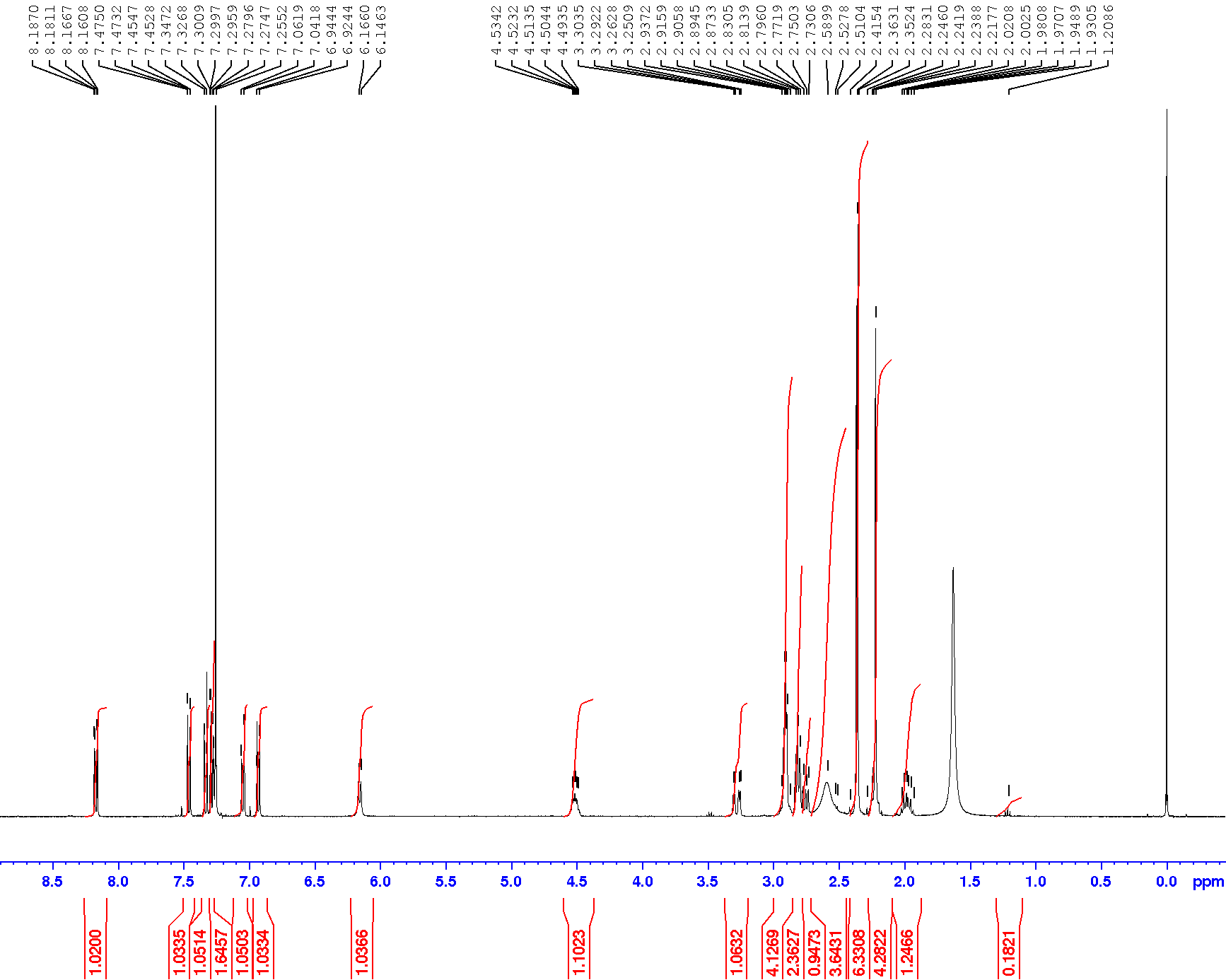

Compound **28R**


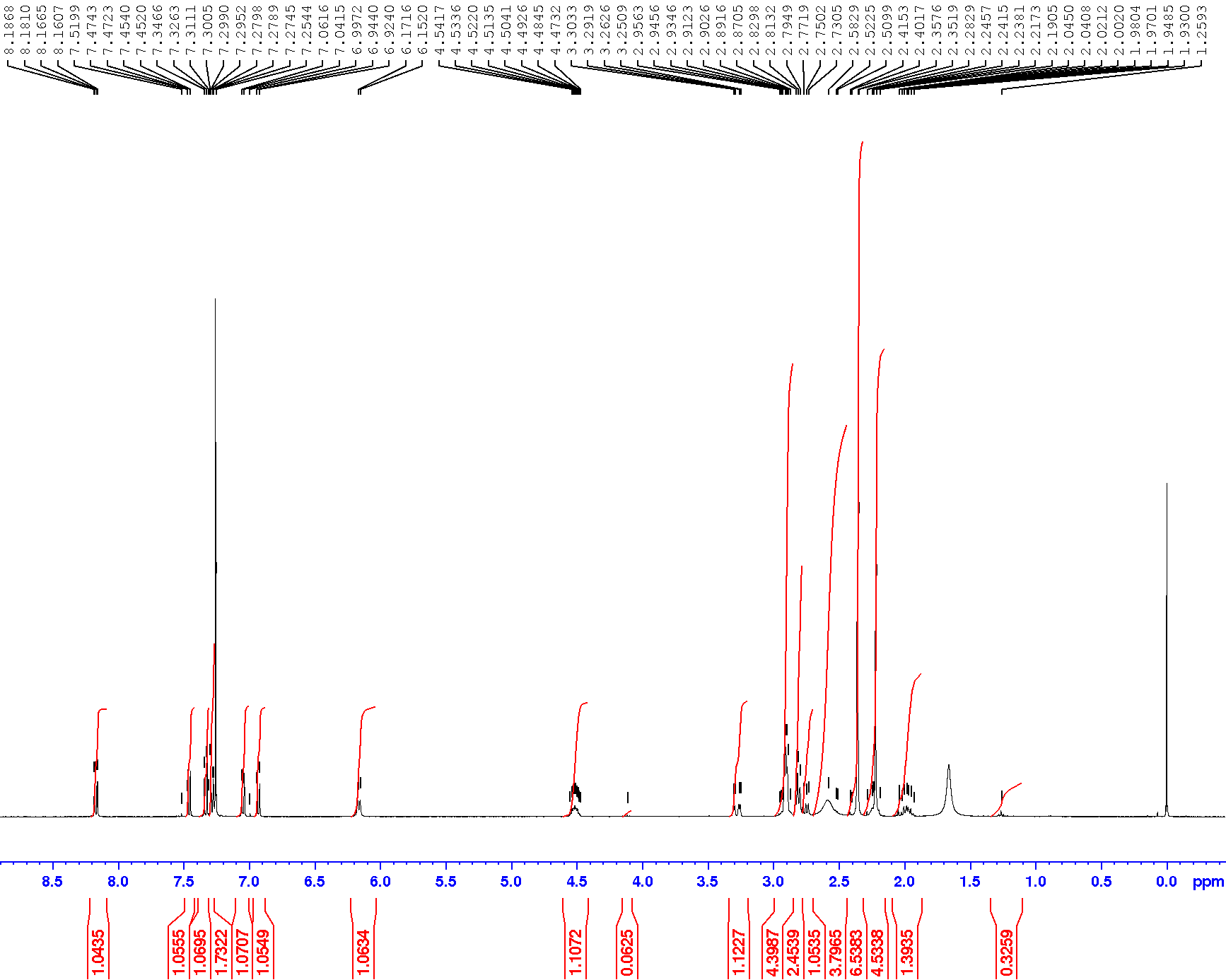


Compound **29**


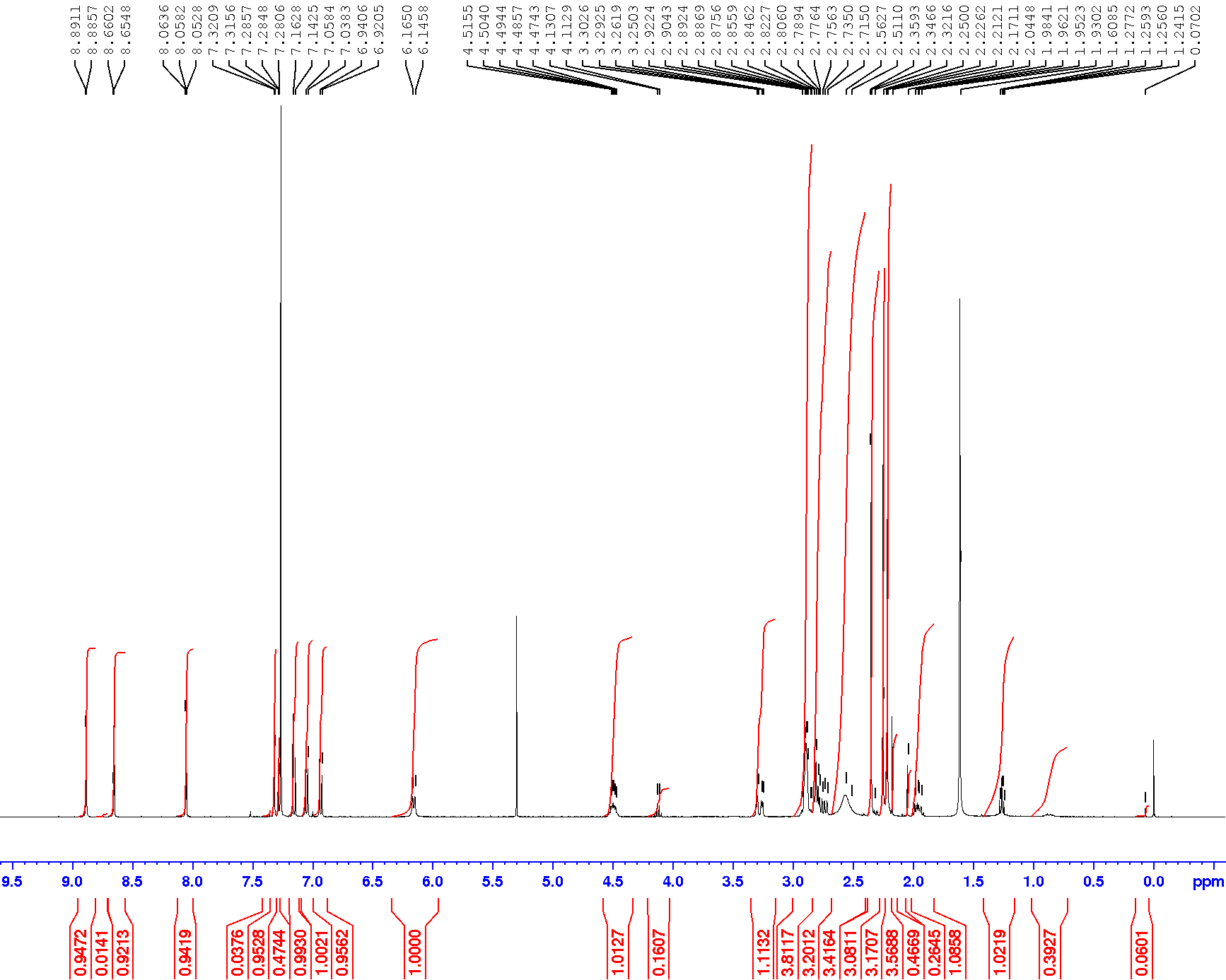

Compound **29R**


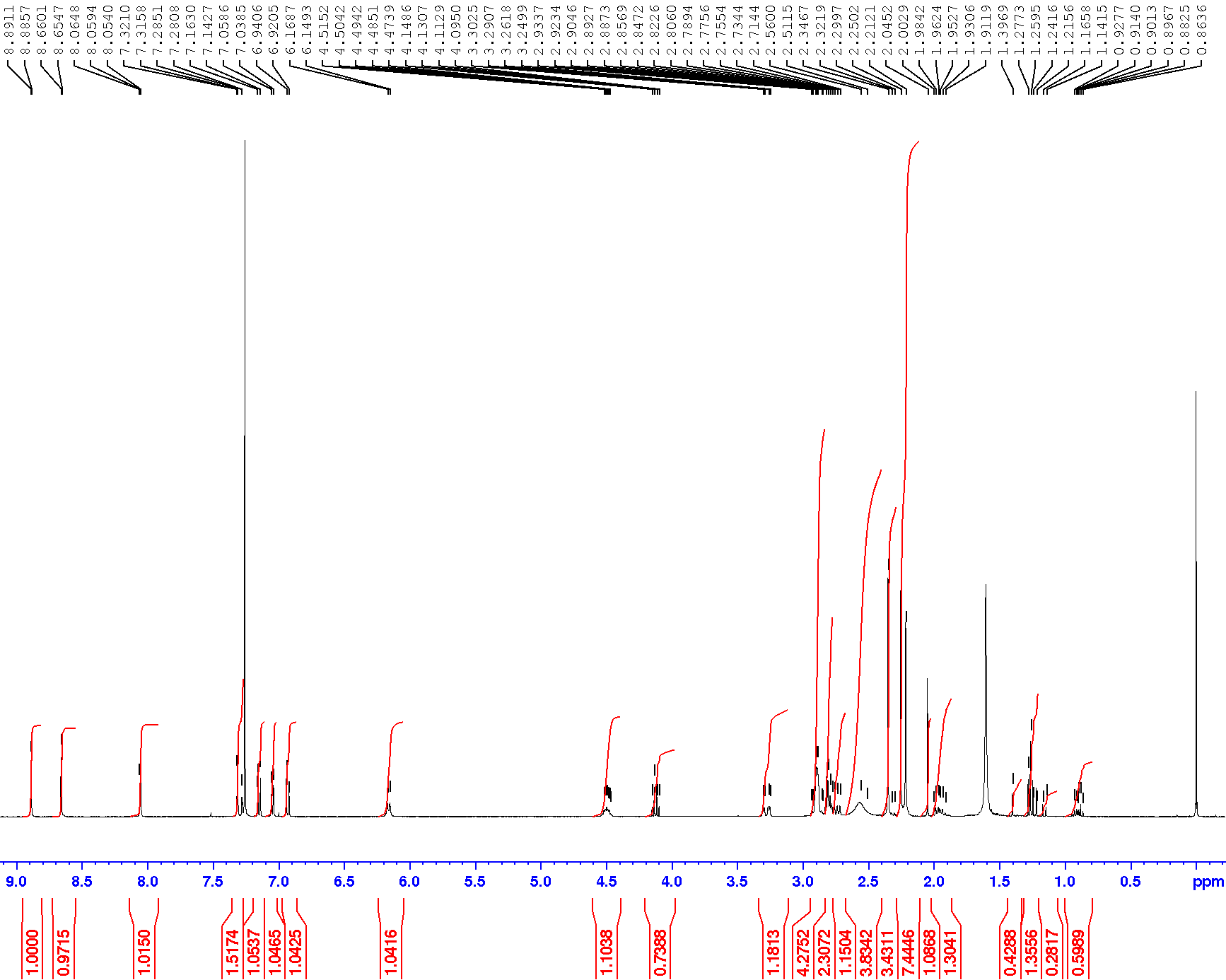


Compound **30**

**
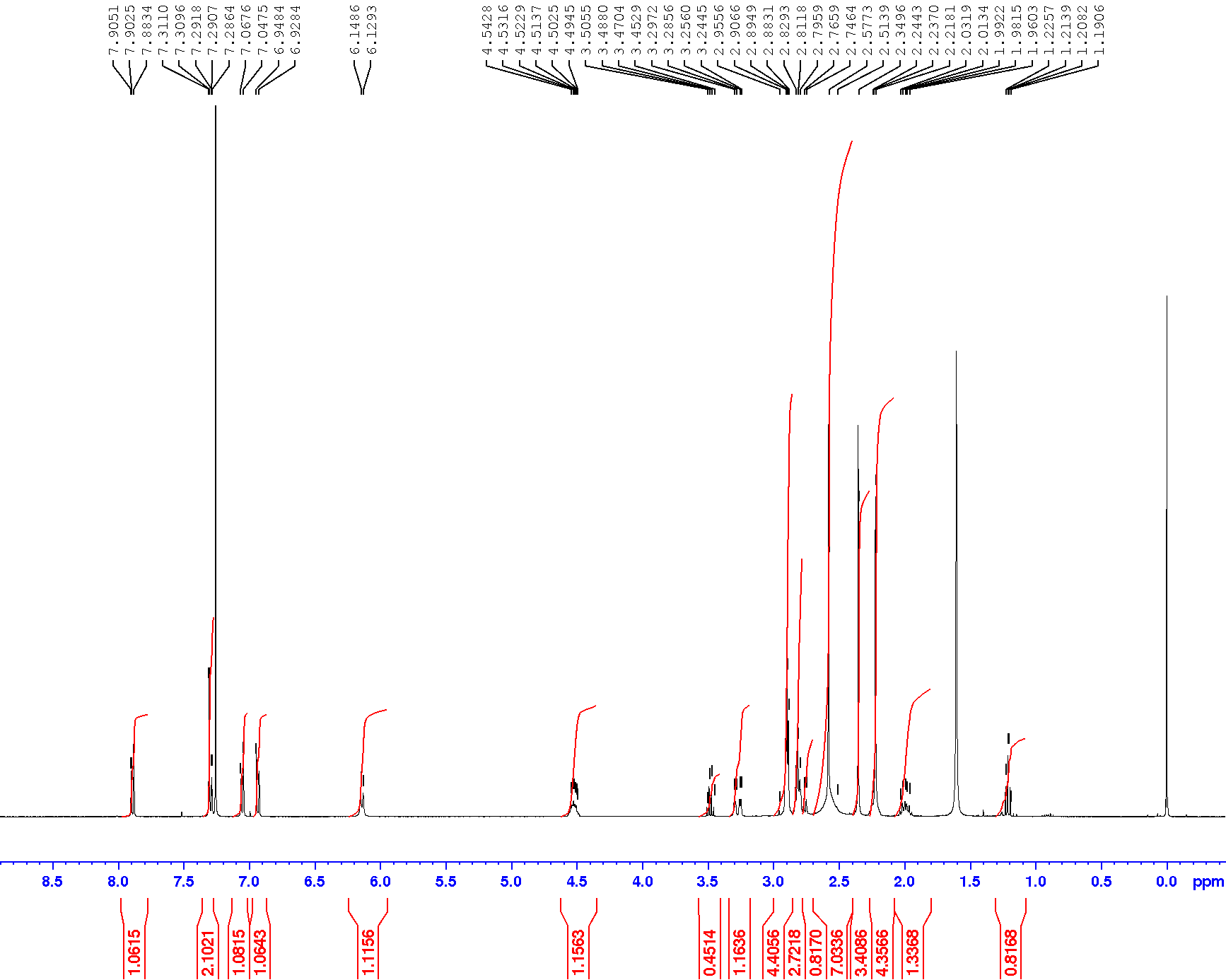
**

Compound **30R**


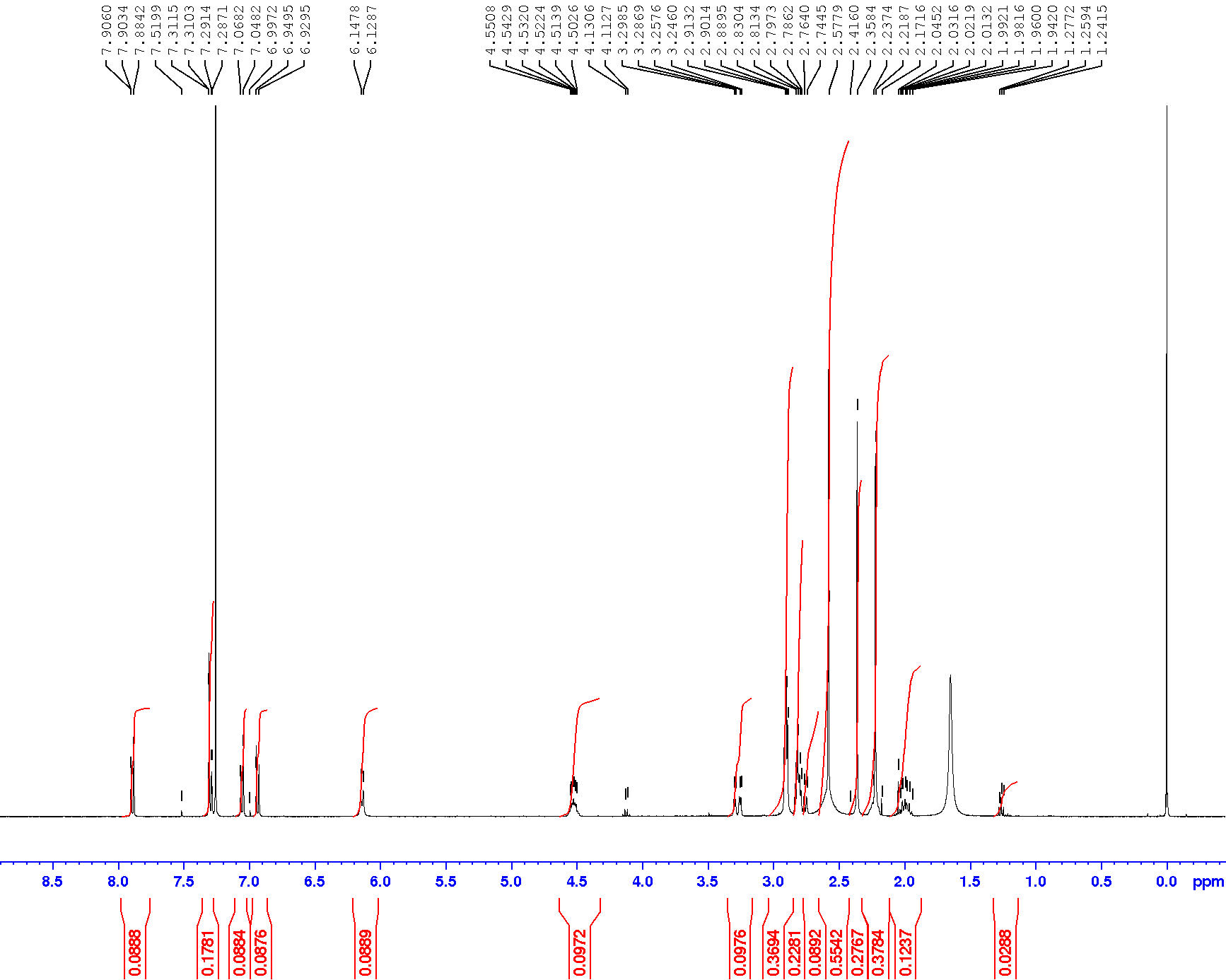


Compound **31**


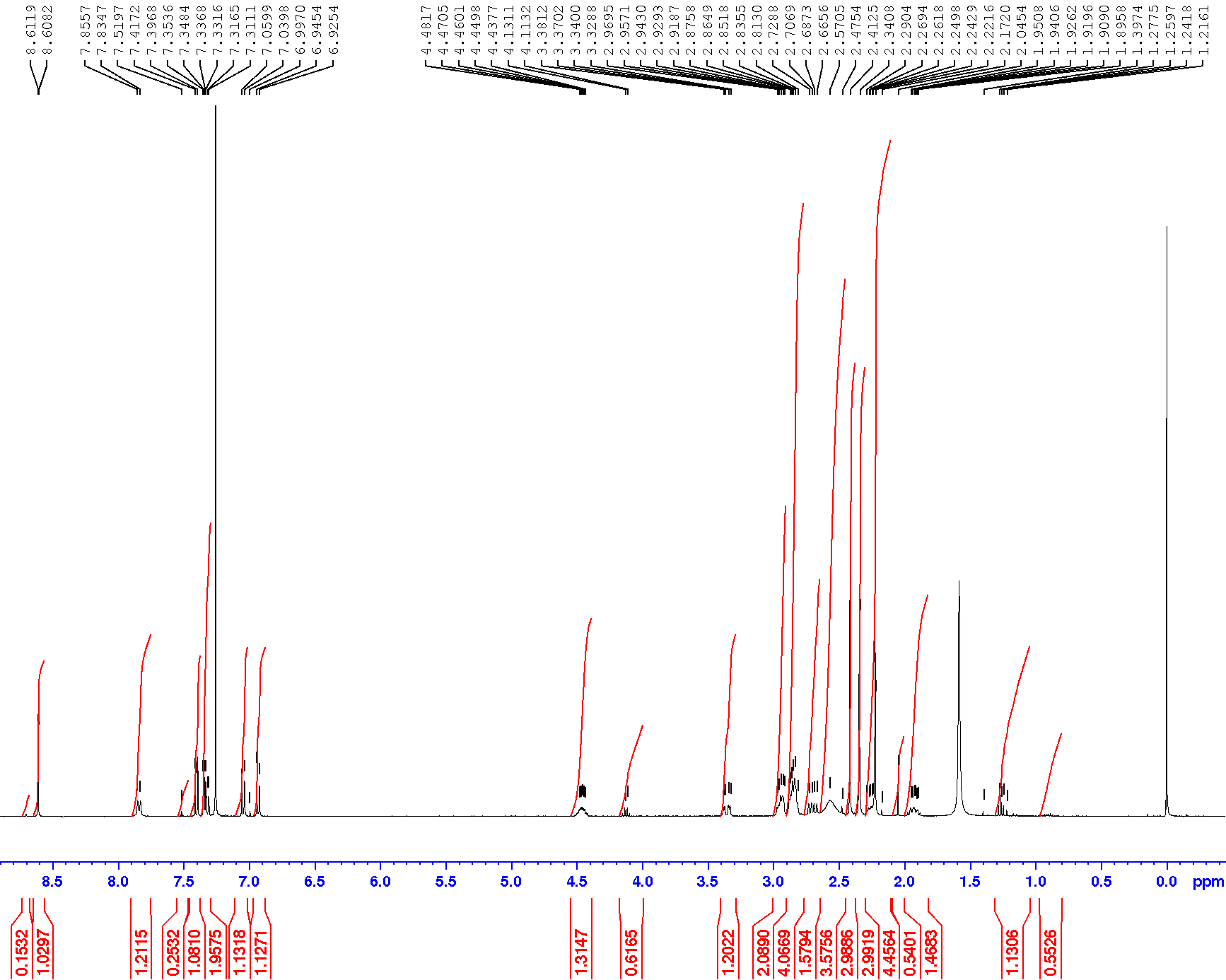

Compound **32**

Compound **32R**


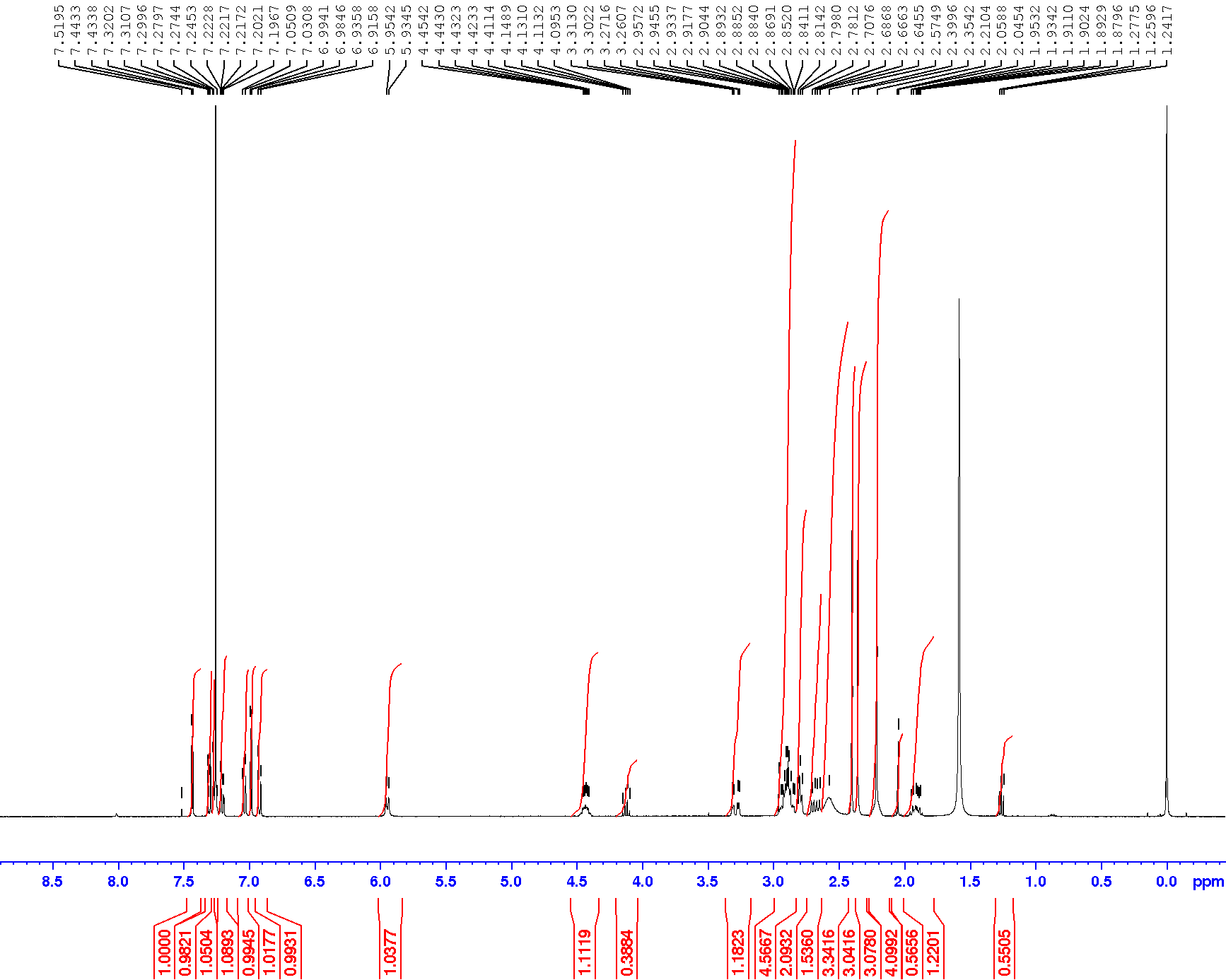


Compound **33**


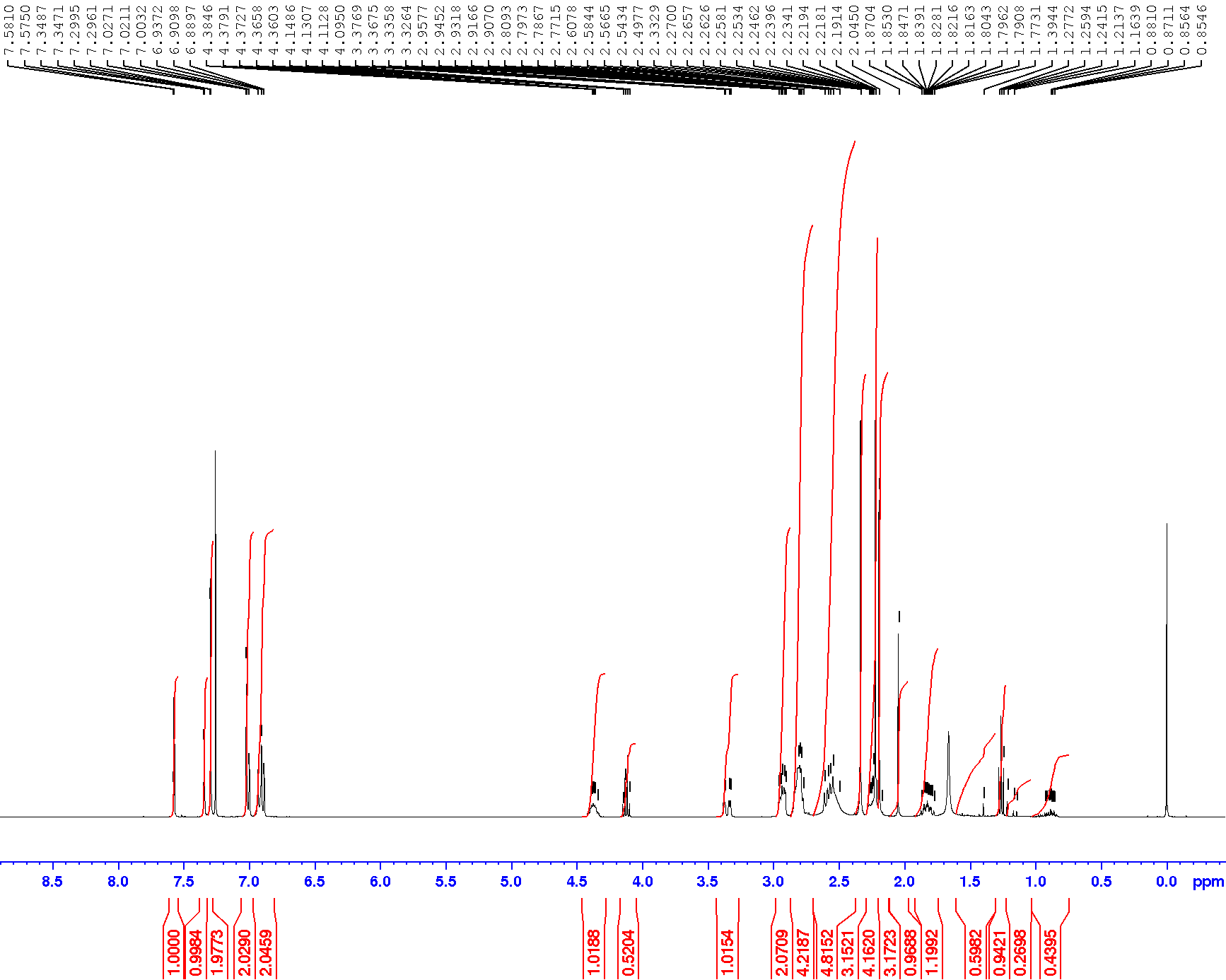


Compound **34**


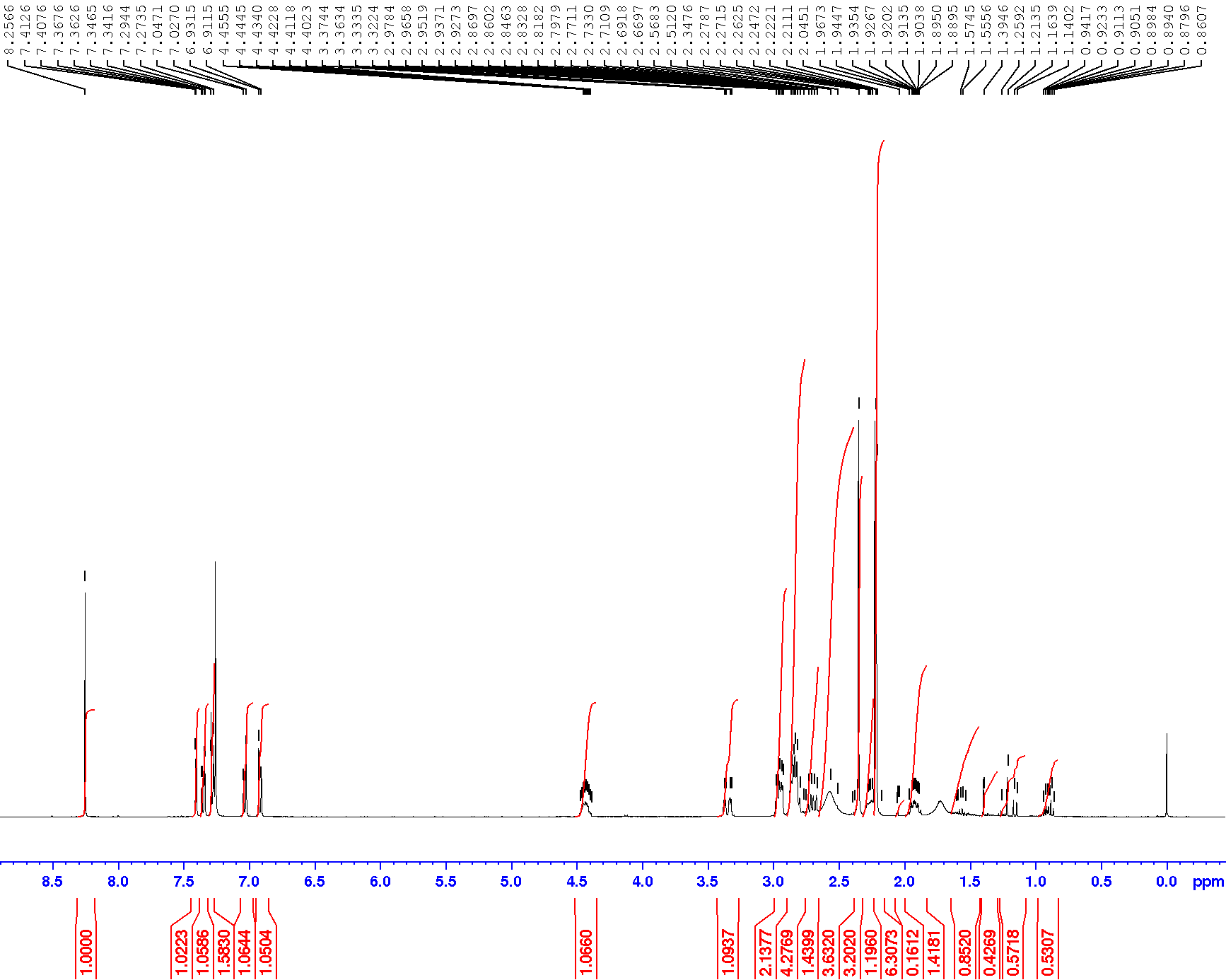


Compound **34R**


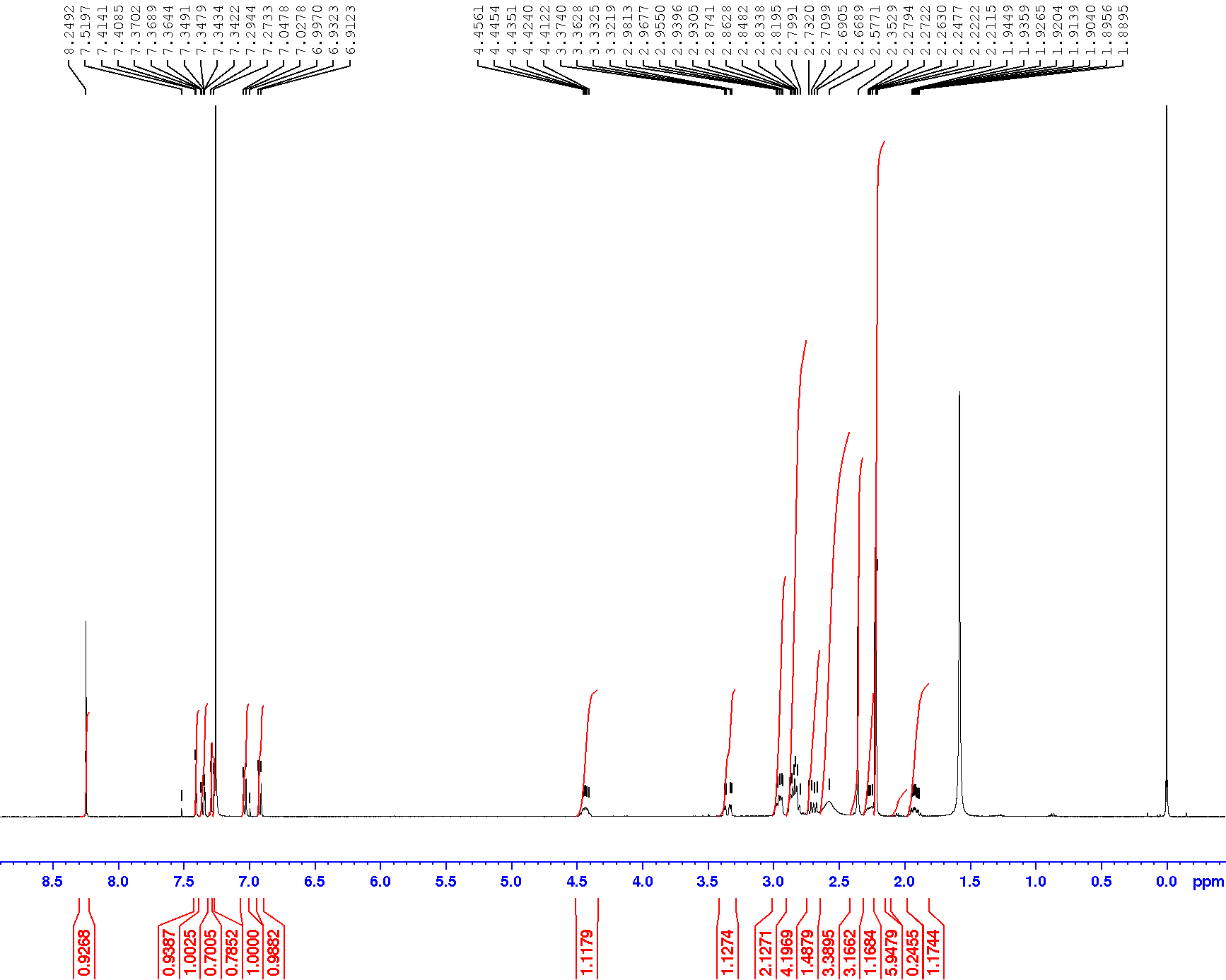


Compound **35**


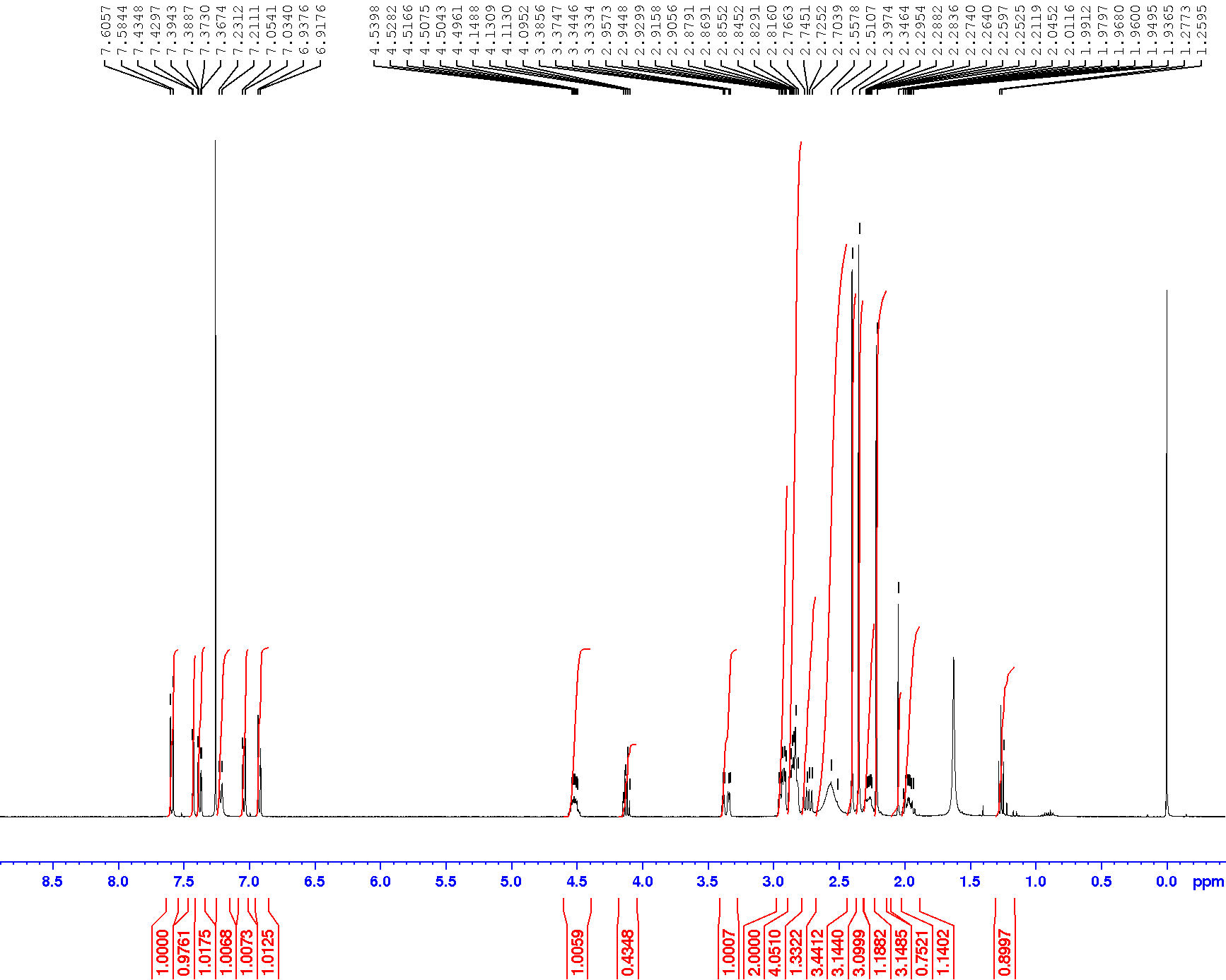


Compound **36**


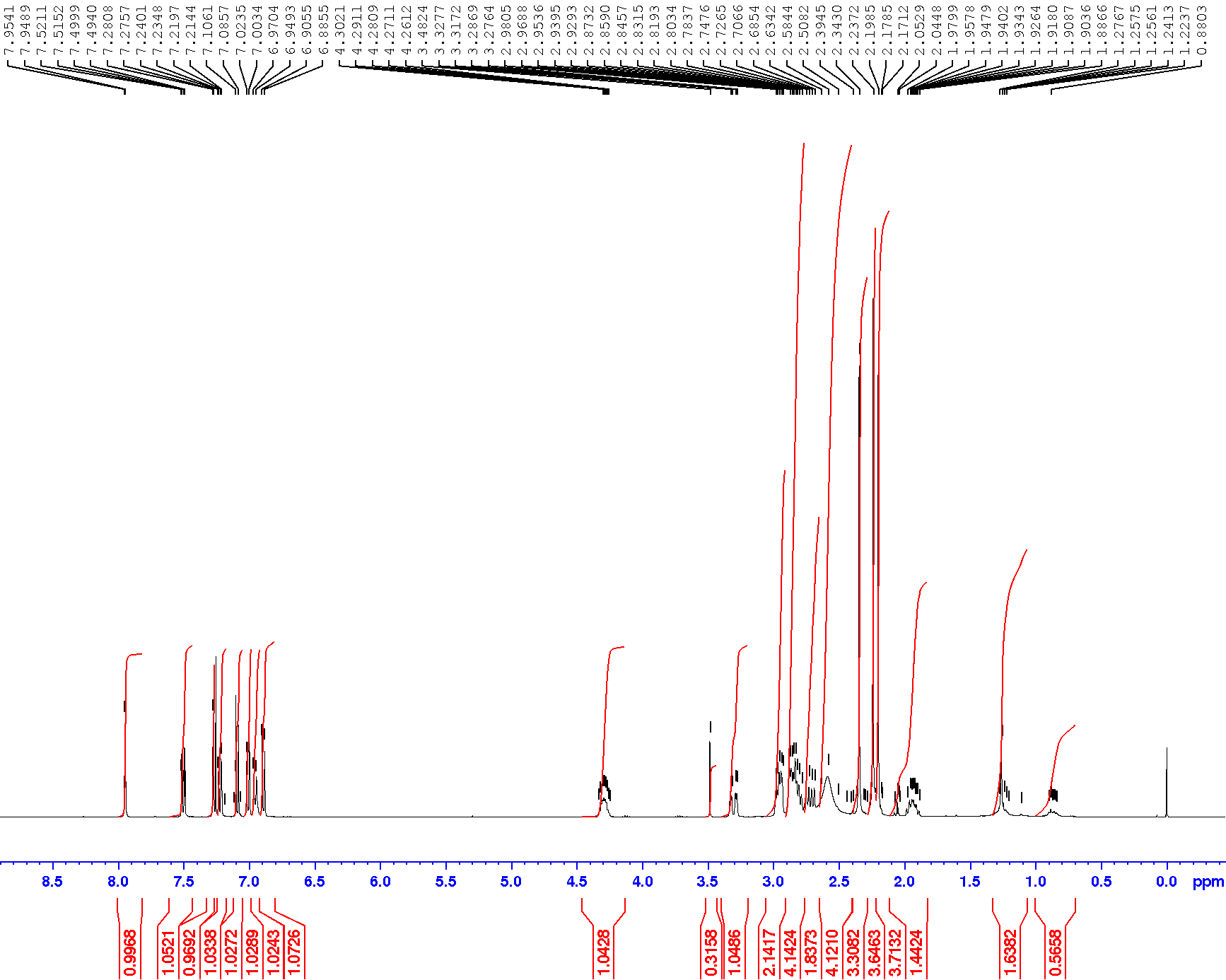


Compound **37**


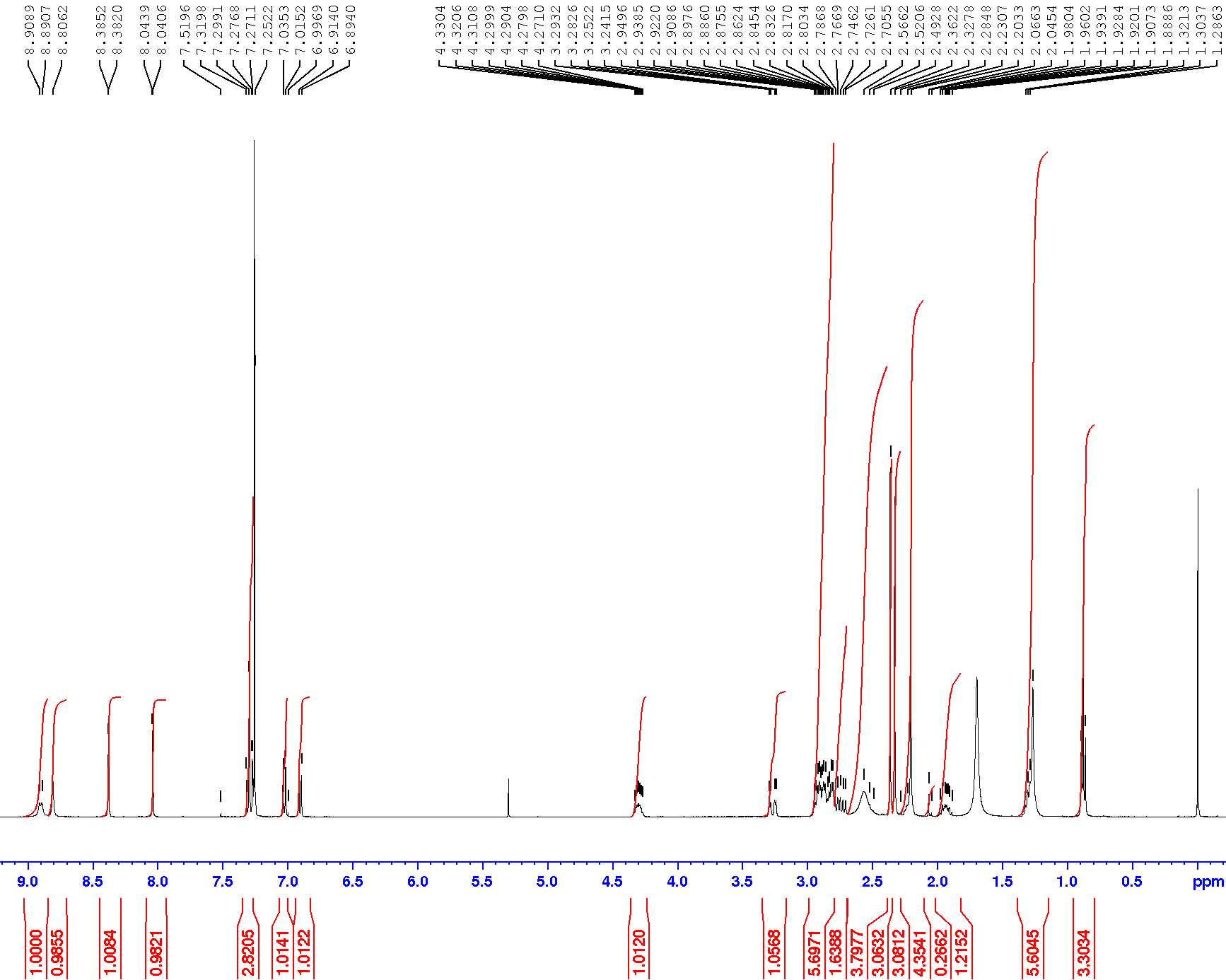


Compound **38**


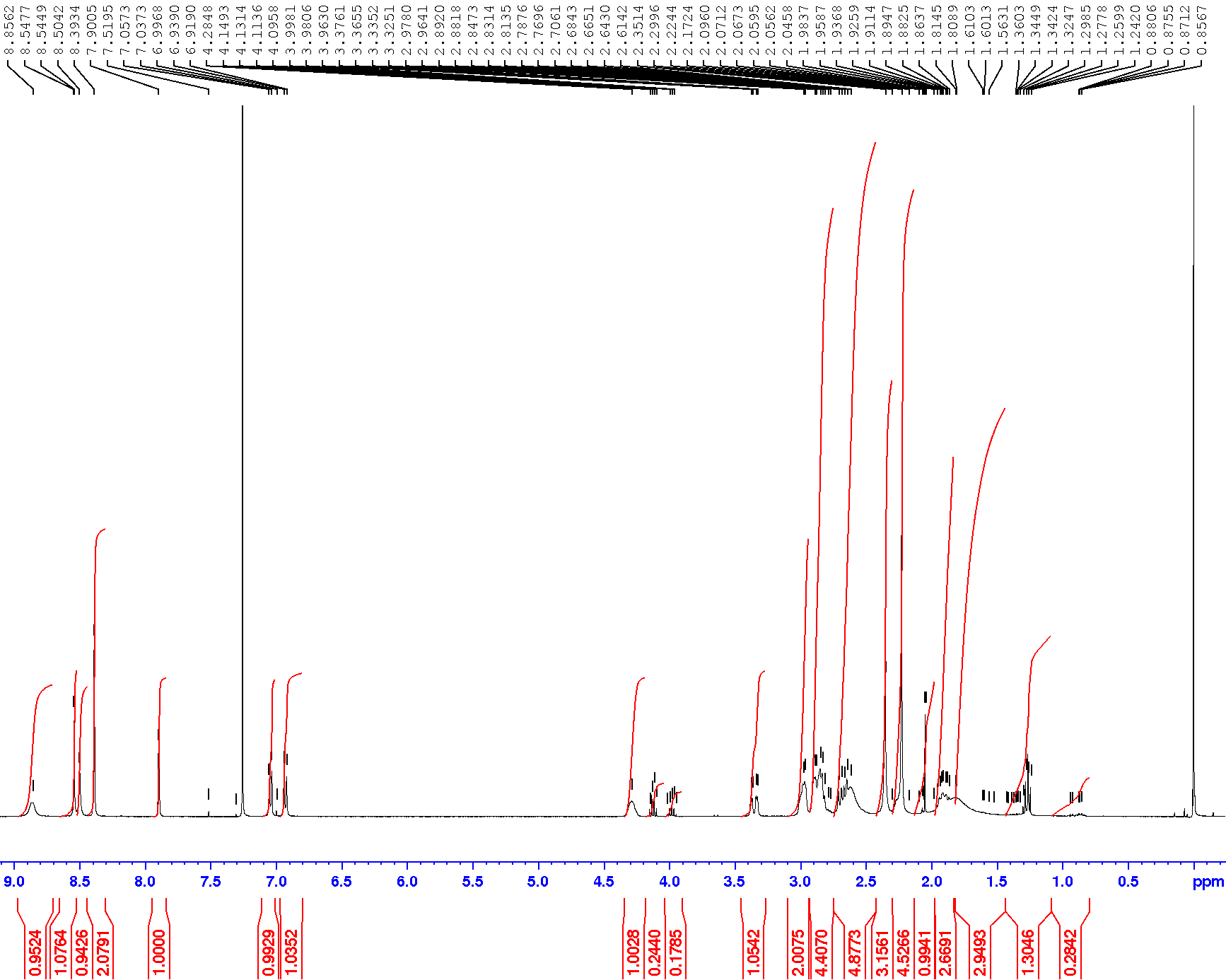


Compound **39**


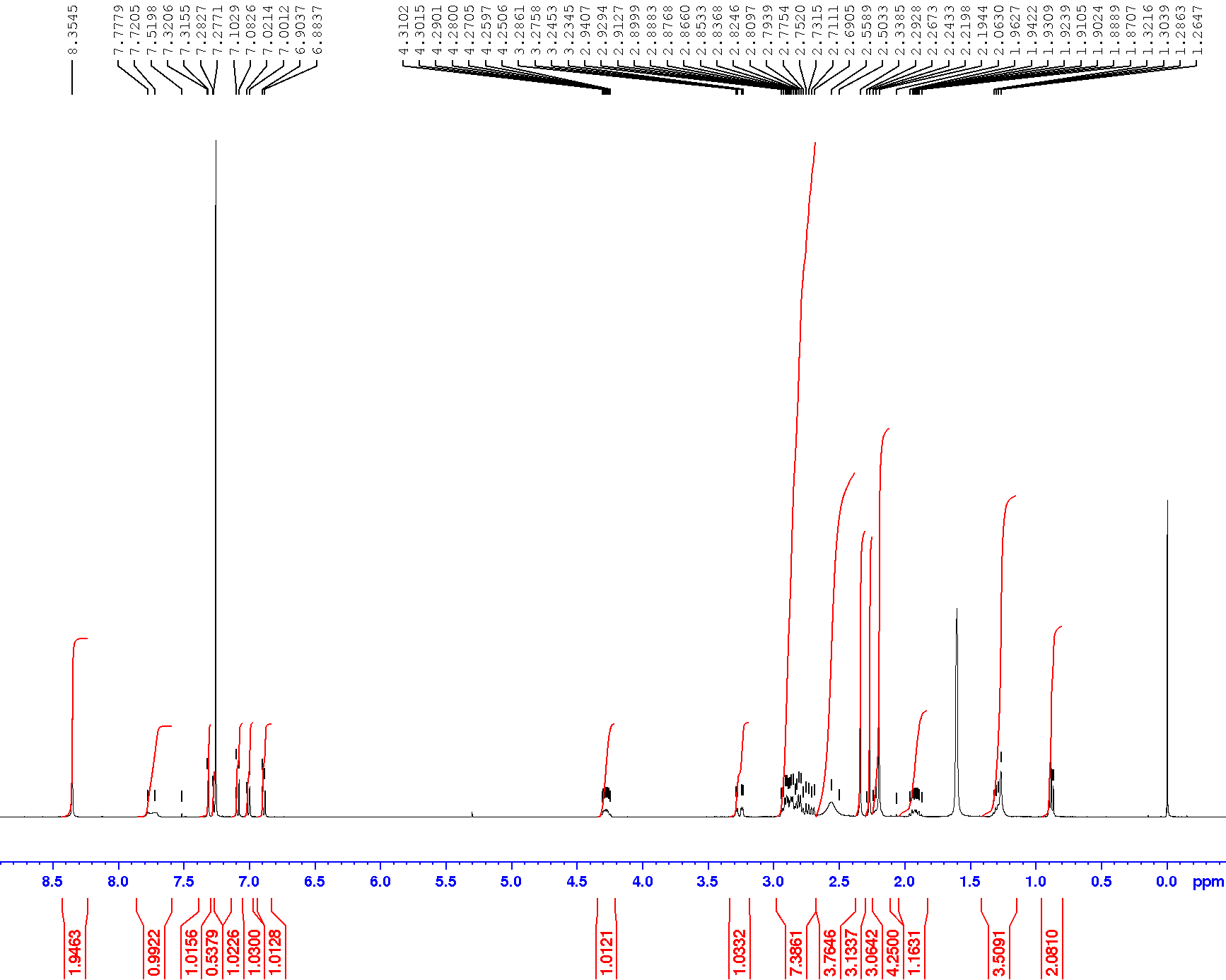


Compound **40**


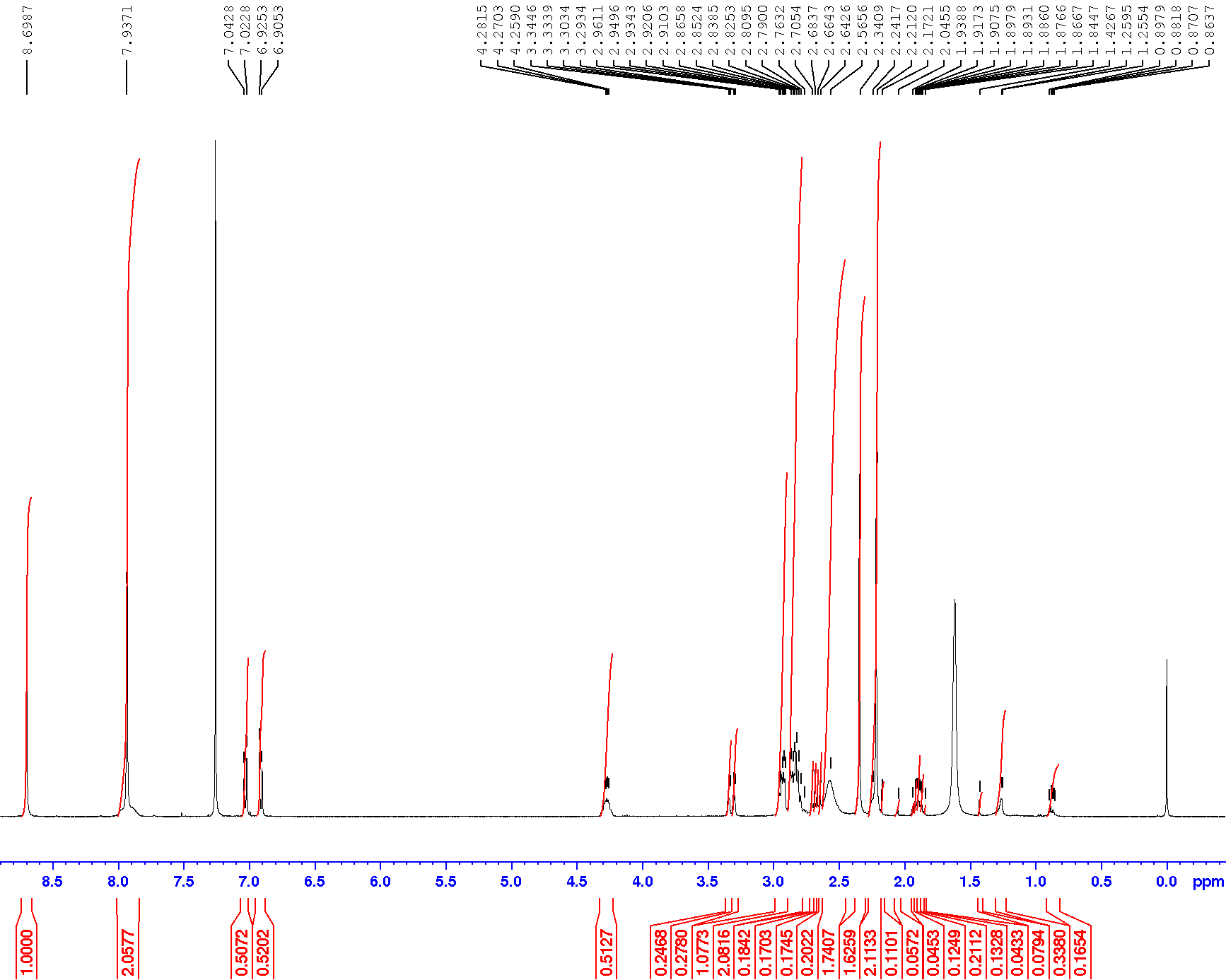


Compound **41**


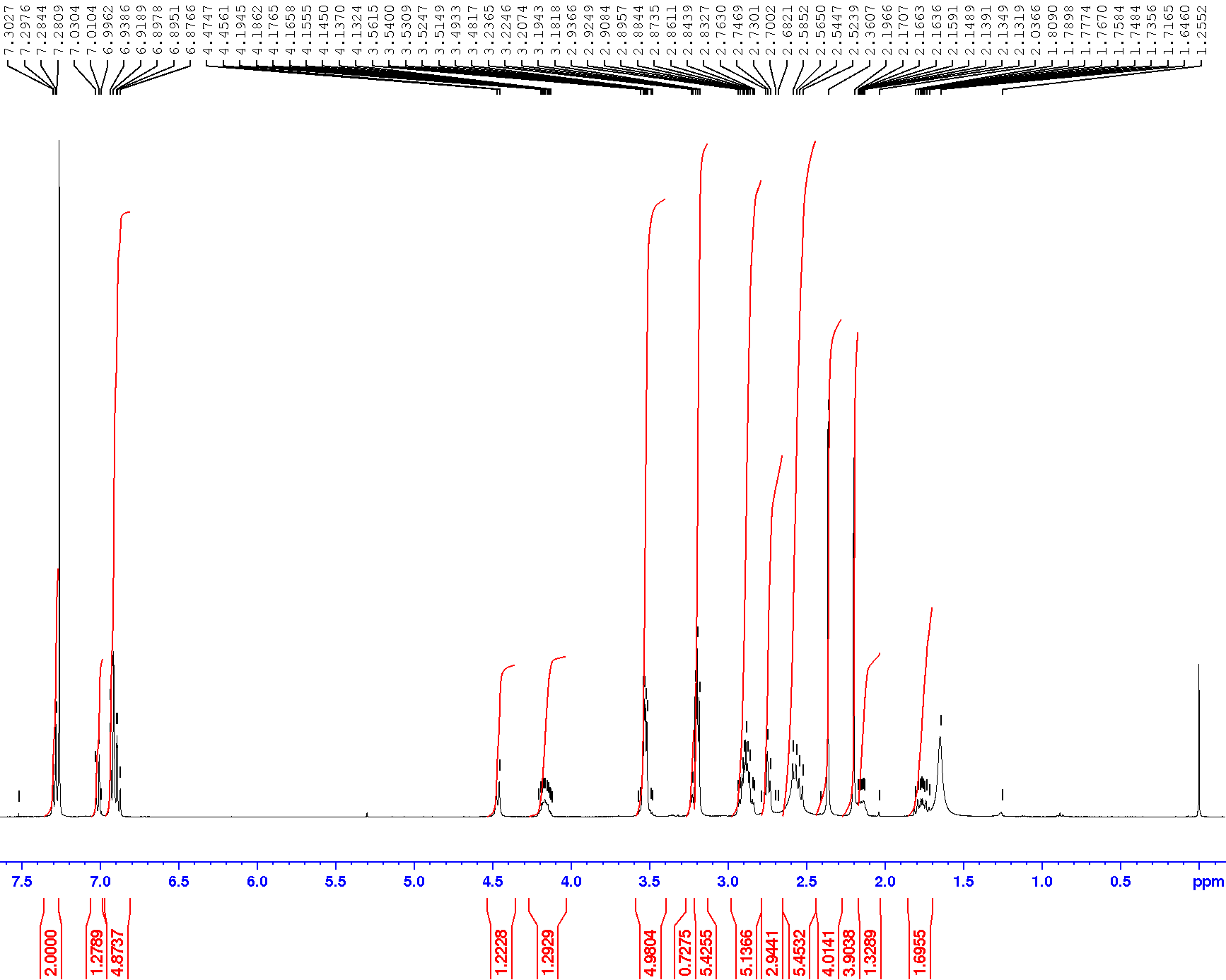


Compound **42**


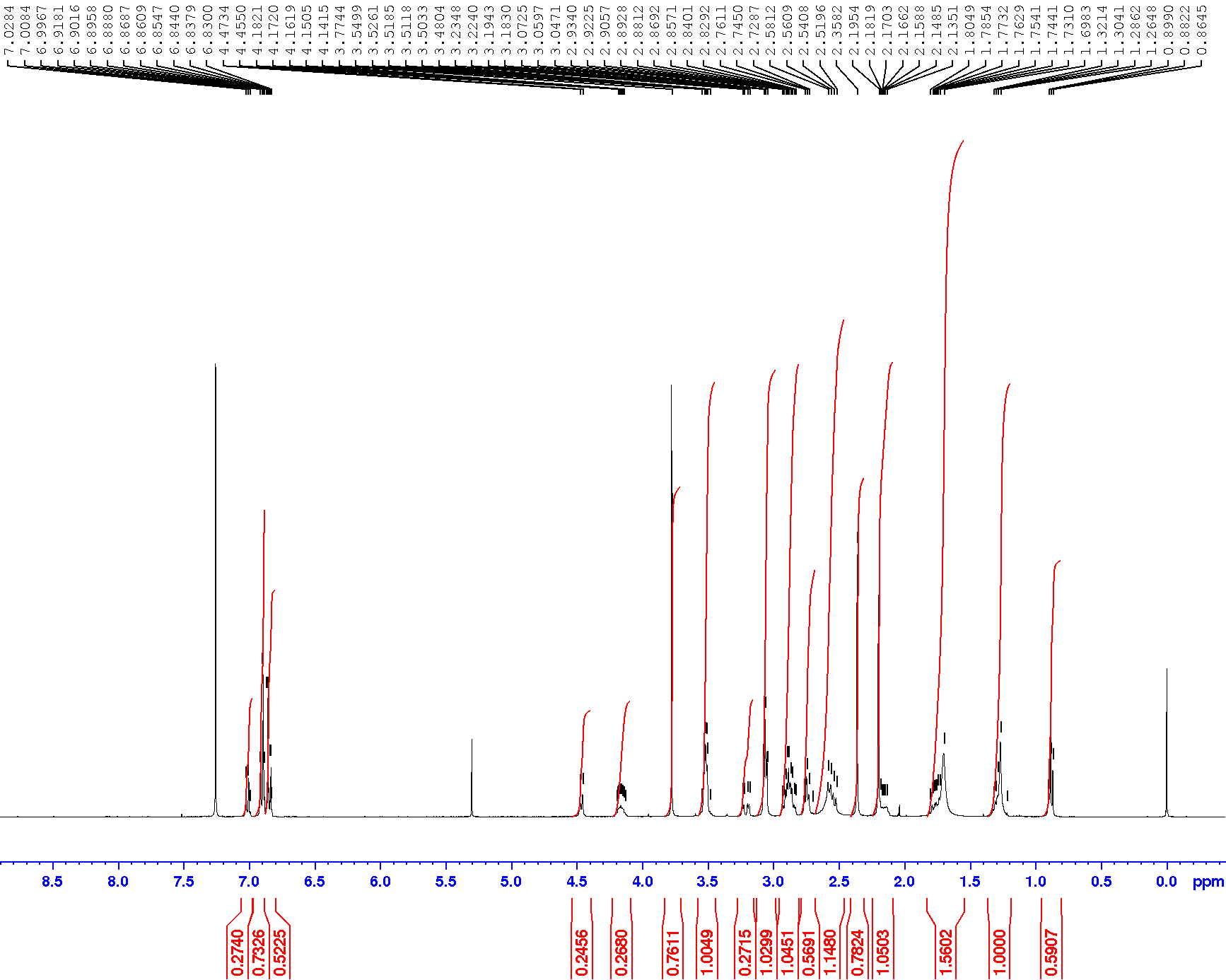


Compound **43**


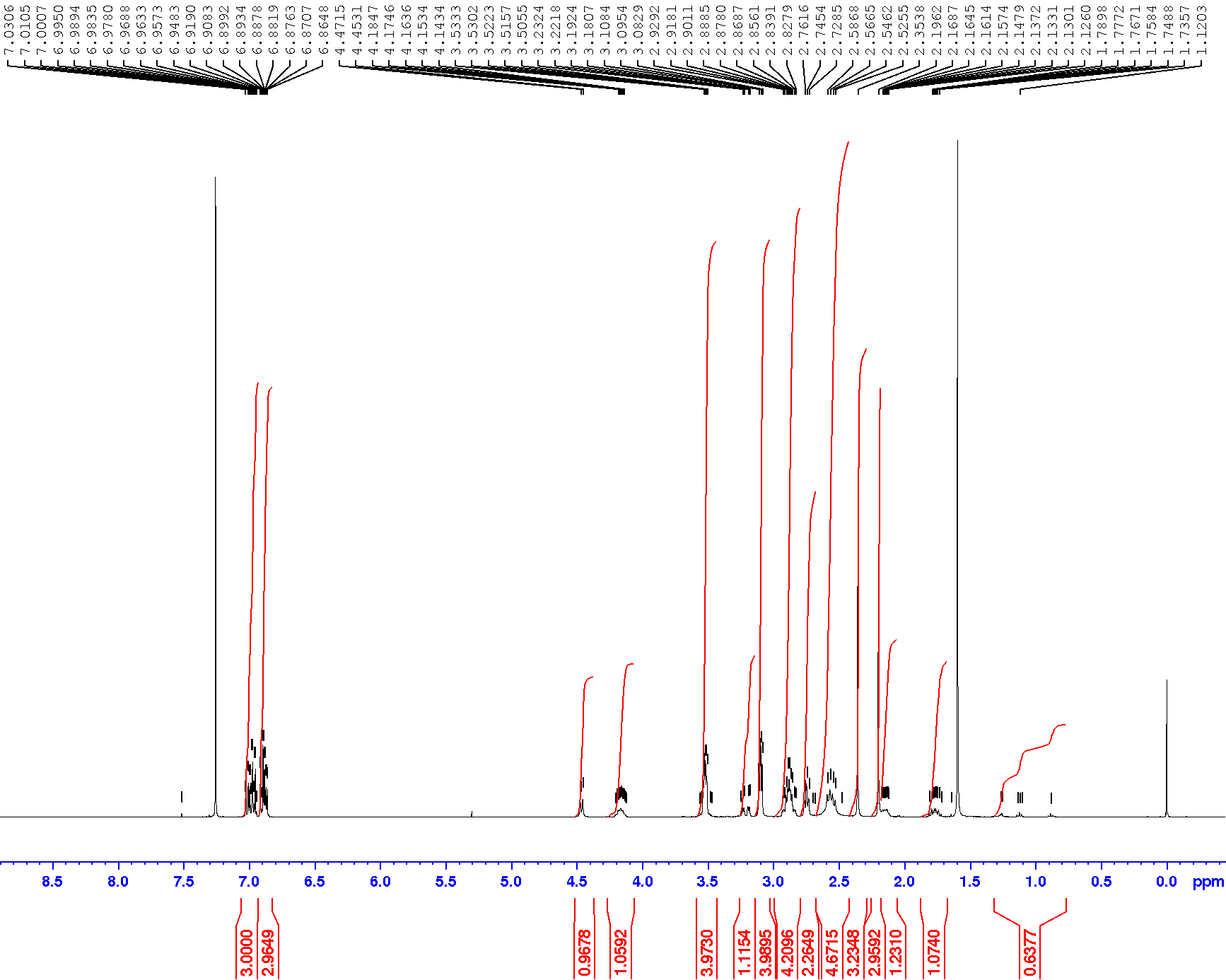

Compound **44**


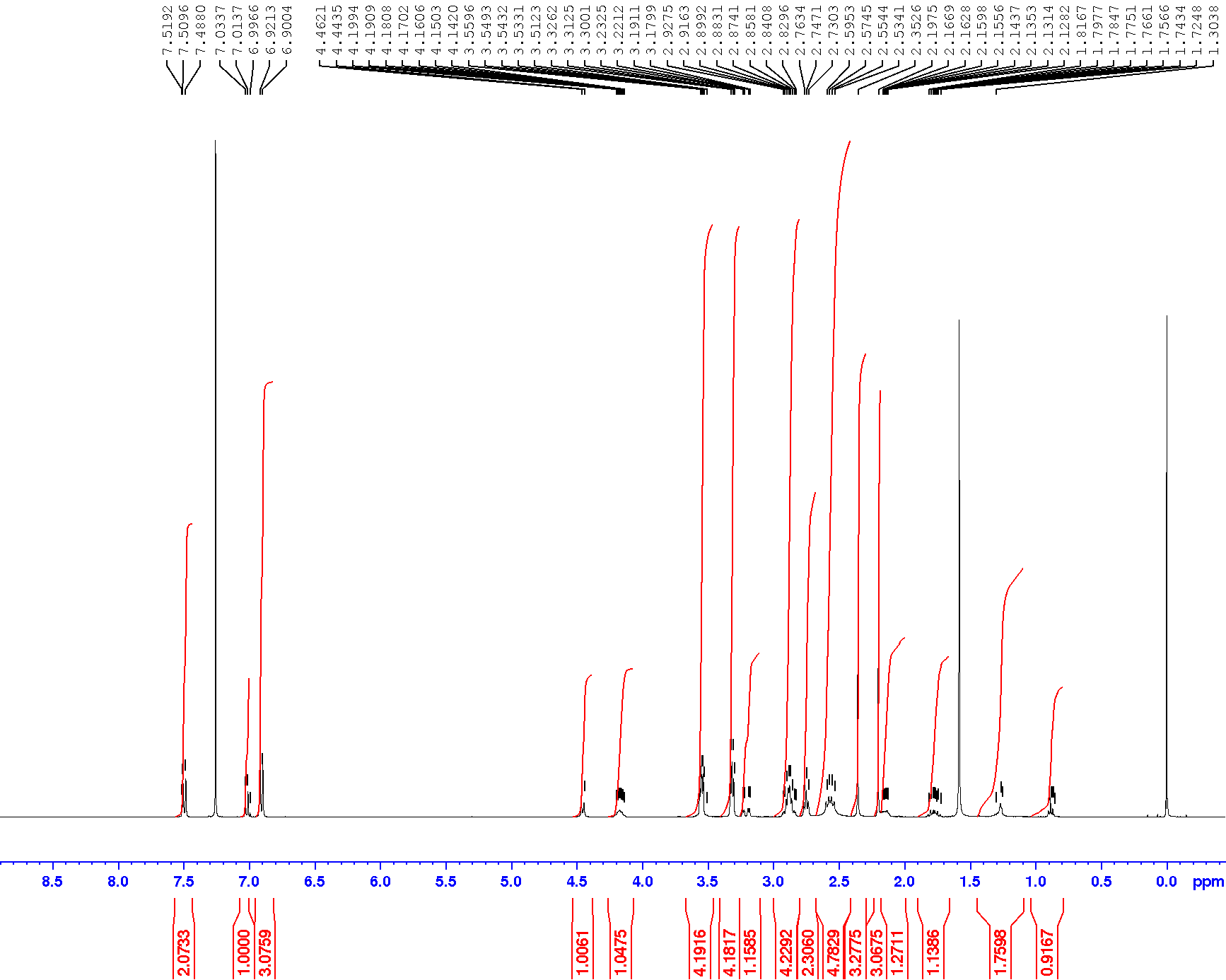


Compound **45**


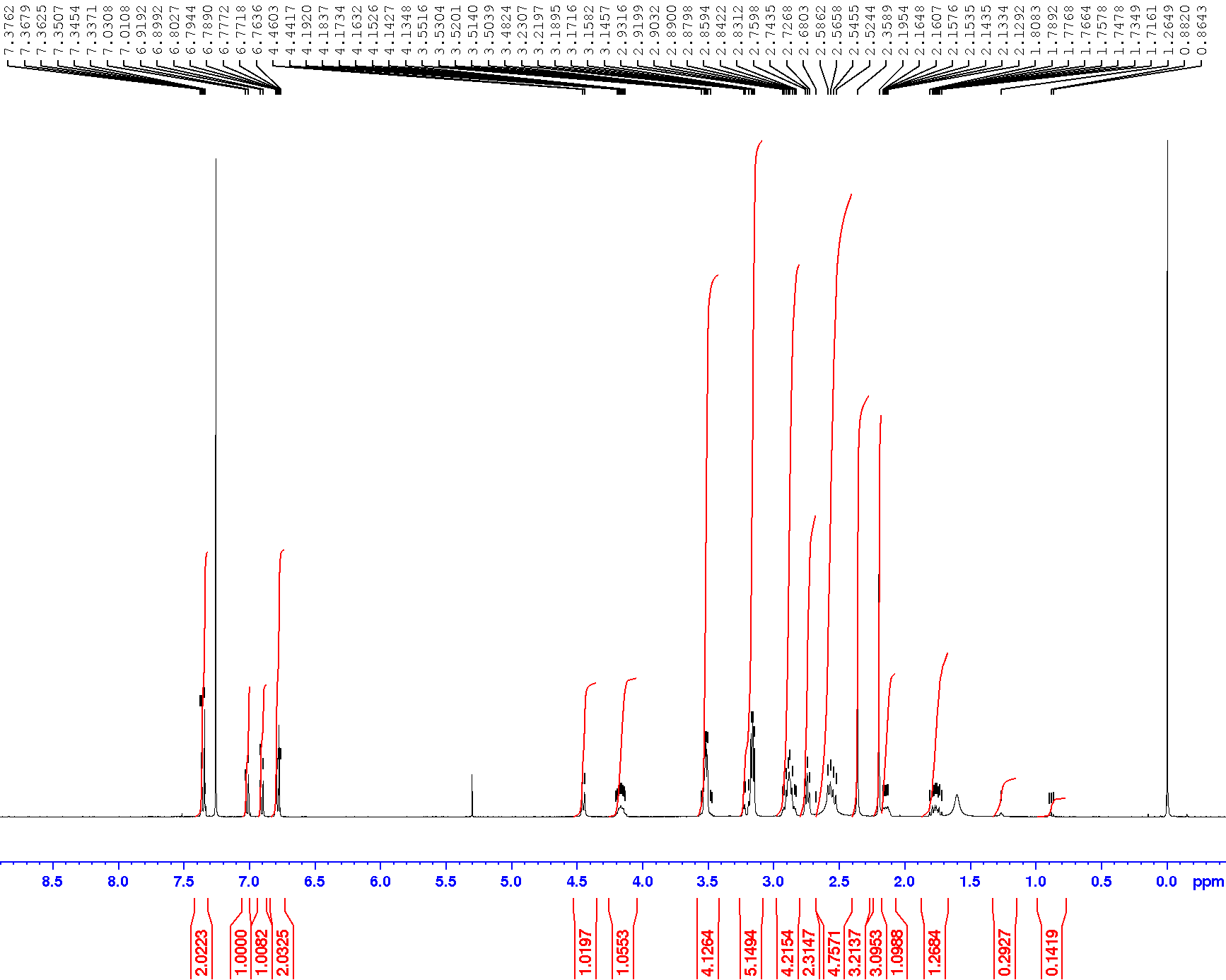


Compound **46**


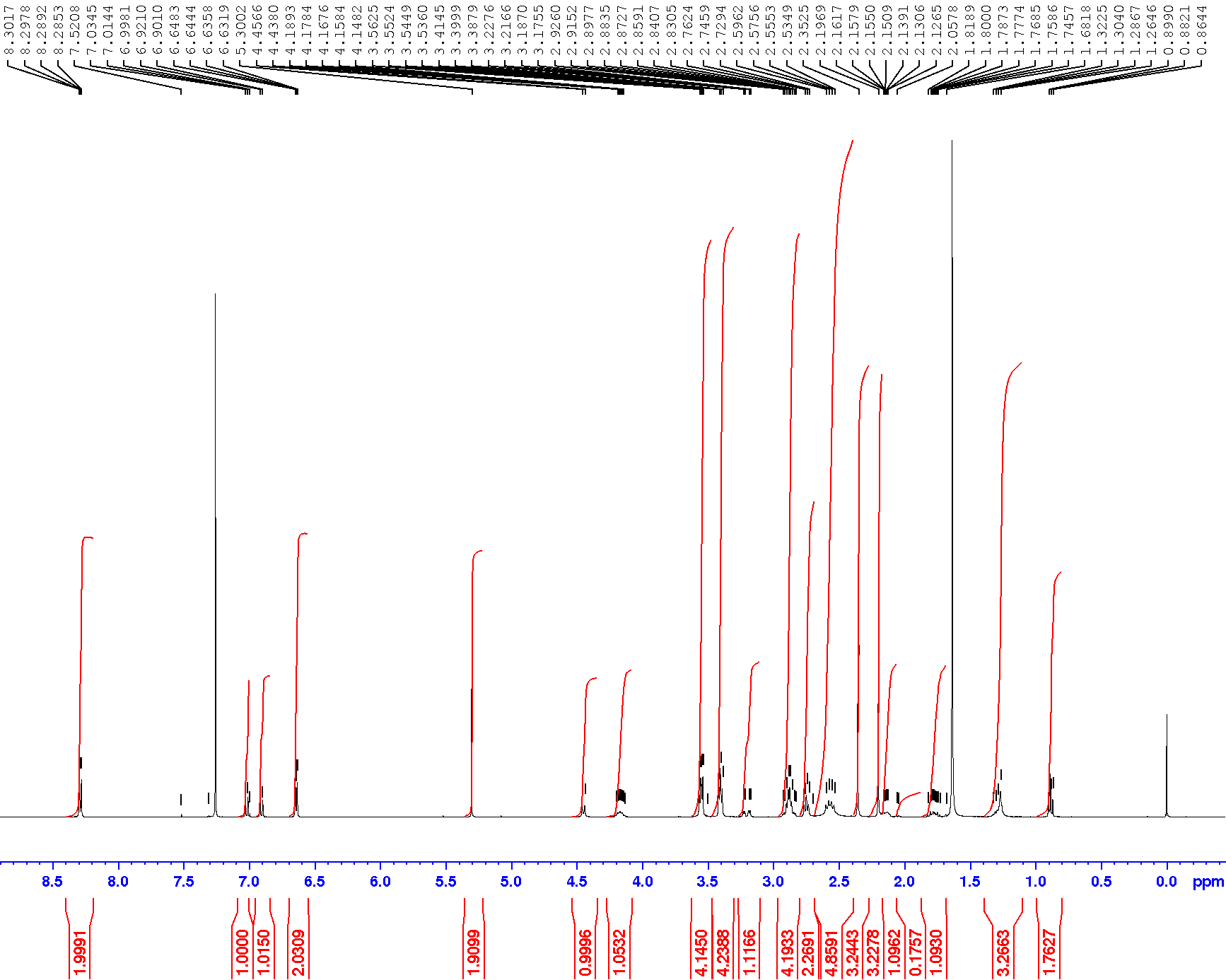


Compound **47**


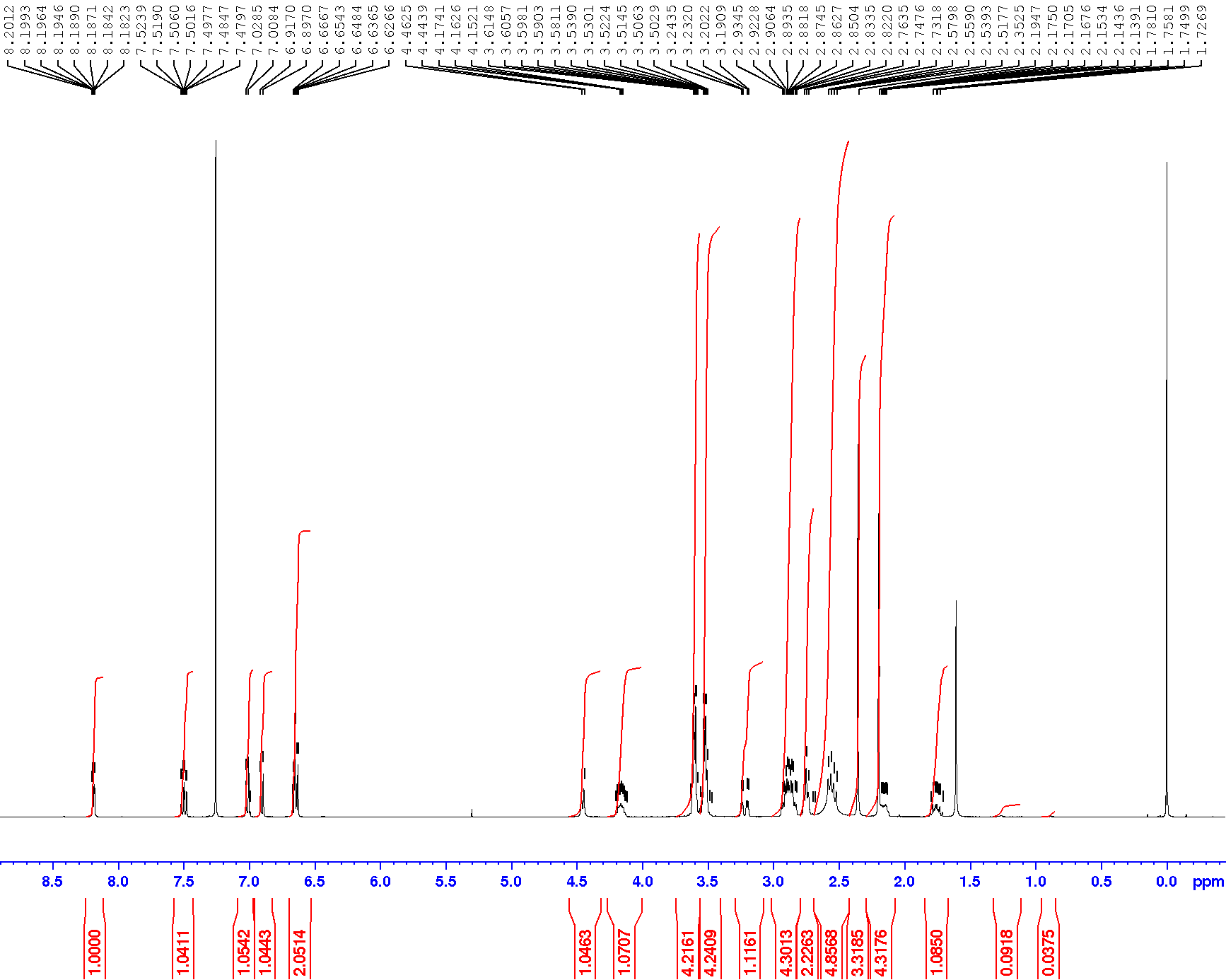


Compound **48**


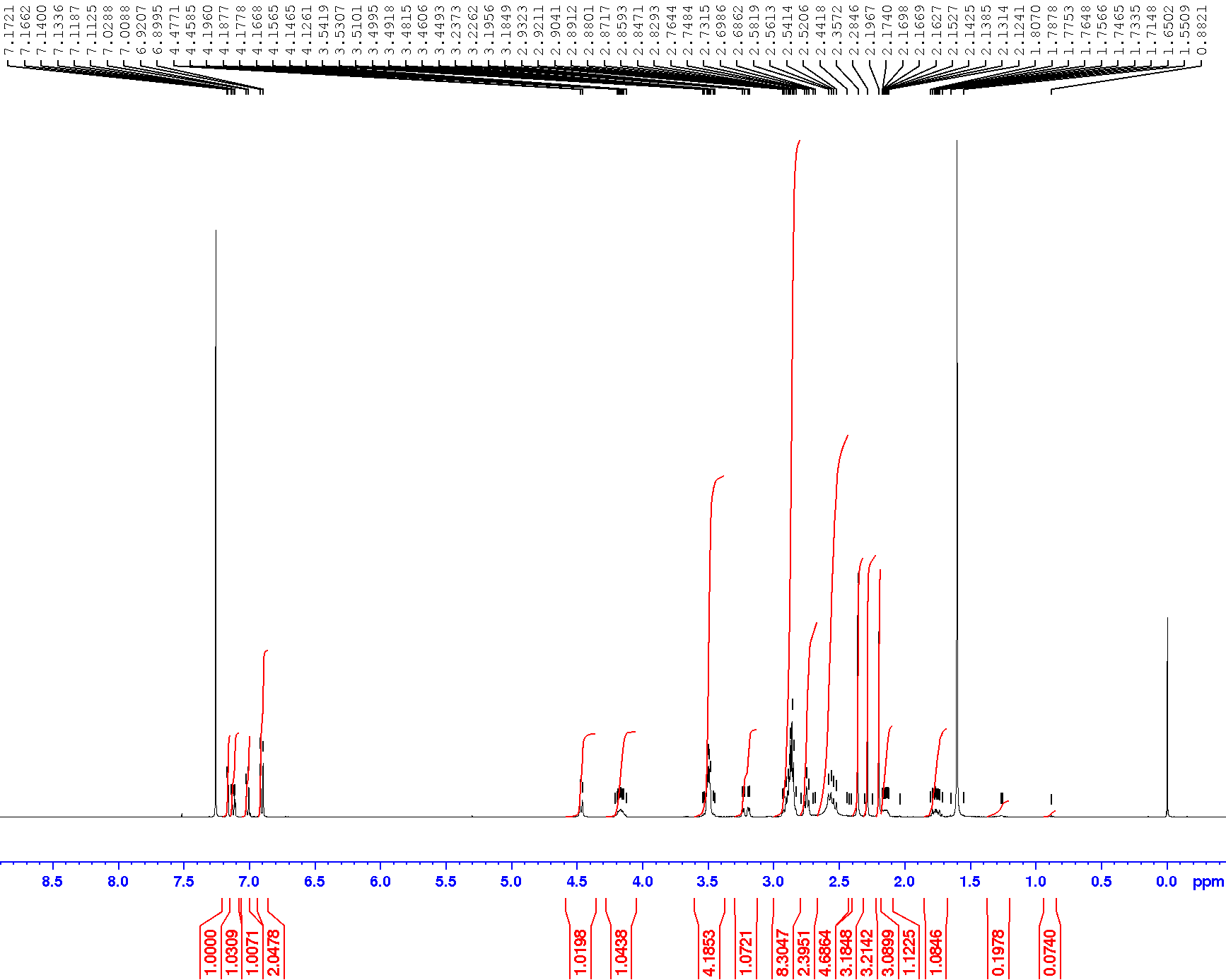


Compound **49**


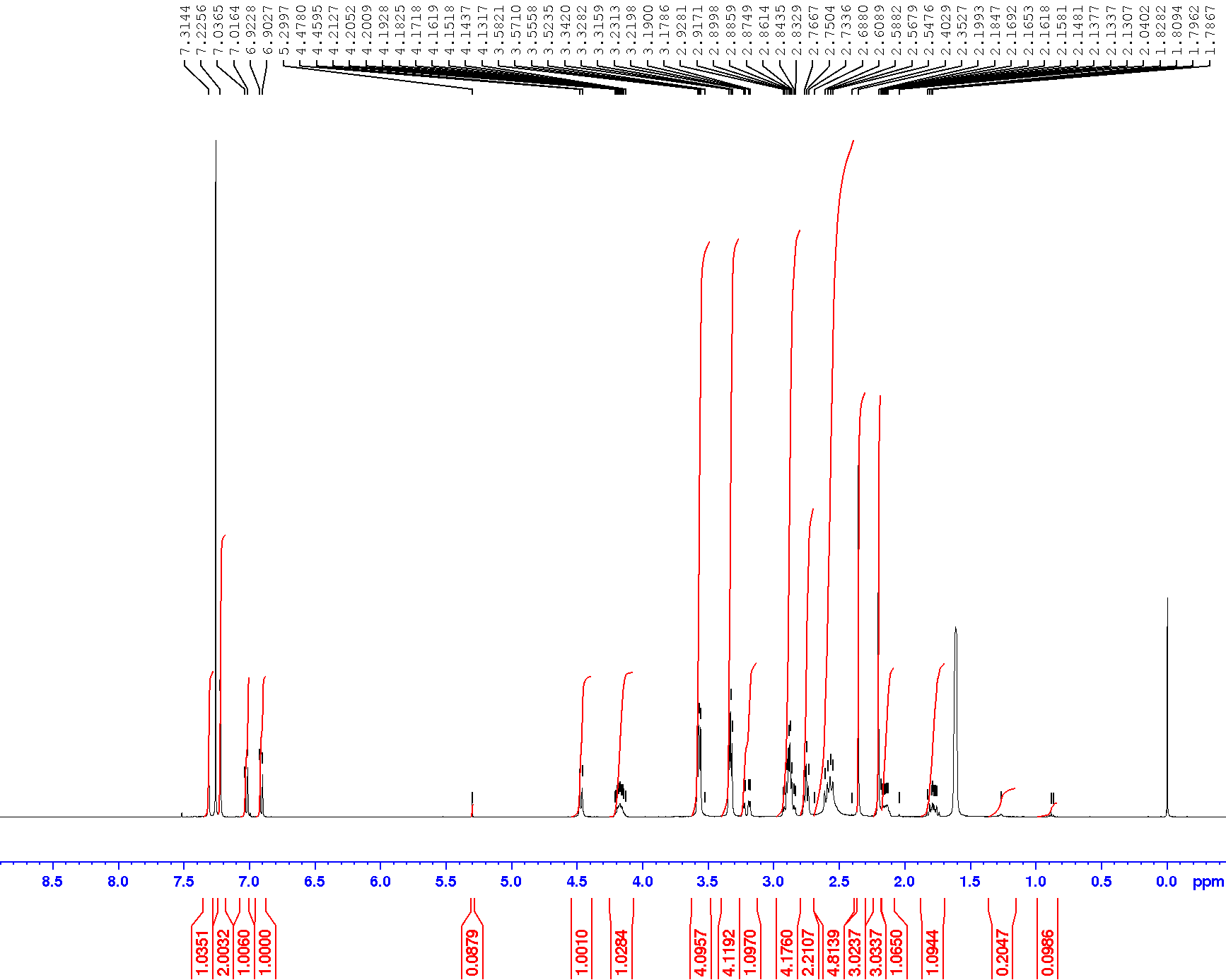


Compound **50**


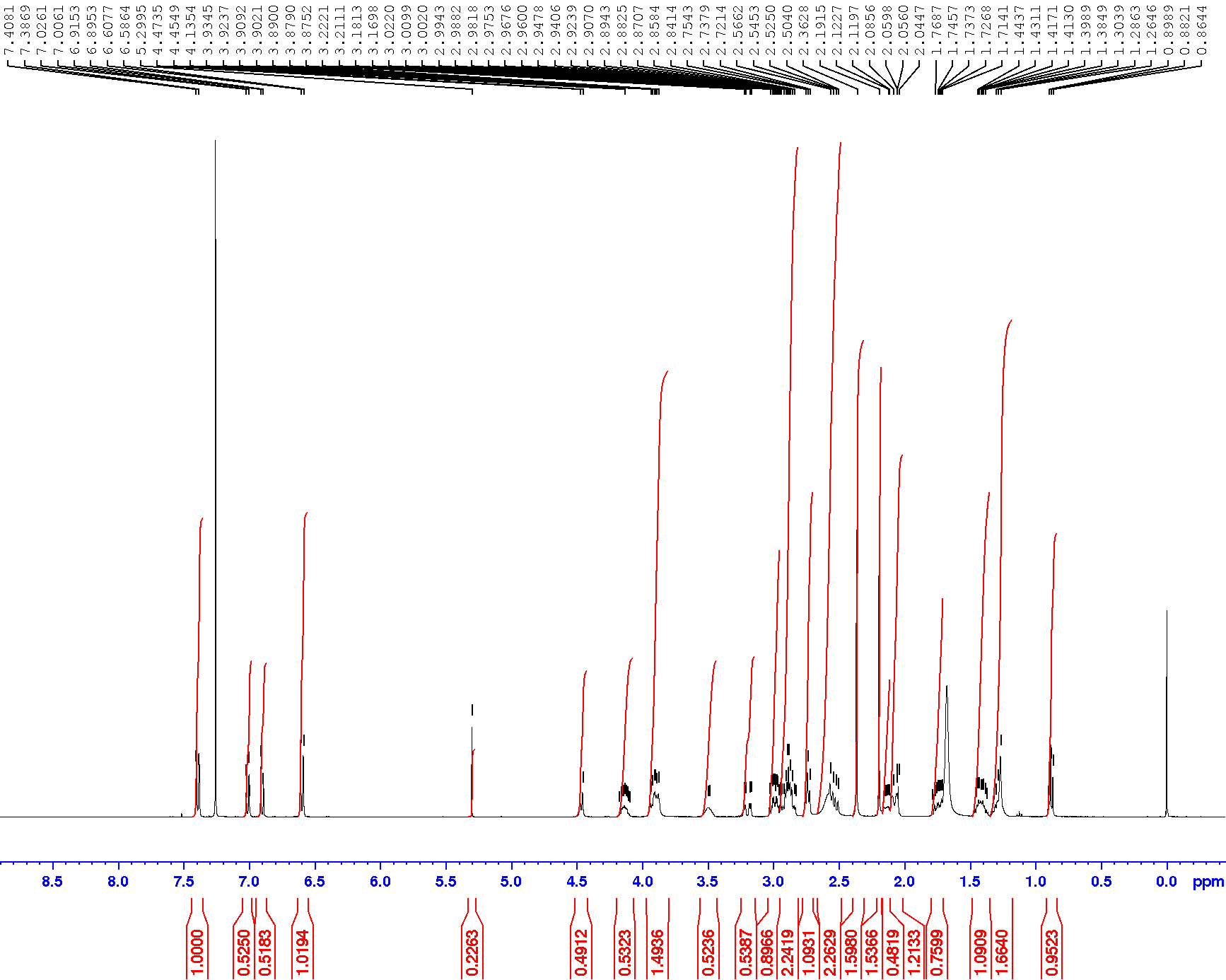


Compound **51**


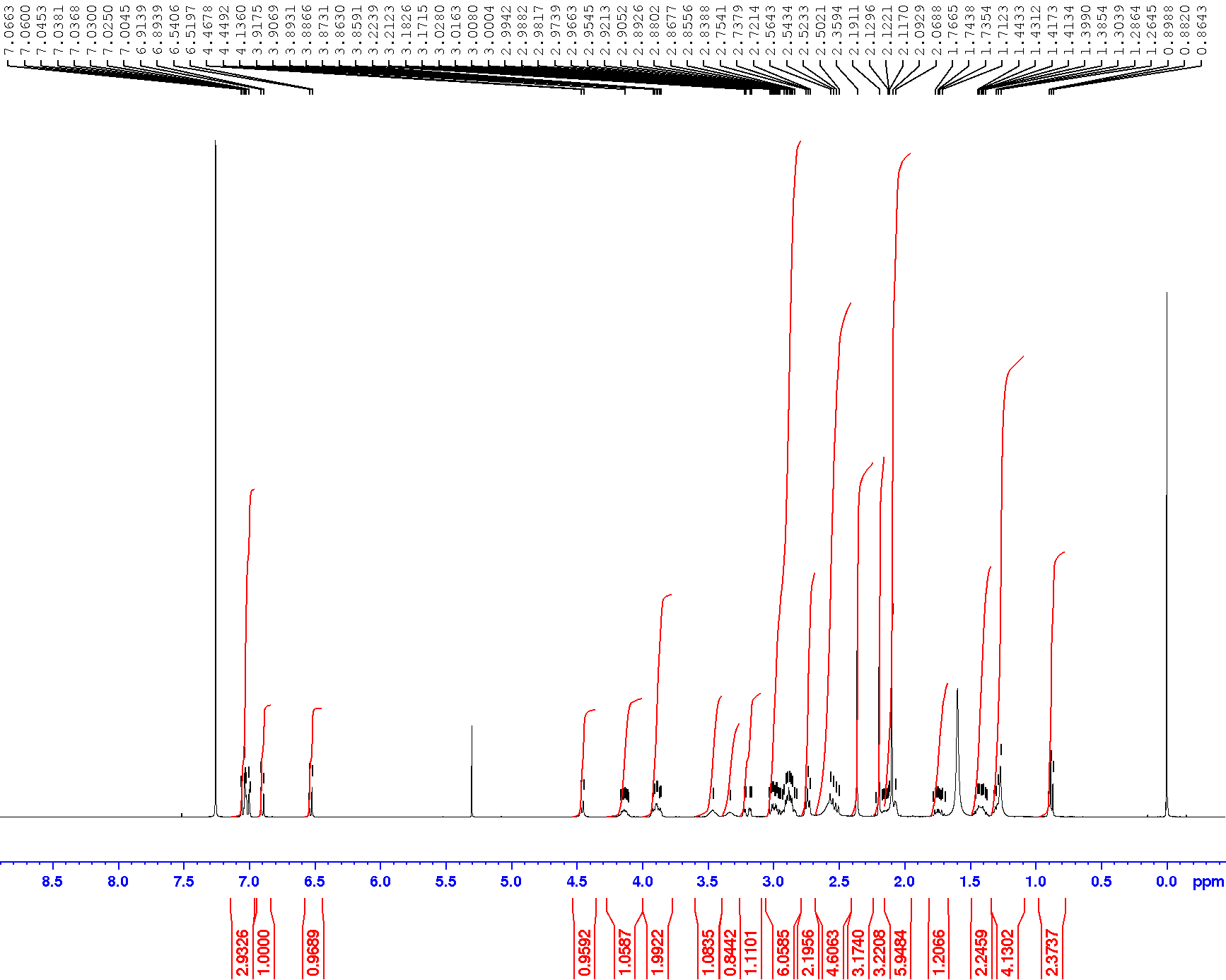


Compound **52**


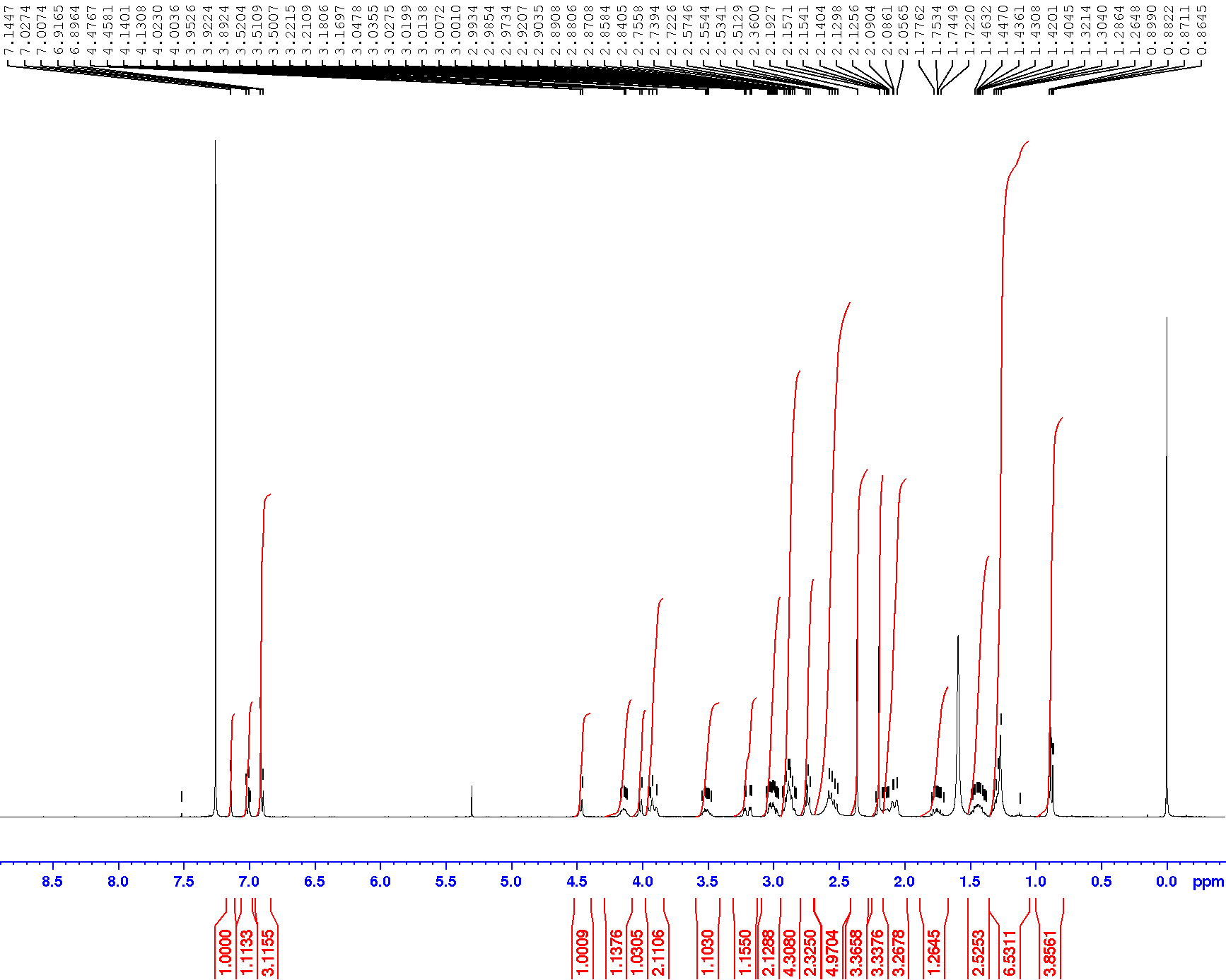


Compound **53**


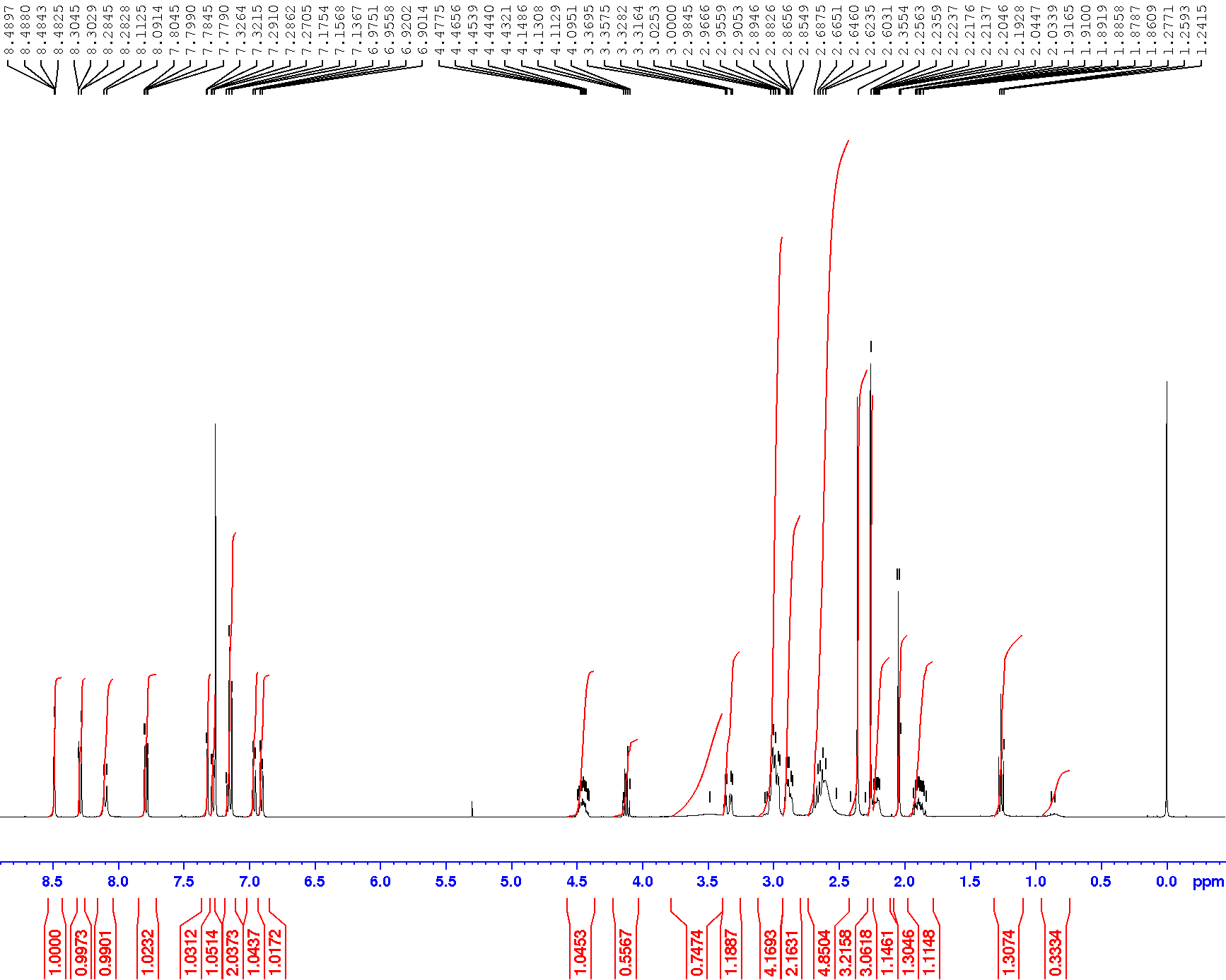

Compound **54**


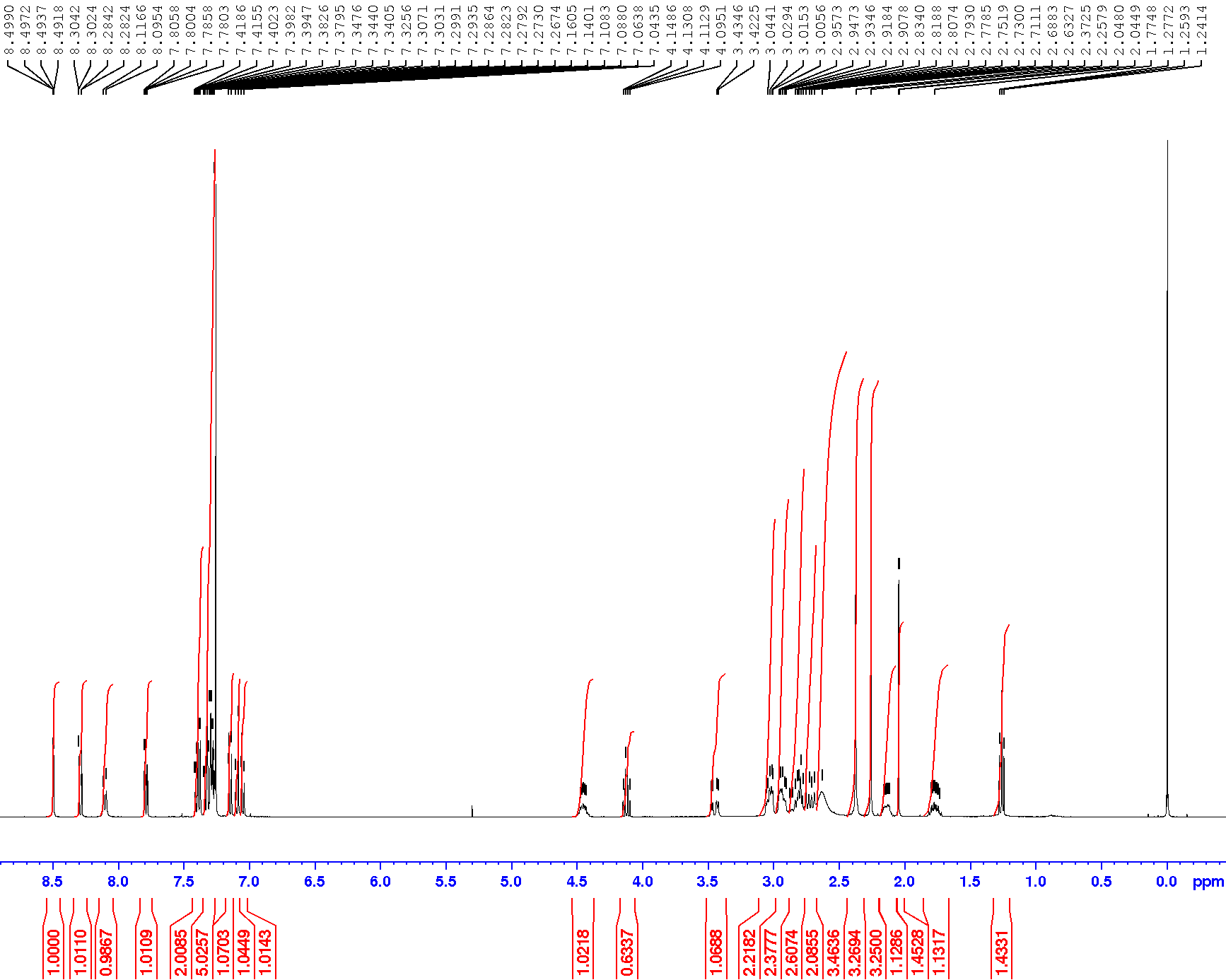

Compound **55**


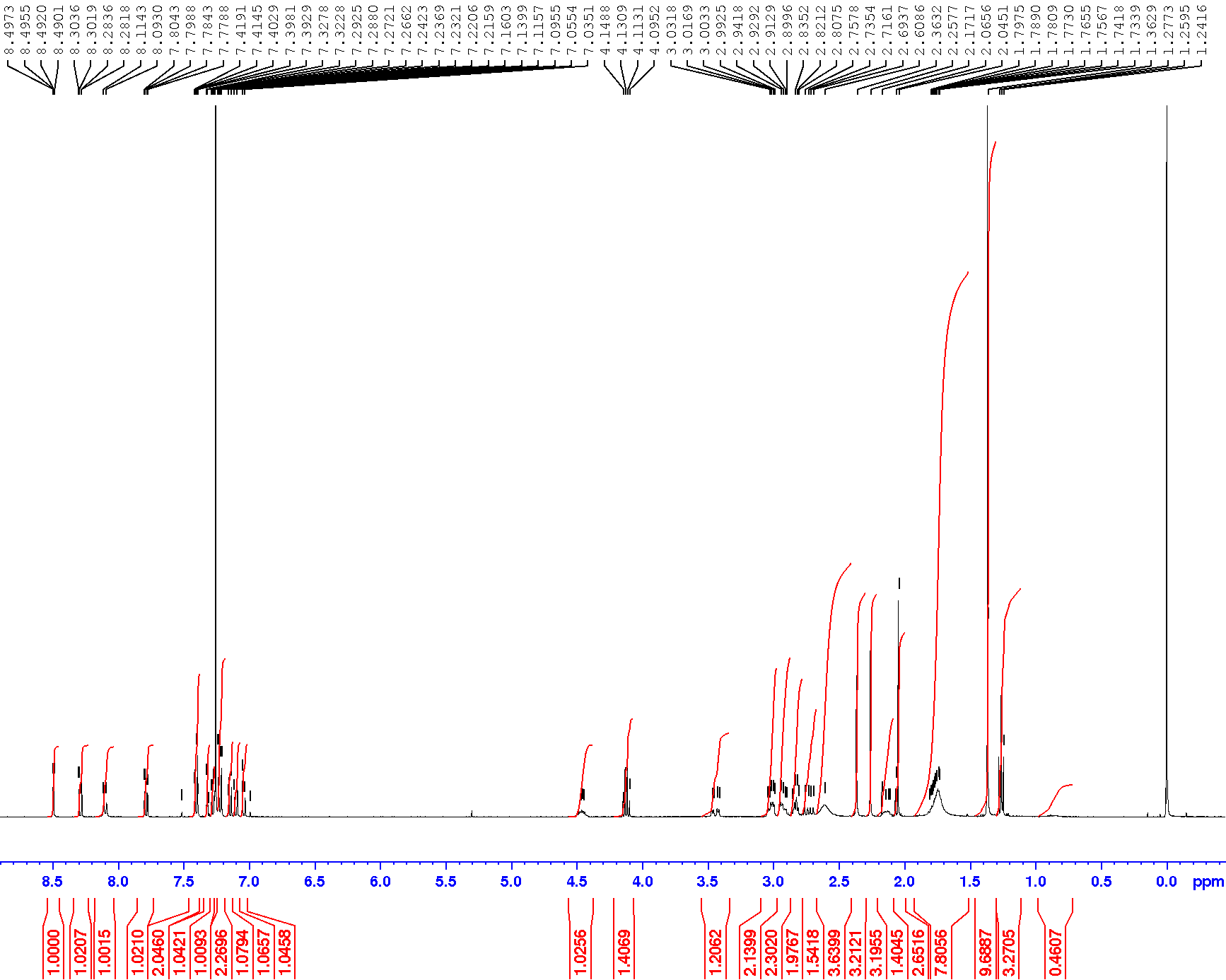


Compound **56**


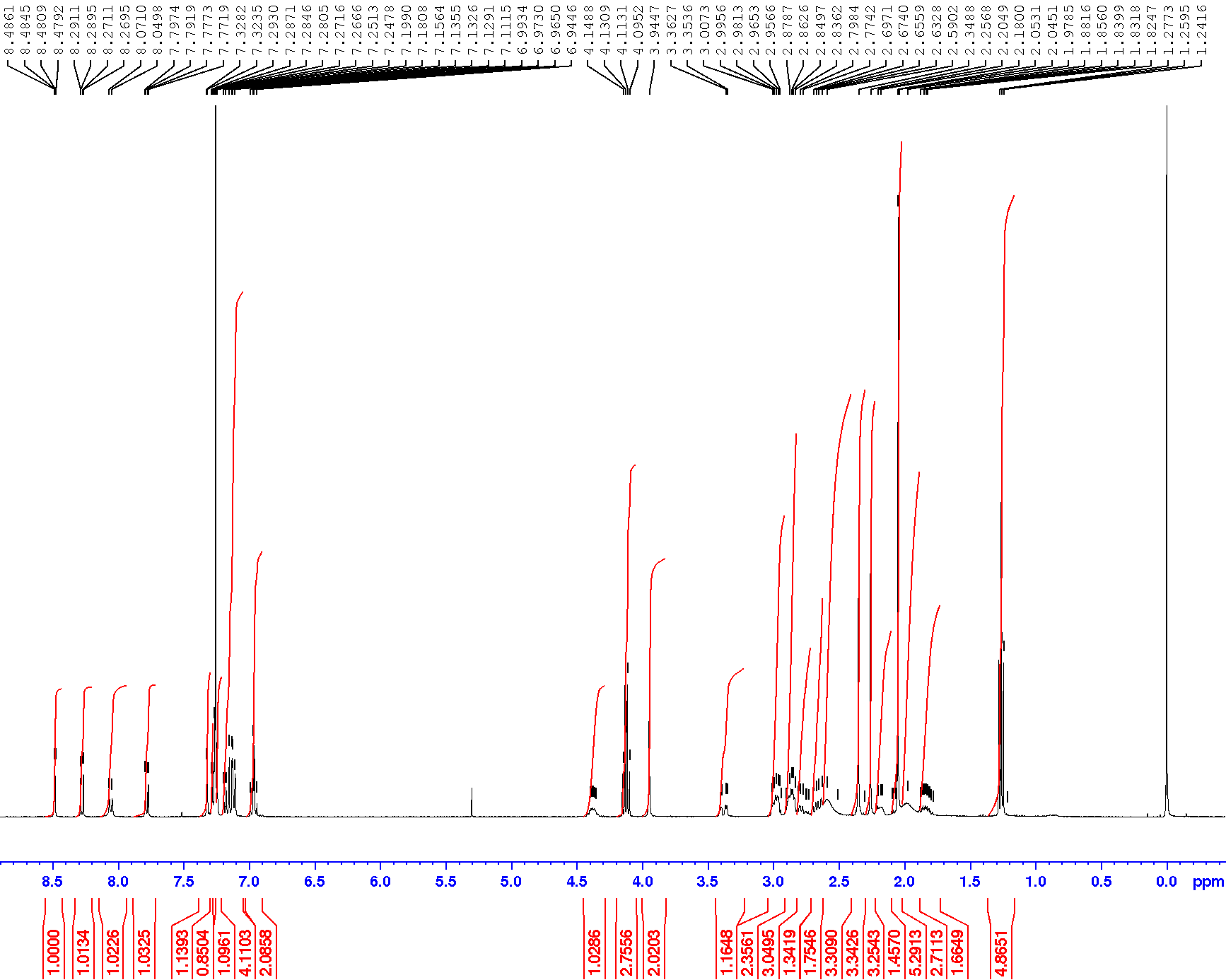

Compound **57**


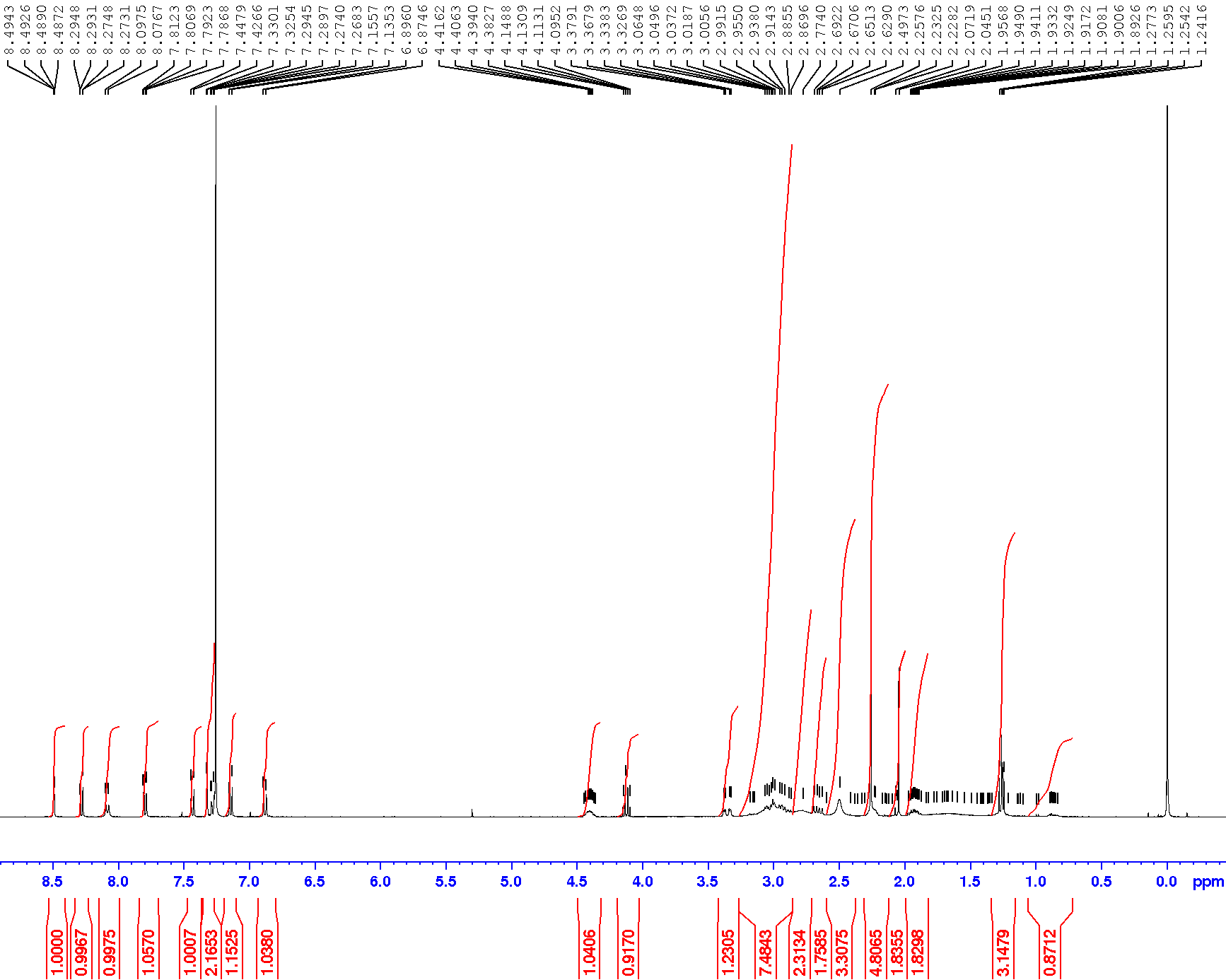


Compound **58**


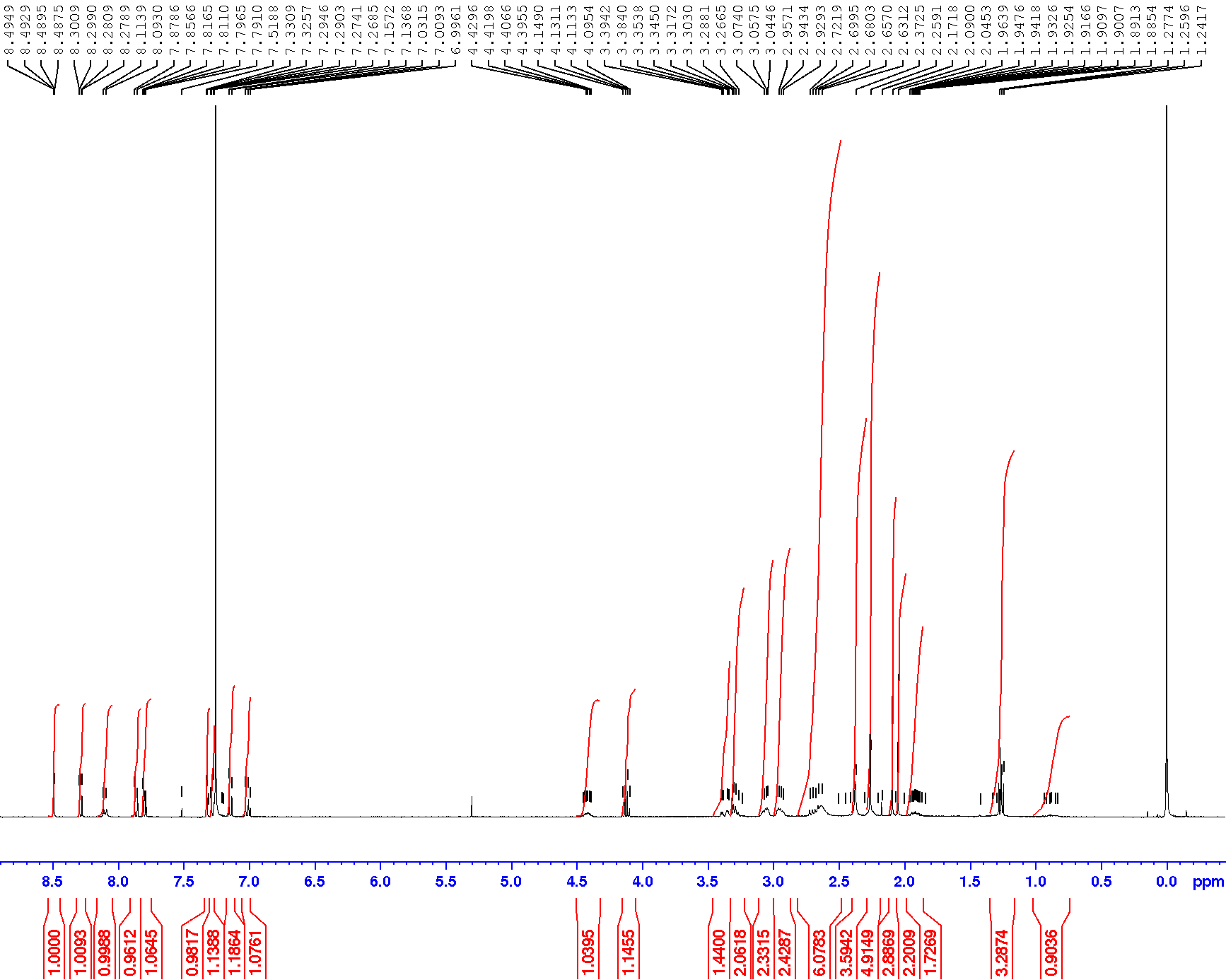


Compound **59**


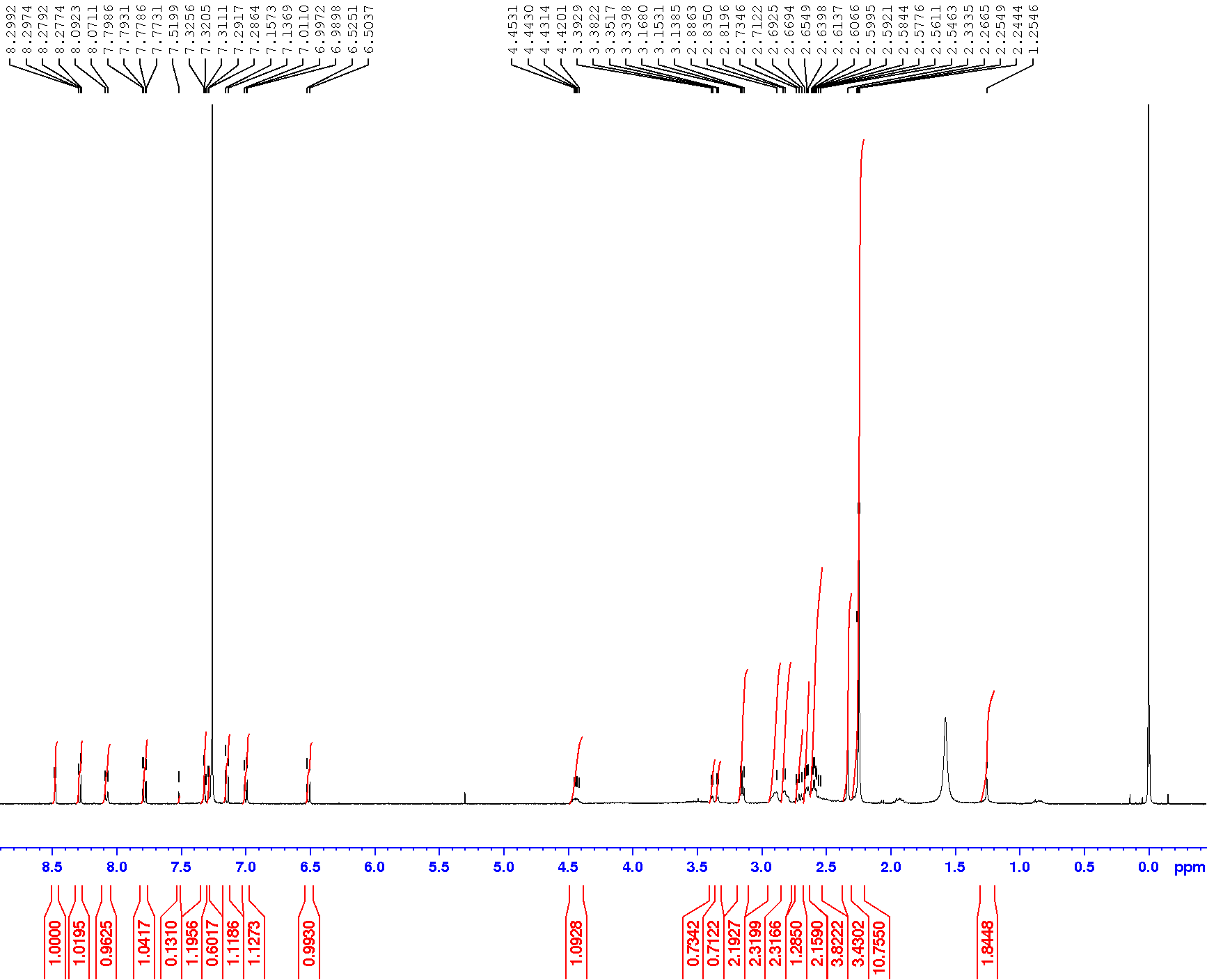


Compound **60**


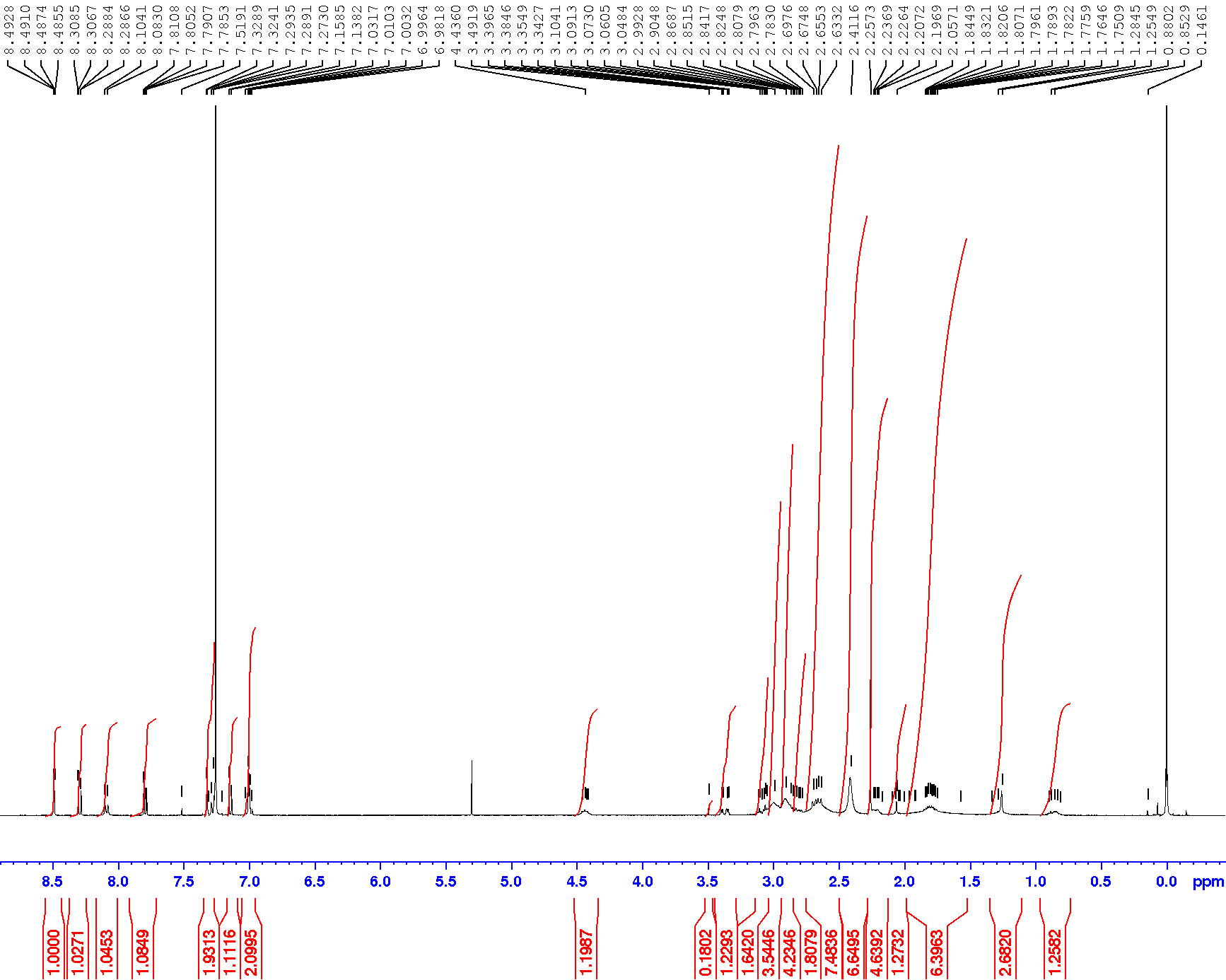


Compound **61**


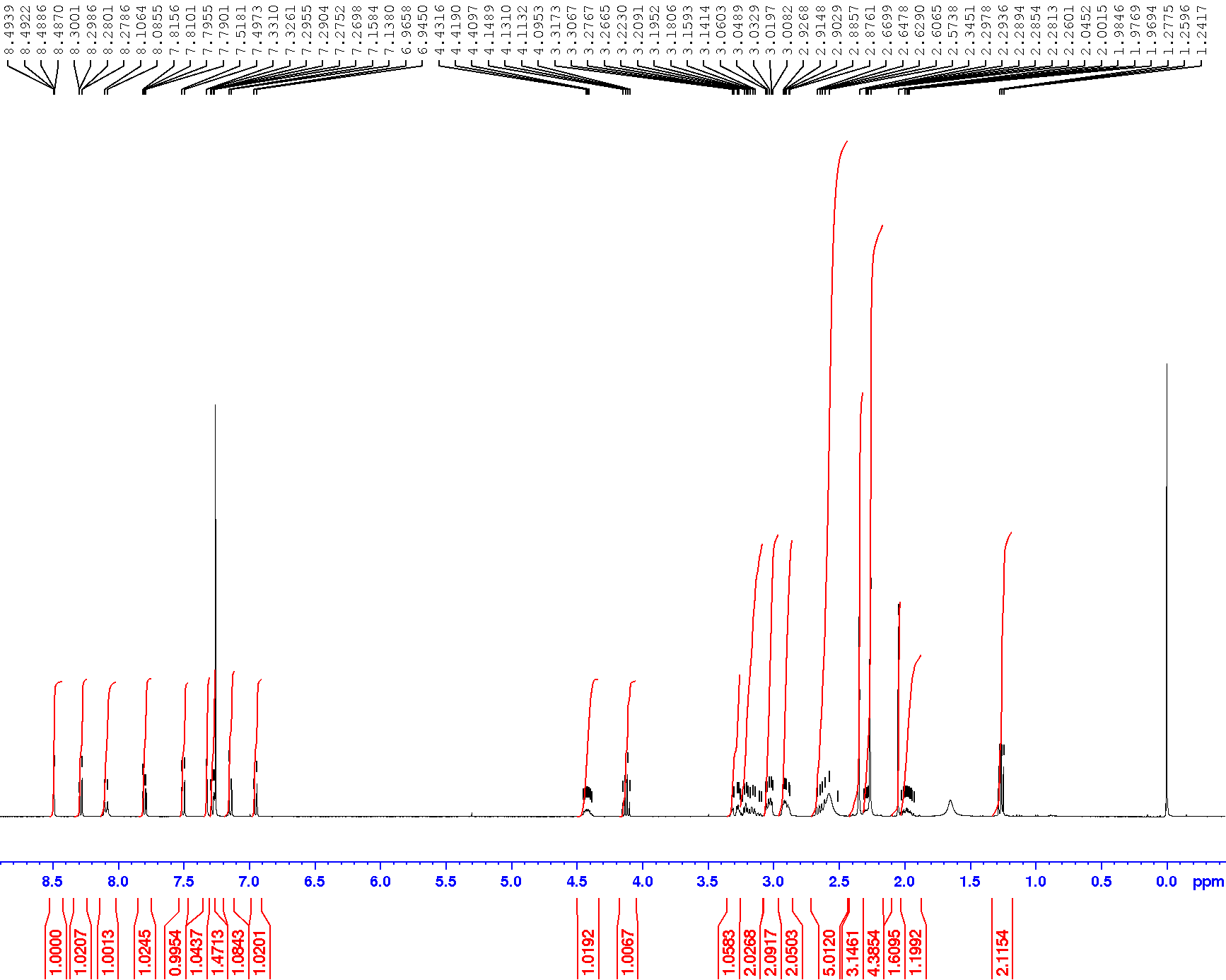


Compound **62**


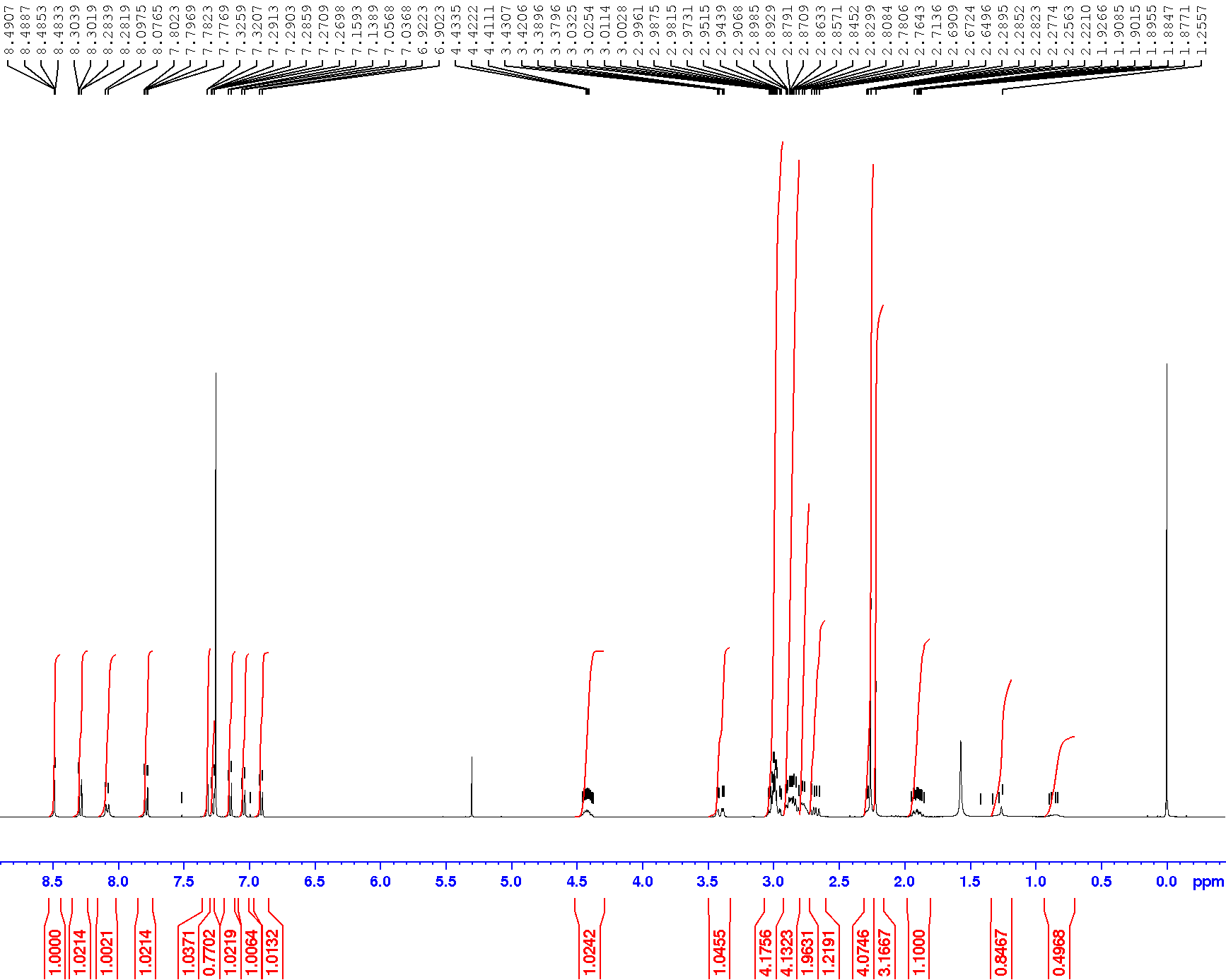


Compound **63**


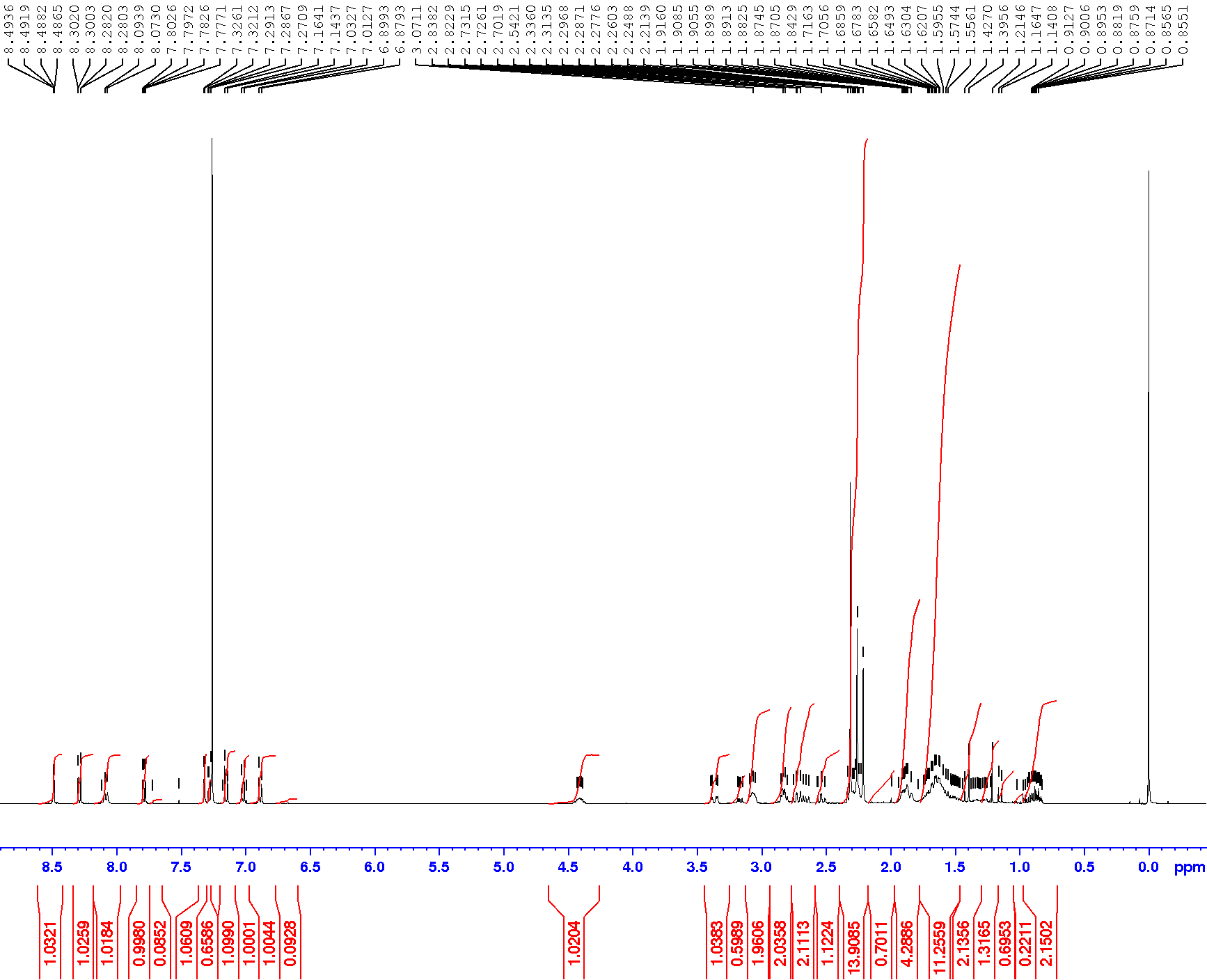

Compound **64**


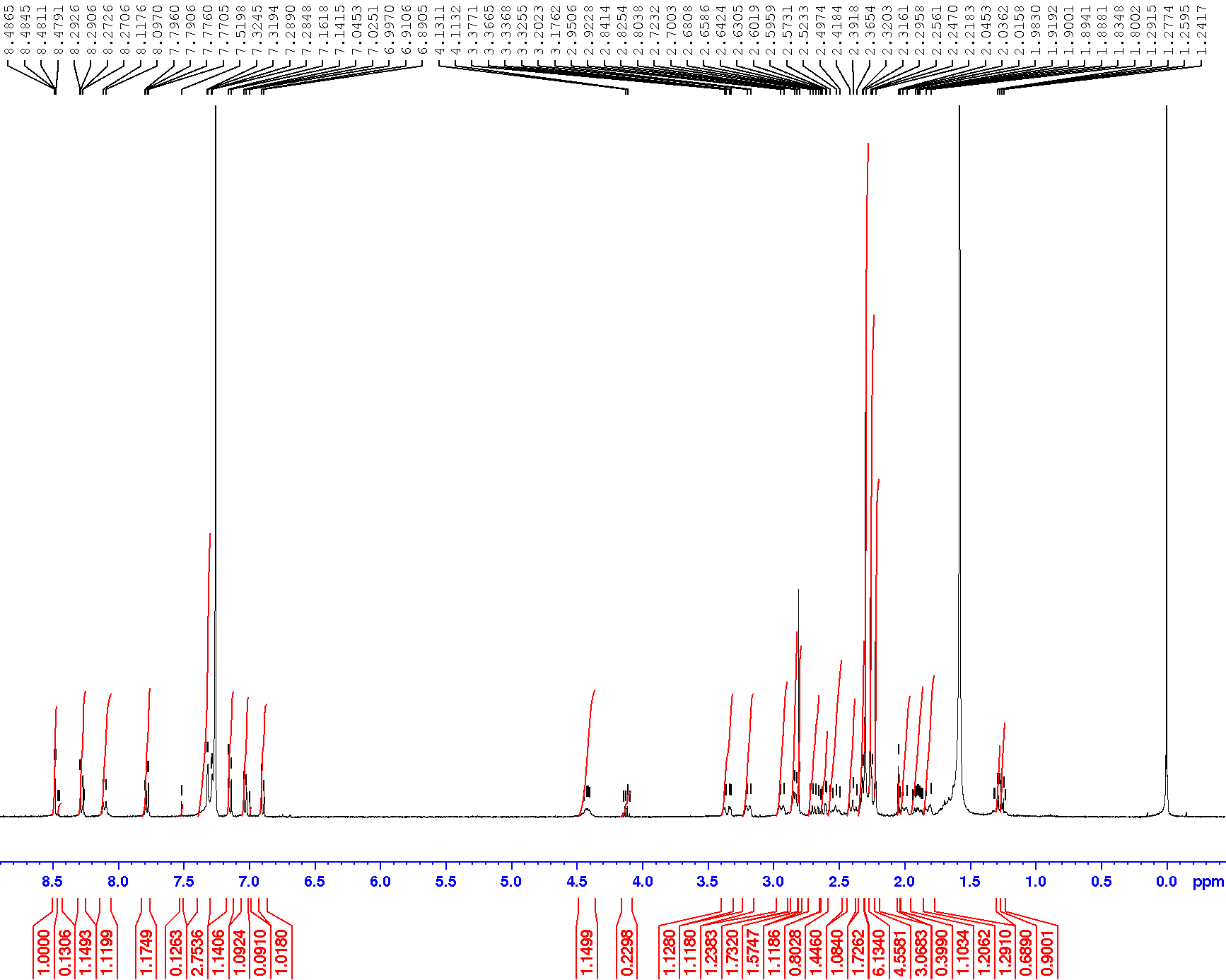


Compound **65**


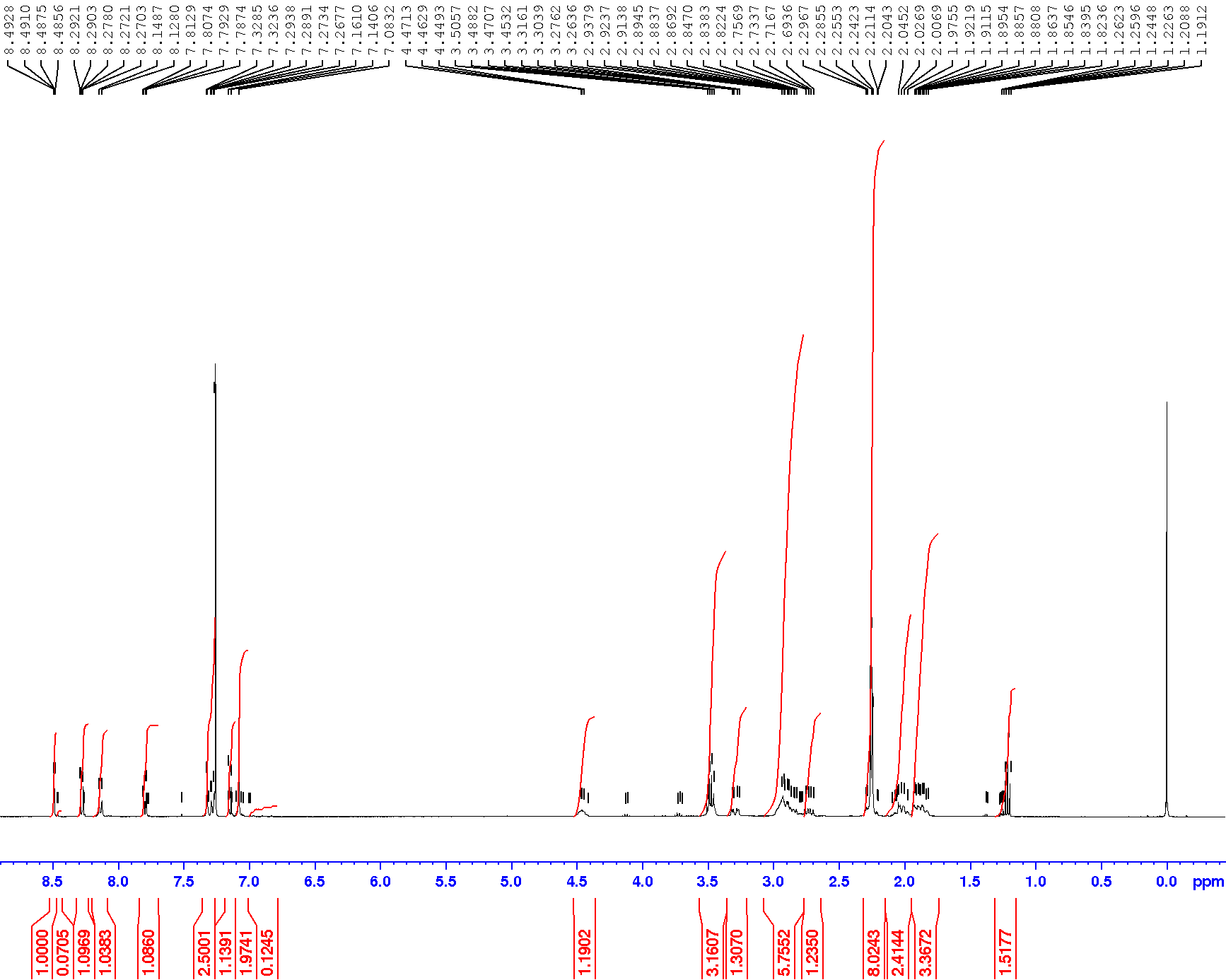


Compound **66**


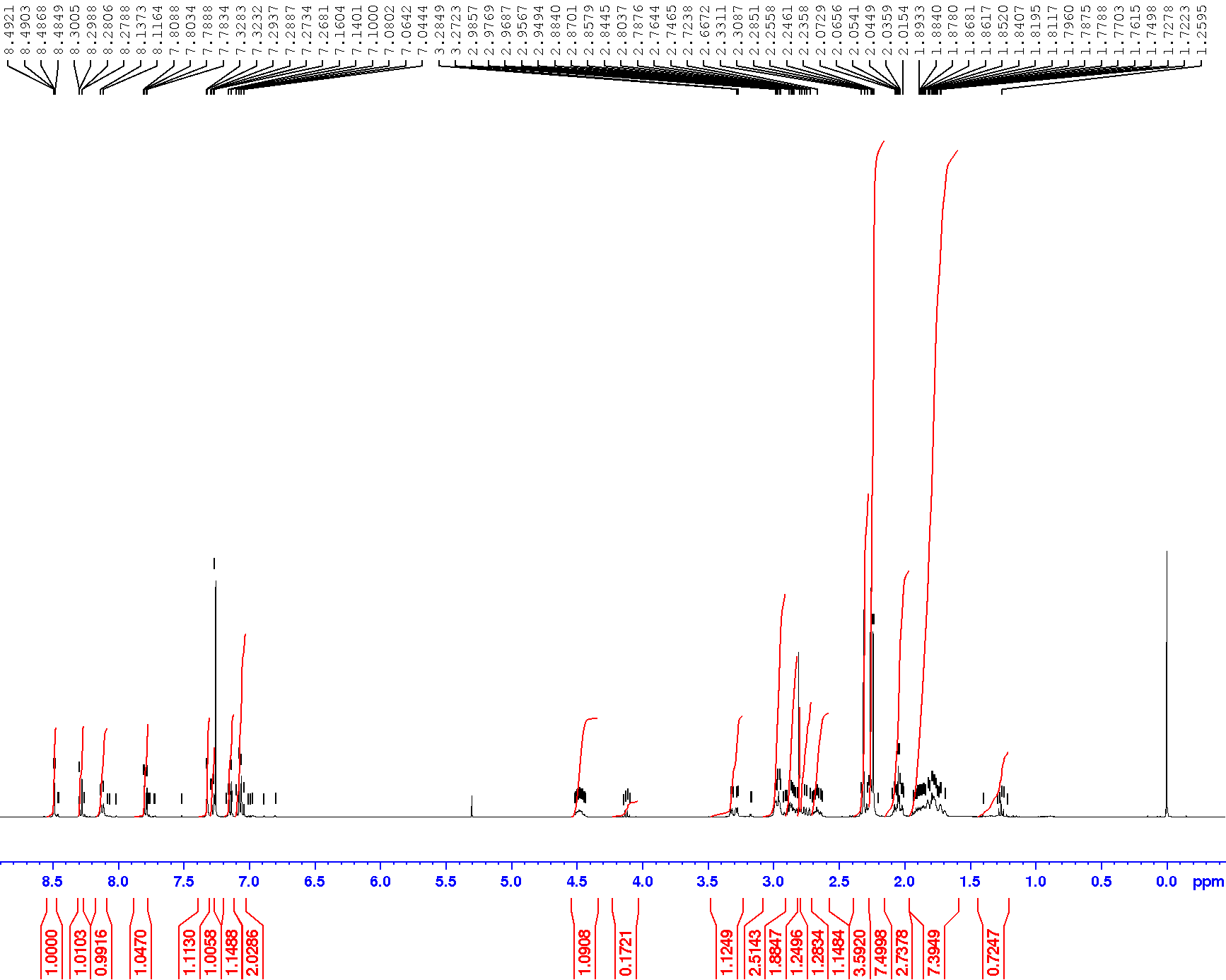


Compound **67**


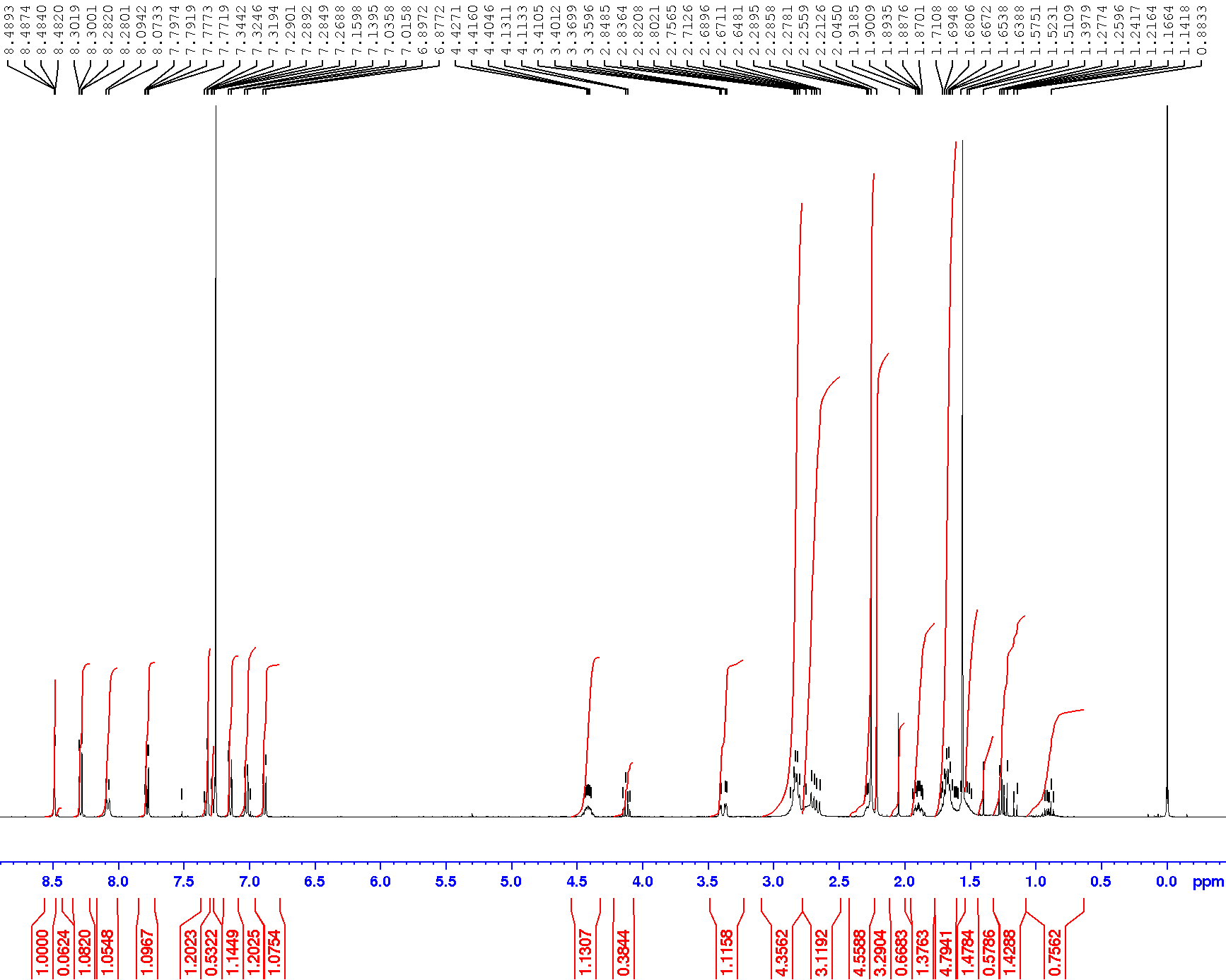


Compound **68**


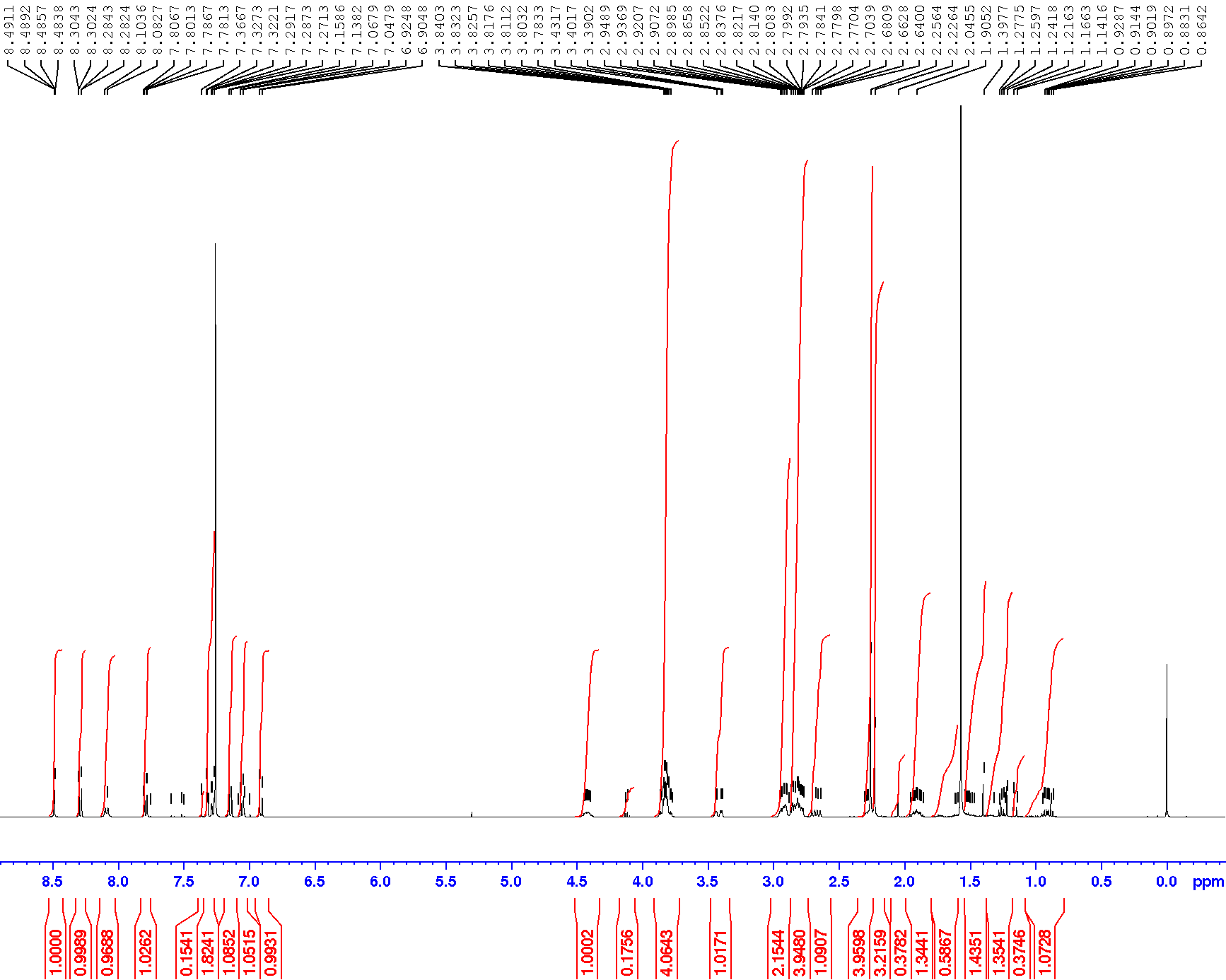


Compound **69**


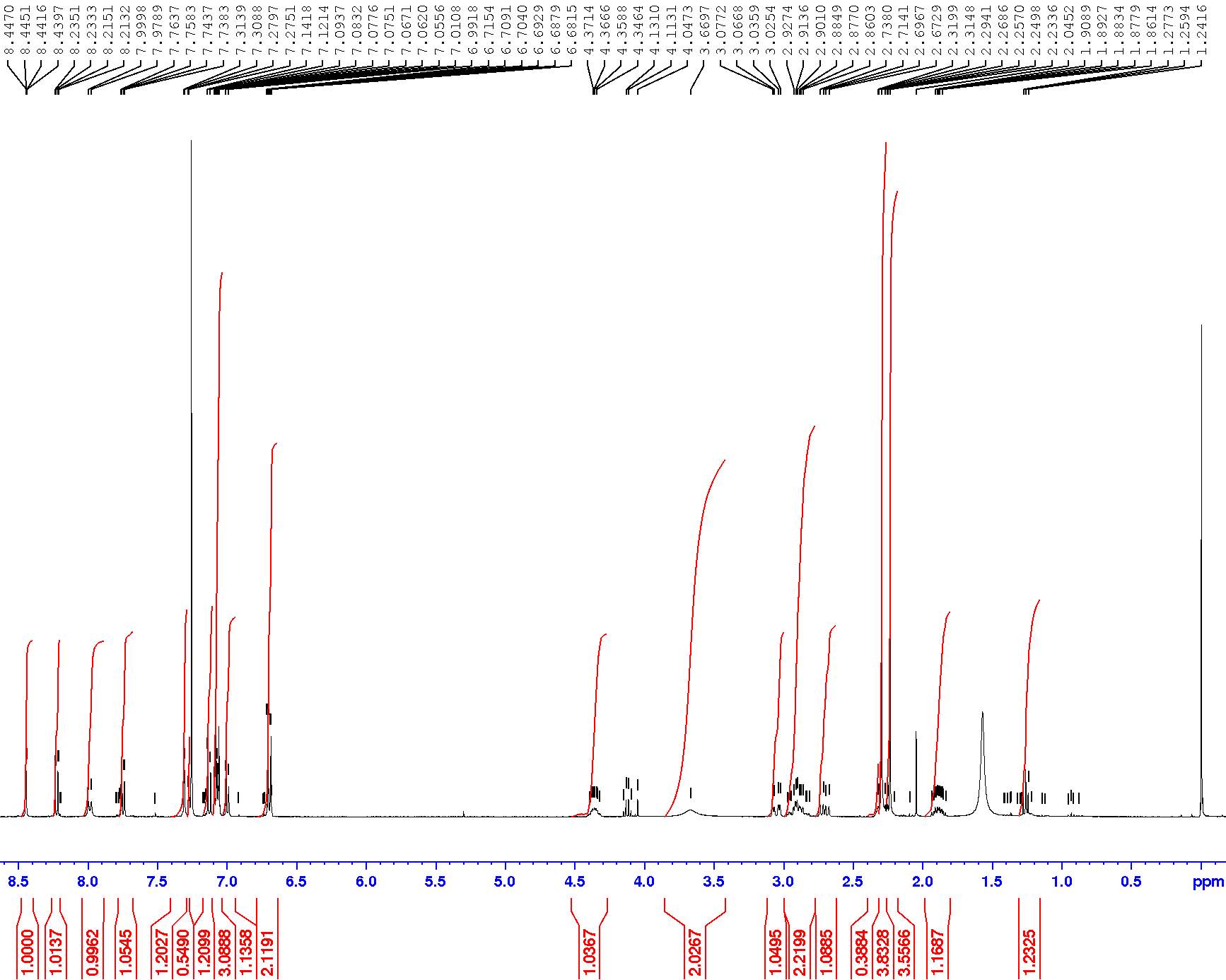


Compound **70**


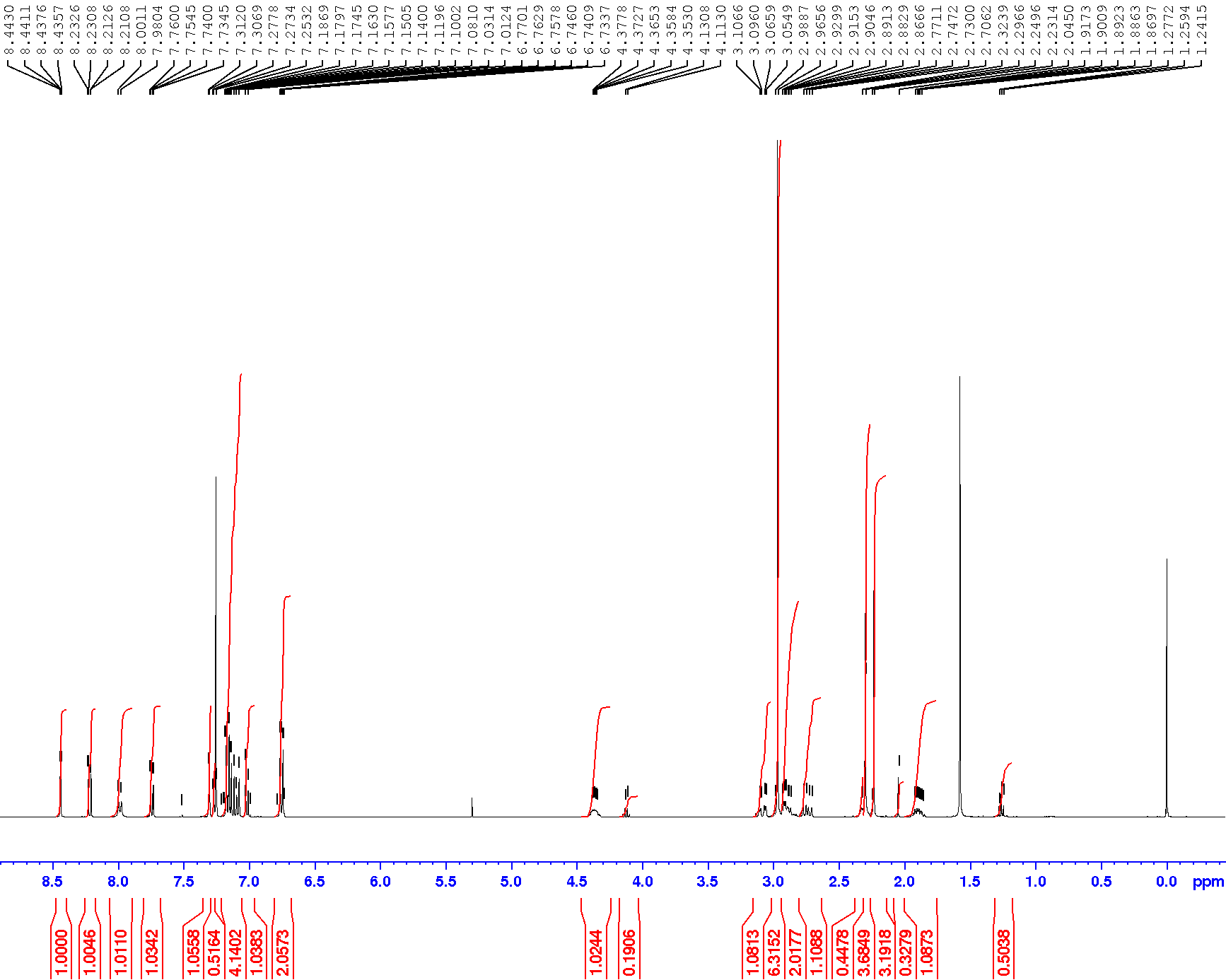


Compound **71**

Compound **72**

Compound **73**


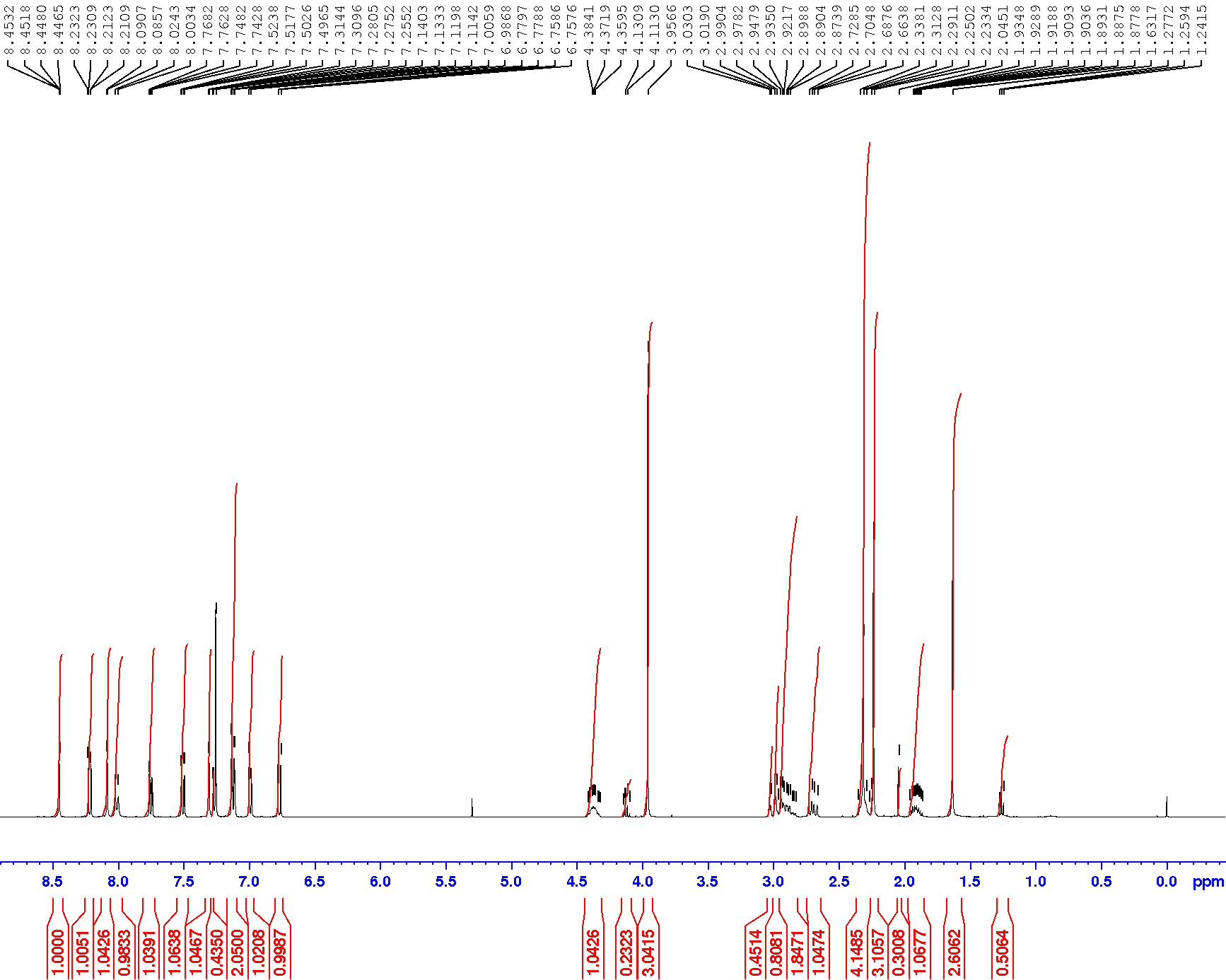


Compound **74**


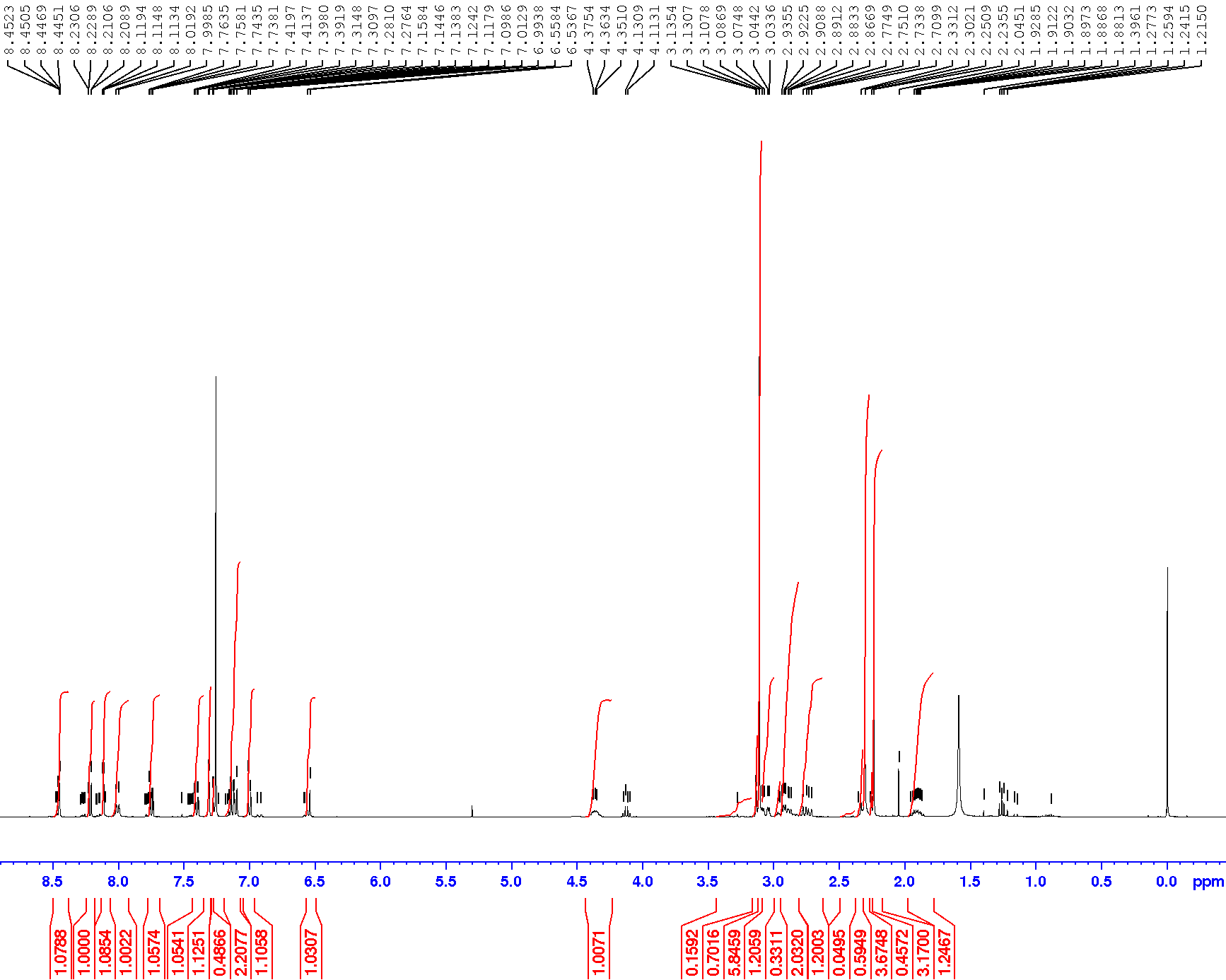


Compound **75**


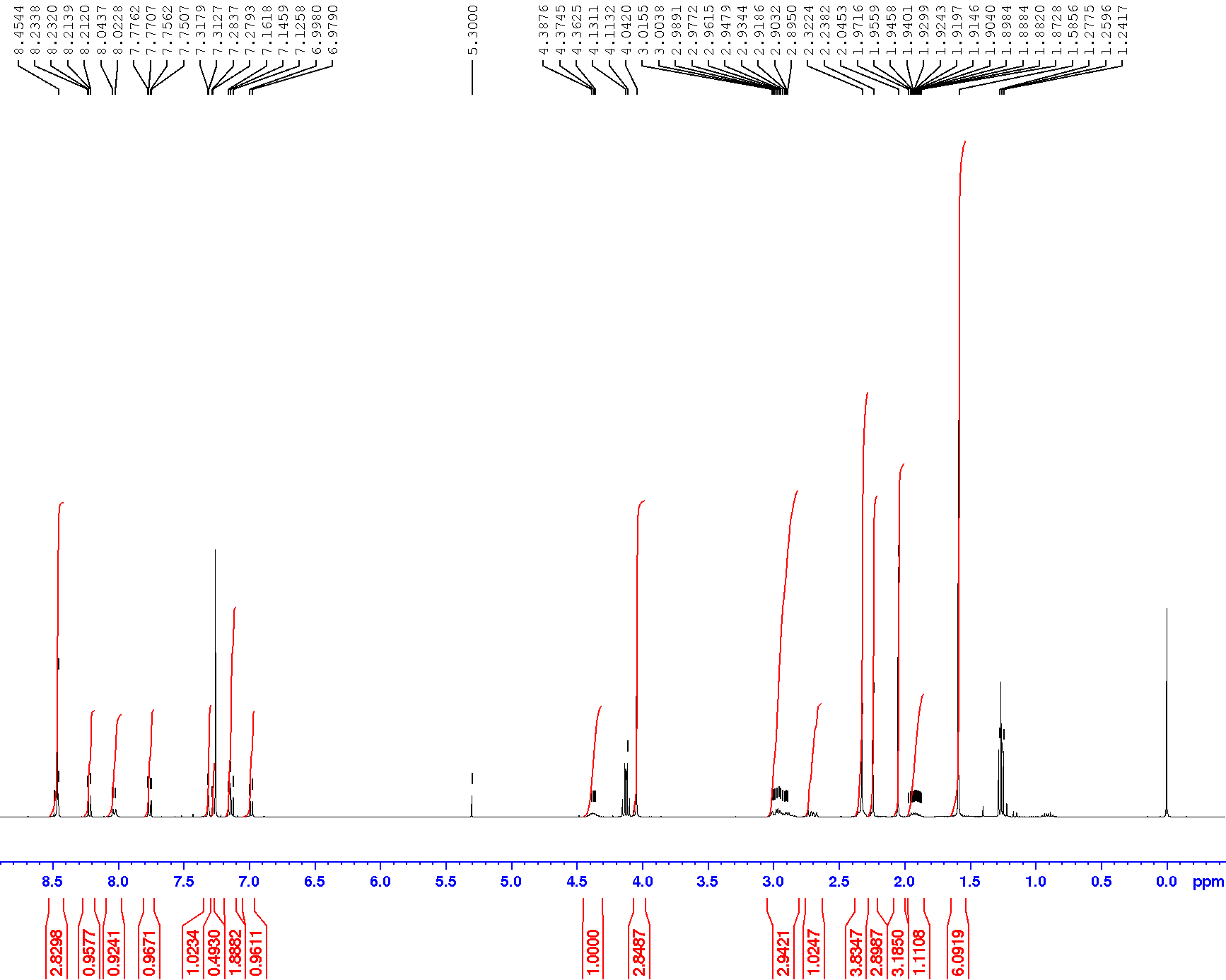


Compound **76**


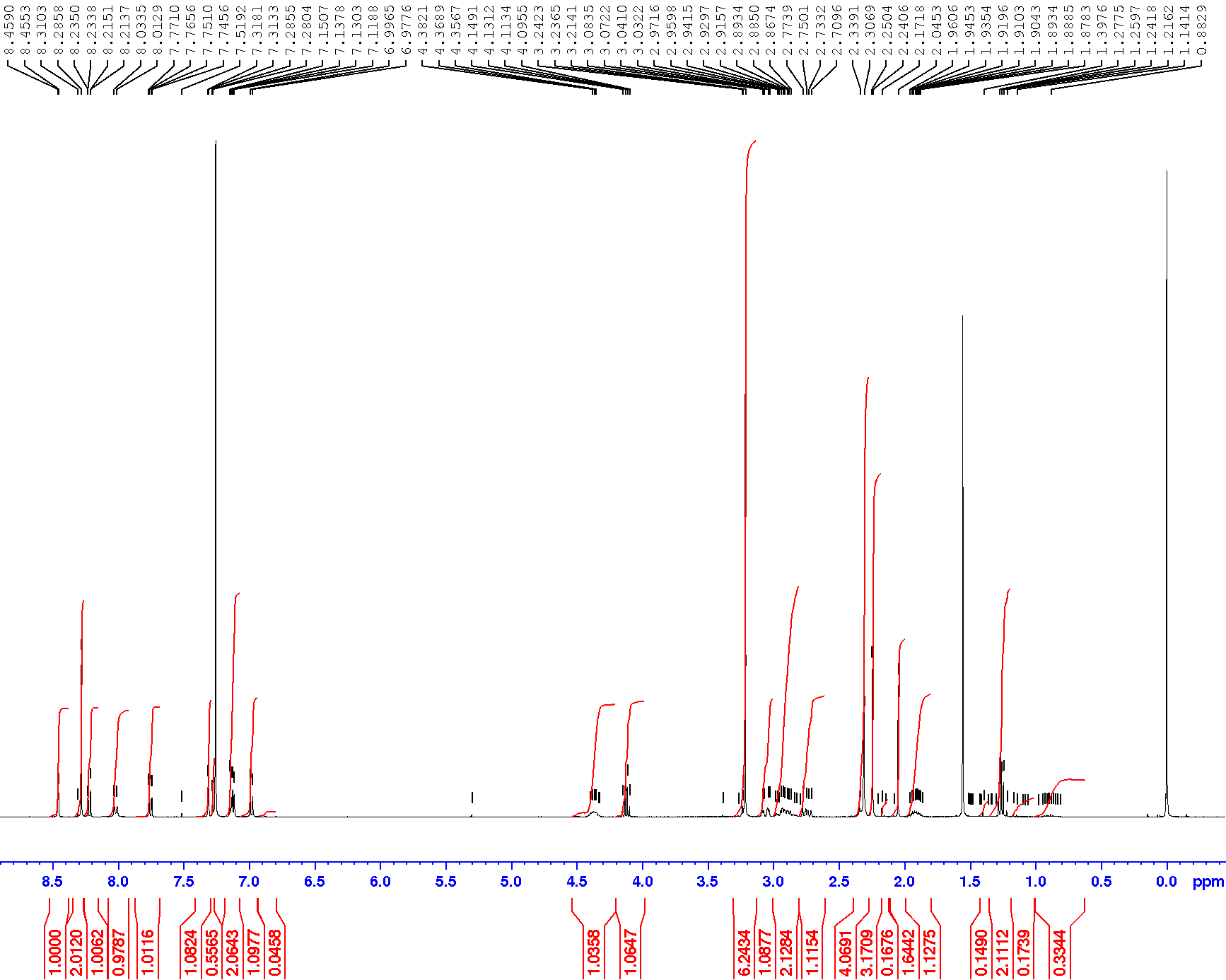


Compound **77**

Compound **78**


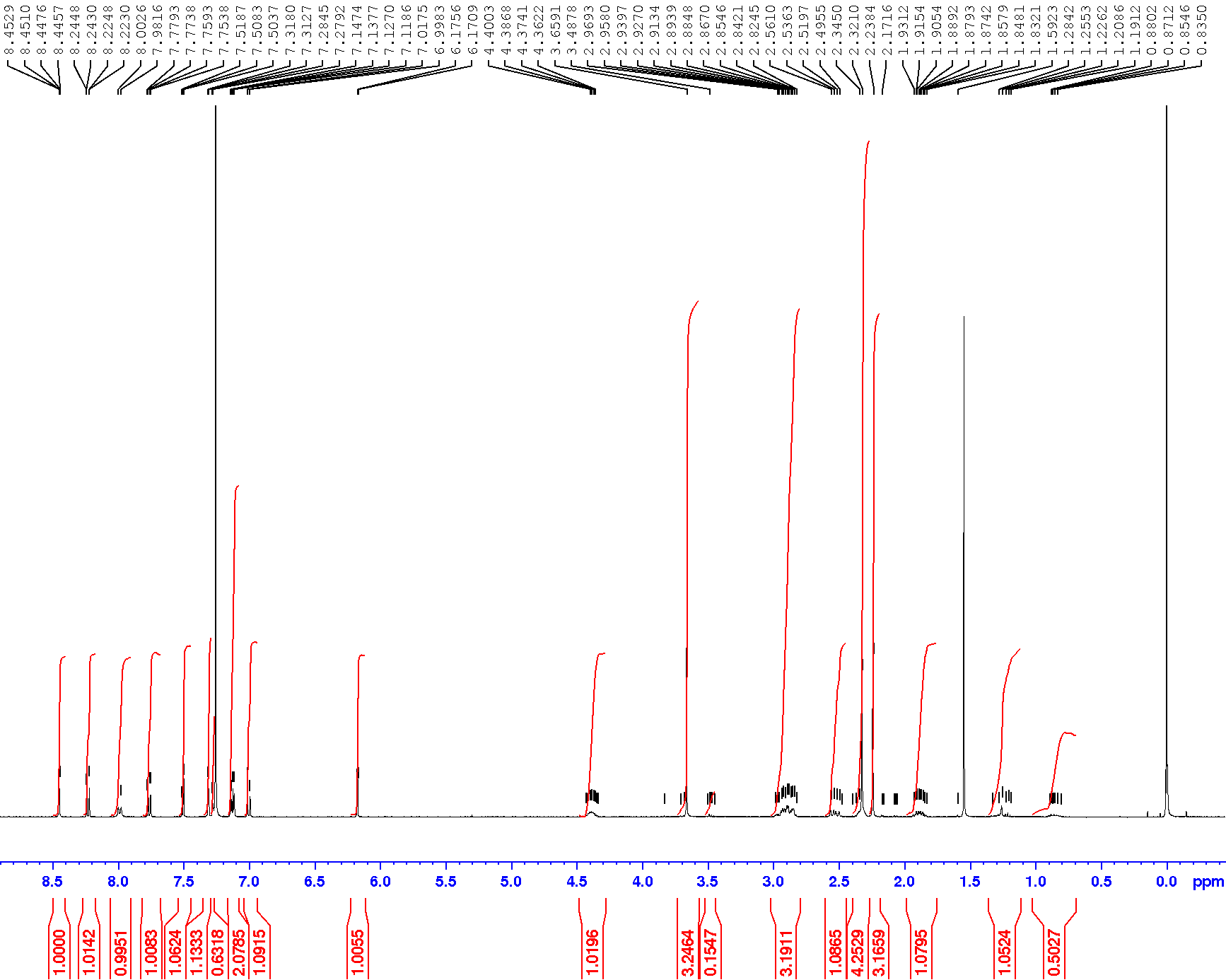


Compound **79**
